# Transition‐Metal‐Free Coupling of Polyfluorinated Arenes and Functionalized, Masked Aryl Nucleophiles

**DOI:** 10.1002/chem.202101731

**Published:** 2021-06-10

**Authors:** Lucie Finck, Martin Oestreich

**Affiliations:** ^1^ Institut für Chemie Technische Universität Berlin Strasse des 17. Juni 115 10623 Berlin Germany

**Keywords:** biaryls, chemoselectivity, Lewis bases, nucleophilic aromatic substitution, silicon

## Abstract

A chemoselective C(sp^2^)−C(sp^2^) coupling of sufficiently electron‐deficient fluorinated arenes and functionalized *N*‐aryl‐*N’*‐silyldiazenes as masked aryl nucleophiles is reported. The fluoride‐promoted transformation involves the in situ generation of the aryl nucleophile decorated with various sensitive functional groups followed by a stepwise nucleophilic aromatic substitution (S_N_Ar). These reactions typically proceed at room temperature within minutes. This catalytic process allows for the functionalization of both coupling partners, furnishing highly fluorinated biaryls in good yields.

## Graphical abstract

**A fluoride‐promoted**, stepwise nucleophilic aromatic substitution of silicon‐based aryl pronucleophiles decorated with sensitive functional groups and polyfluorinated arenes enables the synthesis of partially fluorinated biaryls. The mild transformation operates at room temperature, requiring less than one hour for completion. Hence, the functional‐group compatibility is superb, and regioselectivities are improved compared to known couplings.

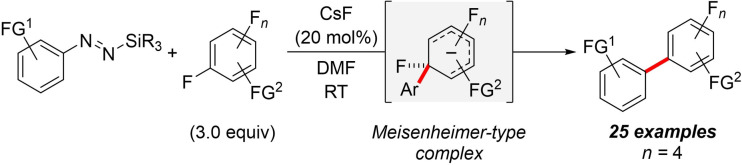

Polyfluorinated biaryls have been identified as versatile and robust building blocks possessing interesting properties relevant for agrochemicals, pharmaceuticals, and materials science.[^1^, ^2^] Hence, the development of viable synthetic strategies for their preparation through C(sp^2^)−C(sp^2^) bond‐forming reactions continues to attract significant interest.^[3]^ Typical approaches to access this motif engage transition‐metal catalysts (Scheme 1, top left).[^4^, ^5^, ^6^, ^7^] Among them, conventional cross‐coupling^[4]^ and oxidative direct arylation^[5]^ generally require the prefunctionalization of a coupling partner while cross‐dehydrogenative coupling^[6]^ involves non‐prefunctionalized arenes. These transformations normally operate at elevated temperature and require long reaction times. As a result, the chemoselectivity of these methods can be deteriorated, and sensitive functional groups poorly tolerated, especially on the electron‐deficient aromatic ring. Also, transition‐metal‐free approaches have been far less explored.[^8^, ^9^, ^10^, ^11^] Radical couplings have been identified as a valuable alternative (Scheme 1, top right).^[8]^ Recently, König and co‐workers disclosed a photocatalytic process involving formal C−H bond activation that proceeds at 40 °C yet requiring 72 h.^[9]^ While the polyfluorinated arylbromide as the other component is readily available, a large excess (20 equiv) was necessary and the regio‐ and chemoselectivity were moderate.

**Scheme 1:**
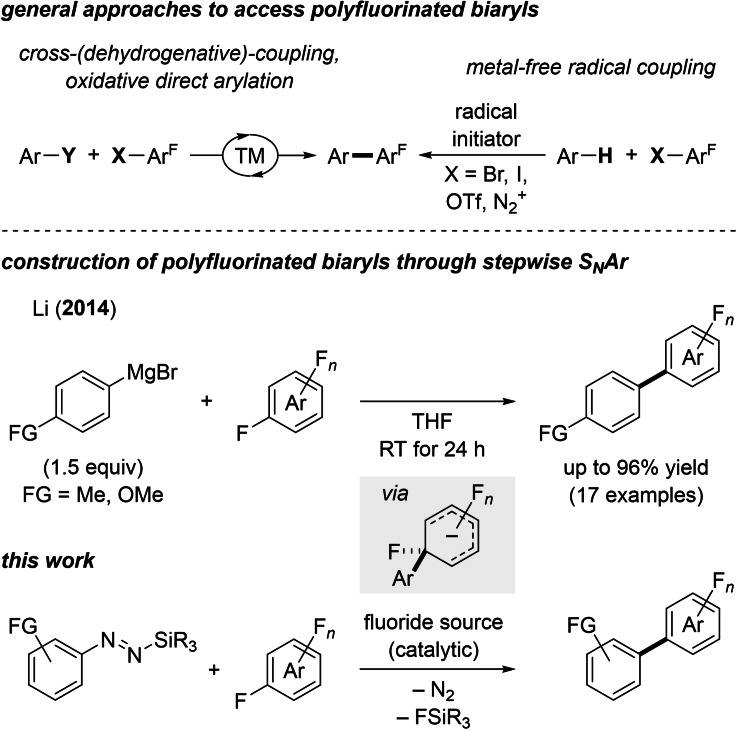
Synthetic methodologies for the preparation of polyfluorinated biaryls through C(sp^2^)−C(sp^2^) bond‐forming reactions. Ar=(hetero)aryl group, Ar^F^=polyfluorinated (hetero)aryl group, TM=transition metal, X and Y=(pro)nucleophile or (pseudo)halogen or H, FG=functional group, R=alkyl or aryl group, *n*=4–9.

Alternatively, these synthetically useful biaryls can be prepared by S_N_Ar at the electron‐deficient fluorinated arene with main‐group organometallic reagents. This approach formerly found application in materials science making use of organolithium reagents.^[12]^ However, this strategy suffers from the lack of control in the degree of substitution, leading to mixtures of mono‐ and polyarylated fluoroarenes.^[10]^ Li and co‐workers addressed this shortcoming by employing Grignard reagents, yet with a limited functional‐group tolerance on each aromatic ring, possibly due to the high reactivity of these polar reagents (Scheme 1, middle).^[11]^ Our laboratory has been studying a new class of kinetically stable and easily accessible silicon‐based aryl pronucleophiles: *N*‐aryl‐*N*’‐silyldiazenes.^[13]^ Upon initiation with a silaphilic Lewis base in catalytic amounts, highly reactive aryl alkali metal reagents are generated in situ.^[14]^ In this context, we considered that these versatile pronucleophiles would be a suitable alternative to displace the strong C(sp^2^)−F bond in sufficiently electron‐deficient fluoroarenes through a stepwise S_N_Ar (Scheme 1, bottom).^[15]^ We herein report a transition‐metal‐free coupling reaction of functionalized aryl nucleophiles and polyfluoroarenes through the intermediacy of stabilized Meisenheimer‐type intermediates (Scheme 1, gray box).

We began our investigation by optimizing the fluoride‐promoted coupling of the 4‐tolyl‐substituted diazene **1 a** and hexafluorobenzene (**2 a**) at room temperature (Table 1). Guided by our previous work,^[14]^ an initial experiment was performed using a catalytic amount of CsF with 18‐crown‐6 as an additive in THF, affording the polyfluorinated biaryl **3 aa** after 2 h in 77 % yield (entry 1). Owing to the three‐fold excess of **2 a** employed, only a minor amount of a regioisomeric mixture of terphenyl **4 a** formed. As expected, reducing the amount of the fluoroarene **2 a** resulted in an increase of bisarylated **4 a** (see the Supporting Information for the complete optimization). Moreover, diazene **1 a** is prone to degradation into the corresponding arylsilane **5 a** upon activation with a Lewis base (see the Supporting Information for details). To prevent this competitive reaction, a catalytic system without 18‐crown‐6 was tested. The “less naked” fluoride ion considerably improved the selectivity of the reaction in favor of the desired coupling product, yet at a prolonged reaction time (entry 2). With these promising results as a starting point, we examined the influence of the solvent. It is known that the solubility of alkali metal fluorides is generally enhanced in DMF compared to other commonly used polar aprotic solvents.^[16]^ The reaction conducted in DMF effectively exhibited both a good selectivity and an improved reaction kinetics without additives, affording the coupling product **3 aa** in excellent yield in less than five minutes (entry 3). Additionally, various fluoride‐based catalysts were probed, albeit CsF had proven to be a competent catalyst initiating the N−Si bond cleavage.^[17]^ Alkali metal salts provided slow reaction kinetics (entry 4) or hardly any conversion (entries 5 and 6), likely owing to their insolubility.^[16]^ In turn, the more soluble tetraalkylammonium fluorides rapidly mediated the reaction. While TBAT afforded **3 aa** in good yield (entry 7), TMAF furnished an inferior yield resulting from competing reactions (entry 8). Similarly, the trimethylsilanolate salt KOSiMe_3_ (generating the poorly soluble KF in situ) successfully promoted the reaction albeit with slower reaction rate (entry 9). It is important to mention that the latter initiator can potentially engage in S_N_Ar with polyfluoroarenes.^[18]^ Ultimately, a control experiment confirmed that there is no reaction in the absence of the initiator (entry 10).

**Table 1:** Selected examples of the optimization of the fluoride‐promoted preparation of polyfluorinated biaryls.^[a]^

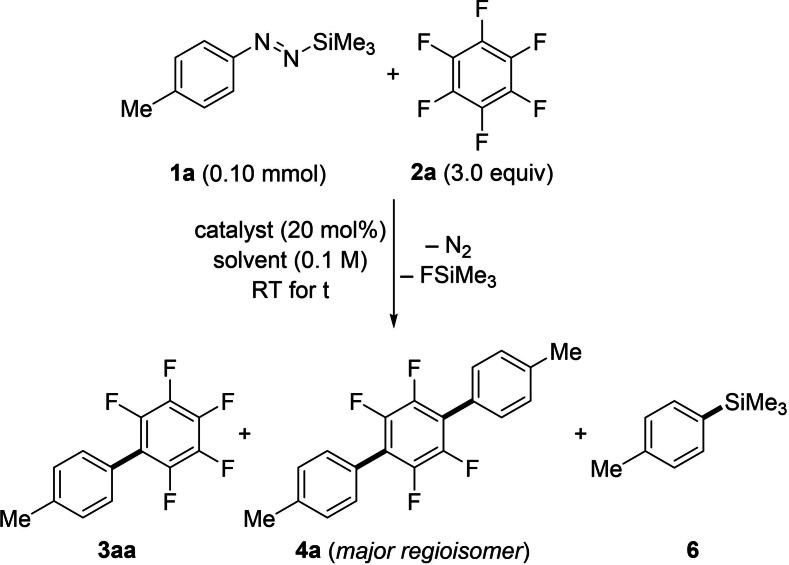					
Entry	Catalyst	Solvent	Time	Yield [%]^[b]^	
				**3 aa 5a**	**6**
1	CsF/18‐C‐6^[c]^	THF	2 h	77	15
2	CsF	THF	48 h	91	5
3	CsF	DMF	<5 min	92 (79)^[d]^	7
4^[e]^	KF	DMF	24 h	57	5
5^[e]^	NaF	DMF	24 h	trace	trace
6^[e]^	LiF	DMF	24 h	trace	trace
7	TBAT	DMF	<5 min	89	7
8	TMAF	DMF	<5 min	62	16
9^[e]^	KOSiMe_3_	DMF	24 h	30	3
10^[e]^	none	DMF	24 h	0	0

[a] All reactions were performed on a 0.10 mmol scale in 0.7 mL (0.1 M) of the indicated solvent. [b] Determined by calibrated GLC analysis with tetracosane as an internal standard. [c] CsF/18‐crown‐6 (1.0 : 1.2 molar ratio). [d] Yield of isolated product on a 0.30 mmol scale after flash chromatography on silica gel in parentheses. [e] Incomplete conversion of **1 a**. TBAT=tetrabutylammonium difluorotriphenylsilicate, TMAF=tetramethylammonium fluoride.

With optimized reaction conditions in hand (Table 1, entry 3), we explored the substrate scope of this transformation (Schemes 2 and 3). We started with investigating the coupling reaction of various aryl‐substituted silyldiazenes **1 a**–**o** and hexafluorobenzene (**2 a**) at room temperature (Scheme 2). It is noteworthy that the addition of 18‐crown‐6 was required for a few substrates that otherwise exhibited a poor reactivity. A broad range of electron‐donating and ‐withdrawing functional groups were compatible with our method, affording the coupling products within a few minutes in high yields. Diazenes bearing sensitive substituents such as cyano (as in **1 d**), methoxycarbonyl (as in **1 e**), nitro (as in **1 f**), and trifluoromethyl (as in **1 k**) were effectively transferred. A larger excess of fluoroarene **2 a** was necessary to provide **3 ka** in 80 % yield due to the competing degradation of the diazene **1 k** into the cognate arylsilane. Coupling products **3 ga**–**ja** with halogen substituents could be successfully accessed. Competitive halogen–metal exchange was observed for both iodo‐ and bromo‐substituted aryl nucleophiles **1 g** and **1 h**, respectively; the corresponding 1,4‐dihalogenated benzene derivatives and 1,4‐bis(pentafluorophenyl)benzene were found in small quantities (see the Supporting Information for details). Additionally, the *ortho*‐fluoro‐substituted diazene **1 l** reacted equally well by delivering **3 la** in 70 % yield; the possible β‐elimination giving the corresponding aryne intermediate was not seen. Increasing the steric bulk in the *ortho*‐positions as in **1 m** and **1 n** was not detrimental. Even the bisdiazene **1 p**, equivalent to an aryl bisnucleophile, smoothly underwent the S_N_Ar to furnish the valuable building block **3 pa**.^[19]^

**Scheme 2:**
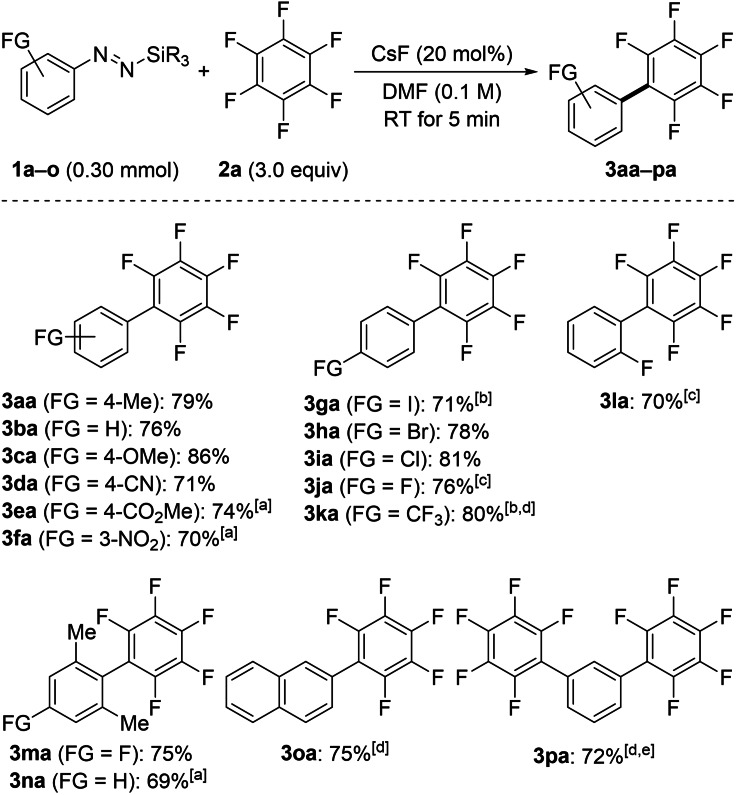
Scope I: Coupling of various functionalized silylated aryldiazenes with hexafluorobenzene (**2 a**). All reactions were performed on a 0.30 mmol scale at room temperature. Yields are isolated yields after flash chromatography on silica gel. [a] CsF/18‐crown‐6 (1.0 : 1.2 molar ratio). [b] 10 equiv of C_6_F_6_ (**2 a**) used. [c] The reaction time was 3 h. [d] The reaction time was 1 h. [e] 20 equiv of C_6_F_6_ (**2 a**) used. R=Me, Et.

**Scheme 3:**
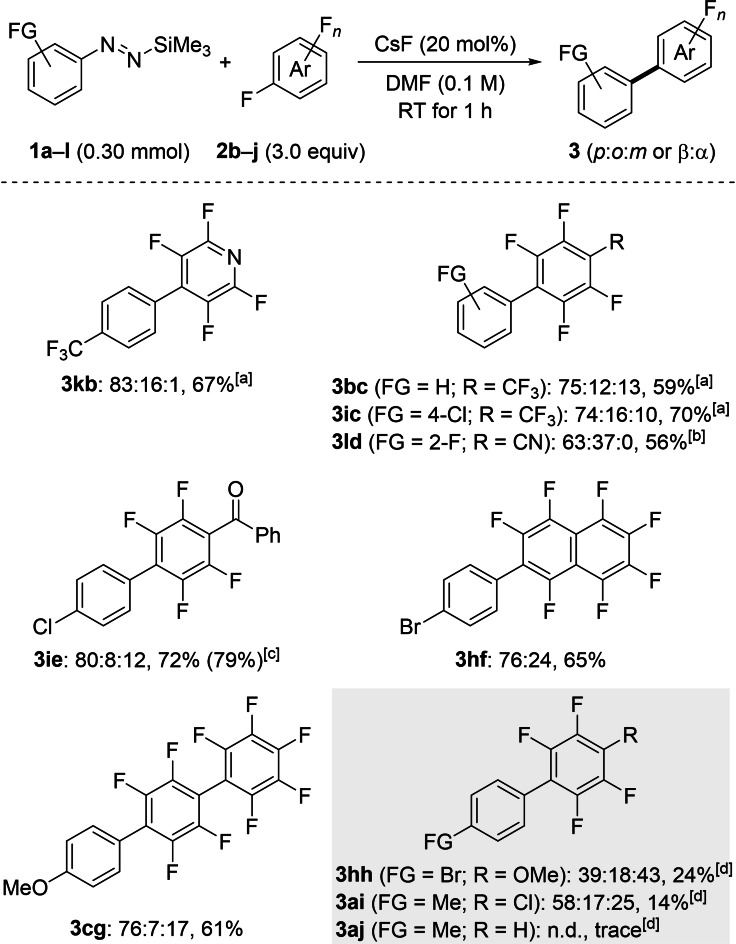
Scope II: Coupling of various silylated aryldiazenes with functionalized polyfluorarenes. All reactions were performed on a 0.30 mmol scale at room temperature. Regioisomeric ratios (*para* : *ortho* : *meta* or β : α) were determined by quantitative ^19^F NMR analysis with hexafluorobenzene as an internal standard. Yields are isolated yields of *para* (β for **3 hf**) regioisomers after purification. [a] 10 equiv of the polyfluorarene used. [b] The reaction time was 12 h. [c] Yield of isolated product on a 1.30 mmol scale after flash chromatography on silica gel in parentheses. [d] Yield determined by quantitative ^19^F NMR analysis with hexafluorobenzene as an internal standard. n.d.=not determined.

To further illustrate the chemoselectivity of our method we achieved the coupling reaction of various aryl‐substituted silyldiazenes with functionalized polyfluoroarenes **2 b**–**j** (Scheme 3). The construction of such biaryls possessing functional‐group diversity on both coupling partners is not trivial, and existing strategies for their preparation are scarce. Considering that the transformation furnishes a mixture of regioisomers,^[20]^ our primary intention was to investigate the influence of the solvent on the regioisomeric distribution. To this end, we screened various solvents for the coupling of pentafluoropyridine (**2 b**) and diazene **1 k** (see the Supporting Information for details). Accordingly, we established that the coupling performed in DMF proceeded with a good level of regiocontrol while it did not in ethereal solvents. All the reactions were therefore conducted in DMF to produce the *para*‐ or β‐substituted regioisomer, respectively with good to moderate selectivities; the products were isolated in a regioisomerically pure form after flash chromatography on silica gel. The pyridine derivative **2 b** was hence successfully coupled with diazene **1 k** to provide **3 kb** in 67 % yield. The trifluoromethyl group was also compatible on the electron‐deficient ring, furnishing **3 bc** and **3 ic**. Pentafluorobenzonitrile (**2 d**) exhibited a moderate regioselectivity, likely due to the increased electrophilic character of the *ortho*‐position arising from the neighbouring cyano group.^[20]^ Competing arylation of the carbonyl group in benzophenone derivative **2 e** did not occur, and the functionalized coupling product **3 ie** was obtained in excellent yield and with good regioselectivity. In line with these findings, polyfluorinated naphtalene **2 f** and biphenyl **2 g** displayed a high reactivity and satisfying selectivity. The steric congestion around the *ortho*‐position of biphenyl **2 g** exerted a non negligible effect on the regioselectivity of the transformation as the S_N_Ar was partially steerd towards the usually less favorable *meta*‐position.

Although our method displayed good chemoselectivity and regiocontrol, a few substrates were not suitable (Scheme 3, gray box). The less reactive pentafluoroanisole (**2 h**) reacted poorly, and the diazene **1 h** instantaneously decomposed into the corresponding arylsilane. Chloropentafluorobenzene (**2 i**) underwent mainly halogen–metal exchange, leading to oligomeric mixtures of chloroperfluoropolyphenyls.^[21]^ As a result of the high basicity of the in situ released aryl nucleophile, pentafluorobenzene (**2 j**) was deprotonated and further decomposed (see the Supporting Information for details). Similarly, 1,2‐difluorobenzene underwent *ortho*‐formylation through deprotonation followed by addition to the solvent (not shown, see the Supporting Information for details). Nucleophilic addition of the aryl nucleophile to DMF had never been observed with our system before.^[14a]^

To demonstrate the synthetic utility of the coupling products, difficult‐to‐make partially fluorinated biaryls **3 ck** and **3 dk** containing a styrene unit were prepared and further derivatized to *trans*‐stilbene derivatives (Scheme 4). Accordingly, pentafluorostyrene (**2 k**) reacted chemoselectively with the aryl pronucleophiles **1 c** and **1 d** in good yields under the standard reaction conditions. The electronic properties of the substituents attached to the diazene (OMe for **1 c** and CN for **1 d**) had no effect on the regioselectivity (Scheme 4, top). Compounds **3 ck** and **3 dk** were subsequently converted into the partially fluorinated stilbene derivatives **7 cka** and **7 dkb** by a Heck reaction in high yields (Scheme 4, gray box).

**Scheme 4:**
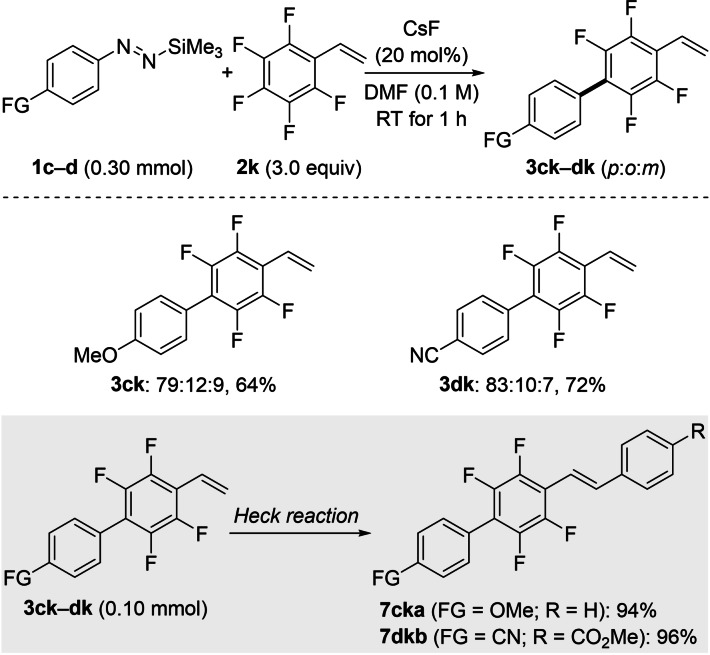
Scope III and IV: Construction of polyfluorinated biaryls having a styrene unit (top) and further diversification of coupling products by a Heck reaction (bottom). Regioisomeric ratios (*para* : *ortho* : *meta*) were determined by quantitative ^19^F NMR analysis with hexafluorobenzene as an internal standard. Yields are isolated yields of *para* regioisomers after purification. Reaction conditions for the Heck reaction: iodobenzene (**6 a**, 1.5 equiv, for **3 ck**) or methyl 4‐iodobenzoate (**6 b**, 1.5 equiv, for **3 dk**), Pd(OAc)_2_ (2.0 mol%), Et_3_N (5.0 equiv), DMF, 100 °C, 20 h.

To summarize, a reliable method for the preparation of partially fluorinated, functionalized biaryls proceeding through an S_N_Ar has been developed. This strategy relies on the fluoride‐initiated, in situ release of aryl nucleophiles decorated with sensitive substituents from readily available *N*‐aryl‐*N*’‐silyldiazenes.^[13]^ Compared to the prior art, the new transition‐metal‐free protocol operates under mild reaction conditions and requires short reaction times, thereby securing excellent functional group tolerance.

## Conflict of interest

The authors declare no conflict of interest.

## Supporting information

As a service to our authors and readers, this journal provides supporting information supplied by the authors. Such materials are peer reviewed and may be re‐organized for online delivery, but are not copy‐edited or typeset. Technical support issues arising from supporting information (other than missing files) should be addressed to the authors.

Supporting Information
