# Supplementary material for: Transition‐Metal‐Free Coupling of Polyfluorinated Arenes and Functionalized, Masked Aryl Nucleophiles
Source: Chemistry. 2021 Jun 10;27(43):11061–4. doi: 10.1002/chem.202101731 (PMC8453572; doi:10.1002/chem.202101731)
Supplement: Supplementary file 1 — Supporting Information [file CHEM-27-11061-s001.pdf]

Chemistry–A European Journal

Supporting Information

Transition-Metal-Free Coupling of Polyfluorinated Arenes and Functionalized, Masked Aryl Nucleophiles

Lucie Finck and Martin Oestreich*

Table of Contents

1 General Information S2

2 Optimization Study S5

2.1 Optimization of the Fluoride-Catalyzed Synthesis of Polyfluorinated Biaryls S5

2.2 Influence of the Solvent on the Regioselectivity of the Coupling S7

3 Experimental Details for the Preparation of *N*-Aryl-*N’*-Silyldiazenes S9

3.1 Synthesis of (*E*)-1-(4-iodophenyl)-2-(trimethylsilyl)diazene (**1g**) S9

3.2 Synthesis of (*E*)-1-(4-(trifluoromethyl)phenyl)-2-(trimethylsilyl)diazene (**1k**) S12

3.3 Synthesis of (*E*)-1-(4-fluoro-2,6-dimethylphenyl)-2-(trimethylsilyl)diazene (**1m**) S13

4 General Procedures S17

4.1 General Procedure for the Coupling of Diazenes with Hexafluorobenzene (GP1) S17

4.2 General Procedure for the Coupling of Diazenes with Polyfluoroarenes (GP2) S17

5 Characterization Data for the Polyfluorinated Biaryls S19

6 Scale-Up Experiment S38

7 Derivatization of the Coupling Products – Experimental Details S39

8 Control Experiments S41

8.1 Degradation of the *N*-Aryl-*N’*-Silyldiazenes in the Absence of Polyfluoroarene S41

8.2 Competitive Deprotonation – Failed Substrates S42

9 NMR Spectra S43

9.1 *N*-Aryl-*N’*-Silyldiazenes S43

9.2 Polyfluorinated Biaryls S67

9.3 Diversified Coupling Products S145

10 References S151

1 General Information

All reactions were performed using standard Schlenk techniques or in an *MBraun* glovebox, respectively under a static pressure of nitrogen or argon, unless otherwise stated. Glassware for reactions outside a glovebox was flame-dried under vacuum using a heat gun. Glassware for reactions performed inside a glovebox was either flame-dried under vacuum using a heat gun or dried overnight in a 160 °C oven before being transferred into the glovebox. Plastic syringes, needles and septa were dried overnight in a 60 °C oven before being transferred into the glovebox. All reactions were stirred with magnetic followers. All stated temperatures refer to external bath temperatures. For the addition of liquid reagents and solvents through silicon/rubber septa, argon- or nitrogen-flushed disposable syringes and needles were used. All glass syringes and stainless steel needles were used several times and stored at 120 °C. Solids were added in a countercurrent of inert atmosphere or in solution. Low-temperature reactions were either cooled by an ice bath, acetone/dry ice bath, or by using cryostats EK90 from *Haake* or TC100E-F from *Huber*. Compound names were generated by the computer program *ChemDraw* according to the guidelines specified by the International Union of Pure and Applied Chemistry (IUPAC).

Reagents and solvents

Standard reagents and solvents were obtained from *ABCR*, *Acros Organics*, *Alfa Aesar*, *Carbolution*, *Merck*, *Sigma-Aldrich*, or *Tokyo Chemical Industry* (TCI) and used as received. Alkali metal fluoride salts (CsF, KF, NaF, LiF) were dried and stored in a glovebox prior to use. Potassium trimethylsilanolate ($KOSiMe_3$) was sublimed under high vacuum and stored in a glovebox prior to use. The *N*-aryl-*N'*-silyldiazenes were prepared according to a previously reported procedure and stored in a glovebox.[S1] Dichloromethane (CH_2Cl_2), *n*-pentane and triethylamine (Et_3N) were dried over CaH_2 and freshly distilled under nitrogen atmosphere prior to use. Tetrahydrofuran (THF), 1,4-dioxane, toluene and diethyl ether (Et_2O) were dried over sodium with benzophenone as indicator and freshly distilled under nitrogen atmosphere prior to use. *N*-Methyl-2-pyrrolidone (NMP), *N*,*N*-dimethylformamide (DMF), *N*,*N'*-dimethylpropylene urea (DMPU), dimethyl sulfoxide (DMSO), and dimethoxyethane (DME) were purchased from *Acros* (99.8%, extra dry, over molecular sieves, AcroSeal®) and used as received. For extraction, flash chromatography and recrystallization, technical grade solvents (*tert*-butyl methyl ether, cyclohexane, *n*-pentane, dichloromethane, diethyl ether, isopropanol and ethyl acetate) were distilled prior to use. All solvents and liquid reagents used in a glovebox were distilled, degassed by the freeze-pump-thaw method, and stored in a glovebox over thermally activated 4 Å molecular sieves. Brine refers to a saturated solution of NaCl in deionized water.

Chromatography

Qualitative **thin-layer chromatography** (TLC) was performed on *Macherey-Nagel* Alugram® Xtra SIL G/UV_{254} silica gel 60 pre-coated aluminum-backed plates (200 µm layer thickness). Product spots were visualized under UV light (λ_{max} = 254 nm), by staining with a ceric ammonium molybdate solution and/or by staining with a potassium permanganate solution.

Flash column chromatography was performed on silica gel Davisil LC60A (grain size 40–63 µm, pore size 60 Å, 230–400 mesh ASTM) from *Grace GmbH* according to the method reported by W.C. Still and coworkers.[S2]

Analytical **gas-liquid chromatography** (GLC) of the reaction mixtures and pure substances were performed using a *Varian* 430-GC gas chromatograph equipped with a *Varian* Factor Four Capillary column (30 m × 0.25 mm, 0.25 µm film thickness of the stationnary phase). All GLC analyses were performed by using the following conditions: carrier gas: N_2; injector temperature: 250 °C; detector temperature: 250 °C; flow rate: 4 mL/min; temperature program: starting temperature: 40 °C, heating rate: 10 °C/min, final temperature: 250 °C for 10 min.

Spectroscopy

Nuclear Magnetic Resonance (NMR)

^{1}H, ^{2}H, ^{13}C, ^{15}N, ^{19}F, and ^{29}Si NMR spectra were recorded in $CDCl_3$, C_6D_6, CD_2Cl_2, DMSO-d_6, THF-d_8 or DMF-d_7 on AV 400, AV 500 or AV 700 instruments from *Bruker* at *Institut für Chemie*, *Technische Universität Berlin*, with the deuterated solvent acting as an internal deuterium lock. ^{1}H NMR spectra were recorded at 400, 500 or 700 MHz, ^{2}H NMR spectra at 77 MHz, ^{13}C NMR spectra at 100 or 126 MHz, ^{15}N spectra at 51 MHz, ^{19}F spectra at 471 MHz, and ^{29}Si spectra at 99 MHz, using broadband proton decoupling when indicated. The ^{1}H and ^{13}C chemical shifts are reported in parts per million (ppm), and NMR spectra are referenced to the residual protic solvent resonances and the deuterated solvent carbon resonances, respectively ($CHCl_3$: δ = 7.26 ppm for ^{1}H NMR and $CDCl_3$: δ = 77.16 ppm for ^{13}C NMR, C_6D_5H: δ = 7.16 ppm for ^{1}H NMR and C_6D_6: δ = 128.1 ppm for ^{13}C NMR, $CDHCl_2$: δ = 5.32 ppm for ^{1}H NMR and CD_2Cl_2: δ = 53.84 ppm for ^{13}C NMR, DMSO-d_5: δ = 2.50 ppm for ^{1}H NMR and DMSO-d_6: δ = 39.52 ppm for ^{13}C NMR, THF-d_7: δ = 1.72, 3.58 ppm for ^{1}H NMR and THF-d_8: δ = 25.31, 67.21 ppm for ^{13}C NMR, DMF-d_6: δ = 2.75, 2.92, 8.03 ppm for ^{1}H NMR and DMF-d_7: δ = 29.76, 34.89, 163.2 ppm for ^{13}C NMR).[S3] For all other nuclei, the NMR resonance signals were internally calibrated according to the IUPAC recommendation, using a unified chemical shift scale based on the proton resonance of tetramethylsilane as primary reference.[S4] Chemical shifts are reported relative to tetramethylsilane to the 0.01 ppm for ^{1}H NMR spectra and to the 0.1 ppm for ^{13}C, ^{19}F and ^{29}Si NMR spectra. Coupling constants are quoted to the nearest 0.1 Hz for ^{1}H NMR spectra and to full Hz for ^{13}C and ^{19}F NMR spectra. Data are reported as follows: chemical

shift, multiplicity (s = singlet, d = doublet, t = triplet, q = quartet, quint = quintet, m = multiplet, m_c = centrosymmetric multiplet, br = broad, app = apparent, and combinations thereof), coupling constants (Hz), and integration. Air- and moisture- sensitive samples were measured in J. Young NMR tubes. THF-d_8 and DMF-d_7 were degassed and stored over activated 4 Å molecular sieves prior to use.

Infrared (IR) spectra were recorded on a Cary 630 FT-IR spectrometer from *Agilent Techologies* equipped with a diamond ATR unit. Infrared spectra of silyl diazenes were recorded in a glovebox using a *Thermo Nicolet Magna-IR* 750 spectrophotometer equipped with an ATR unit. Selected absorption maxima are reported in wavenumbers (cm^{-1}).

High Resolution Mass Spectrometry (HRMS) measurements were performed at the analytical facilities of the *Institut für Chemie, Technische Universität Berlin* with an LTQ Orbitrap XL using atmospheric-pressure chemical ionization (APCI), electrospray ionization (ESI) or liquid injection field desorption/ionization (LIFDI) methods. The in-detail fragmentation was omitted and only the molecular ion peak or characteristic molecular fragments are considered.

Physical Data

Melting Points (M.p.) were determined using a melting-point-determination apparatus *Leica* Galen III hot-stage microscope from *Wagner & Munz*. The values are not corrected.

Boiling Points (B.p.) were measured at the distillation head and are not corrected. Distillation under reduced pressure was determined directly by the connected pressure gauge (VAP 5, from *Vacuumbrand*).

2 Optimization Study

2.1 Optimization of the Fluoride-Catalyzed Synthesis of Polyfluorinated Biaryls

General procedure for the optimization reactions. In an argon-filled glovebox, an oven-dried 1.5-mL screw-caped vial equipped with a magnetic stirring bar was charged with the indicated amount of catalyst and hexafluorobenzene (**2a**, 55.8 mg, 0.300 mmol, 3.0 equiv). The reaction vessel was sealed, transferred out of the glovebox, and connected to a nitrogen manifold using a syringe needle. The indicated dry solvent (0.35 mL) was added, and to the vigorously stirred mixture was added in one portion a solution of (*E*)-1-(*p*-tolyl)-2-(trimethyl-silyl)diazene (**1a**, 19.2 mg, 0.100 mmol, 1.0 equiv) in the indicated dry solvent (0.35 mL) at room temperature. Full conversion of the silylated aryldiazene was monitored by GLC analysis. Upon completion, a known amount of tetracosane in toluene (0.5 mL) was added, the resulting mixture was stirred for 5 min, and an aliquot of the reaction mixture was subjected to GLC analysis.

Note. Coupling reactions engaging alkali metal fluorides KF, NaF and LiF as catalysts were additionally conducted with their related crown ethers 18-crown-6, 15-crown-5 and 12-crown-4, respectively (1.0:1.2 molar ratio). Reaction rates were nonetheless not significantly improved. Moreover, both lower (Table S1, entry 14) and higher (Table S1, entry 15) loadings of catalyst resulted in diminished yields.

Table S1. Optimization of the fluoride-catalyzed preparation of polyfluorinated biaryls.[a]

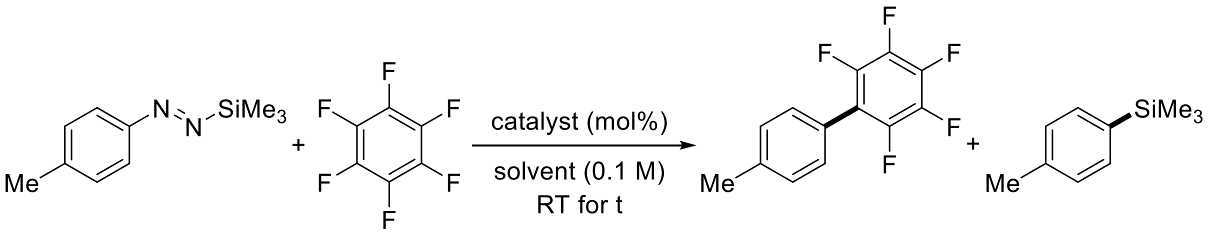

1a (0.10 mmol) **2a** (3.0 equiv) **3aa** **5a**

entry	catalyst	mol%	solvent	time	yield **3aa** [%][b]	yield **5a** [%][b]
1[c]	CsF	20	toluene	48 h	trace	trace
2[c]	CsF	20	CH_2Cl_2	48 h	0	0
3	CsF	20	THF	48 h	91	5
4[d]	CsF/18-C-6	20	THF	2 h	77	15
5	CsF	20	DMF	< 5 min	92 (79)[e]	7
6[d]	CsF/18-C-6	20	DMF	< 5 min	86	8
7[c]	KF	20	DMF	24 h	57	5
8[c]	NaF	20	DMF	24 h	trace	trace
9[c]	LiF	20	DMF	24 h	trace	trace
10	TMAF	20	DMF	< 5 min	62	16
11	TBAT	20	DMF	< 5 min	89	7
12[c]	$KOSiMe_3$	20	DMF	24 h	30	3
13[c]	none	–	DMF	24 h	0	0
14	CsF	10	DMF	< 5 min	78	9
15	CsF	100	DMF	< 5 min	60	6
16[f]	CsF	20	DMF	< 5 min	84	11
17[g]	CsF	20	DMF	< 5 min	62	18
18[h]	CsF	20	DMF	< 5 min	82	8

[a] Unless otherwise noted, reactions were performed on a 0.10 mmol scale in 0.7 mL (0.1 M) of the indicated solvent. [b] Determined by calibrated GLC analysis with tetracosane as an internal standard. [c] Incomplete conversion of **1a**. [d] CsF/18-crown-6 (1.0:1.2 molar ratio). [e] Yield of isolated product on a 0.30 mmol scale after flash chromatography on silica gel in parentheses. [f] 2.0 equivalents of C_6F_6 (**2a**). [g] 1.0 equivalent of C_6F_6 (**2a**). [h] 0.5 mL (0.1 M) of the indicated solvent. TBAT = tetrabutylammonium difluorotriphenylsilicate, TMAF = tetramethylammonium fluoride.

2.2 Influence of the Solvent on the Regioselectivity of the Coupling

General procedure for the solvent screening. In an argon-filled glovebox, an oven-dried 1.5-mL screw-caped vial equipped with a magnetic stirring bar was charged with cesium fluoride (CsF, 3.0 mg, 20 µmol, 20 mol%) and pentafluoropyridine (**2b**, 50.7 mg, 0.300 mmol, 3.0 equiv). The reaction vessel was sealed, transferred out of the glovebox, and connected to a nitrogen manifold using a syringe needle. The indicated dry solvent (0.35 mL) was added, and to the vigorously stirred mixture was added in one portion a solution of (*E*)-1-(4-(tri-fluoromethyl)phenyl)-2-(trimethylsilyl)diazene (**1k**, 24.6 mg, 0.100 mmol, 1.0 equiv) in the indicated dry solvent (0.35 mL) at room temperature. Full conversion of the silylated aryldiazene was monitored by GLC analysis. Upon completion, the crude mixture was concentrated under reduced pressure, and the crude residue was diluted in $CDCl_3$ (0.8 mL). A known amount of hexafluorobenzene was added, and the resulting mixture was stirred for 5 min. The solution was then transferred into a NMR tube and analyzed straightaway by quantitative ^{19}F NMR spectroscopy. For a more precise integration of ^{19}F NMR spectra, relaxation delays were set to 50 sec.

Note. The *meta*-substituted regioisomer was formed in trace amount and was therefore omitted from the table.

Table S2. Influence of the solvent on the regioselectivity of the coupling.[a]

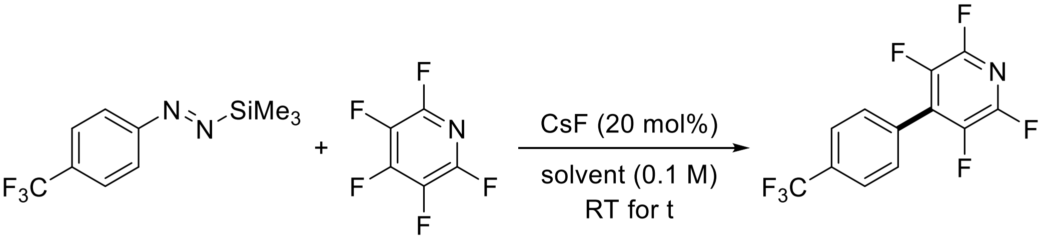

1k (0.10 mmol) **2b** (3.0 equiv) **3kb**

entry	solvent	time	ratio *para*:*ortho* [%][b]	yield *para*:*ortho* [%][b]
1	DMF	1 h	83:17	54:12
2	DMA	1 h	82:18	47:10
3	NMP	1 h	84:16	38:7
4	DMPU	1 h	n.d.	trace
5	THF	20 h	67:33	59:29
6	2-MeTHF	48 h	n.d.	trace
7	1,4-dioxane	24 h	60:40	54:36
8	DME	7 h	76:24	61:19
9[c]	toluene	24 h	71:29	24:10
10[d]	CH_2Cl_2	48 h	n.d.	trace
11	CH_3CN	1 h	n.d.	trace
12	DMSO	1 h	n.d.	trace
13[e]	DMF	1 h	83:17	75(67):15(14)[f]

[a] Unless otherwise noted, reactions were performed on a 0.10 mmol scale in 0.7 mL (0.1 M) of the indicated solvent. [b] Product ratios and conversions were determined by quantitative ^{19}F NMR analysis with hexafluorobenzene as an internal standard. [c] CsF/18-crown-6 (1.0:1.2 molar ratio). [d] Incomplete conversion of **1k**. [e] 10 equivalents of **2b**. [f] Yield of isolated product on a 0.30 mmol scale after purification in parentheses.

Note. In most instances lower yields are explained by the overarylation of the fluoroarene and/or by the formation of the aryl silane $ArSiMe_3$ (Ar = 4-$CF_3C_6H_4$) originating from the degradation of the diazene.

3 Experimental Details for the Preparation of *N*-Aryl-*N'*-Silyldiazenes

All additional *N*-aryl-*N'*-silyldiazenes were prepared according to previously reported procedures.[S1,S5]

3.1 Synthesis of (*E*)-1-(4-iodophenyl)-2-(trimethylsilyl)diazene (**1g**)

2-(4-Iodophenyl)hydrazin-1-ium chloride (S1)

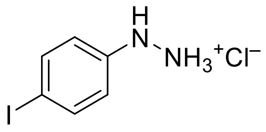

S1
$C_6H_8ClIN_2$
M = 270.50 g/mol

In accordance with modified literature procedures,[S6] a 250-mL flask equipped with a large olive-shaped magnetic stirring bar was charged with anhydrous tin chloride ($SnCl_2$, 19.0 g, 100 mmol, 2.5 equiv) and concentrated hydrochloric acid (100 mL). The resulting solution was stirred at room temperature to ensure complete dissolution of $SnCl_2$.
In parallel, a 500-mL three-necked flask equipped with an overhead mechanical stirrer, a dropping funnel and an internal thermometer was charged with the solid grinded 4-iodoaniline (8.76 g, 40.0 mmol, 1.0 equiv) followed by hydrochloric acid (6 M, 100 mL). The resulting suspension was vigorously stirred for 20 min at –5 °C. A solution of sodium nitrite ($NaNO_2$, 3.31 g, 48.0 mmol, 1.2 equiv) in water (20 mL) was then added dropwise to the reaction mixture over 30 min (keeping internal reaction temperature below –5 °C). The obtained diazonium salt solution was further stirred at –5 °C for 1 h, and the $SnCl_2$ solution was subsequently added dropwise with vigorous stirring over 30 min (keeping internal reaction temperature below –5 °C). The resulting mixture was further stirred for 1 h at room temperature.
The resulting off-white suspension was filtered over a fritted funnel, and the filter cake was washed with isopropanol followed by Et_2O. The obtained hydrazinium salt was further dried under high *vacuum* (10^{-2} mbar) for 15 h (the crude product was protected from light with an aluminium foil). The title compound **S1** (8.05 g, 29.8 mmol, 75%) was obtained as a light brown powder and was used for the next step without further purification.

M.p.: 205–207 °C (diethyl ether). **^{1}H NMR** (700 MHz, $(CD_3)_2SO$): δ/ppm = 6.82 (m_c, 2H), 7.59 (m_c, 2H), 8.47 (bs, 1H), 10.4 (bs, 3H). **^{13}C{1H} NMR** (126 MHz, $(CD_3)_2SO$): δ/ppm = 83.9, 116.8 (2C), 137.3 (2C), 145.4. **^{1}H/^{15}N HMQC NMR** (700/71 MHz, $(CD_3)_2SO$, optimized for J = 90.0 Hz): δ/ppm = 8.47/85.5, 10.4/65.7. **HRMS** (APCI) calculated for $C_6H_8IN_2^+$ [(M–Cl)$^+$]: 234.9727; found: 234.9728. **IR** (ATR): $\tilde{\nu}$/cm^{-1} = 3229, 3073, 2866, 2674, 2258, 1543, 1508, 1482, 1399, 1308, 1236, 1185, 1113, 1080, 1062, 1025, 1004, 876, 820, 759, 704.

1-(4-Iodophenyl)-2-(trimethylsilyl)hydrazine (S2)

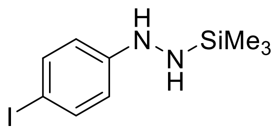

S2
$C_9H_{15}IN_2Si$
M = 306.22 g/mol

In accordance with a modified literature procedure,[S1] a 250-mL flame-dried Schlenk flask equipped with a magnetic stirring bar and a rubber septum was charged with the solid hydrazinium salt **S1** (7.93 g, 29.3 mmol, 1.0 equiv) followed by Et_2O (140 mL, 0.2 M) at room temperature. The resulting suspension was vigorously stirred and cooled to –15 °C. 1,8-Diazabicyclo[5.4.0]undec-7-ene (DBU, 1.5 mL, 10 mmol, 0.34 equiv) and triethylamine (Et_3N, 8.6 mL, 62 mmol, 2.1 equiv) were then successively added dropwise over 10 min. To the resulting mixture was dropwise added trimethylchlorosilane (4.5 mL, 35 mmol, 1.2 equiv) over 5 min. The obtained suspension was vigorously stirred for 4 h at –15 °C. The crude reaction mixture was then filtered over a fritted funnel and the filtrate was concentrated under reduced pressure at room temperature to afford the crude silylated hydrazine which was further evacuated under high *vacuum* (10^{-2} mbar) for 1 h. The title compound **S2** (7.76 g, 25.3 mmol, 86%) was obtained as a pale brown solid and was engaged in the subsequent oxidation step without any further purification.

M.p.: 97–100 °C (diethyl ether). **^{1}H NMR** (500 MHz, C_6D_6): δ/ppm = –0.02 (s, 9H), 2.18 (bs, 1H), 4.29 (bs, 1H), 6.29 (d, J = 8.6 Hz, 2H), 7.41 (d, J = 8.6 Hz, 2H). **^{13}C{1H} NMR** (126 MHz, C_6D_6): δ/ppm = –1.15 (3C), 79.7, 114.8 (2C), 137.8 (2C), 151.8. **^{1}H/^{29}Si HMQC NMR** (500/99 MHz, C_6D_6, optimized for J = 7.0 Hz): δ/ppm = –0.02/5.80. **^{1}H/^{15}N HMQC NMR** (500/51 MHz, C_6D_6, optimized for J = 90.0 Hz): δ/ppm = 2.18/61.1, 4.29/86.2. **HRMS** (APCI) calculated for $C_9H_{14}IN_2Si^+$ [(M–H)$^+$]: 304.9965; found: 304.9972. **IR** (ATR): $\tilde{\nu}$/cm^{-1} = 3335, 3292, 2952, 2894, 1878, 1585, 1482, 1303, 1244, 1091, 996, 810.

(*E*)-1-(4-Iodophenyl)-2-(trimethylsilyl)diazene (1g)

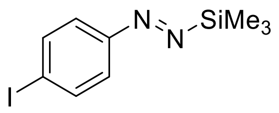

1g
$C_9H_{13}IN_2Si$
M = 304.21 g/mol

In accordance with a modified literature procedure,[S1] a single-necked 250-mL flask equipped with a magnetic stirring bar was charged in a glovebox with the silylated arylhydrazine **S2** (3.98 g, 13.0 mmol, 1.0 equiv) followed by dry hexamethyldisiloxane (60 mL, 0.2 M). To the resulting vigorously stirred suspension was added solid di-*tert*-butyl azodicarboxylate (DBAD, 2.84 g, 12.3 mmol, 0.95 equiv) portion–wise over 5 min at room temperature. The resulting mixture was further stirred for 2 h at room temperature. The suspension was then placed in a freezer (–35 °C) for 1 h and filtered over a fritted funnel. The blue filtrate was concentrated *in vacuo* to afford the crude silylated aryldiazene, which was then distilled under reduced pressure *via* a short path distillation (**b.p.**: 79–85 °C at 4.1 × 10^{-1} mbar) to afford the title compound **1g** (2.25 g, 7.40 mmol, 57%) as a deep blue solid.

^{1}H NMR (500 MHz, $CDCl_3$): δ/ppm = 0.37 (s, 9H), 7.44 (d, *J* = 8.6 Hz, 2H), 7.84 (d, *J* = 8.6 Hz, 2H). **^{13}C{^{1}H} NMR** (126 MHz, C_6D_6): δ/ppm = –2.74 (3C), 98.6, 122.8 (2C), 138.5 (2C), 155.3. **^{1}H/^{29}Si HMQC NMR** (500/99 MHz, $CDCl_3$, optimized for *J* = 7.0 Hz): δ/ppm = 0.37/13.9. **HRMS** (APCI) calculated for $C_9H_{14}IN_2Si^+$ [(M+H)$^+$]: 304.9965; found: 304.9966. **IR** (ATR): $\tilde{\nu}$/cm^{-1} = 3068, 2958, 2899, 1723, 1569, 1488, 1392, 1301, 1248, 1132, 1050, 1002, 832.

3.2 Synthesis of (*E*)-1-(4-(trifluoromethyl)phenyl)-2-(trimethylsilyl)diazene (**1k**)

1-(4-(Trifluoromethyl)phenyl)-2-(trimethylsilyl)hydrazine (S3)

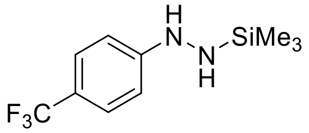

S3
$C_{10}H_{15}F_3N_2Si$
M = 248.32 g/mol

In accordance with a reported literature procedure,[S1] a two-necked flask equipped with a magnetic stirring bar, a rubber septum, and a reflux condenser fitted with a nitrogen inlet was charged with the solid 4-(trifluoromethyl)phenylhydrazine hydrochloride (4.25 g, 20.0 mmol, 1.0 equiv) followed by dry Et_2O (50 mL) at room temperature. The resulting suspension was vigorously stirred and Et_3N (6.7 mL, 48 mmol, 2.4 equiv) was added in one portion. Chlorotrimethylsilane (3.1 mL, 24 mmol, 1.2 equiv) was then added neat over 10 min at room temperature. The resulting suspension was heated to reflux and vigorously stirred for 15 h (the conversion was monitored by ^{1}H NMR analysis) and then cooled to room temperature. The crude reaction mixture was then filtered over a fritted funnel, the filtrate was concentrated *in vacuo* and further evacuated under high vacuum (10^{-2} mbar) for ca. 1 h to remove remaining trace amount of $Et_3N{\cdot}HCl$ by sublimation. The title compound **S3** (4.92 g, 19.8 mmol, 99%) was obtained as a light orange liquid and was used for the next step without further purification.

^{1}H NMR (500 MHz, $CDCl_3$): δ/ppm = 0.12 (s, 9H), 3.02 (bs, 1H), 5.40 (bs, 1H), 6.86 (d, *J* = 8.6 Hz, 2H), 7.41 (d, *J* = 8.6 Hz, 2H). **^{13}C{1H} NMR** (126 MHz, $CDCl_3$): δ/ppm = –1.01 (3C), 111.3 (2C), 120.1 (q, *J* = 32.8 Hz), 125.1 (q, *J* = 270.2 Hz), 126.5 (q, 2C, *J* = 3.8 Hz), 154.5. **^{19}F NMR** (471 MHz, $CDCl_3$): δ/ppm = –61.0 (s, 3F). **^{1}H/^{29}Si HMQC NMR** (500/99 MHz, $CDCl_3$, optimized for *J* = 7 Hz): δ/ppm = –0.12/7.46. **^{1}H/^{15}N HMQC NMR** (500/51 MHz, $CDCl_3$): δ/ppm = 3.02/61.1, 5.40/88.3. **HRMS** (APCI) calculated for $C_{10}H_{16}F_3N_2Si^+$ [(M+H)$^+$]: 249.1029; found: 249.1032. **IR** (ATR): $\tilde{\nu}$/ cm^{-1} = 3359, 2956, 1614, 1522, 1318, 1249, 1156, 1098, 1061, 1005, 872, 829.

(*E*)-1-(4-(Trifluoromethyl)phenyl)-2-(trimethylsilyl)diazene (1k)

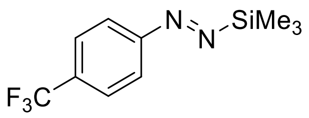

1k
$C_{10}H_{13}F_3N_2Si$
M = 246.31 g/mol

In accordance with a reported literature procedure,[S1] in a glovebox, a 250-mL flask equipped with a magnetic stirring bar was charged with the silylated arylhydrazine **S3** (4.70 g, 18.9 mmol, 1.0 equiv) followed by dry *n*-pentane (50 mL). To the resulting homogeneous, vigorously stirred solution was added portion–wise the solid DBAD (4.14 g, 18.0 mmol, 0.95 equiv) at room temperature. An almost instantaneous color change from yellowish to dark blue was noted with the concomitant precipitation of the hydrazine $(BocNH)_2$. The resulting suspension was further stirred for 1 h at room temperature, and the conversion was monitored by ^{1}H NMR analysis.

The crude reaction mixture was then filtered in a glovebox over a fritted funnel, and the blue filtrate was concentrated *in vacuo* to afford the crude silylated aryldiazene, which was then distilled under reduced pressure *via* a short path distillation (**b.p.**: 36–38 °C at 3.2 × 10^{-1} mbar) affording the title compound **1k** (2.55 g, 10.4 mmol, 55%) as a deep blue liquid.

^{1}H NMR (500 MHz, $CDCl_3$): δ/ppm = 0.40 (s, 9H), 7.77 (m_c, 4H). **^{13}C{1H} NMR** (126 MHz, $CDCl_3$): δ/ppm = –2.61 (3C), 121.0 (2C), 124.2 (q, *J* = 272.5 Hz), 126.4 (q, 2C, *J* = 3.8 Hz), 132.4 (q, *J* = 32.1 Hz), 157.2. **^{19}F NMR** (471 MHz, $CDCl_3$): δ/ppm = –62.5 (s, 3F). **^{1}H/^{29}Si HMQC NMR** (500/99 MHz, $CDCl_3$, optimized for *J* = 7 Hz): δ/ppm = 0.40/14.6. **HRMS** (APCI) calculated for $C_{10}H_{14}F_3N_2Si^+$ [$(M+H)^+$]: 247.0873; found: 247.0871. **IR** (ATR): $\tilde{\nu}$/ cm^{-1} = 2961, 1611, 1517, 1483, 1411, 1319, 1250, 1163, 1118, 1062, 1010, 925, 871, 840.

3.3 Synthesis of (*E*)-1-(4-fluoro-2,6-dimethylphenyl)-2-(trimethylsilyl)diazene (**1m**)

Di-*tert*-butyl 1-(4-fluoro-2,6-dimethylphenyl)hydrazine-1,2-dicarboxylate (S4)

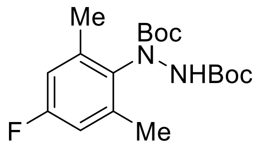

S4
$C_{18}H_{27}FN_2O_4$
M = 354.42 g/mol

In accordance with modified literature procedures,[S7] a flame-dried 500-mL three-necked flask equipped with a magnetic stirrer, a reflux condenser, and a dropping funnel was charged with magnesium turnings (1.26 g, 51.7 mmol, 1.10 equiv), THF (10 mL) and a granule of iodine. A few drops of 2-bromo-5-fluoro-1,3-dimethylbenzene (9.54 g, 47.0 mmol, 1.0 equiv) were subsequently added to the stirred suspension. The formation of the Grignard reagent was observed as the reaction medium became cloudy. The remaining 2-bromo-5-fluoro-1,3-dimethylbenzene in THF (75 mL) was then added to the Grignard reagent solution under vigorous stirring at room temperature. The resulting mixture was further heated to reflux and stirred for 90 min before being cooled to room temperature.

Subsequently, the obtained Grignard reagent solution was added dropwise via a dropping funnel to a solution of DBAD (10.7 g, 46.5 mmol, 0.99 equiv) in THF (80 mL) at –78 °C over 30 min. The obtained mixture was stirred for further 15 min at this temperature. Full conversion of the aryl bromide was monitored by TLC analysis. The reaction was quenched by the

dropwise addition of glacial acetic acid (AcOH, 3.0 mL, 53 mmol, 1.12 equiv) at –78 °C over 5 min. The reaction mixture was subsequently warmed to room temperature, diluted with water (250 mL) and extracted with Et_2O (3 x 150 mL). The combined organic phases were washed with water (250 mL) and brine (250 mL), dried over $MgSO_4$, filtered and concentrated under reduced pressure to afford the title compound **S4** (15.4 g, 43.5 mmol, 93%) as a white solid. The product was engaged in the subsequent step without any further purification.

Carbamate rotamers exist for **S4**.
R_f = 0.62 (*n*-pentane:ethyl acetate 5:1). **M.p.**: 135–137 °C (*n*-pentane). **^{1}H NMR** (400 MHz, $CDCl_3$): δ/ppm = 1.36 (s, 5.5H), 1.46 (s, 5.5H), 1.48 (s, 3H), 1.52 (s, 4H), 2.30–2.39 (m, 6H), 6.58 (bs, 0.5H), 6.65 (bs, 0.5H), 6.70–6.78 (m, 2H). **^{13}C{1H} NMR** (101 MHz, $CDCl_3$): δ/ppm = 18.67 (C_{Ar}–CH_3), 18.73 (C_{Ar}–CH_3), 28.16 (CH$_3$)$_3$), 28.27 (CH$_3$)$_3$), 28.31 (CH$_3$)$_3$), 28.36 (CH$_3$)$_3$), 81.9 (C(CH_3)$_3$), 82.2 (C(CH_3)$_3$), 81.3–81.8 (m, C(CH_3)$_3$), 114.7 (d, J = 22.0 Hz, C_{Ar}–H), 115.0 (d, J = 22.0 Hz, C_{Ar}–H), 135.2 (d, J = 3.1 Hz, $C_{Ar\text{-}quat}$), 135.8 (d, J = 2.9 Hz, $C_{Ar\text{-}quat}$), 138.6 (d, J = 8.8 Hz, $C_{Ar\text{-}quat}$), 139.0–139.3 (m, $C_{Ar\text{-}quat}$), 153.7 (C=O), 154.7 (C=O), 155.5 (C=O), 161.5 (d, J = 246.9 Hz, C–F), 160.7 (d, J = 245.7 Hz, C–F). **^{19}F NMR{1H}** (471 MHz, $CDCl_3$): δ/ppm = –115.4, –115.2. **HRMS** (APCI) calculated for $C_{18}H_{28}FN_2O_4^+$ [(M+H)$^+$]: 355.2028; found: 355.2030. **IR** (ATR): $\tilde{\nu}$/cm^{-1} = 3342, 3289, 2976, 2933, 1746, 1702, 1605, 1478, 1339, 1280, 1147, 1049, 1024, 899, 859, 821, 768.

2-(4-Fluoro-2,6-dimethylphenyl)hydrazin-1-ium chloride (S5)

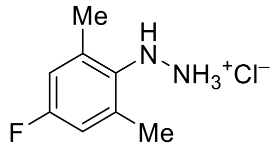

S5
$C_8H_{12}ClFN_2$
M = 190.65 g/mol

In accordance with modified literature procedures,[S7] a flame-dried 250-mL flask equipped with a magnetic stirrer and a nitrogen inlet was charged with the Boc-protected hydrazine **S4** (14.8 g, 41.8 mmol, 1.0 equiv) and absolute ethanol (100 mL). Subsequently, an anhydrous solution of hydrogen chloride (4.0 M in 1,4-dioxane, 52.0 mL, 209 mmol, 5.0 equiv) was added dropwise via syringe over 10 min, and the resulting mixture was stirred at room temperature for 21 h. After removal of volatiles under reduced pressure, the obtained crude solid was triturated with Et_2O (100 mL) and filtered over a fritted funnel. The filter cake was further washed with Et_2O and dried under high vacuum (10^{-2} mbar) for 12 h, affording the hydrazinium salt **S5** (4.89 g,

25.6 mmol, 61%) as a white fluffy solid. The product was engaged in the subsequent step without any further purification.

M.p.: > 230 °C (diethyl ether). **^{1}H NMR** (500 MHz, $(CD_3)_2SO$): δ/ppm = 2.39 (s, 6H), 6.95 (d, *J* = 9.3 Hz, 2H), 7.31 (bs, 1H), 9.70 (bs, 3H). **^{13}C{1H} NMR** (126 MHz, $(CD_3)_2SO$): δ/ppm = 18.0 (2C), 114.6 (d, *J* = 21.3 Hz, 2C), 136.8 (d, *J* = 2.3 Hz), 138.4 (d, *J* = 8.8 Hz, 2C), 160.2 (d, *J* = 244.8 Hz). **^{19}F{1H} NMR** (471 MHz, $(CD_3)_2SO$): δ/ppm = –115.6. **^{1}H/^{15}N HMQC NMR** (500/51 MHz, $(CD_3)_2SO$, optimized for *J* = 90.0 Hz): δ/ppm = 7.31/25.3, 9.70/70.3. **HRMS** (APCI) calculated for $C_8H_{12}FN_2^+$ [(M–Cl)$^+$]: 155.0979; found: 155.0979. **IR** (ATR): $\tilde{\nu}$/cm^{-1} = 3282, 2952, 2906, 2677, 1595, 1550, 1506, 1479, 1308, 1129, 1024, 861, 829, 753, 712.

1-(4-Fluoro-2,6-dimethylphenyl)-2-(trimethylsilyl)hydrazine (S6)

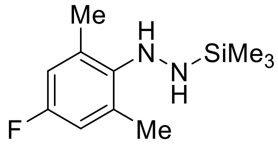

S6
$C_{11}H_{19}FN_2Si$
M = 226.37 g/mol

In accordance with a reported literature procedure,[S1] a two-necked flask equipped with a magnetic stirring bar, a rubber septum, and a reflux condenser fitted with a nitrogen inlet was charged with the solid hydrazinium salt **S5** (4.53 g, 14.9 mmol, 1.0 equiv) followed by dry Et_2O (40 mL) at room temperature. The resulting suspension was vigorously stirred and Et_3N (5.0 mL, 36 mmol, 2.4 equiv) was added in one portion. Chlorotrimethylsilane (2.3 mL, 18 mmol, 1.2 equiv) was then added neat over 10 min at room temperature. The resulting suspension was heated to reflux and vigorously stirred for 15 h (the conversion was monitored by ^{1}H NMR analysis) and then cooled to room temperature. The crude reaction mixture was then filtered over a fritted funnel, the filtrate was concentrated *in vacuo* and further evacuated under high vacuum (10^{-2} mbar) for ca. 1 h to remove remaining trace amount of $Et_3N{\cdot}HCl$ by sublimation. The title compound **S6** (1.89 g, 8.35 mmol, 56%) was obtained as a yellow oil and was used for the next step without further purification.

^{1}H NMR (500 MHz, $CDCl_3$): δ/ppm = 0.07 (s, 9H), 2.32 (s, 6H), 2.92 (bs, 1H), 4.82 (bs, 1H), 6.67 (d, *J* = 9.1 Hz, 2H). **^{13}C{1H} NMR** (126 MHz, $CDCl_3$): δ/ppm = –0.88 (3C), 18.9 (2C), 115.1 (d, 2C, *J* = 21.4 Hz), 130.8 (d, 2C, *J* = 8.0 Hz), 143.2 (d, *J* = 2.2 Hz), 158.3 (d, *J* = 240.1 Hz). **^{19}F{^{1}H} NMR** (471 MHz, $CDCl_3$): δ/ppm = –122.6. **^{1}H/^{29}Si HMQC NMR** (500/99 MHz, $CDCl_3$,

optimized for J = 7 Hz): δ/ppm = 0.07/5.90. **^{1}H/^{15}N HMQC NMR** (500/51 MHz, $CDCl_3$): δ/ppm = 2.92/72.5, 4.82/73.4. **HRMS** (APCI) calculated for $C_{11}H_{19}FN_2Si^+$ [M^+]: 226.1297; found: 226.1293. **IR** (ATR): $\tilde{\nu}$/cm^{-1} = 3332, 2954, 2872, 1605, 1506, 1399, 1219, 1088, 1015, 837.

(*E*)-1-(4-Fluoro-2,6-dimethylphenyl)-2-(trimethylsilyl)diazene (1m)

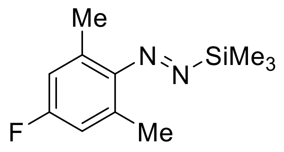

1m
$C_{11}H_{17}FN_2Si$
M = 224.35 g/mol

In accordance with a reported literature procedure,[S1] in a glovebox, a 100-mL flask equipped with a magnetic stirring bar was charged with the silylated arylhydrazine **S6** (1.46 g, 6.43 mmol, 1.0 equiv) followed by dry *n*-pentane (30 mL). To the resulting homogeneous, vigorously stirred solution was added portion–wise the solid DBAD (1.44 g, 6.10 mmol, 0.95 equiv) at room temperature. An almost instantaneous color change from yellowish to dark blue was noted with the concomitant precipitation of the hydrazine $(BocNH)_2$. The resulting suspension was further stirred for 1 h at room temperature, and the conversion was monitored by ^{1}H NMR analysis. The crude reaction mixture was then filtered in a glovebox over a fritted funnel, and the blue filtrate was concentrated *in vacuo* to afford the crude silylated aryldiazene, which was then distilled under reduced pressure *via* a short path distillation (**b.p.**: 53–55 °C at 2.1 × 10^{-1} mbar) affording the title compound **1m** (0.890 g, 3.97 mmol, 62%) as a deep blue liquid.

^{1}H NMR (500 MHz, $CDCl_3$): δ/ppm = 0.40 (s, 9H), 2.17 (s, 6H), 6.77 (d, 2H, J = 9.4 Hz). **^{13}C{1H} NMR** (126 MHz, $CDCl_3$): δ/ppm = –2.45 (3C), 18.1 (2C), 115.5 (d, 2C, J = 22.1 Hz), 131.1 (d, 2C, J = 8.9 Hz), 154.3 (d, J = 2.8 Hz), 161.5 (d, J = 246.5 Hz). **^{19}F NMR** (471 MHz, $CDCl_3$): δ/ppm = –115.6 (t, J = 9.5 Hz). **^{1}H/^{29}Si HMQC NMR** (500/99 MHz, $CDCl_3$, optimized for J = 7 Hz): δ/ppm = 0.40/12.6. **HRMS** (APCI) calculated for $C_{11}H_{18}FN_2Si^+$ [$(M+H)^+$]: 225.1218; found: 225.1215. **IR** (ATR): $\tilde{\nu}$/cm^{-1} = 2962, 2928, 1589, 1502, 1377, 1297, 1248, 1124, 1020, 841.

4 General Procedures

4.1 General Procedure for the Coupling of Diazenes with Hexafluorobenzene (GP1)

In an argon-filled glovebox, an oven-dried 10-mL vial equipped with a magnetic stirring bar was charged with CsF (9.1 mg, 60 µmol, 20 mol%) and hexafluorobenzene (**2a**, 104 µL, 167 mg, 0.900 mmol, 3.0 equiv). The reaction vessel was sealed with a septum, transferred out of the glovebox, and connected to a nitrogen manifold using a syringe needle. Anhydrous DMF (1 mL) was added, and to the vigorously stirred mixture was added in one portion a solution of the corresponding silylated aryldiazene (0.300 mmol, 1.0 equiv, purity > 99%) in anhydrous DMF (1 mL) at room temperature. A gentle nitrogen evolution starts instantaneously and a color change from dark blue or dark purple to light red was noted. The conversion was monitored by GLC or ^{1}H NMR analysis, and unless otherwise stated, the silylated aryldiazenes were fully converted within 5 min. The crude mixture was diluted with ethyl acetate (20 mL) and water (10 mL). The layers were separated, and the organic phase was washed with brine (3 x 10 mL). The organic layer was dried over anhydrous $MgSO_4$, filtered, and concentrated under reduced pressure. The resulting crude residue was washed thrice with *n*-pentane. Purification by flash column chromatography on silica gel using *n*-pentane:dichloromethane mixtures afforded the polyfluorinated biaryls as white solids or colorless oils.

Note. In the event that the 18-crown-6 was used as an additive (1.0:1.2 molar ratio), the suspension of the catalyst and crown ether in DMF was stirred for 10 min at room temperature prior to adding the silylated aryldiazene.

4.2 General Procedure for the Coupling of Diazenes with Polyfluoroarenes (GP2)

In an argon-filled glovebox, an oven-dried 10-mL vial equipped with a magnetic stirring bar was charged with CsF (9.1 mg, 60 µmol, 20 mol%) and the corresponding fluoroarene (0.900 mmol, 3.0 equiv). The reaction vessel was sealed with a septum, transferred out of the glovebox, and connected to a nitrogen manifold using a syringe needle. Anhydrous DMF (1 mL) was added, and to the vigorously stirred mixture was added in one portion a solution of the corresponding silylated aryldiazene (0.300 mmol, 1.0 equiv, purity > 99%) in anhydrous DMF (1 mL) at room temperature. A gentle nitrogen evolution starts instantaneously and a color change from dark blue or dark purple to light red was noted. The conversion was monitored by GLC or ^{1}H NMR analysis, and unless otherwise stated, the silylated aryldiazenes were fully converted within 1 h. The crude mixture was diluted with ethyl acetate (20 mL) and water (10 mL). The layers were separated, and the organic phase was washed with brine (3 x 10 mL). The organic layer was dried over anhydrous $MgSO_4$, filtered, and concentrated under reduced pressure. The resulting crude residue was washed thrice with *n*-pentane. Purification

by flash column chromatography on silica gel using *n*-pentane:dichloromethane mixtures afforded the *major* regioisomers as white solids or colorless liquids. In the event that the desired regioisomer was contaminated (with either the *ortho*-, *meta*- or α-substituted regioisomers), the obtained mixture was either recrystallized or triturated with a minimum amount of cold *n*-pentane, affording in both cases the regioisomerically pure polyfluorinated biaryls.

Regioisomeric ratios (*para*:*ortho*:*meta* and β:α) and yields of **3hh**, **3ai** and **3aj** were determined according to **GP2** on a 0.10 mmol scale by quantitative ^{19}F NMR analysis of the crude reaction mixtures in $CDCl_3$ (0.7 mL) with hexafluorobenzene as an internal standard. For a more precise integration of ^{19}F NMR spectra the relaxation delay was determined and set to either 50 or 100 sec.

Note. Owing to the dinstinct reactivities of the various polyfluoroarenes and diazenes employed, competitive degradation of the diazene or bisarylation were likely to occur at different degrees. To overcome this potential loss of yield, a larger excess of fluoroarene was employed in some cases. It is also important to note that the unreacted starting polyfluoroarenes **2e–g** were fully recovered by column chromatography on silica gel.

5 Characterization Data for the Polyfluorinated Biaryls

2,3,4,5,6-Pentafluoro-4'-methyl-1,1'-biphenyl (3aa)

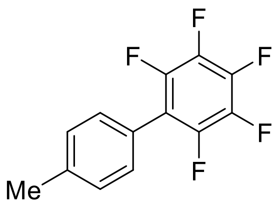

3aa
$C_{13}H_7F_5$
M = 258.19 g/mol

Prepared according to **GP1** from the (*E*)-1-(*p*-tolyl)-2-(trimethylsilyl)diazene (**1a**, 57.7 mg, 0.300 mmol, 1.0 equiv). Purification by flash column chromatography on silica gel using *n*-pentane as eluent afforded the title compound **3aa** (60.9 mg, 0.236 mmol, 79%) as a white solid.

R_f = 0.58 (*n*-pentane). **M.p.**: 116–118 °C (*n*-pentane). **^{1}H NMR** (500 MHz, $CDCl_3$): δ/ppm = 2.44 (s, 3H), 7.32 (app s, 4H). **^{13}C{1H} NMR** (126 MHz, $CDCl_3$): δ/ppm = 21.5, 116.1 (app td, *J* = 16.9, 3.4 Hz), 123.5, 129.6 (2C), 130.1 (2C), 138.0 (dm, *J* = 250.8 Hz, 2C), 139.6, 140.4 (dm, *J* = 252.4 Hz), 144.4 (dm, *J* = 250.8 Hz, 2C). **^{19}F NMR** (471 MHz, $CDCl_3$): δ/ppm = –162.5 (app td, *J* = 22.7, 8.1 Hz, 2F), –156.2 (t, *J* = 21.9 Hz), –143.4 (dd, *J* = 22.9, 8.1 Hz, 2F). **HRMS** (APCI) calculated for $C_{13}H_6F_5^+$ [$(M–H)^+$]: 257.0384; found: 257.0387. **IR** (ATR): $\tilde{\nu}$/cm^{-1} = 3023, 2924, 2865, 1653, 1613, 1482, 1405, 1383, 1312, 1215, 1192, 1042, 975, 854, 823, 770.
The spectroscopic and analytical data are in accordance with those reported.[S8]

2,3,4,5,6-Pentafluoro-1,1'-biphenyl (3ba)

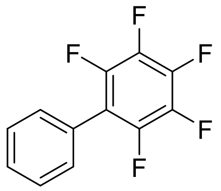

3ba
$C_{12}H_5F_5$
M = 244.16 g/mol

Prepared according to **GP1** from the (*E*)-1-phenyl-2-(trimethylsilyl)diazene (**1b**, 53.5 mg, 0.300 mmol, 1.0 equiv). Purification by flash column chromatography on silica gel using *n*-pentane as eluent afforded the title compound **3ba** (55.6 mg, 0.228 mmol, 76%) as a white solid.

R$_f$ = 0.55 (*n*-pentane). **M.p.**: 109–111 °C (*n*-pentane). **^{1}H NMR** (500 MHz, $CDCl_3$): δ/ppm = 7.40–7.45 (m, 2H), 7.45–7.53 (m, 3H). **^{13}C{1H} NMR** (101 MHz, $CDCl_3$): δ/ppm = 116.1 (app td, *J* = 17.4, 4.4 Hz), 126.6, 128.9 (2C), 129.4, 130.3 (2C), 138.0 (dm, *J* = 254.3 Hz, 2C), 140.6 (dm, *J* = 254.3 Hz), 144.3 (dm, *J* = 249.7 Hz, 2C). **^{19}F NMR** (471 MHz, $CDCl_3$): δ/ppm = –162.3 (app td, *J* = 22.7, 8.6 Hz, 2F), –155.7 (t, *J* = 20.7 Hz), –143.3 (dd, *J* = 22.9, 8.8 Hz, 2F). **HRMS** (APCI) calculated for $C_{12}H_5F_5^+$ [M$^+$]: 244.0306; found: 244.0307. **IR** (ATR): $\tilde{\nu}$/cm^{-1} = 2920, 1652, 1521, 1483, 1438, 1397, 1319, 1198, 1061, 977, 832.
The spectroscopic and analytical data are in accordance with those reported.[S8]

2,3,4,5,6-Pentafluoro-4'-methoxy-1,1'-biphenyl (3ca)

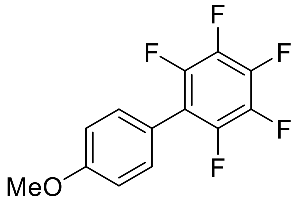

3ca
$C_{13}H_7F_5O$
M = 274.19 g/mol

Prepared according to **GP1** from the (*E*)-1-(4-methoxyphenyl)-2-(trimethylsilyl)diazene (**1c**, 62.5 mg, 0.300 mmol, 1.0 equiv). Purification by flash column chromatography on silica gel using *n*-pentane as eluent afforded the title compound **3ca** (70.6 mg, 0.258 mmol, 86%) as a white solid.

R$_f$ = 0.29 (*n*-pentane). **M.p.**: 114–116 °C (*n*-pentane). **^{1}H NMR** (500 MHz, $CDCl_3$): δ/ppm = 3.87 (s, 3H), 7.02 (d, *J* = 8.7 Hz, 2H), 7.36 (d, *J* = 8.7 Hz, 2H). **^{13}C{1H} NMR** (101 MHz, $CDCl_3$): δ/ppm = 55.5, 114.4 (2C), 115.9 (app td, *J* = 17.3, 4.1 Hz), 118.5, 131.6 (2C), 138.0 (dm, *J* = 244.6 Hz, 2C), 140.2 (dm, *J* = 241.7 Hz), 144.3 (dm, *J* = 246.1 Hz, 2C), 160.4. **^{19}F NMR** (471 MHz, $CDCl_3$): δ/ppm = –162.6 (app td, *J* = 22.9, 8.2 Hz, 2F), –156.5 (t, *J* = 21.2 Hz), –143.6 (dd, *J* = 22.6, 8.3 Hz, 2F). **HRMS** (APCI) calculated for $C_{13}H_7F_5O^+$ [M$^+$]: 274.0412; found: 274.0412. **IR** (ATR): $\tilde{\nu}$/cm^{-1} = 3023, 2941, 2846, 1654, 1604, 1575, 1481, 1411, 1293, 1248, 1177, 1026, 975, 826, 776.
The spectroscopic and analytical data are in accordance with those reported.[S8]

2',3',4',5',6'-Pentafluoro-[1,1'-biphenyl]-4-carbonitrile (3da)

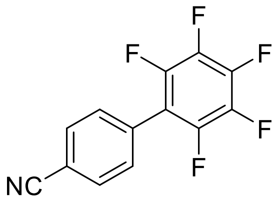

3da
$C_{13}H_4F_5N$
M = 269.17 g/mol

Prepared according to **GP1** from the (*E*)-4-((trimethylsilyl)diazenyl)benzonitrile (**1d**, 61.0 mg, 0.300 mmol, 1.0 equiv). Purification by flash column chromatography on silica gel using *n*-pentane:dichloromethane (4:1) as eluent afforded the title compound **3da** (57.2 mg, 0.213 mmol, 71%) as a white solid.

R$_f$ = 0.31 (*n*-pentane:dichloromethane 4:1). **M.p.**: 128–130 °C (*n*-pentane). **^{1}H NMR** (400 MHz, $CDCl_3$): δ/ppm = 7.56 (d, *J* = 8.4 Hz, 2H), 7.80 (d, *J* = 8.4 Hz, 2H). **^{13}C{1H} NMR** (101 MHz, $CDCl_3$): δ/ppm = 113.5, 114.2 (app td, *J* = 17.3, 4.0 Hz), 118.2, 131.1 (2C), 131.3, 132.6 (2C), 138.1 (dm, *J* = 258.8 Hz, 2C), 141.3 (dm, *J* = 245.9 Hz), 144.2 (dm, *J* = 255.6 Hz, 2C). **^{19}F NMR** (471 MHz, $CDCl_3$): δ/ppm = –161.1 (app td, *J* = 21.4, 7.9 Hz, 2F), –152.9 (t, *J* = 21.3 Hz), –142.8 (dd, *J* = 22.9, 8.0 Hz, 2F). **HRMS** (APCI) calculated for $C_{13}H_5F_5N^+$ [(M+H)$^+$]: 270.0337; found: 270.0335. **IR** (ATR): $\tilde{\nu}$/cm^{-1} = 3059, 2236, 1650, 1525, 1482, 1407, 1320, 1185, 1070, 984, 844, 778.
The spectroscopic and analytical data are in accordance with those reported.[S8]

Methyl 2',3',4',5',6'-pentafluoro-[1,1'-biphenyl]-4-carboxylate (3ea)

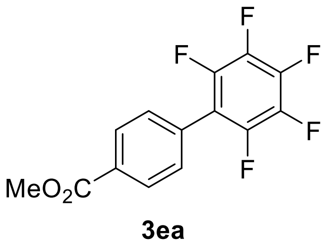

3ea
$C_{14}H_7F_5O_2$
M = 302.20 g/mol

Prepared according to **GP1** from the methyl (*E*)-4-((trimethylsilyl)diazenyl)benzoate (**1e**, 70.9 mg, 0.300 mmol, 1.0 equiv). 18-Crown-6 (19 mg, 72 µmol, 24 mol%) was used as an

additive for this reaction. Purification by flash column chromatography on silica gel using *n*-pentane:dichloromethane (100:0 → 9:1 → 4:1) as eluent afforded the title compound **3ea** (67.4 mg, 0.223 mmol, 74%) as a white solid.

R$_f$ = 0.27 (*n*-pentane:dichloromethane 4:1). **M.p.**: 110–112 °C (*n*-pentane). **^{1}H NMR** (500 MHz, $CDCl_3$): δ/ppm = 3.96 (s, 3H), 7.51 (d, *J* = 8.4 Hz, 2H), 8.16 (d, *J* = 8.4 Hz, 2H). **^{13}C{1H} NMR** (126 MHz, $CDCl_3$): δ/ppm = 52.5, 115.1 (app td, *J* = 17.3, 4.9 Hz), 130.0 (2C), 130.4 (2C), 131.1, 138.1 (dm, *J* = 253.6 Hz, 2C), 140.9 (dm, *J* = 252.4 Hz), 144.3 (dm, *J* = 245.6 Hz, 2C), 145.2, 166.5. **^{19}F NMR** (471 MHz, $CDCl_3$): δ/ppm = –161.6 (app td, *J* = 21.5, 8.0 Hz, 2F), –154.1 (t, *J* = 21.4 Hz), –142.8 (dd, *J* = 22.8, 8.1 Hz, 2F). **HRMS** (APCI) calculated for $C_{14}H_8F_5O_2^+$ [(M+H)$^+$]: 303.0439; found: 303.0442. **IR** (ATR): $\tilde{\nu}$/cm^{-1} = 2961, 2922, 2852, 1726, 1651, 1484, 1435, 1404, 1276, 1195, 1103, 1062, 1045, 980, 856, 812, 754.
The spectroscopic and analytical data are in accordance with those reported.[S8]

2,3,4,5,6-Pentafluoro-3'-nitro-1,1'-biphenyl (3fa)

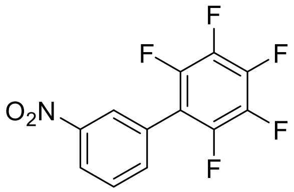

3fa
$C_{12}H_4F_5NO_2$
M = 289.16 g/mol

Prepared according to **GP1** from the (*E*)-1-(3-nitrophenyl)-2-(trimethylsilyl)diazene (**1f**, 67.0 mg, 0.300 mmol, 1.0 equiv). 18-Crown-6 (19 mg, 72 µmol, 24 mol%) was used as an additive for this reaction. Purification by flash column chromatography on silica gel using *n*-pentane:dichloromethane (100:0 → 9:1) as eluent afforded the title compound **3fa** (60.4 mg, 0.209 mmol, 70%) as a white solid.

R$_f$ = 0.58 (*n*-pentane:dichloromethane 4:1). **M.p.**: 111–113 °C (*n*-pentane). **^{1}H NMR** (500 MHz, $CDCl_3$): δ/ppm = 7.69–7.74 (m, 1H), 7.75–7.80 (m, 1H), 8.31–8.37 (m, 2H). **^{13}C{1H} NMR** (126 MHz, $CDCl_3$): δ/ppm = 113.7 (app td, *J* = 17.1, 4.4 Hz), 124.4, 125.4, 128.2, 130.0, 136.2, 138.1 (dm, *J* = 256.1 Hz, 2C), 141.4 (dm, *J* = 253.4 Hz), 144.3 (dm, *J* = 248.0 Hz, 2C), 148.6. **^{19}F NMR** (471 MHz, $CDCl_3$): δ/ppm = –160.8 (app td, *J* = 21.3, 7.6 Hz, 2F), –152.8 (t, *J* = 21.4 Hz), –142.9 (dd, *J* = 22.1, 7.8 Hz, 2F). **HRMS** (LIFDI) calculated for $C_{12}H_5F_5NO_2^+$ [(M+H)$^+$]:

290.0235; found: 290.0225. **IR** (ATR): ṽ/cm^{-1} = 3097, 2922, 2853, 1649, 1619, 1487, 1431, 1347, 1285, 1259, 1210, 1069, 984, 902, 834, 812, 722.

The spectroscopic and analytical data are in accordance with those reported.[S9]

2,3,4,5,6-Pentafluoro-4'-iodo-1,1'-biphenyl (3ga)

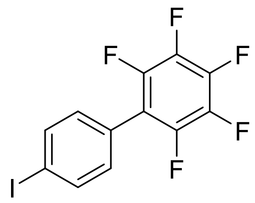

3ag
$C_{12}H_4F_5I$
M = 370.06 g/mol

Prepared according to **GP1** from the (*E*)-1-(4-iodophenyl)-2-(trimethylsilyl)diazene (**1g**, 91.3 mg, 0.300 mmol, 1.0 equiv) and hexafluorobenzene (347 µL, 558 mg, 3.00 mmol, 10 equiv). Purification by flash column chromatography on silica gel using *n*-pentane as eluent afforded the title compound **3ga** (75.8 mg, 0.212 mmol, 71%) as a white solid.

Note: A minor reoptimization was required for this transformation as side products originating from competing halogen–metal exchange were observed. After reoptimization, 1,4-diiodobenzene (10%) formed along with 1,4-bis(pentafluorophenyl)benzene (6%); yields were estimated by quantitative ^{1}H NMR with dibromomethane as an internal standard.

R$_f$ = 0.66 (*n*-pentane). **M.p.**: 116–118 °C (*n*-pentane). **^{1}H NMR** (500 MHz, $CDCl_3$): δ/ppm = 7.16 (d, *J* = 8.2 Hz, 2H), 7.84 (d, *J* = 8.2 Hz, 2H). **^{13}C{1H} NMR** (126 MHz, $CDCl_3$): δ/ppm = 95.8, 115.1 (app td, *J* = 17.1, 3.5 Hz), 126.0, 131.9 (2C), 138.1 (dm, *J* = 252.2 Hz, 2C), 138.2 (2C), 140.8 (dm, *J* = 254.1 Hz), 144.2 (dm, *J* = 249.9 Hz, 2C). **^{19}F NMR** (471 MHz, $CDCl_3$): δ/ppm = –161.7 (app td, *J* = 23.2, 8.5 Hz, 2F), –154.7 (t, *J* = 20.8 Hz), –143.0 (dd, *J* = 22.8, 8.5 Hz, 2F). **HRMS** (APCI) calculated for $C_{12}H_4F_5I^+$ [M$^+$]: 369.9272; found: 369.9272. **IR** (ATR): ṽ/cm^{-1} = 2922, 1652, 1521, 1477, 1385, 1196, 1055, 976, 942, 849, 823, 768.

The spectroscopic and analytical data are in accordance with those reported.[S10]

4'-Bromo-2,3,4,5,6-pentafluoro-1,1'-biphenyl (3ha)

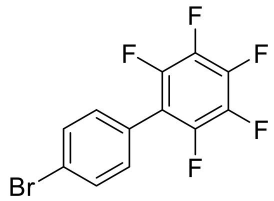

3ha
$C_{12}H_4BrF_5$
M = 323.06 g/mol

Prepared according to **GP1** from the (*E*)-1-(4-bromophenyl)-2-(trimethylsilyl)diazene (**1h**, 77.2 mg, 0.300 mmol, 1.0 equiv). Purification by flash column chromatography on silica gel using *n*-pentane as eluent afforded the title compound **3ha** (75.8 mg, 0.235 mmol, 78%) as a white solid.

Note. Side products originating from the competing halogen–metal exchange were observed, and 1,4-dibromobenzene (2%) formed along with 1,4-bis(pentafluorophenyl)benzene (3%); yields were estimated by quantitative ^{1}H NMR with dibromomethane as an internal standard.

R$_f$ = 0.58 (*n*-pentane). **M.p.**: 88–90 °C (*n*-pentane). **^{1}H NMR** (500 MHz, $CDCl_3$): δ/ppm = 7.30 (d, *J* = 8.3 Hz, 2H), 7.64 (d, *J* = 8.3 Hz, 2H). **^{13}C{1H} NMR** (126 MHz, $CDCl_3$): δ/ppm = 115.0 (app td, *J* = 17.2, 4.0 Hz), 124.0, 125.4, 131.8 (2C), 132.2 (2C), 138.1 (dm, *J* = 248.5 Hz, 2C), 140.8 (dm, *J* = 254.1 Hz), 144.2 (dm, *J* = 251.9 Hz). **^{19}F NMR** (471 MHz, $CDCl_3$): δ/ppm = –161.8 (app td, *J* = 21.7, 8.0 Hz, 2F), –154.7 (t, *J* = 21.2 Hz), –143.1 (dd, *J* = 22.8, 7.9 Hz, 2F). **HRMS** (APCI) calculated for $C_{12}H_4BrF_5^+$ [M$^+$]: 321.9411; found: 321.9408. **IR** (ATR): $\tilde{\nu}$/cm^{-1} = 2921, 1651, 1587, 1520, 1477, 1391, 1320, 1199, 1060, 1009, 977, 829, 772.
The spectroscopic and analytical data are in accordance with those reported.[S10]

4'-Chloro-2,3,4,5,6-pentafluoro-1,1'-biphenyl (3ia)

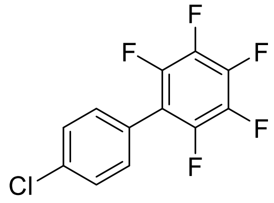

3ia
$C_{12}H_4ClF_5$
M = 278.61 g/mol

Prepared according to **GP1** from the (*E*)-1-(4-chlorophenyl)-2-(trimethylsilyl)diazene (**1i**, 63.8 mg, 0.300 mmol, 1.0 equiv). Purification by flash column chromatography on silica gel using *n*-pentane as eluent afforded the title compound **3ia** (67.6 mg, 0.243 mmol, 81%) as a white solid.

R$_f$ = 0.60 (*n*-pentane). **M.p.**: 82–84 °C (*n*-pentane). **^{1}H NMR** (400 MHz, $CDCl_3$): δ/ppm = 7.37 (d, *J* = 8.5 Hz, 2H), 7.48 (d, *J* = 8.5 Hz, 2H). **^{13}C{1H} NMR** (101 MHz, $CDCl_3$): δ/ppm = 114.9 (app td, *J* = 16.9, 3.5 Hz), 124.9, 129.2 (2C), 131.6 (2C), 135.7, 138.1 (dm, *J* = 253.9 Hz, 2C), 140.7 (dm, *J* = 256.2 Hz), 144.2 (dm, *J* = 248.8 Hz, 2C). **^{19}F NMR** (471 MHz, $CDCl_3$): δ/ppm = –161.9 (app td, *J* = 23.6, 9.0 Hz, 2F), –154.8 (t, *J* = 21.1 Hz), –143.1 (dd, *J* = 22.6, 8.0 Hz, 2F). **HRMS** (APCI) calculated for $C_{12}H_4ClF_5^+$ [M^+]: 277.9916; found: 277.9916. **IR** (ATR): $\tilde{\nu}$/cm^{-1} = 2956, 2923, 1655, 1596, 1525, 1482, 1395, 1199, 1090, 1061, 1015, 978, 832, 772.
The spectroscopic and analytical data are in accordance with those reported.[S10]

2,3,4,4',5,6-Hexafluoro-1,1'-biphenyl (3ja)

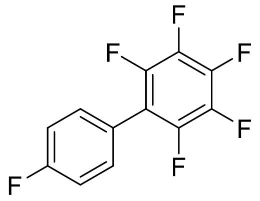

3ja
$C_{12}H_4F_6$
M = 262.15 g/mol

Prepared according to **GP1** from the (*E*)-1-(4-fluorophenyl)-2-(triethylsilyl)diazene (**1j**, 71.5 mg, 0.300 mmol, 1.0 equiv). The reaction mixture was stirred at room temperature for 3 h (gentle evolution of N_2). Purification by flash column chromatography on silica gel using *n*-pentane as eluent afforded the title compound **3ja** (59.7 mg, 0.228 mmol, 76%) as colorless crystalline solid.

R$_f$ = 0.61 (*n*-pentane). **M.p.**: 114–116 °C (*n*-pentane). **^{1}H NMR** (500 MHz, $CDCl_3$): δ/ppm = 7.19 (app t, *J* = 8.6 Hz, 2H), 7.38–7.44 (m, 2H). **^{13}C{1H} NMR** (126 MHz, $CDCl_3$): δ/ppm = 115.1 (app td, *J* = 16.7, 3.7 Hz), 116.1 (d, *J* = 21.6 Hz, 2C), 122.4, 132.2 (d, *J* = 8.3 Hz, 2C), 138.0 (dm, *J* = 246.3 Hz), 140.7 (dm, *J* = 246.3 Hz), 144.3 (dm, *J* = 247.6 Hz, 2C), 163.3 (d, *J* = 249.7 Hz, 2C). **^{19}F NMR** (471 MHz, $CDCl_3$): δ/ppm = –162.0 (app td, *J* = 22.5, 7.9 Hz, 2F), –155.2 (t, *J* = 21.0 Hz), –143.3 (dd, *J* = 22.8, 8.5 Hz, 2F), –111.3 (m$_c$). **HRMS** (APCI) calculated

for $C_{12}H_4F_6^+$ [M+]: 262.0212; found: 262.0208. **IR** (ATR): $\tilde{\nu}$/cm^{-1} = 2923, 2854, 1600, 1486, 1401, 1224, 1160, 1095, 1063, 979, 841, 776.

The spectroscopic and analytical data are in accordance with those reported.[S8]

2,3,4,5,6-Pentafluoro-4'-(trifluoromethyl)-1,1'-biphenyl (3ka)

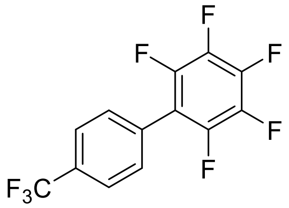

3ka
$C_{13}H_4F_8$
M = 312.16 g/mol

Prepared according to **GP1** from the (*E*)-1-(4-(trifluoromethyl)phenyl)-2-(trimethylsilyl)diazene (**1k**, 73.9 mg, 0.300 mmol, 1.0 equiv) and hexafluorobenzene (347 µL, 558 mg, 3.00 mmol, 10 equiv). The reaction mixture was stirred at room temperature for 1 h (vigorous and then gentle evolution of N_2). Purification by flash column chromatography on silica gel using *n*-pentane as eluent afforded the title compound **3ka** (74.5 mg, 0.239 mmol, 80%) as white solid.

R_f = 0.62 (*n*-pentane). **M.p.**: 57–59 °C (*n*-pentane). **^{1}H NMR** (500 MHz, $CDCl_3$): δ/ppm = 7.57 (d, *J* = 8.4 Hz, 2H), 7.77 (d, *J* = 8.4 Hz, 2H). **^{13}C{1H} NMR** (126 MHz, $CDCl_3$): δ/ppm = 114.7 (app td, *J* = 16.9, 4.1 Hz), 123.9 (q, *J* = 272.1 Hz), 125.9 (q, *J* = 3.9 Hz, 2C), 130.3, 130.8 (2C), 131.6 (q, *J* = 33.6 Hz), 138.1 (dm, *J* = 251.6 Hz, 2C), 141.1 (dm, *J* = 257.2 Hz), 144.3 (dm, *J* = 246.0 Hz, 2C). **^{19}F NMR** (471 MHz, $CDCl_3$): δ/ppm = –161.5 (app td, *J* = 22.7, 8.5 Hz, 2F), –153.8 (t, *J* = 21.2 Hz), –143.0 (dd, *J* = 22.5, 8.1 Hz, 2F), –63.0 (s, 3F). **HRMS** (APCI) calculated for $C_{13}H_4F_8^+$ [M+]: 312.0180; found: 312.0180. **IR** (ATR): $\tilde{\nu}$/cm^{-1} = 2923, 2852, 1653, 1619, 1578, 1483, 1429, 1406, 1321, 1162, 1124, 1058, 1017, 979, 843, 781.

The spectroscopic and analytical data are in accordance with those reported.[S8]

2,2',3,4,5,6-Hexafluoro-1,1'-biphenyl (3la)

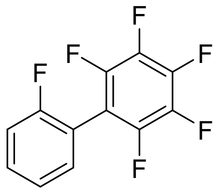

3la
$C_{12}H_4F_6$
M = 262.15 g/mol

Prepared according to **GP1** from the (*E*)-1-(2-fluorophenyl)-2-(trimethylsilyl)diazene (**1l**, 58.9 mg, 0.300 mmol, 1.0 equiv). The reaction mixture was stirred at room temperature for 3 h. Purification by flash column chromatography on silica gel using *n*-pentane as eluent afforded the title compound **3la** (55.4 mg, 0.211 mmol, 70%) as a colorless crystalline solid.

R_f = 0.67 (*n*-pentane). **M.p.**: 58–60 °C (*n*-pentane). **^{1}H NMR** (500 MHz, $CDCl_3$): δ/ppm = 7.23 (app t, *J* = 9.3 Hz, 1H), 7.27 (app t, *J* = 7.5 Hz, 1H), 7.35 (app t, *J* = 7.4 Hz, 1H), 7.45–7.52 (m, 1H). **^{13}C{1H} NMR** (126 MHz, $CDCl_3$): δ/ppm = 110.3 (app td, *J* = 18.3, 4.1 Hz), 114.3 (d, *J* = 16.6 Hz), 116.3 (d, *J* = 21.9 Hz), 124.5 (d, *J* = 3.5 Hz), 131.9 (d, *J* = 8.3 Hz), 132.1, 137.9 (dm, *J* = 251.3 Hz, 2C), 141.3 (dm, *J* = 254.4 Hz), 144.6 (dm, *J* = 259.9 Hz, 2C), 160.1 (d, *J* = 250.2 Hz). **^{19}F NMR** (471 MHz, $CDCl_3$): δ/ppm = –162.1 (app td, *J* = 22.2, 7.7 Hz, 2F), –154.1 (t, *J* = 20.6 Hz), –140.3 (app dt, *J* = 24.2, 8.4 Hz, 2F), –112.8 (m_c). **HRMS** (APCI) calculated for $C_{12}H_4F_6^+$ [M^+]: 262.0212; found: 262.0210. **IR** (ATR): $\tilde{\nu}$/cm^{-1} = 2922, 1654, 1613, 1523, 1483, 1443, 1388, 1323, 1270, 1231, 1192, 1110, 1061, 980, 868, 801, 754.
The spectroscopic and analytical data are in accordance with those reported.[S8]

2,3,4,4',5,6-Hexafluoro-2',6'-dimethyl-1,1'-biphenyl (3ma)

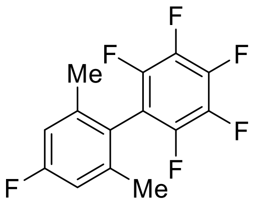

3ma
$C_{14}H_8F_6$
M = 290.21 g/mol

Prepared according to **GP1** from the (*E*)-1-(4-fluoro-2,6-dimethylphenyl)-2-(trimethylsilyl)diazene (**1m**, 67.3 mg, 0.300 mmol, 1.0 equiv). Purification by flash column chromatography on silica gel using *n*-pentane as eluent afforded the title compound **3ma** (65.2 mg, 0.225 mmol, 75%) as a colorless oil.

R$_f$ = 0.66 (*n*-pentane). **^{1}H NMR** (500 MHz, $CDCl_3$): δ/ppm = 2.08 (s, 6H), 6.89 (d, *J* = 9.4 Hz, 2H). **^{13}C{1H} NMR** (126 MHz, $CDCl_3$): δ/ppm = 20.4 (2C), 113.7 (app td, *J* = 20.6, 3.8 Hz), 114.8 (d, *J* = 21.5 Hz, 2C), 121.5, 138.0 (dm, *J* = 252.6 Hz, 2C), 140.1 (d, *J* = 8.8 Hz, 2C), 140.9 (dm, *J* = 246.2 Hz), 144.0 (dm, *J* = 249.1 Hz, 2C), 163.2 (d, *J* = 248.6 Hz). **^{19}F NMR** (471 MHz, $CDCl_3$): δ/ppm = –161.6 (app td, *J* = 22.6, 8.2 Hz, 2F), –154.5 (t, *J* = 21.6 Hz), –140.0 (dd, *J* = 23.3, 8.4 Hz, 2F), –111.4 (t, *J* = 9.5 Hz). **HRMS** (APCI) calculated for $C_{14}H_8F_6^+$ [M$^+$]: 290.0525; found: 290.0520. **IR** (ATR): $\tilde{\nu}$/cm^{-1} = 2928, 1608, 1520, 1486, 1303, 1187, 1130, 1057, 1025, 984, 859, 832, 705.

2,3,4,5,6-Pentafluoro-2',6'-dimethyl-1,1'-biphenyl (3na)

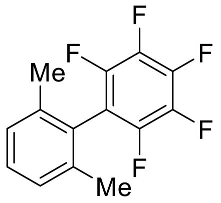

3na
$C_{14}H_9F_5$
M = 272.22 g/mol

Prepared according to **GP1** from the (*E*)-1-(2,6-dimethylphenyl)-2-(triethylsilyl)diazene (**1n**, 74.5 mg, 0.300 mmol, 1.0 equiv). 18-Crown-6 (19 mg, 72 µmol, 24 mol%) was used as an additive for this reaction. Purification by flash column chromatography on silica gel using *n*-pentane as eluent afforded the title compound **3na** (56.4 mg, 0.207 mmol, 69%) as a white solid.

R$_f$ = 0.64 (*n*-pentane). **M.p.**: 51–53 °C (*n*-pentane). **^{1}H NMR** (400 MHz, $CDCl_3$): δ/ppm = 2.10 (s, 6H), 7.18 (d, *J* = 7.6 Hz, 2H), 7.29 (t, *J* = 7.5 Hz, 1H). **^{13}C{1H} NMR** (101 MHz, $CDCl_3$): δ/ppm = 20.2 (2C), 114.5 (app td, *J* = 21.3, 3.6 Hz), 125.8, 127.8 (2C), 129.6 (2C), 137.5, 137.9 (dm, *J* = 251.5 Hz, 2C), 140.8 (dm, *J* = 252.0 Hz), 143.9 (dm, *J* = 246.3 Hz, 2C). **^{19}F NMR** (471 MHz, $CDCl_3$): δ/ppm = –162.0 (app td, *J* = 22.2, 8.3 Hz, 2F), –155.2 (t, *J* = 21.0 Hz), –140.2 (dd, *J* = 22.4, 8.5 Hz, 2F). **HRMS** (LIFDI) calculated for $C_{14}H_9F_5^+$ [M$^+$]: 272.0619; found:

272.0611. **IR** (ATR): $\tilde{\nu}$/cm^{-1} = 3029, 2959, 2926, 2860, 1651, 1584, 1517, 1486, 1382, 1308, 1186, 1096, 1054, 979, 854, 773, 726.

2-(Perfluorophenyl)naphthalene (3oa)

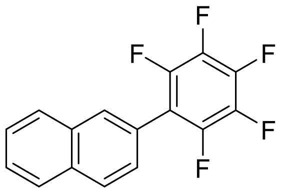

3oa
$C_{16}H_7F_5$
M = 294.22 g/mol

Prepared according to **GP1** from the (*E*)-1-(naphthalen-2-yl)-2-(trimethylsilyl)diazene (**1o**, 68.5 mg, 0.300 mmol, 1.0 equiv). The reaction mixture was stirred at room temperature for 1 h. Purification by flash column chromatography on silica gel using *n*-pentane as eluent afforded the title compound **3oa** (66.5 mg, 0.226 mmol, 75%) as a white solid.

R$_f$ = 0.57 (*n*-pentane). **M.p.**: 169–171 °C (*n*-pentane). **^{1}H NMR** (500 MHz, C_6D_6): δ/ppm = 7.22–7.29 (m, 3H), 7.55–7.62 (m, 3H), 7.65 (s, 1H). **^{13}C{1H} NMR** (101 MHz, CD_2Cl_2): δ/ppm = 116.4 (app td, *J* = 18.1, 4.4 Hz), 124.2, 127.2, 127.4, 127.7, 128.1, 128.6, 128.8, 130.5, 133.5, 133.7, 138.4 (dm, *J* = 251.3 Hz, 2C), 140.9 (dm, *J* = 254.4 Hz), 144.8 (dm, *J* = 243.1 Hz, 2C). **^{19}F NMR** (471 MHz, C_6D_6): δ/ppm = –162.7 (app td, *J* = 23.3, 8.1 Hz, 2F), –156.2 (t, *J* = 21.5 Hz), –143.7 (dd, *J* = 23.5, 8.0 Hz, 2F). **HRMS** (APCI) calculated for $C_{16}H_7F_5^+$ [M$^+$]: 294.0462; found: 294.0463. **IR** (ATR): $\tilde{\nu}$/cm^{-1} = 3067, 2922, 1650, 1482, 1431, 1346, 1274, 1206, 1176, 1052, 1013, 977, 898, 865, 827, 779, 746.

2,2'',3,3'',4,4'',5,5'',6,6''-Decafluoro-1,1':3',1''-terphenyl (3pa)

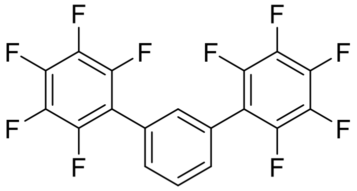

3pa
$C_{18}H_4F_{10}$
M = 410.21 g/mol

Prepared according to **GP1** from the 1,3-bis((*E*)-(trimethylsilyl)diazenyl)benzene (**1p**, 83.6 mg, 0.300 mmol, 1.0 equiv) and hexafluorobenzene (693 µL, 1.12 g, 6.00 mmol, 20 equiv). The reaction mixture was stirred at room temperature for 1 h. Purification by flash column chromatography on silica gel using *n*-pentane as eluent afforded the title compound **3pa** (88.3 mg, 0.215 mmol, 72%) as a white solid.

R$_f$ = 0.43 (*n*-pentane). **M.p.**: 95–97 °C (*n*-pentane). **^{1}H NMR** (400 MHz, $CDCl_3$): δ/ppm = 7.50–7.57 (3H, m), 7.62–7.67 (1H, m). **^{13}C{1H} NMR** (101 MHz, $CDCl_3$): δ/ppm = 115.1 (app td, *J* = 16.9, 3.9 Hz, 2C), 127.2 (2C), 129.4, 131.2 (2C), 132.0, 138.1 (dm, *J* = 251.4 Hz, 4C), 140.9 (dm, *J* = 259.5 Hz, 2C), 144.3 (dm, *J* = 254.1 Hz, 4C). **^{19}F NMR** (471 MHz, $CDCl_3$): δ/ppm = –161.8 (app td, *J* = 22.0, 8.2 Hz, 4F), –154.6 (t, *J* = 21.0 Hz, 2F), –143.1 (dd, *J* = 22.6, 8.1 Hz, 4F). **HRMS** (EI) calculated for $C_{18}H_4F_{10}^+$ [M$^+$]: 410.0148; found: 410.0141. **IR** (ATR): $\tilde{\nu}$/cm^{-1} = 2921, 2852, 1647, 1584, 1520, 1495, 1472, 1434, 1397, 1329, 1147, 1073, 985, 926, 906, 825, 803, 763.

The spectroscopic and analytical data are in accordance with those reported.[S11]

2,3,5,6-Tetrafluoro-4-(4-(trifluoromethyl)phenyl)pyridine (3kb)

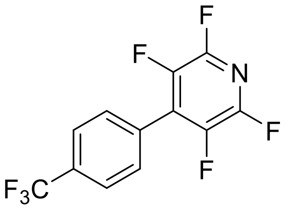

major
2,3,5,6-tetrafluoro-4-(4-(trifluoromethyl)phenyl)pyridine

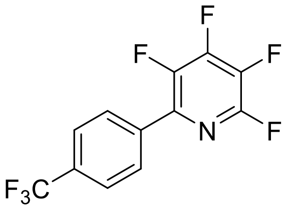

minor
2,3,4,5-tetrafluoro-6-(4-(trifluoromethyl)phenyl)pyridine

3kb
$C_{12}H_4F_7N$
M = 295.16 g/mol
rs = 83:16:1

Prepared according to **GP2** from the (*E*)-1-(4-(trifluoromethyl)phenyl)-2-(trimethylsilyl)diazene (**1k**, 73.9 mg, 0.300 mmol, 1.0 equiv) and pentafluoropyridine (**2a**, 329 µL, 507 mg, 3.00 mmol, 10 equiv). Purification by flash column chromatography on silica gel using *n*-pentane as eluent afforded the *major* isomer *para*-**3kb** (59.7 mg, 0.202 mmol, 67%) as a colorless liquid, and the *minor* isomer *ortho*-**3kb** (12.2 mg, 41.3 µmol, 14%) as a pale yellow liquid.

Analytical data for *para*-**3kb** (*major* isomer):

R$_f$ = 0.32 (*n*-pentane). **^{1}H NMR** (700 MHz, $CDCl_3$): δ/ppm = 7.67 (d, *J* = 8.3 Hz, 2H), 7.83 (d, *J* = 8.3 Hz, 2H). **^{13}C{1H} NMR** (176 MHz, $CDCl_3$): δ/ppm = 123.7 (q, *J* = 273.6 Hz), 126.1 (q, *J* = 3.1 Hz, 2C), 129.6, 130.4 (2C), 132.0 (tt, *J* = 14.6, 2.6 Hz), 132.7 (q, *J* = 32.8 Hz), 139.3 (dm, *J* = 259.5 Hz, 2C), 144.2 (dm, *J* = 250.3 Hz, 2C). **^{19}F NMR** (471 MHz, $CDCl_3$): δ/ppm = –144.7 (app td, *J* = 29.4, 13.7 Hz, 2F), –89.7 (app td, *J* = 29.5, 14.2 Hz, 2F), –63.2 (s, 3F). **HRMS** (APCI) calculated for $C_{12}H_4F_7N^+$ [M$^+$]: 295.0227; found: 295.0232. **IR** (ATR): $\tilde{\nu}$/cm^{-1} = 2919, 2850, 1641, 1618, 1572, 1462, 1404, 1318, 1293, 1149, 1111, 1064, 1018, 968, 876, 841, 768, 713.

The spectroscopic and analytical data are in accordance with those reported.[S12]

Analytical data for *ortho*-**3kb** (*minor* isomer):

R$_f$ = 0.36 (*n*-pentane). **^{1}H NMR** (700 MHz, $CDCl_3$): δ/ppm = 7.76 (d, *J* = 8.4 Hz, 2H), 8.07 (d, *J* = 8.4 Hz, 2H). **^{13}C{1H} NMR** (176 MHz, $CDCl_3$): δ/ppm = 123.9 (q, *J* = 273.1 Hz), 125.9 (q, *J* = 3.5 Hz, 2C), 126.2, 129.0 (d, *J* = 6.9 Hz, 2C), 132.2 (q, *J* = 33.0 Hz) [$C_{quat/Ar}$–F were omitted from this analysis]. **^{19}F NMR** (471 MHz, $CDCl_3$): δ/ppm = –156.6 (m$_c$), –144.2 (m$_c$), –137.4 (app q, *J* = 17.6 Hz), –81.7 (app td, *J* = 25.9, 16.2 Hz), –63.0 (s, 3F). **HRMS** (APCI) calculated for $C_{12}H_5F_7N^+$ [(M+H)$^+$]: 296.0305; found: 296.0306. **IR** (ATR): $\tilde{\nu}$/cm^{-1} = 2925, 2855, 1727, 1613, 1580, 1505, 1461, 1405, 1320, 1244, 1169, 1128, 1096, 1066, 1014, 850, 770.

2,3,5,6-Tetrafluoro-4-(trifluoromethyl)-1,1'-biphenyl (3bc)

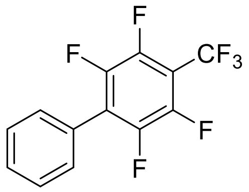

3bc
$C_{13}H_5F_7$
M = 294.17 g/mol
rs = 75:12:13

Prepared according to **GP2** from the (*E*)-1-phenyl-2-(trimethylsilyl)diazene (**1b**, 53.5 mg, 0.300 mmol, 1.0 equiv) and octafluorotoluene (**2c**, 425 µL, 708 mg, 3.00 mmol, 10 equiv). Purification by flash column chromatography on silica gel using *n*-pentane as eluent afforded the title compound **3bc** as a white powder in a mixture of regioisomers. The mixture was triturated with a minimum amount of cold *n*-pentane and filtered off affording the *major* isomer *para*-**3bc** (51.8 mg, 0.176 mmol, 59%) as a white solid.

Analytical data for *para*-**3bc** (*major* isomer):

R$_f$ = 0.57 (*n*-pentane). **M.p.**: 146–148 °C (*n*-pentane). **^{1}H NMR** (500 MHz, $CDCl_3$): δ/ppm = 7.46–7.50 (m, 2H), 7.51–7.56 (m, 3H). **^{13}C{1H} NMR** (126 MHz, $CDCl_3$): δ/ppm = 108.7 (m$_c$), 121.0 (q, *J* = 273.3 Hz), 125.0 (t, *J* = 16.7 Hz), 126.2, 129.0 (2C), 130.09 (2C), 130.14, 144.3 (dm, *J* = 247.9 Hz, 2C), 144.6 (dm, *J* = 262.7 Hz, 2C). **^{19}F NMR** (471 MHz, $CDCl_3$): δ/ppm = –141.6 (m$_c$, 2F), –140.8 (m$_c$, 2F), –56.3 (t, *J* = 21.6, 3F). **HRMS** (APCI) calculated for $C_{13}H_5F_7^+$ [M$^+$]: 294.0274; found: 294.0274. **IR** (ATR): $\tilde{\nu}$/cm^{-1} = 1656, 1602, 1575, 1475, 1438, 1318, 1234, 1189, 1136, 1076, 1028, 977, 925, 826, 794, 736.

4'-Chloro-2,3,5,6-tetrafluoro-4-(trifluoromethyl)-1,1'-biphenyl (3ic)

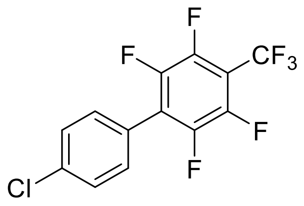

3ic
$C_{13}H_4ClF_7$
M = 328.61 g/mol
rs = 74:16:10

Prepared according to **GP2** from the (*E*)-1-(4-chlorophenyl)-2-(trimethylsilyl)diazene (**1i**, 63.8 mg, 0.300 mmol, 1.0 equiv) and octafluorotoluene (**2c**, 425 µL, 708 mg, 3.00 mmol, 10 equiv). Purification by flash column chromatography on silica gel using *n*-pentane as eluent afforded the *major* isomer *para*-**3ic** (68.6 mg, 0.209 mmol, 70%) as a white solid.

Analytical data for *para*-**3ic** (*major* isomer):

R$_f$ = 0.60 (*n*-pentane). **M.p.**: 95–97 °C (*n*-pentane). **^{1}H NMR** (500 MHz, $CDCl_3$): δ/ppm = 7.42 (d, *J* = 8.8 Hz, 2H), 7.51 (d, *J* = 8.8 Hz, 2H). **^{13}C{1H} NMR** (126 MHz, $CDCl_3$): δ/ppm = 109.1 (m$_c$), 120.9 (q, *J* = 274.3 Hz), 123.8 (t, *J* = 16.6 Hz), 124.5, 129.4 (2C), 131.4 (2C), 136.6, 144.2 (dm, *J* = 249.3 Hz, 2C), 144.6 (dm, *J* = 259.5 Hz, 2C). **^{19}F NMR** (471 MHz, $CDCl_3$): δ/ppm = –141.4 (m$_c$, 2F), –140.3 (m$_c$, 2F), –56.3 (t, *J* = 21.8, 3F). **HRMS** (APCI) calculated for $C_{13}H_4ClF_7^+$ [M$^+$]: 327.9885; found: 327.9882. **IR** (ATR): $\tilde{\nu}$/cm^{-1} = 2925, 2854, 1658, 1599, 1567, 1473, 1396, 1333, 1249, 1192, 1148, 1090, 1014, 979, 831, 707.

2,2',3,5,6-Pentafluoro-[1,1'-biphenyl]-4-carbonitrile (3ld)

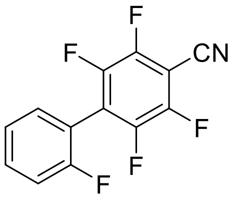

3ld
$C_{13}H_4F_5N$
M = 269.17 g/mol
rs = 63:37:0

Prepared according to **GP2** from the (*E*)-1-(2-fluorophenyl)-2-(trimethylsilyl)diazene (**1l**, 58.9 mg, 0.300 mmol, 1.0 equiv) and 2,3,4,5,6-pentafluorobenzonitrile (**2d**, 114 µL, 174 mg, 0.900 mmol, 3.0 equiv). The reaction mixture was stirred at room temperature for 12 h (slow evolution of N_2). Purification by flash column chromatography on silica gel using *n*-pentane:dichloromethane (90:10) as eluent afforded the *major* isomer *para*-**3ld** (45.6 mg, 0.169 mmol, 56%) as a white solid.

Analytical data for *para*-**3ld** (*major* isomer):
R_f = 0.19 (*n*-pentane:dichloromethane 9:1). **M.p.**: 91–93 °C (*n*-pentane). **^{1}H NMR** (500 MHz, $CDCl_3$): δ/ppm = 7.20–7.25 (m, 1H), 7.28 (m_c, 1H), 7.33 (m_c, 1H), 7.51 (m_c, 1H). **^{13}C{1H} NMR** (126 MHz, $CDCl_3$): δ/ppm = 94.2 (m_c), 107.5 (m_c), 113.7 (d, *J* = 15.4 Hz), 116.6 (d, *J* = 20.9 Hz), 121.8 (app t, *J* = 18.1 Hz), 124.7 (d, *J* = 3.7 Hz), 131.6, 132.9 (d, *J* = 8.6 Hz), 144.2 (dm, *J* = 253.6 Hz, 2C), 147.4 (dm, *J* = 259.8 Hz, 2C), 159.8 (d, *J* = 254.2 Hz). **^{19}F NMR** (471 MHz, $CDCl_3$): δ/ppm = –136.9 (m_c, 2F), –132.4 (m_c, 2F), –111.9 (m_c). **HRMS** (APCI) calculated for $C_{13}H_5F_5N^+$ [$(M+H)^+$]: 270.0337; found: 270.0338. **IR** (ATR): $\tilde{\nu}$/cm^{-1} = 3069, 2922, 2852, 2246, 1649, 1484, 1443, 1324, 1269, 1227, 1187, 1107, 981, 859, 801, 761.

(4'-Chloro-2,3,5,6-tetrafluoro-[1,1'-biphenyl]-4-yl)(phenyl)methanone (3ie)

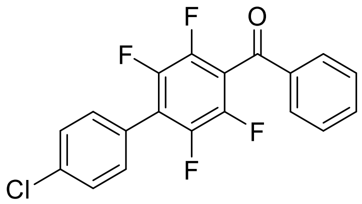

3ie
$C_{19}H_9ClF_4O$
M = 364.72 g/mol
rs = 80:8:12

Prepared according to **GP2** from the (*E*)-1-(4-chlorophenyl)-2-(trimethylsilyl)diazene (**1i**, 63.8 mg, 0.300 mmol, 1.0 equiv) and 2,3,4,5,6-pentafluorobenzophenone (**2e**, 245 mg, 0.900 mmol, 3.0 equiv). Purification by flash column chromatography on silica gel using *n*-pentane:dichloromethane (90:10) as eluent afforded the title compound **3ie** as a yellowish powder in a mixture of regioisomers. The mixture was triturated with a minimum amount of cold *n*-pentane and filtered off affording the *major* isomer *para*-**3ie** (78.7 mg, 0.216 mmol, 72%) as a white solid.

Analytical data for *para*-**3ie** (*major* isomer):
R_f = 0.13 (*n*-pentane:dichloromethane 9:1). **M.p.**: 93–95 °C (*n*-pentane). **^{1}H NMR** (500 MHz, $CDCl_3$): δ/ppm = 7.46 (d, *J* = 8.6 Hz, 2H), 7.50–7.57 (m, 4H), 7.70 (tt, *J* = 7.1, 1.3 Hz, 1H), 7.93 (d, *J* = 7.7 Hz, 2H). **^{13}C{1H} NMR** (126 MHz, $CDCl_3$): δ/ppm = 118.3 (t, *J* = 20.3 Hz), 121.7 (t, *J* = 16.2 Hz), 125.3, 129.2 (2C), 129.3 (2C), 129.9 (2C), 131.6 (2C), 135.1, 136.1, 136.2, 143.79 (dm, *J* = 247.9 Hz, 2C), 143.90 (dm, *J* = 241.5 Hz, 2C), 186.3. **^{19}F NMR** (471 MHz, $CDCl_3$): δ/ppm = –142.0 (m_c, 2F), –140.5 (m_c, 2F). **HRMS** (APCI) calculated for $C_{19}H_{10}ClF_4O^+$ [$(M+H)^+$]: 365.0351; found: 365.0352. **IR** (ATR): $\tilde{\nu}$/cm^{-1} = 3098, 3053, 2922, 1669, 1596, 1499, 1466, 1393, 1302, 1248, 1181, 1089, 1009, 974, 879, 838, 801, 753.

2-(4-Bromophenyl)-1,3,4,5,6,7,8-heptafluoronaphthalene (3hf)

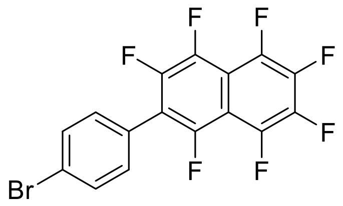

3hf
$C_{16}H_4BrF_7$
M = 409.10 g/mol
rs = 76:24

Prepared according to **GP2** from the (*E*)-1-(4-bromophenyl)-2-(trimethylsilyl)diazene (**1h**, 77.2 mg, 0.300 mmol, 1.0 equiv) and octafluoronaphtalene (**2f**, 245 mg, 0.900 mmol, 3.0 equiv). Purification by flash column chromatography on silica gel using *n*-pentane as eluent afforded the title compound **3hf** as a white solid in a mixture of regioisomers. The mixture was triturated with a minimum amount of cold *n*-pentane and filtered off affording the *major* isomer β-**3hf** (79.7 mg, 0.195 mmol, 65%) as a white solid.

Analytical data for β-**3hf** (*major* isomer):

R$_f$ = 0.30 (*n*-pentane:dichloromethane 9:1). **M.p.**: 154–156 °C (*n*-pentane). **^{1}H NMR** (700 MHz, $CDCl_3$): δ/ppm = 7.40 (d, *J* = 8.3 Hz, 2H), 7.67 (d, *J* = 8.3 Hz, 2H). **^{13}C{1H} NMR** (176 MHz, $CDCl_3$): δ/ppm = 108.2 (t, *J* = 14.3 Hz), 111.1 (t, *J* = 10.9 Hz), 118.5 (t, *J* = 20.2 Hz), 124.1, 126.1, 132.0 (2C), 132.2 (2C), 139.0 (dt, *J* = 256.5 Hz), 140.0 (dt, *J* = 258.3 Hz), 141.1 (dm, *J* = 256.5 Hz), 192.3 (dm, *J* = 243.9 Hz), 141.8 (dm, *J* = 265.5 Hz), 145.3 (dm, *J* = 249.3 Hz), 149.3 (dm, *J* = 256.5 Hz). **^{19}F NMR** (659 MHz, $CDCl_3$): δ/ppm = –155.1 (m_c), –152.9 (t, *J* = 18.4 Hz), –148.2 (m_c), –145.9 (dt, *J* = 58.8, 17.6 Hz), –143.5 (dt, *J* = 71.3, 16.6 Hz), –137.2 (m_c), –121.3 (dd, *J* = 70.8, 18.0 Hz). **HRMS** (APCI) calculated for $C_{16}H_4BrF_7^+$ [M^+]: 407.9380; found: 407.9381. **IR** (ATR): $\tilde{\nu}$/cm^{-1} = 2922, 2853, 1650, 1591, 1522, 1492, 1429, 1386, 1324, 1213, 1124, 1100, 1070, 1010, 944, 901, 830, 801, 758.

2,2',3,3',4,5,5',6,6'-Nonafluoro-4''-methoxy-1,1':4',1''-terphenyl (3cg)

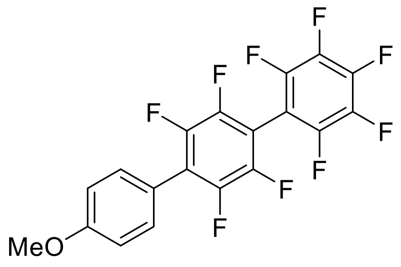

3cg
$C_{19}H_7F_9O$
M = 422.04 g/mol
rs = 76:7:17

Prepared according to **GP2** from the (*E*)-1-(4-methoxyphenyl)-2-(trimethylsilyl)diazene (**1c**, 62.5 mg, 0.300 mmol, 1.0 equiv) and decafluorobiphenyl (**2g**, 301 mg, 0.900 mmol, 3.0 equiv). Purification by flash column chromatography on silica gel using *n*-pentane:dichloromethane (90:10) as eluent afforded the title compound **3cg** as a white solid in a mixture of regioisomers. The mixture was recrystallized from isopropanol affording the *major* isomer *para*-**3cg** (77.2 mg, 0.183 mmol, 61%) as white crystals.

Analytical data for *para*-**3cg** (*major* isomer):
R$_f$ = 0.31 (*n*-pentane:dichloromethane 9:1). **M.p.**: 164–166 °C (isopropanol). **^{1}H NMR** (500 MHz, $CDCl_3$): δ/ppm = 3.89 (s, 3H), 7.06 (d, *J* = 8.1 Hz, 2H), 7.48 (d, *J* = 8.1 Hz, 2H). **^{13}C{1H} NMR** (126 MHz, $CDCl_3$): δ/ppm = 55.5, 102.8 (t, *J* = 18.2 Hz), 104.4 (t, *J* = 17.1 Hz), 114.4 (2C), 119.0, 122.9 (t, *J* = 17.2 Hz), 131.6 (2C), 138.1 (dm, *J* = 255.5 Hz, 2C), 142.5 (dm, *J* = 247.7 Hz), 144.2 (dm, *J* = 247.6 Hz, 2C), 144.7 (dm, *J* = 255.2 Hz, 2C), 144.8 (dm, *J* = 247.1 Hz, 2C), 160.7. **^{19}F NMR** (471 MHz, $CDCl_3$): δ/ppm = = –160.7 (m_c, 2F), –150.6 (m_c), –143.0

(m_c, 2F), –139.1 (m_c, 2F), –137.4 (m_c, 2F). **HRMS** (APCI) calculated for $C_{19}H_7F_9^+$ [M$^+$]: 422.0348; found: 422.0340. **IR** (ATR): $\tilde{\nu}$/cm^{-1} = 3025, 2960, 2934, 2840, 1650, 1604, 1524, 1489, 1461, 1410, 1292, 1252, 1176, 1108, 1032, 998, 968, 835, 802.

2,3,5,6-Tetrafluoro-4'-methoxy-4-vinyl-1,1'-biphenyl (3ck)

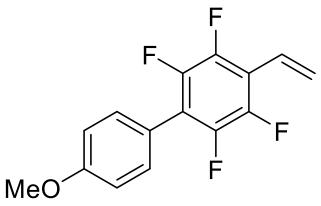

3ck
$C_{15}H_{10}F_4O$
M = 282.24 g/mol
rs = 79:12:9

Prepared according to **GP2** from the (*E*)-1-(4-methoxyphenyl)-2-(trimethylsilyl)diazene (**1c**, 62.5 mg, 0.300 mmol, 1.0 equiv) and 2,3,4,5,6-pentafluorostyrene (**2k**, 124 µL, 175 mg, 0.900 mmol, 3.0 equiv). Purification by flash column chromatography on silica gel using *n*-pentane as eluent afforded the title compound **3ck** as a white solid in a mixture of regioisomers. The mixture was recrystallized from cyclohexane affording the *major* isomer *para*-**3ck** (54.6 mg, 0.193 mmol, 64%) as a white solid.

Analytical data for *para*-**3ck** (*major* isomer):
R$_f$ = 0.30 (*n*-pentane:dichloromethane 9:1). **M.p.**: 87–89 °C (cyclohexane). **^{1}H NMR** (700 MHz, $CDCl_3$): δ/ppm = 3.87 (s, 3H), 5.73 (d, *J* = 11.9 Hz, 1H), 6.15 (d, *J* = 18.3 Hz, 1H), 6.74 (dd, *J* = 18.3, 11.9 Hz, 1H), 7.02 (d, *J* = 8.8 Hz, 2H), 7.43 (d, *J* = 8.8 Hz, 2H). **^{13}C{1H} NMR** (176 MHz, $CDCl_3$): δ/ppm = 55.4, 114.2 (2C), 115.3 (t, *J* = 13.7 Hz), 119.0 (t, *J* = 17.0 Hz), 119.7, 122.6, 123.4 (t, *J* = 7.8 Hz), 131.6 (2C), 144.5 (dm, *J* = 244.8 Hz, 2C), 145.1 (dm, *J* = 249.6 Hz, 2C), 160.3. **^{19}F NMR** (659 MHz, $CDCl_3$): δ/ppm = –145.8 (app dd, *J* = 21.8, 12.6 Hz, 2F), –144.6 (app dd, *J* = 21.5, 12.5 Hz, 2F). **HRMS** (APCI) calculated for $C_{15}H_{10}F_4O^+$ [M$^+$]: 282.0663; found: 282.0658. **IR** (ATR): $\tilde{\nu}$/cm^{-1} = 3032, 2999, 2962, 2937, 2900, 2837, 1604, 1516, 1468, 1408, 1290, 1244, 1176, 1110, 1088, 1032, 956, 931, 825, 776.

2',3',5',6'-Tetrafluoro-4'-vinyl-[1,1'-biphenyl]-4-carbonitrile (3dk)

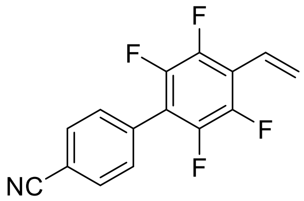

3dk
$C_{15}H_7F_4N$
M = 277.22 g/mol
rs = 83:10:7

Prepared according to **GP2** from the (*E*)-4-((trimethylsilyl)diazenyl)benzonitrile (**1d**, 61.0 mg, 0.300 mmol, 1.0 equiv) and 2,3,4,5,6-pentafluorostyrene (**2k**, 414 µL, 582 mg, 3.00 mmol, 10 equiv). Purification by flash column chromatography on silica gel using *n*-pentane:dichloromethane (70:30) as eluent afforded the title compound **3dk** as a white solid in a mixture of regioisomers. The mixture was triturated with a minimum amount of cold *n*-pentane and filtered off affording the *major* isomer *para*-**3dk** (60.0 mg, 0.216 mmol, 72%) as a white solid.

Analytical data for *para*-**3dk** (*major* isomer):
R_f = 0.28 (*n*-pentane:dichloromethane 7:3). **M.p.**: 136–138 °C (*n*-pentane). **^{1}H NMR** (500 MHz, $CDCl_3$): δ/ppm = 5.79 (d, *J* = 11.9 Hz, 1H), 6.19 (d, *J* = 18.1 Hz, 1H), 6.75 (dd, *J* = 18.1, 11.9 Hz, 1H), 7.60 (d, *J* = 8.3 Hz, 2H), 7.79 (d, *J* = 8.3 Hz, 2H). **^{13}C{1H} NMR** (126 MHz, $CDCl_3$): δ/ppm = 113.2, 117.2 (q, *J* = 15.2 Hz, 2C), 118.4, 122.3, 124.7 (t, *J* = 8.2 Hz), 131.1 (2C), 132.5 (2C), 143.8 (dm, *J* = 251.7 Hz, 2C), 145.1 (dm, *J* = 249.6 Hz, 2C), 160.3. **^{19}F NMR** (471 MHz, $CDCl_3$): δ/ppm = –145.0 (app dd, *J* = 20.9, 12.1 Hz, 2F), –143.2 (app dd, *J* = 21.1, 12.3 Hz, 2F). **HRMS** (APCI) calculated for $C_{15}H_8F_4N^+$ [(M+H)$^+$]: 278.0588; found: 278.0582. **IR** (ATR): $\tilde{\nu}$/cm^{-1} = 3065, 2233, 1629, 1466, 1399, 1308, 1273, 1195, 1092, 1025, 955, 925, 842, 777.

6 Scale-Up Experiment

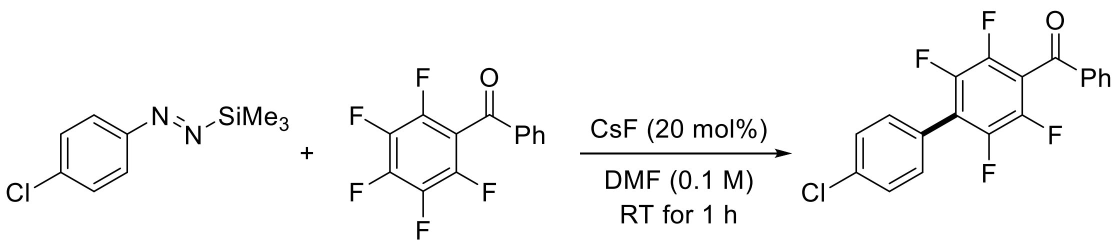

1i (1.30 mmol) **2e** (3.0 equiv) *1.97 equiv reisolated* **3ie**: 80:8:12, 79%

Scheme S1. Scale-up experiment for the synthesis of **3ie**.

In an argon-filled glovebox, an oven-dried 50-mL Schlenk flask equipped with a magnetic stirring bar was charged with cesium fluoride (39.5 mg, 0.260 mmol, 20 mol%) and 2,3,4,5,6-pentafluorobenzophenone (**2e**, 1.06 g, 3.90 mmol, 3.0 equiv). The reaction vessel was sealed with a septum, transferred out of the glovebox, and connected to a nitrogen manifold. Anhydrous DMF (5 mL) was added, and to the vigorously stirred mixture was added in a fine stream over a minute a solution of (*E*)-1-(4-chlorophenyl)-2-(trimethylsilyl)diazene (**1i**, 277 mg, 1.30 mmol, 1.0 equiv) in anhydrous DMF (5 mL) at room temperature. A gentle nitrogen evolution starts instantaneously. The silylated aryldiazene was fully converted within an hour. The crude mixture was diluted with ethyl acetate (50 mL) and water (25 mL). The layers were separated, and the organic phase was washed with brine (3 x 25 mL). The organic layer was dried over anhydrous $MgSO_4$, filtered, and concentrated under reduced pressure. The resulting crude residue was further washed with *n*-pentane (3 x). Purification by flash column chromatography on silica gel using *n*-pentane:dichloromethane (100:0 → 9:1) as eluent afforded the *major* isomer *para*-**3ie** (372 mg, 1.02 mmol, 79%) as a white solid. The unreacted starting material 2,3,4,5,6-pentafluorobenzophenone (**2e**) could be fully recovered (697 mg, 2.56 mmol, 1.97 equiv recovered) as a colorless oil.

The NMR spectroscopic and mass spectrometric data were in accordance with those reported on a 0.300 mmol scale.

7 Derivatization of the Coupling Products – Experimental Details

(*E*)-2,3,5,6-Tetrafluoro-4'-methoxy-4-styryl-1,1'-biphenyl (7cka)

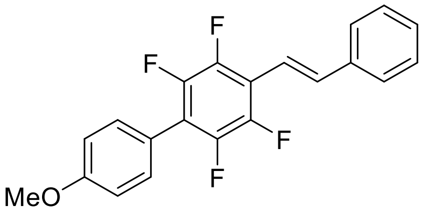

7cka
$C_{21}H_{14}F_4O$
M = 358.34 g/mol

According to a modified literature procedure,[S13] an oven-dried 15-mL high pressure Schlenk tube equipped with a magnetic stirring bar and a teflon plug valve was charged with the alkene **3ck** (28.2 mg, 0.100 mmol, 1.0 equiv), and $Pd(OAc)_2$ (0.5 mg, 2 µmol, 2 mol%). Anhydrous DMF (0.5 mL) was added, followed by freshly distilled triethylamine (70 µL, 51 mg, 0.50 mmol, 5.0 equiv) and iodobenzene (**6a**, 17 µL, 31 mg, 0.15 mmol, 1.5 equiv). The resulting mixture was stirred at 100 °C for 20 h. After cooling down to room temperature, the suspension was diluted with *tert*-butyl methyl ether (15 mL) and water (10 mL). The layers were separated, and the aqueous phase was extracted thrice with *tert*-butyl methyl ether (3 x 10 mL). The combined organic layers were dried over anhydrous $MgSO_4$, filtered, and concentrated under reduced pressure. The resulting crude residue was washed thrice with *n*-pentane. Purification by flash column chromatography on silica gel using *n*-pentane:dichloromethane (90:10) as eluent afforded the title compound **7cka** (33.8 mg, 94.3 µmol, 94%) as a white solid.

R_f = 0.21 (*n*-pentane:dichloromethane 9:1). **M.p.**: 184–186 °C (dichloromethane). **^{1}H NMR** (500 MHz, $CDCl_3$): δ/ppm = 3.88 (s, 3H), 7.03 (d, *J* = 8.5 Hz, 2H), 7.13 (d, *J* = 17.0 Hz, 1H), 7.34 (app t, *J* = 7.7 Hz, 1H), 7.41 (app t, *J* = 7.7 Hz, 2H), 7.45 (d, *J* = 8.5 Hz, 2H), 7.53 (d, *J* = 17.0 Hz, 1H), 7.57 (d, *J* = 7.7 Hz, 2H). **^{13}C{1H} NMR** (126 MHz, $CDCl_3$): δ/ppm = 55.5, 114.2, 114.3 (2C), 115.5 (t, *J* = 13.8 Hz), 118.5 (t, *J* = 16.7 Hz), 119.8, 127.1 (2C), 129.0 (3C), 131.6 (2C), 136.9, 137.1 (t, *J* = 8.6 Hz), 144.1 (dm, *J* = 249.5 Hz, 2C), 145.1 (dm, *J* = 248.5 Hz, 2C), 160.3. **^{19}F NMR** (471 MHz, $CDCl_3$): δ/ppm = –145.7 (app dd, *J* = 21.1, 11.5 Hz, 2F), –143.8 (app dd, *J* = 21.6, 12.1 Hz, 2F). **HRMS** (APCI) calculated for $C_{21}H_{14}F_4O^+$ [M^+]: 358.0976; found: 358.0974. **IR** (ATR): $\tilde{\nu}$/cm^{-1} = 2922, 2851, 1605, 1517, 1463, 1410, 1293, 1244, 1174, 1109, 1026, 961, 821, 754.

Methyl (*E*)-4-(2-(4'-cyano-2,3,5,6-tetrafluoro-[1,1'-biphenyl]-4-yl)vinyl)benzoate (7dkb)

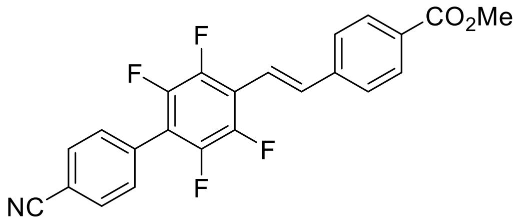

7dkb
$C_{23}H_{13}F_4NO_2$
M = 411.36 g/mol

According to a modified literature procedure,[S13] an oven-dried 15-mL high pressure Schlenk tube equipped with a magnetic stirring bar and a teflon plug valve was charged with the alkene **3dk** (27.7 mg, 0.100 mmol, 1.0 equiv), methyl 4-iodobenzoate (**6b**, 39.3 mg, 0.150 mmol, 1.5 equiv), and $Pd(OAc)_2$ (0.5 mg, 2 µmol, 2 mol%). Anhydrous DMF (0.5 mL) was added, followed by freshly distilled triethylamine (70 µL, 51 mg, 0.50 mmol, 5.0 equiv). The resulting mixture was stirred at 100 °C for 20 h. After cooling down to room temperature, the suspension was diluted with dichloromethane (15 mL) and water (10 mL). The layers were separated, and the aqueous phase was extracted thrice with dichloromethane (3 x 10 mL). The combined organic layers were dried over anhydrous $MgSO_4$, filtered, and concentrated under reduced pressure. The resulting crude residue was washed thrice with *n*-pentane. Purification by flash column chromatography on silica gel using *n*-pentane:dichloromethane (1:1) as eluent afforded the title compound **7dkb** (39.6 mg, 96.3 µmol, 96%) as a white solid.

R_f = 0.13 (*n*-pentane:dichloromethane 1:1). **M.p.**: 221–223 °C (dichloromethane). **^{1}H NMR** (500 MHz, CD_2Cl_2): δ/ppm = 3.95 (s, 3H), 7.29 (d, J = 16.9 Hz, 1H), 7.65 (d, J = 16.9 Hz, 1H), 7.67–7.73 (m, 4H), 7.86 (d, J = 8.3 Hz, 2H), 8.10 (d, J = 8.3 Hz, 2H). **^{13}C{1H} NMR** (126 MHz, CD_2Cl_2): δ/ppm = 52.5, 113.5, 116.3, 117.2 (t, J = 13.3 Hz), 117.6 (t, J = 16.6 Hz), 118.6, 127.3 (2C), 130.4 (2C), 130.9, 131.3 (2C), 132.5, 132.8 (2C), 137.1 (t, J = 8.7 Hz), 141.2, 144.3 (dm, J = 243.0 Hz, 2C), 145.5 (dm, J = 250.3 Hz, 2C), 166.8. **^{19}F NMR** (471 MHz, CD_2Cl_2): δ/ppm = –145.3 (app dd, J = 20.7, 11.8 Hz, 2F), –142.9 (app dd, J = 20.7, 11.9 Hz, 2F). **HRMS** (APCI) calculated for $C_{23}H_{14}F_4NO_2^+$ [$(M+H)^+$]: 412.0956; found: 412.0952. **IR** (ATR): $\tilde{\nu}$/cm^{-1} = 2955, 2927, 2856, 2226, 1709, 1603, 1468, 1407, 1331, 1275, 1180, 1098, 1040, 1017, 968, 875, 839, 815, 766.

Note. Both **7cka** and **7dkb** are poorly soluble in both $CDCl_3$ and CD_2Cl_2.

8 Control Experiments

General procedure for the control experiments. In an argon-filled glovebox, an oven-dried 1.5-mL screw-caped vial equipped with a magnetic stirring bar was charged with CsF (3.0 mg, 20 µmol, 20 mol%) and the corresponding fluoroarene (0.300 mmol, 3.0 equiv) *if applicable*. The reaction vessel was sealed, transferred out of the glovebox, and connected to a nitrogen manifold using a syringe needle. The indicated dry deuterated solvent (DMF-d_7 or THF-d_8, 0.35 mL) was added, and to the vigorously stirred mixture was added in one portion a solution of the corresponding silylated aryldiazene (0.100 mmol, 1.0 equiv) in the indicated deuterated solvent (0.35 mL) at room temperature. Full conversion of the silylated aryldiazene was monitored by GLC analysis. Upon completion, known amounts of hexafluorobenzene and dibromomethane were added, and the resulting mixture was strirred for 5 min. The solution was then transferred into a J. Young NMR tube and analyzed straightaway by quantitative ^{1}H and ^{19}F NMR spectroscopy. For a more precise integration of ^{1}H and ^{19}F NMR spectra, relaxation delays were set to 30 (or 50) and 200 sec respectively.

8.1 Degradation of the *N*-Aryl-*N*'-Silyldiazenes in the Absence of Polyfluorarene

In the absence of the polyfluoroarene substrate, solvent-dependent competitive reactions were identified (Scheme S2). In THF, the arylsilane **5a** (or **S7**) is formed along with the corresponding bissilylated hydrazine **S8** (or **S9**) formally originating from the addition of the in situ released aryl anion on the N=N bond of the diazene (Scheme S2, top).[S5] In contrast, in DMF the formation of the arylsilane **5a** (or **S7**) is minor, while the aryl metal mainly reacts with the solvent itself furnishing **S10** (or **S11**) (Scheme S2, bottom).

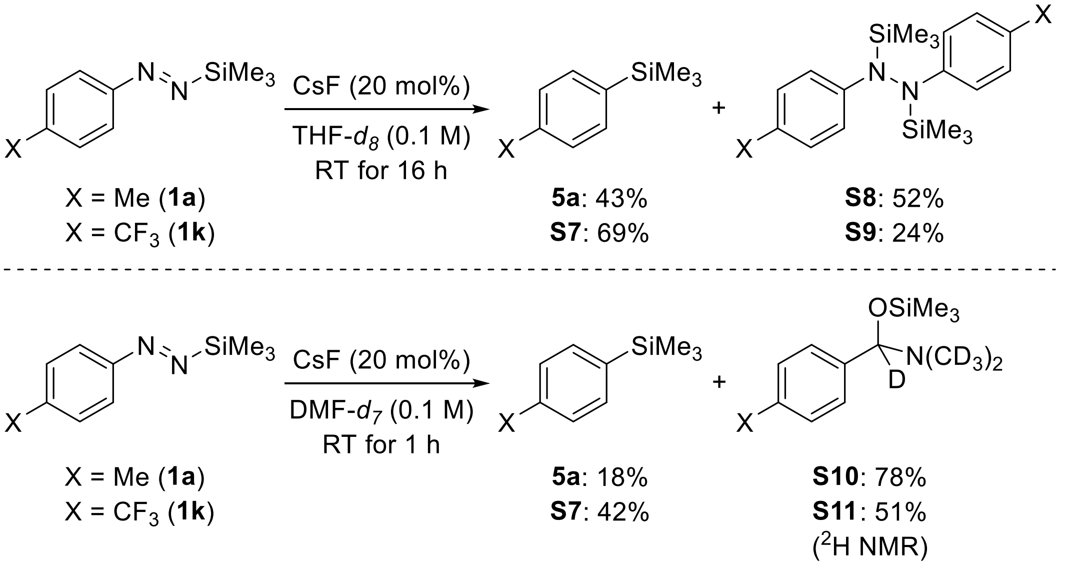

Scheme S2. Influence of the solvent on the degradation of diazenes **1a** and **1k**.

8.2 Competitive Deprotonation – Failed Substrates

The basicity of the system employed hampered the coupling reactions of diazenes with pentafluorobenzene (Scheme S3, top) and 1,2-difluorobenzene (Scheme S3, bottom). The almost quantitative deprotonation of pentafluorobenzene led to a messy mixture resulting from the decomposition of the corresponding pentafluorophenyl anion. 1,2-Difluorobenzene, in turn, underwent *ortho*-formylation through depronomation followed by addition on the electrophilic DMF leading to the tetrahedral intermediate **S12**.

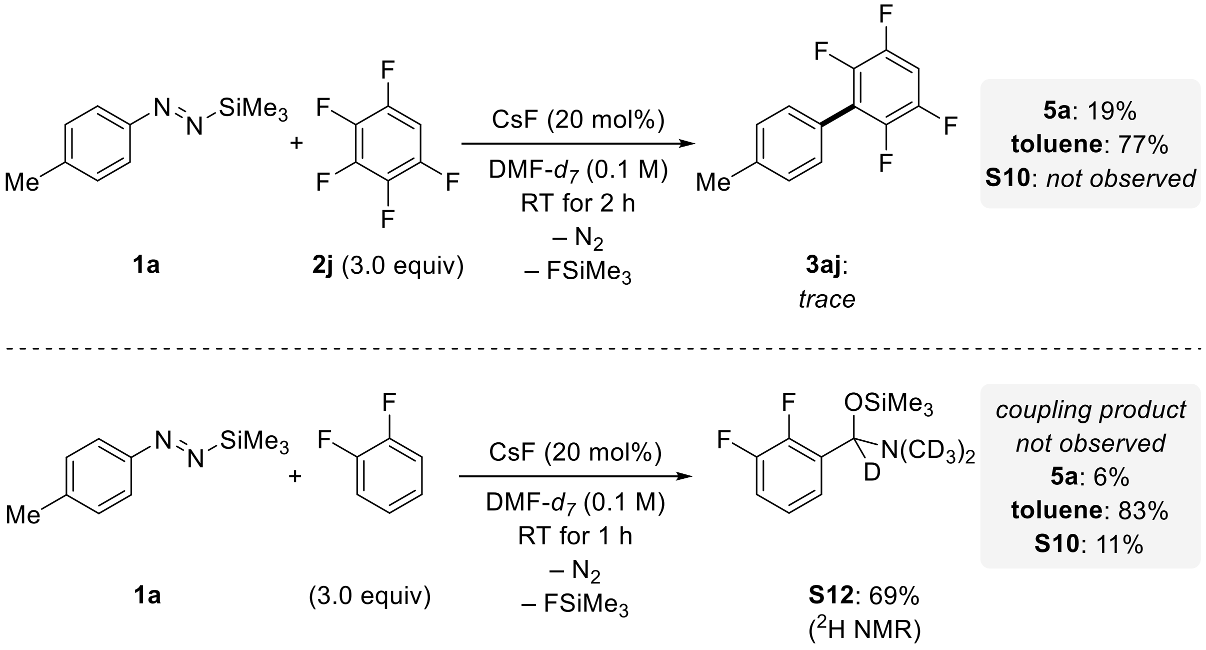

Scheme S3. Competitive deprotonation of pentafluorobenzene and 1,2-difluorobenzene.

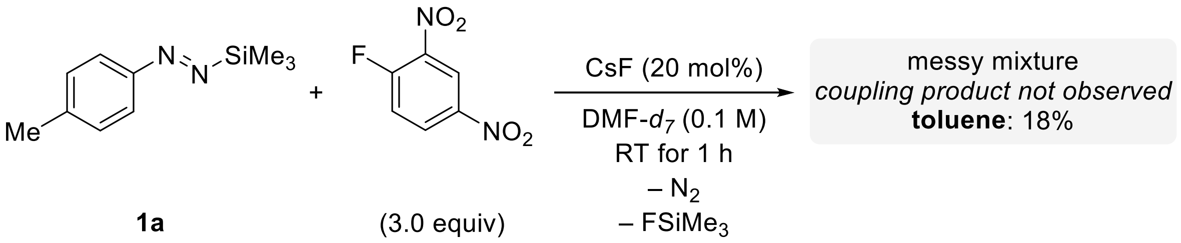

Scheme S4. Failed substrate: 1-fluoro-2,4-dinitrobenzene.

9 NMR Spectra

9.1 *N*-Aryl-*N'*-Silyldiazenes

Figure S1. ^{1}H NMR spectrum (700 MHz, $(CD_3)_2SO$) of 2-(4-iodophenyl)hydrazin-1-ium chloride (**S1**).

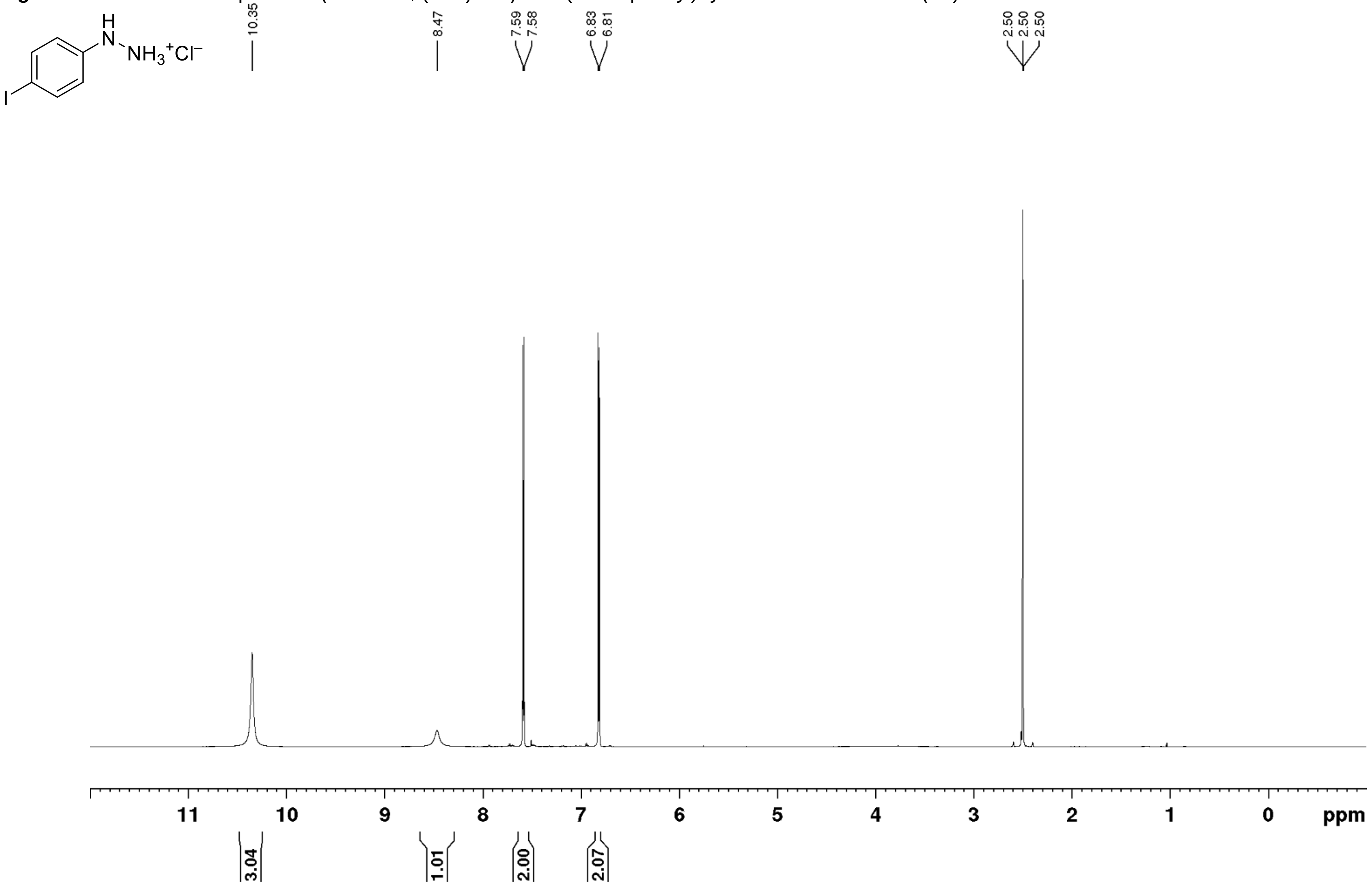

Figure S2. $^{13}C\{^1H\}$ NMR spectrum (126 MHz, $(CD_3)_2SO$) of 2-(4-iodophenyl)hydrazin-1-ium chloride (**S1**).

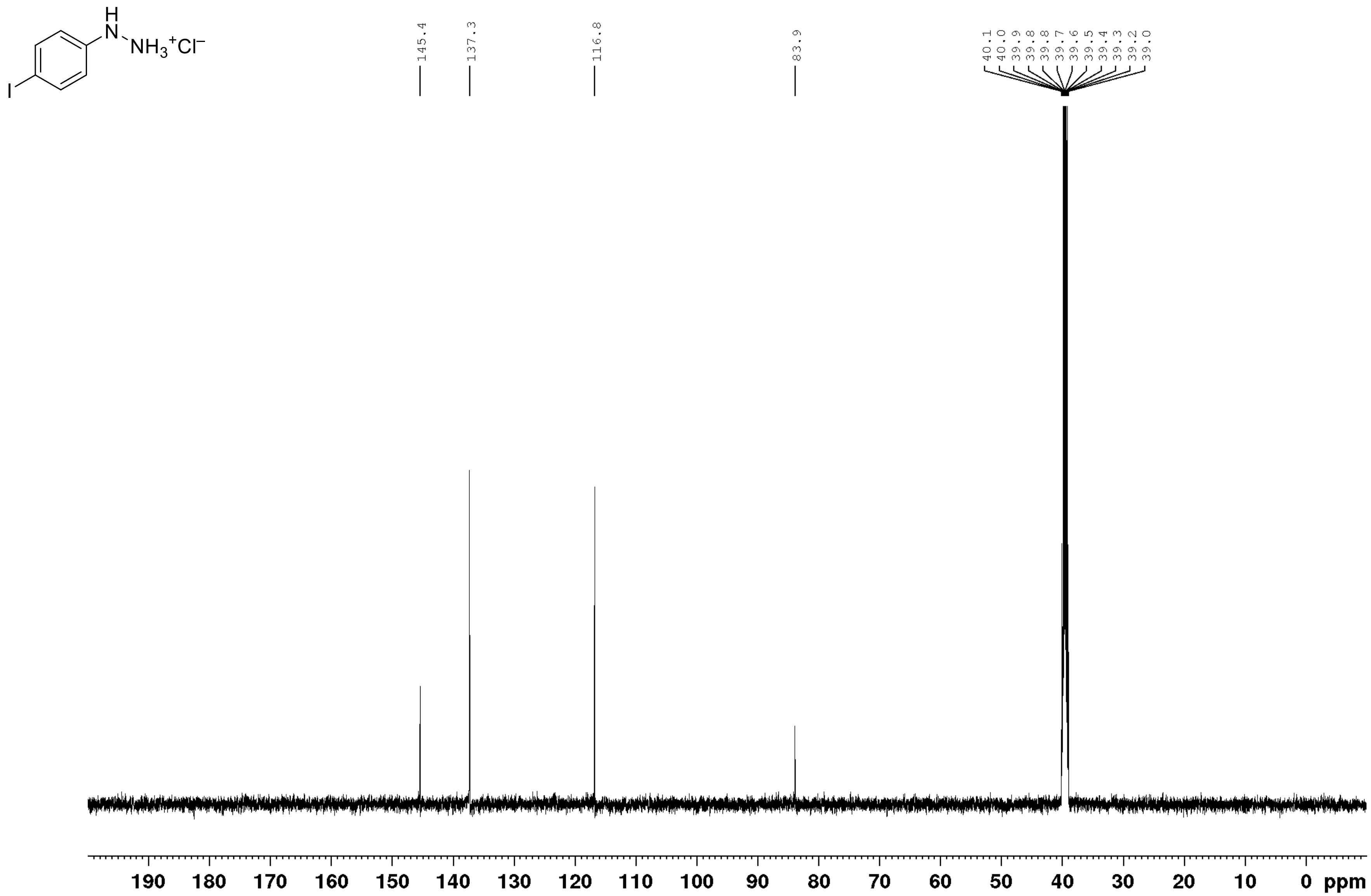

Figure S3. ^{1}H NMR spectrum (500 MHz, C_6D_6) of 1-(4-iodophenyl)-2-(trimethylsilyl)hydrazine (**S2**).

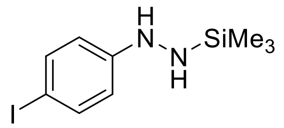

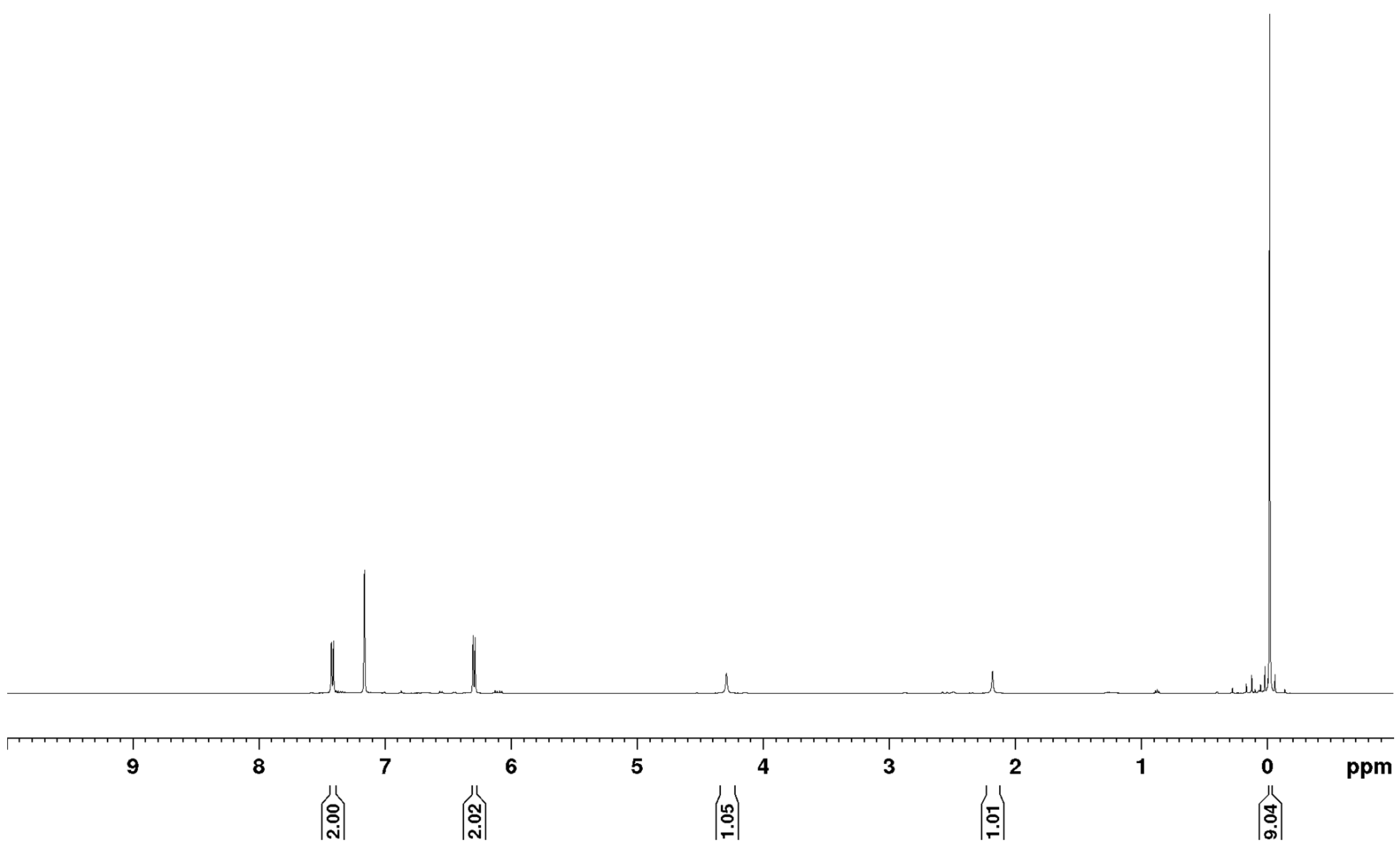

Figure S4. $^{13}C\{^{1}H\}$ NMR spectrum (126 MHz, C_6D_6) of 1-(4-iodophenyl)-2-(trimethylsilyl)hydrazine (**S2**).

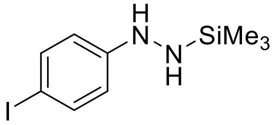

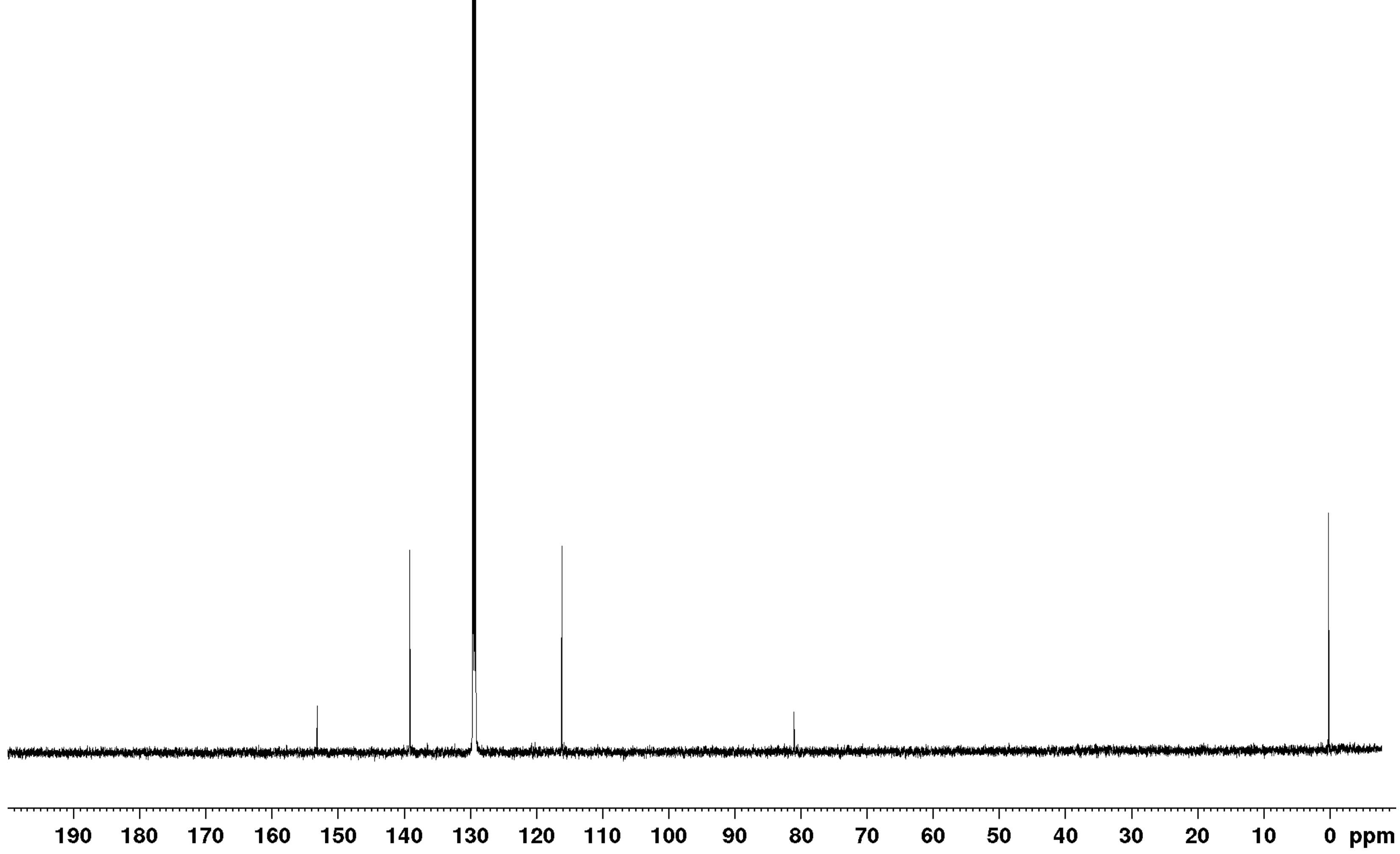

Figure S5. ^{1}H NMR spectrum (500 MHz, $CDCl_3$) of (*E*)-1-(4-iodophenyl)-2-(trimethylsilyl)diazene (**1g**).

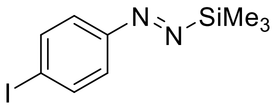

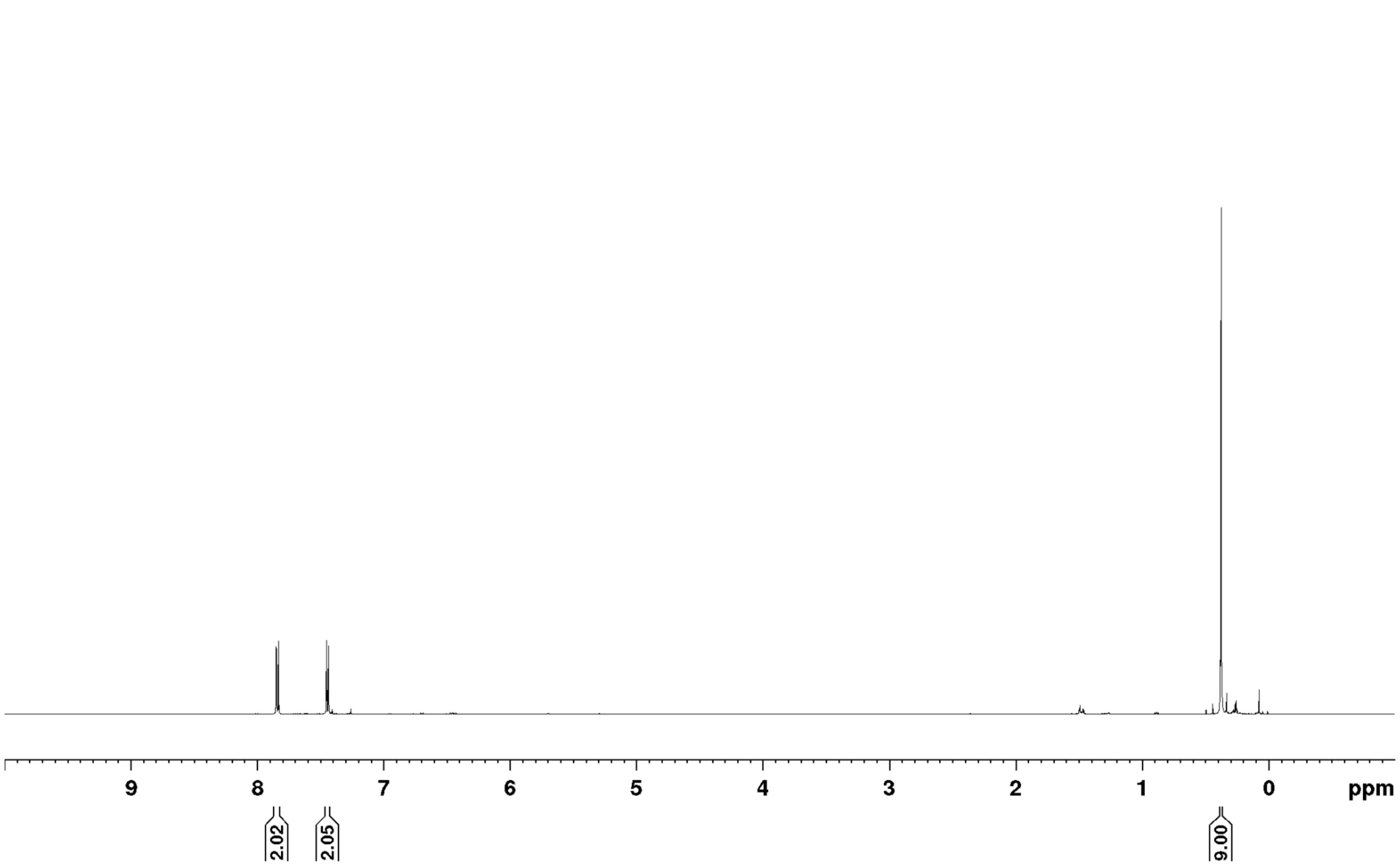

Figure S6. $^{13}C\{^{1}H\}$ NMR spectrum (126 MHz, C_6D_6) of (*E*)-1-(4-iodophenyl)-2-(trimethylsilyl)diazene (**1g**).

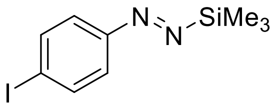

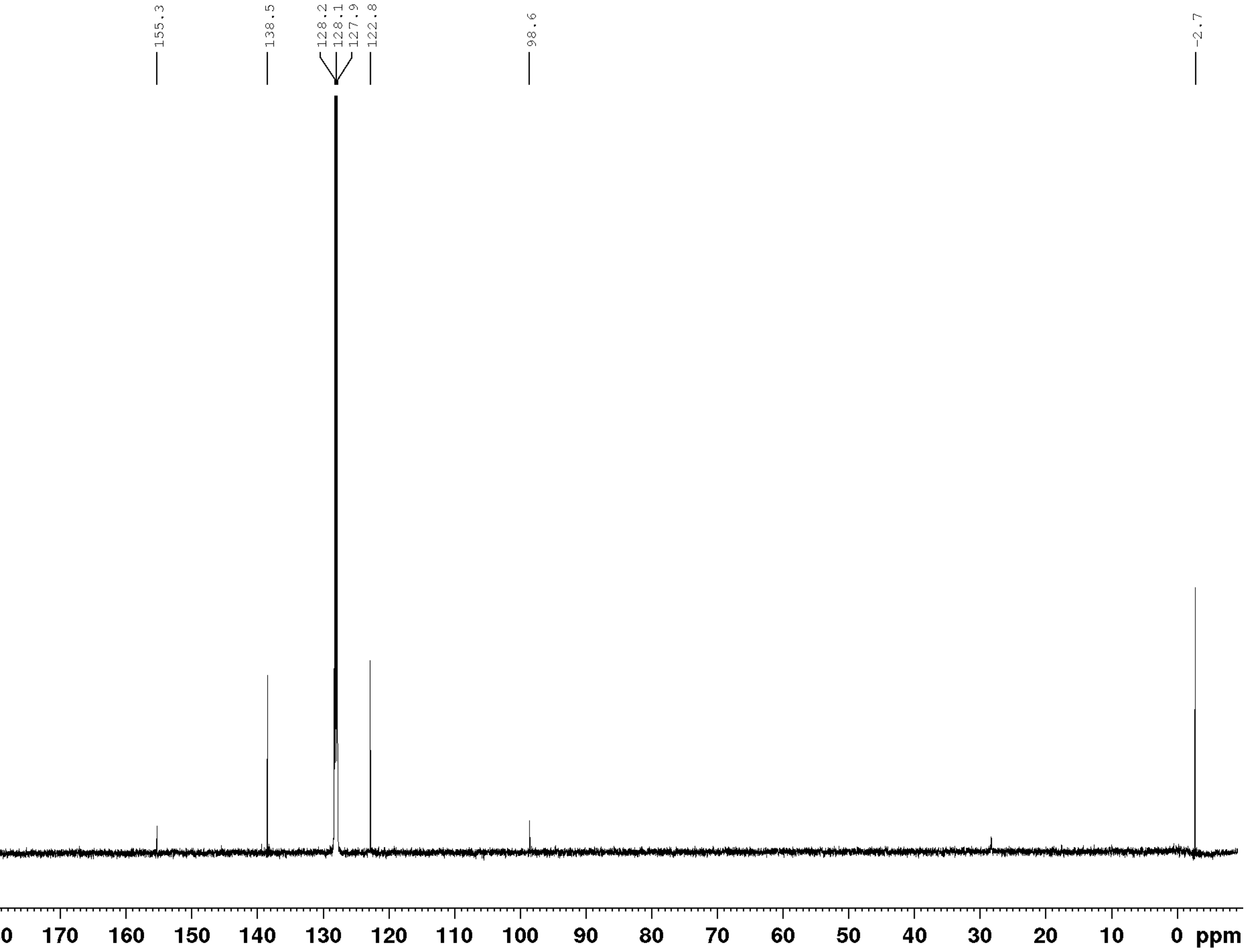

Figure S7. ^{1}H NMR spectrum (500 MHz, $CDCl_3$) of 1-(4-(trifluoromethyl)phenyl)-2-(trimethylsilyl)hydrazine (**S3**).

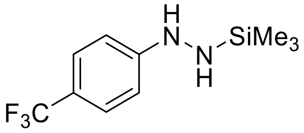

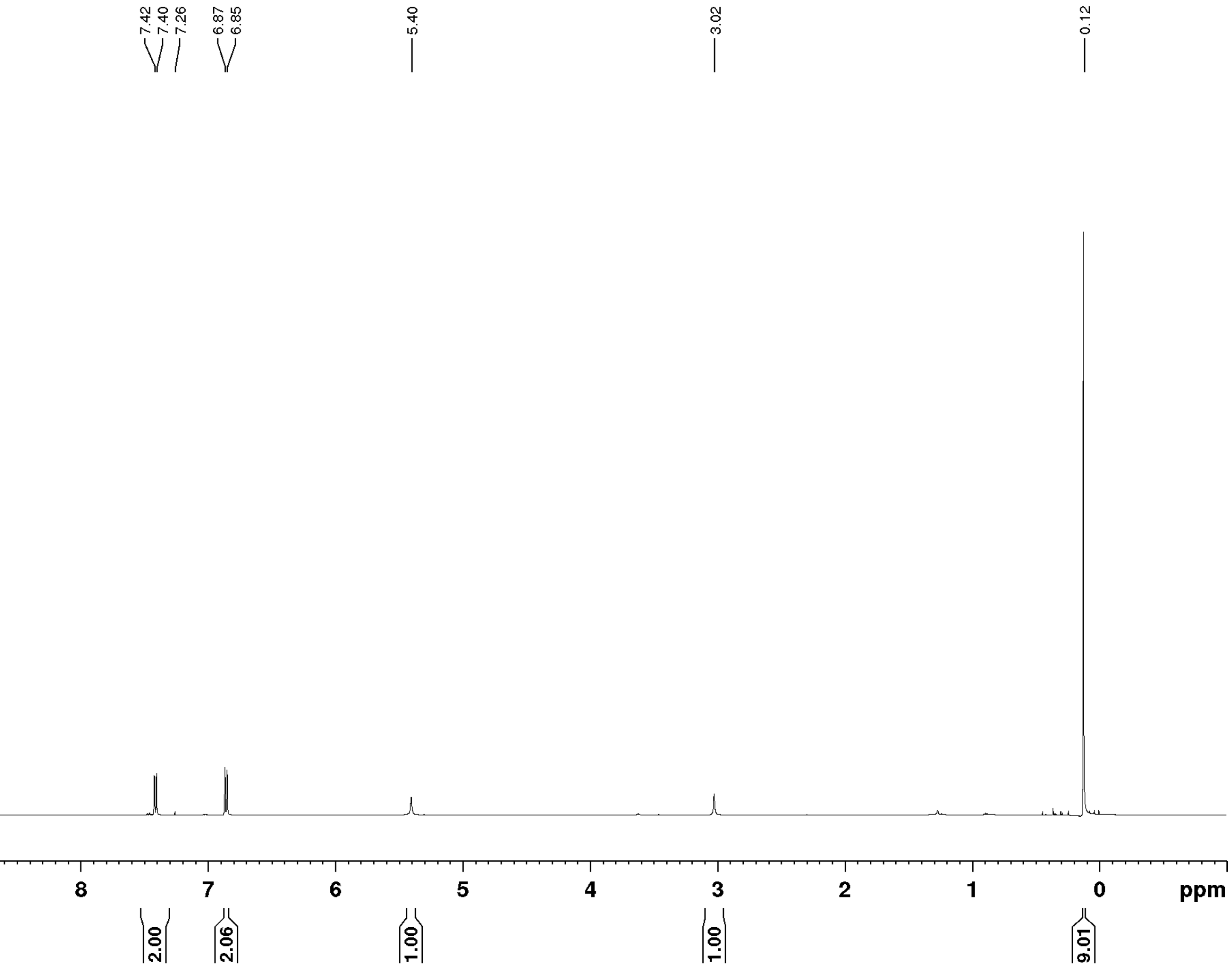

Figure S8. $^{13}C\{^1H\}$ NMR spectrum (126 MHz, $CDCl_3$) of 1-(4-(trifluoromethyl)phenyl)-2-(trimethylsilyl)hydrazine (**S3**).

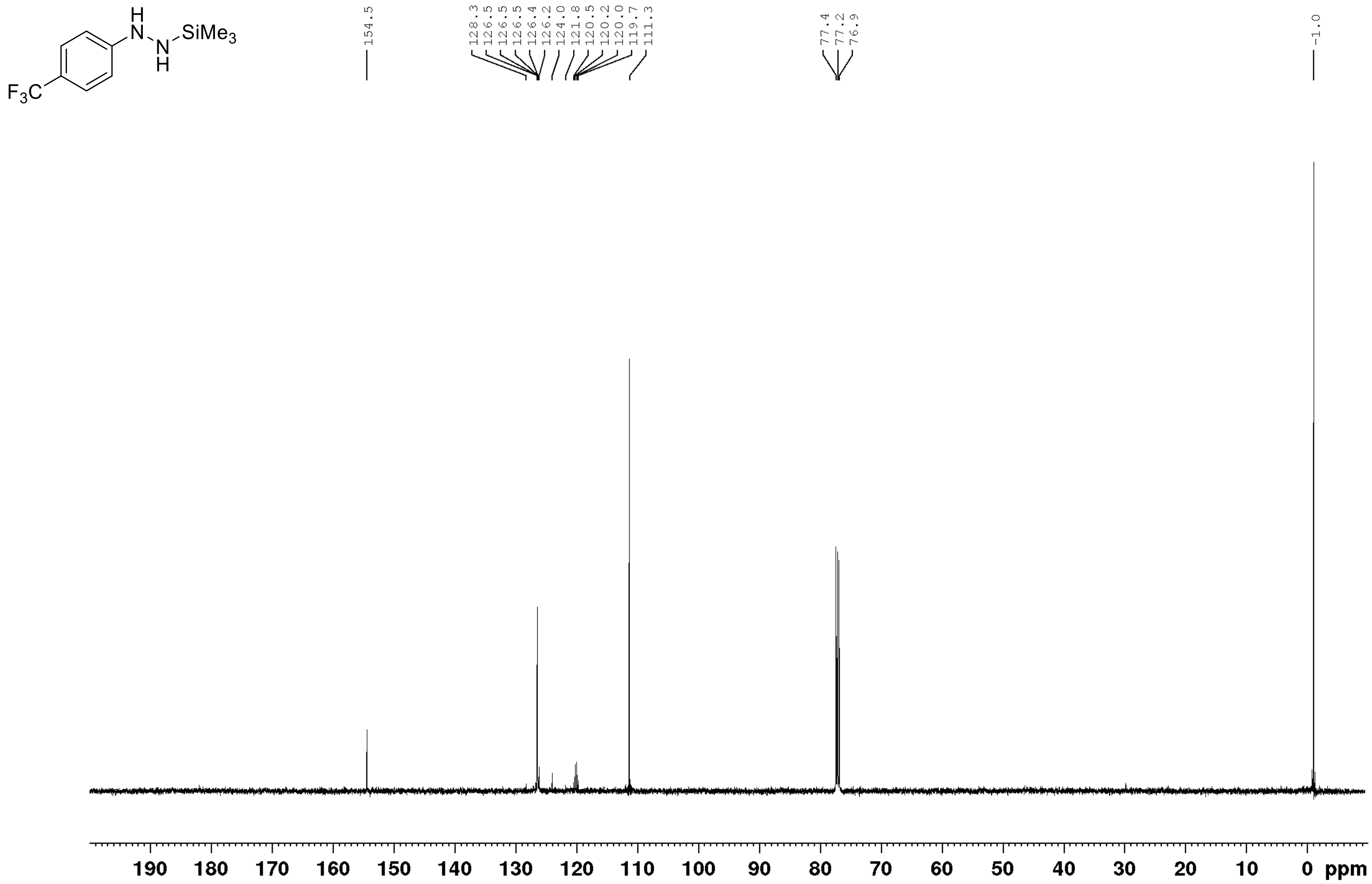

Figure S9. ^{19}F NMR spectrum (471 MHz, $CDCl_3$) of 1-(4-(trifluoromethyl)phenyl)-2-(trimethylsilyl)hydrazine (**S3**).

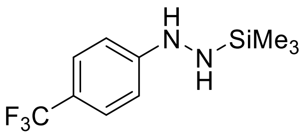

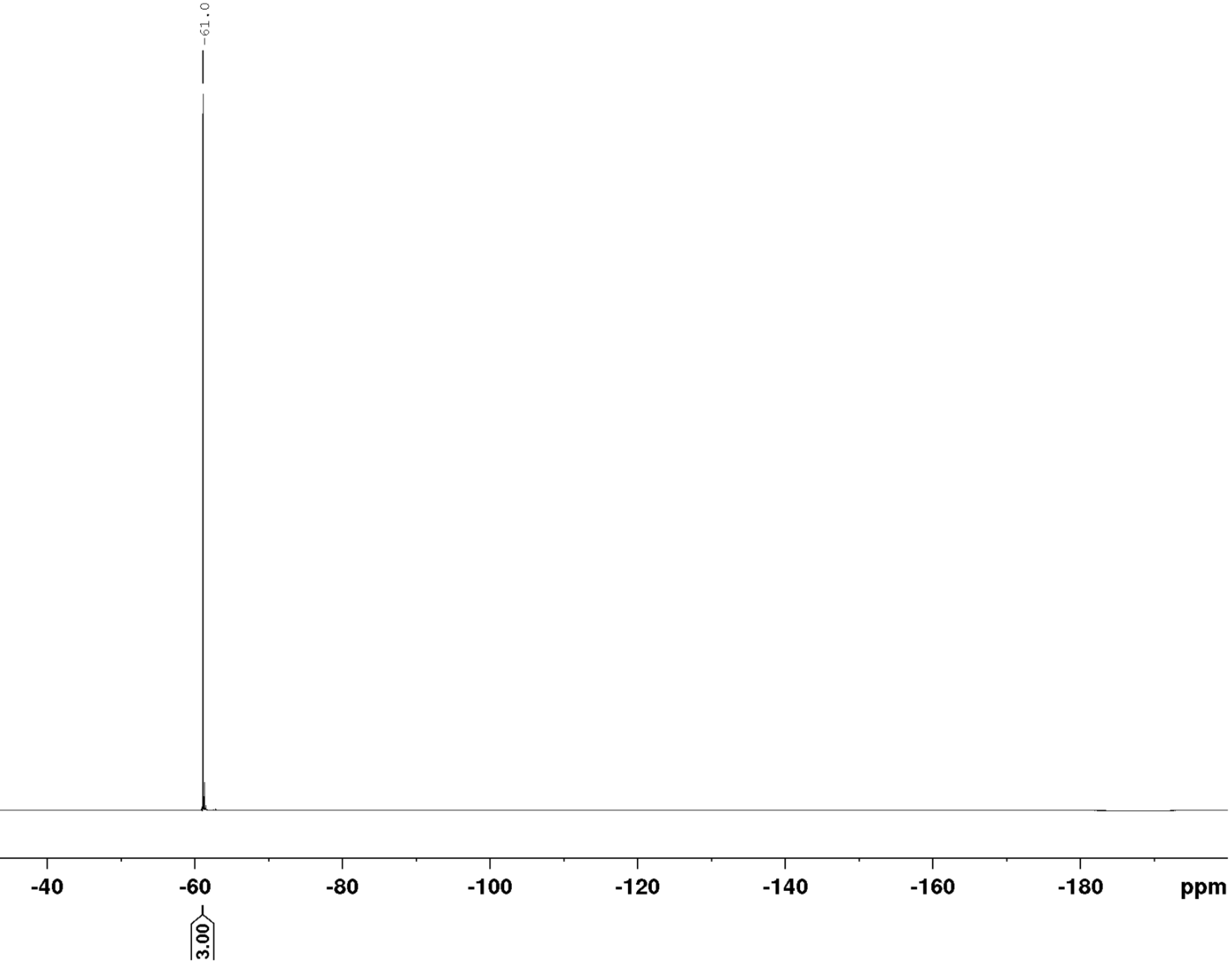

Figure S10. ^{1}H NMR spectrum (500 MHz, $CDCl_3$) of (*E*)-1-(4-(trifluoromethyl)phenyl)-2-(trimethylsilyl)diazene (**1k**).

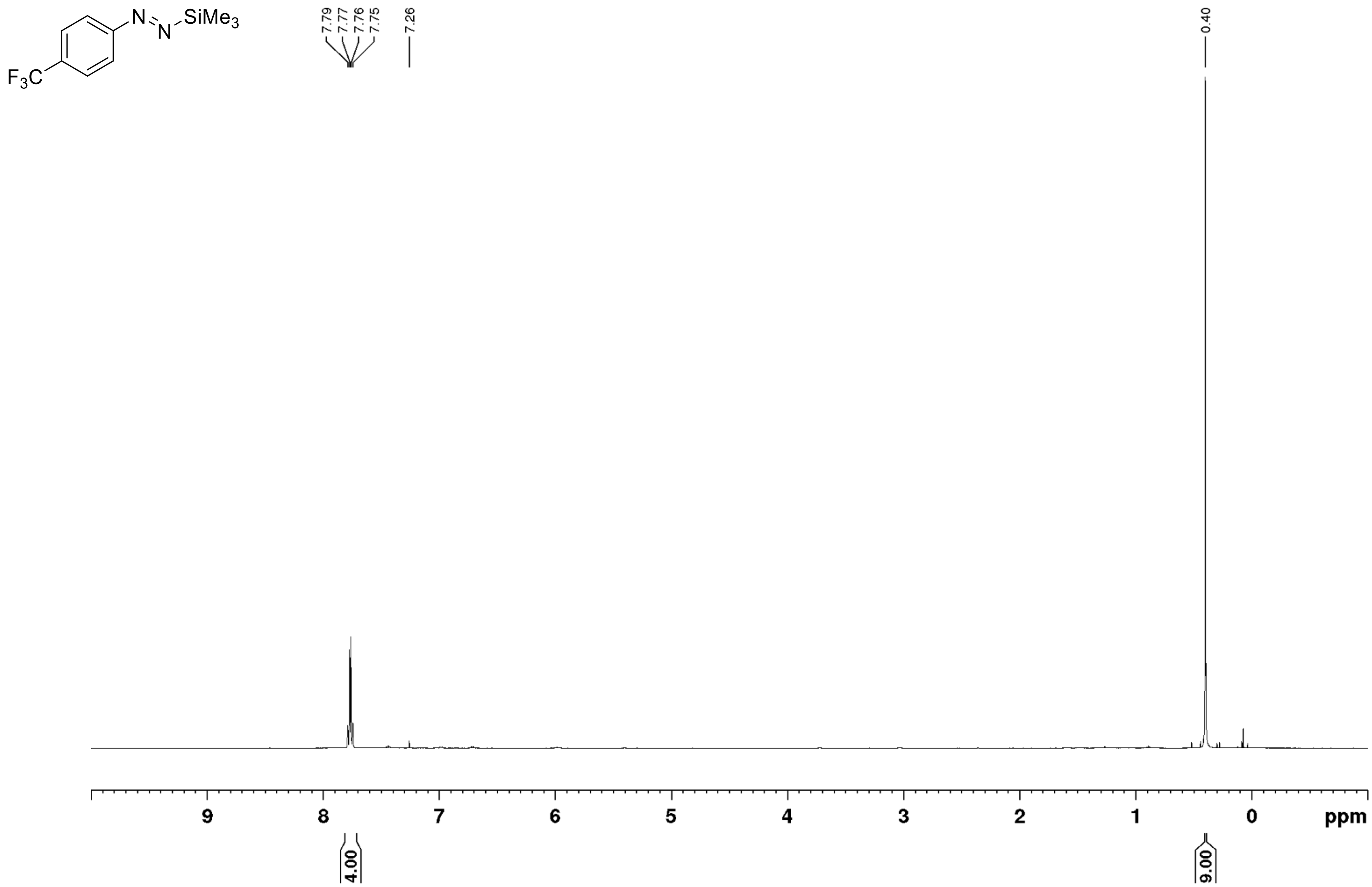

Figure S11. $^{13}C\{^{1}H\}$ NMR spectrum (126 MHz, $CDCl_3$) of (*E*)-1-(4-(trifluoromethyl)phenyl)-2-(trimethylsilyl)diazene (**1k**).

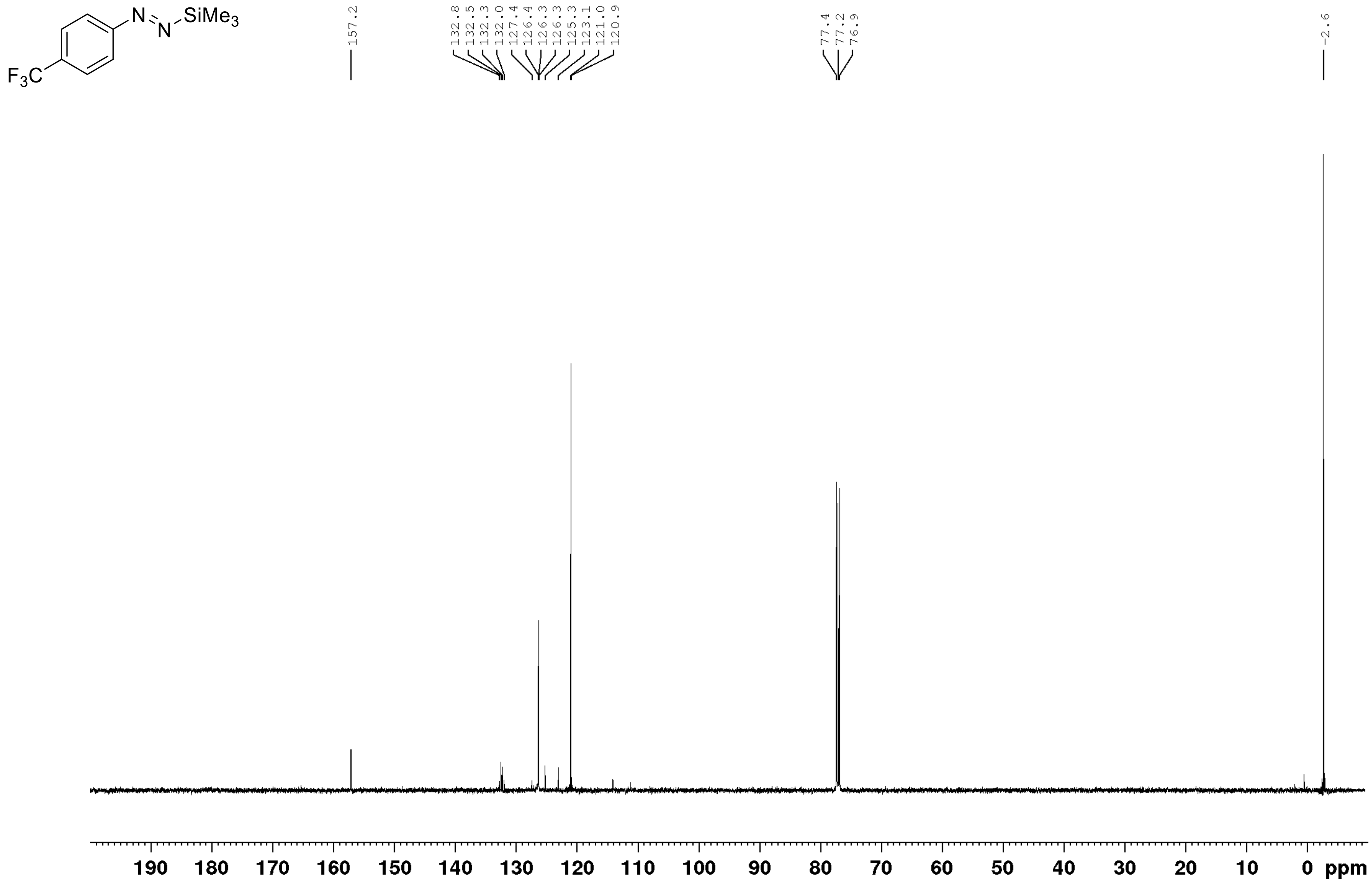

Figure S12. ^{19}F NMR spectrum (471 MHz, $CDCl_3$) of (*E*)-1-(4-(trifluoromethyl)phenyl)-2-(trimethylsilyl)diazene (**1k**).

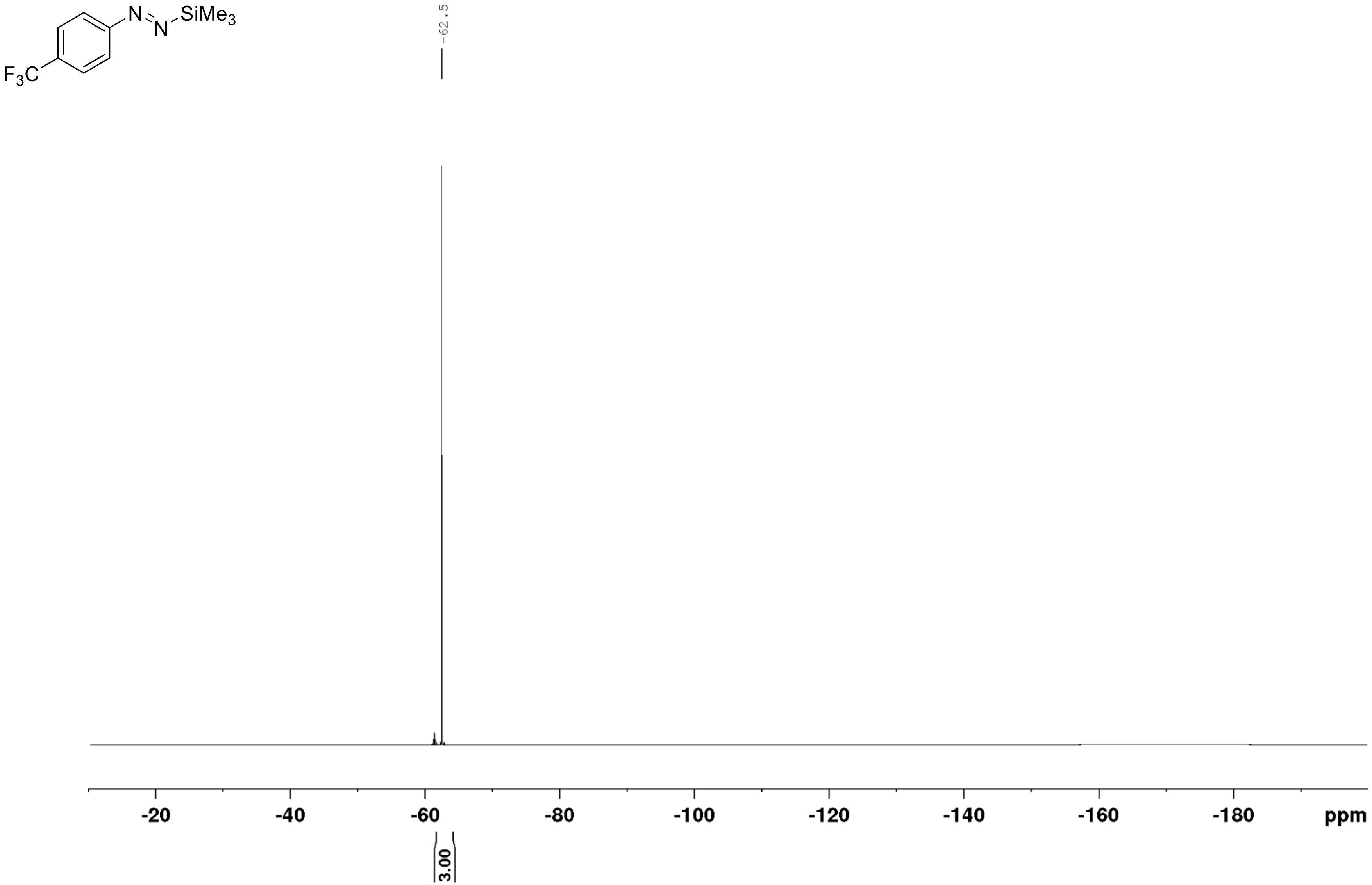

Figure S13. ^1H NMR spectrum (400 MHz, $CDCl_3$) of di-*tert*-butyl 1-(4-fluoro-2,6-dimethylphenyl)hydrazine-1,2-dicarboxylate (**S4**).

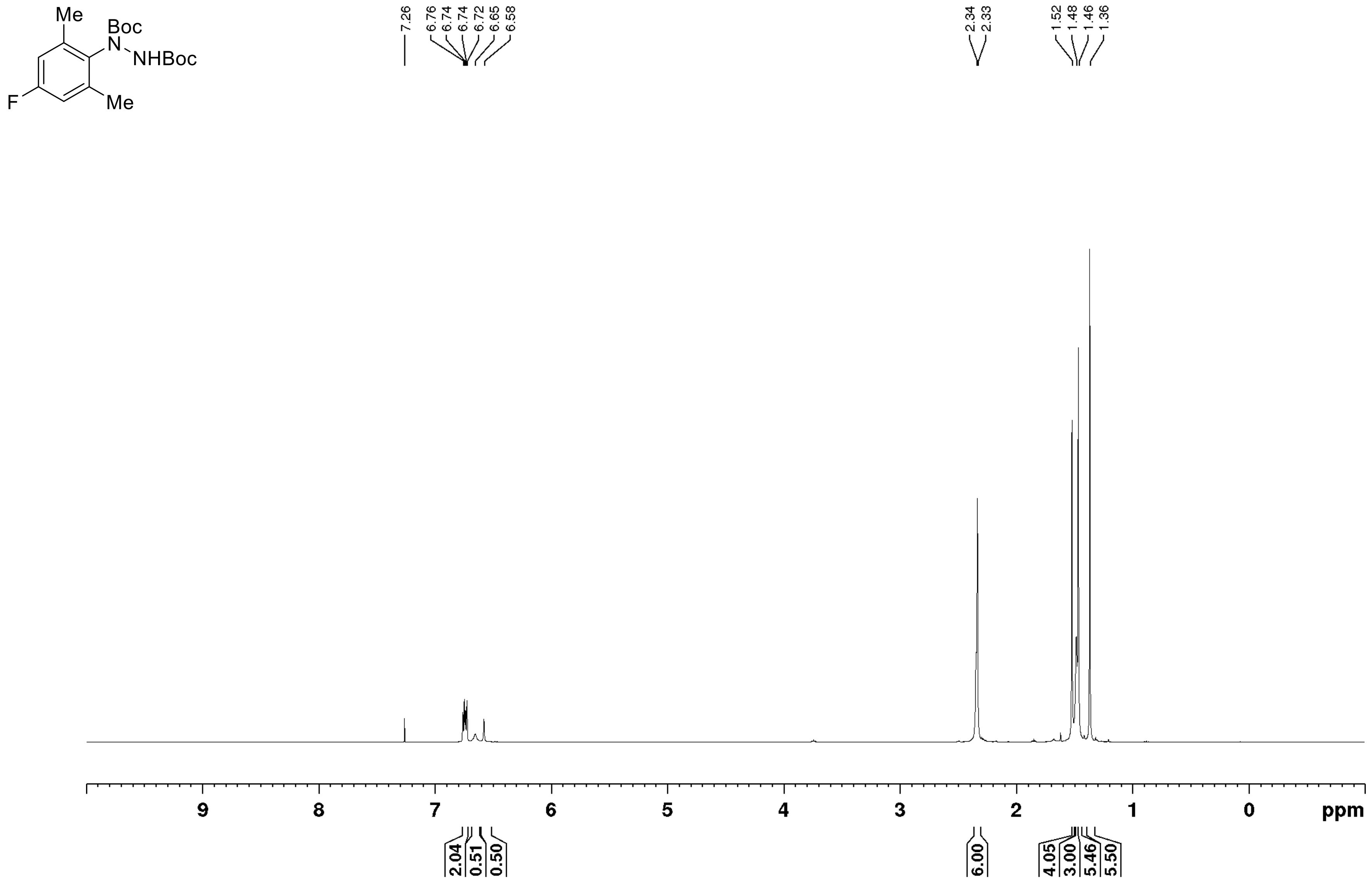

Figure S14. $^{13}C\{^{1}H\}$ NMR spectrum (101 MHz, $CDCl_3$) of di-*tert*-butyl 1-(4-fluoro-2,6-dimethylphenyl)hydrazine-1,2-dicarboxylate (**S4**).

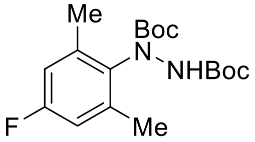

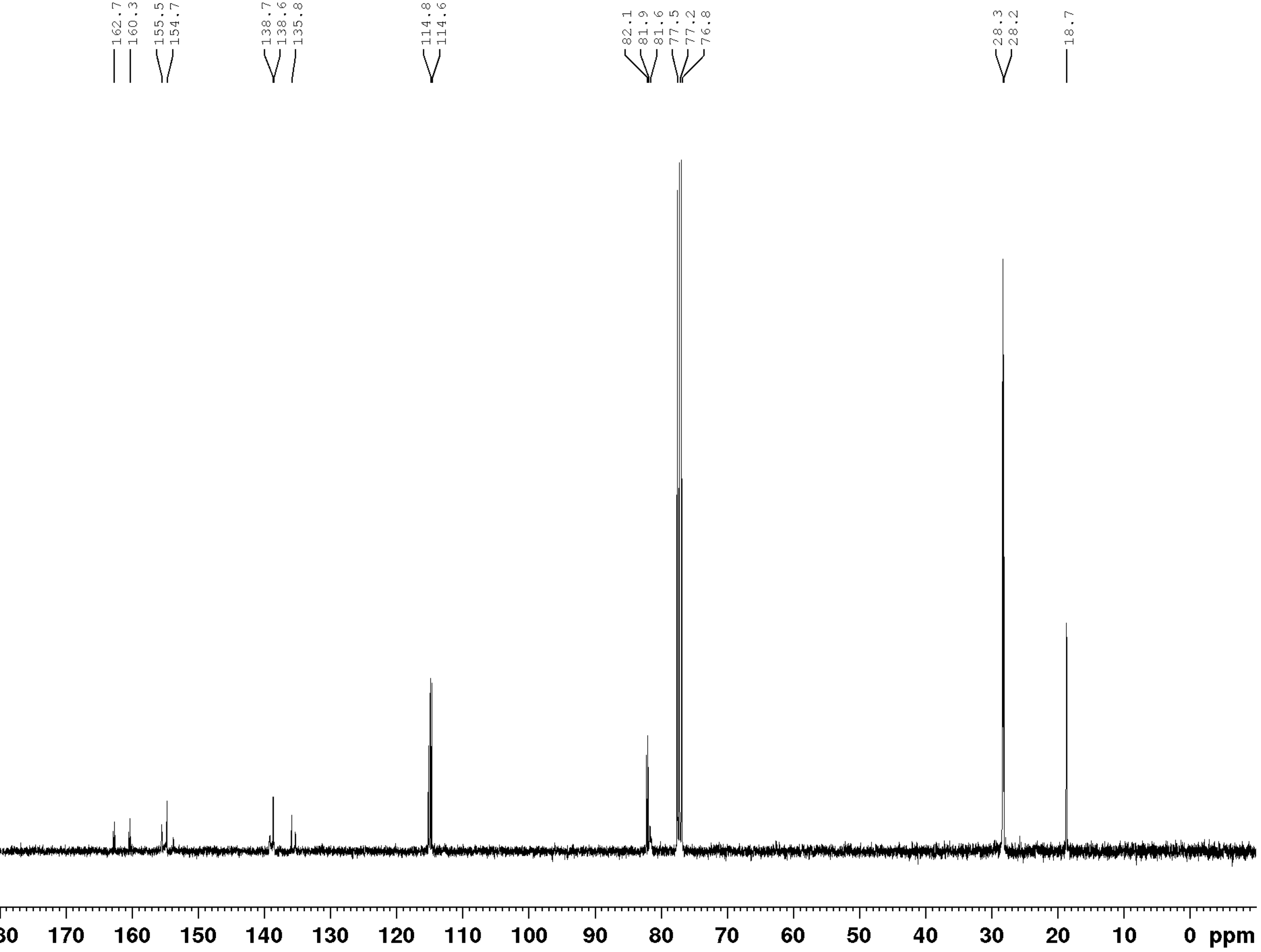

Figure S15. $^{19}F\{^{1}H\}$ NMR spectrum (471 MHz, $CDCl_3$) of di-*tert*-butyl 1-(4-fluoro-2,6-dimethylphenyl)hydrazine-1,2-dicarboxylate (**S4**).

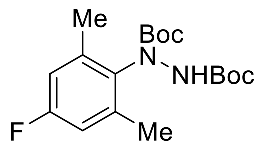

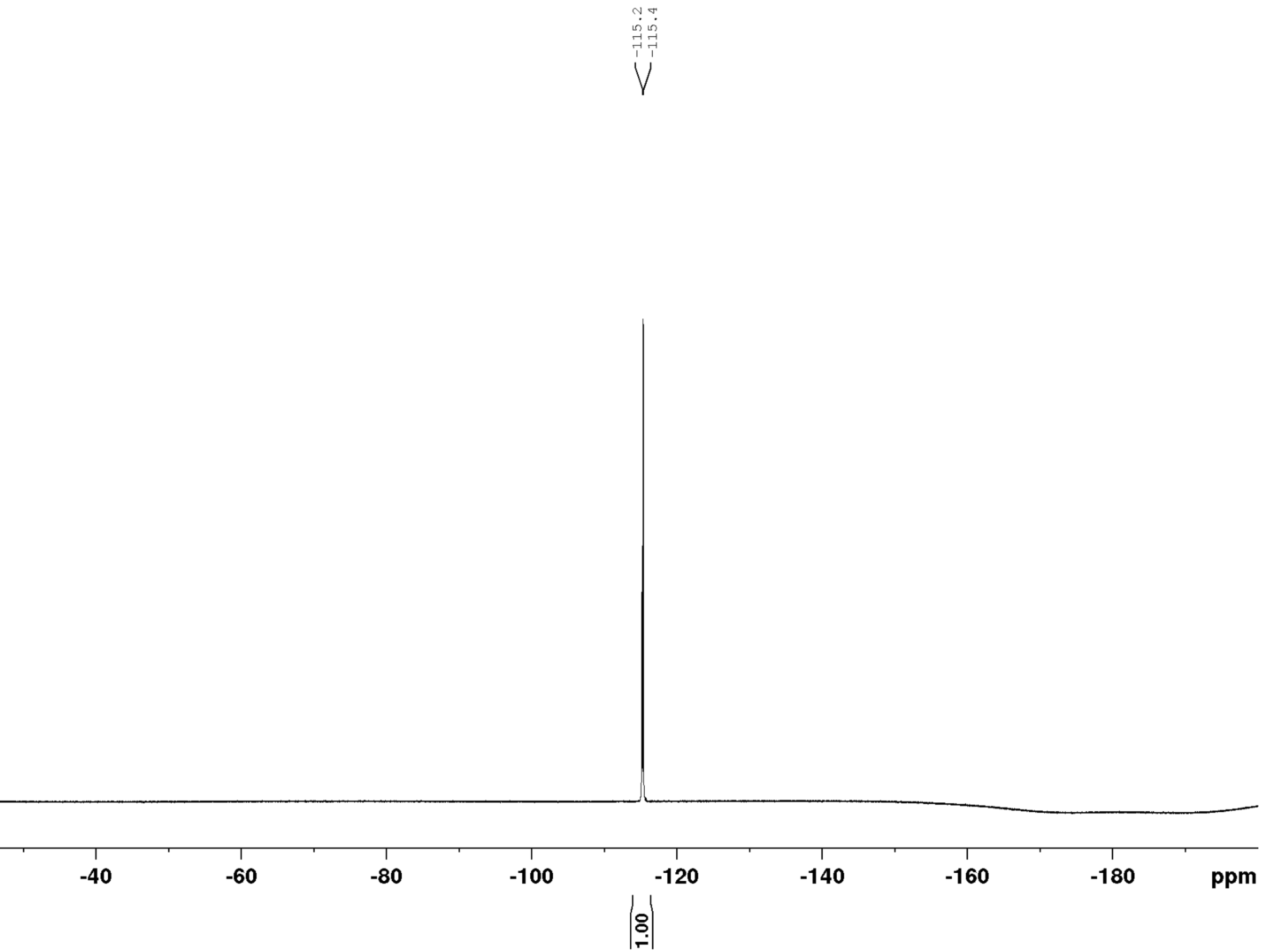

Figure S16. ^{1}H NMR spectrum (500 MHz, $(CD_3)_2SO$) of 2-(4-fluoro-2,6-dimethylphenyl)hydrazin-1-ium chloride (**S5**).

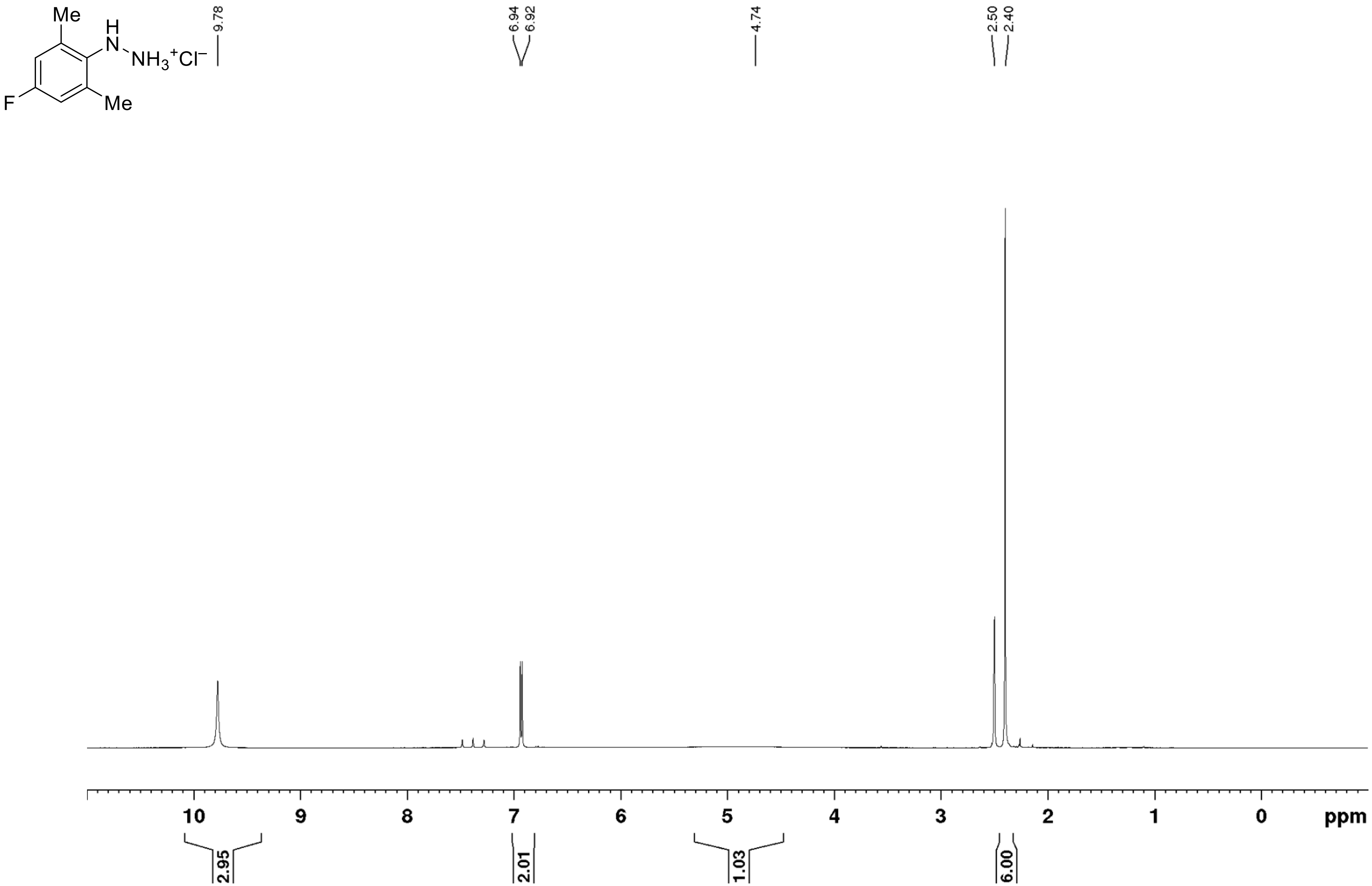

Figure S17. $^{13}C\{^1H\}$ NMR spectrum (126 MHz, $(CD_3)_2SO$) of 2-(4-fluoro-2,6-dimethylphenyl)hydrazin-1-ium chloride (**S5**).

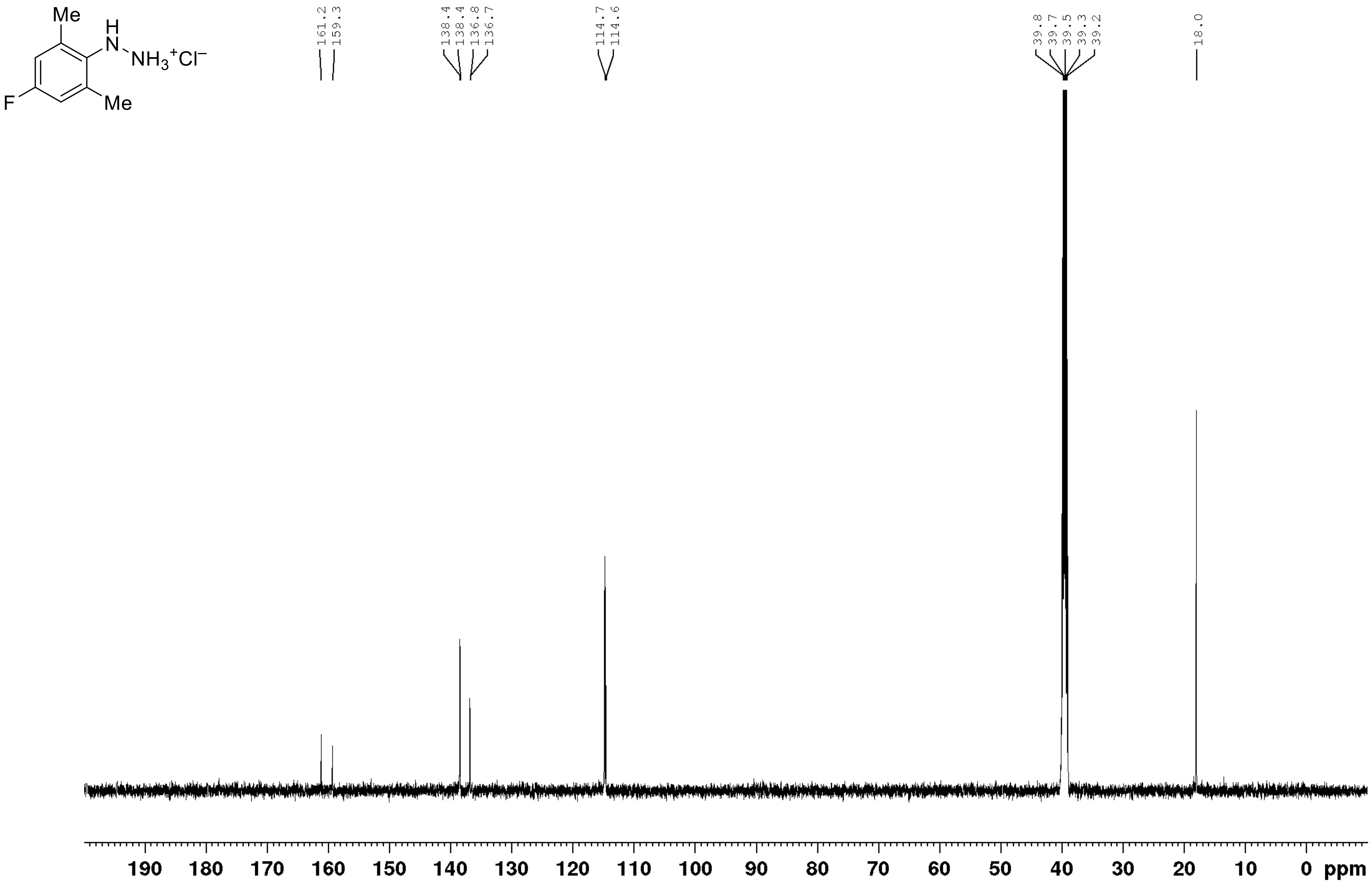

Figure S18. $^{19}F\{^{1}H\}$ NMR spectrum (471 MHz, $(CD_3)_2SO$) of 2-(4-fluoro-2,6-dimethylphenyl)hydrazin-1-ium chloride (**S5**).

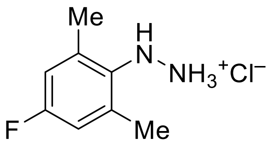

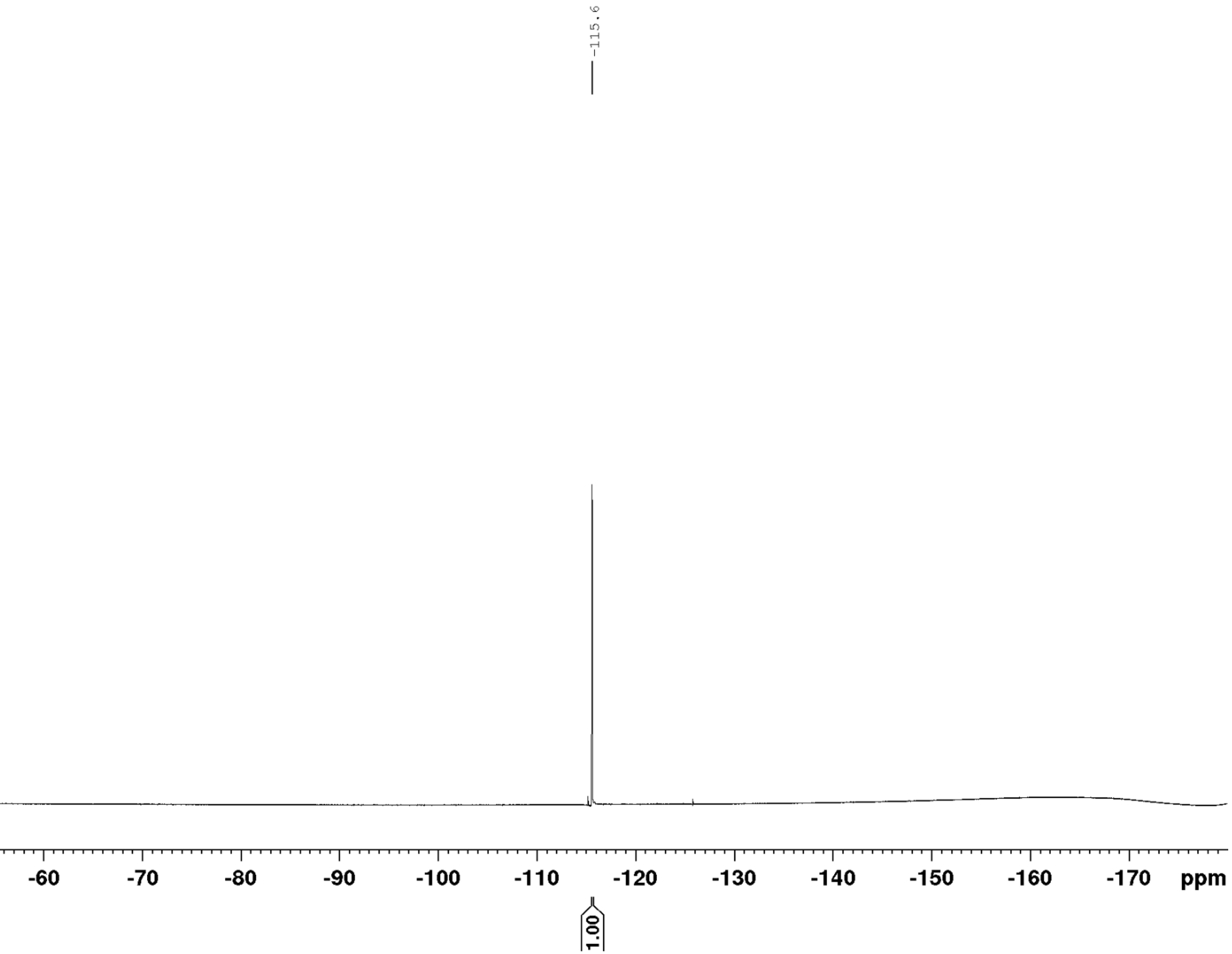

Figure S19. ^{1}H NMR spectrum (500 MHz, $CDCl_3$) of 1-(4-fluoro-2,6-dimethylphenyl)-2-(trimethylsilyl)hydrazine (**S6**).

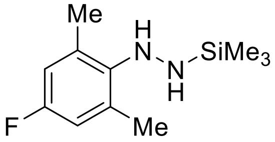

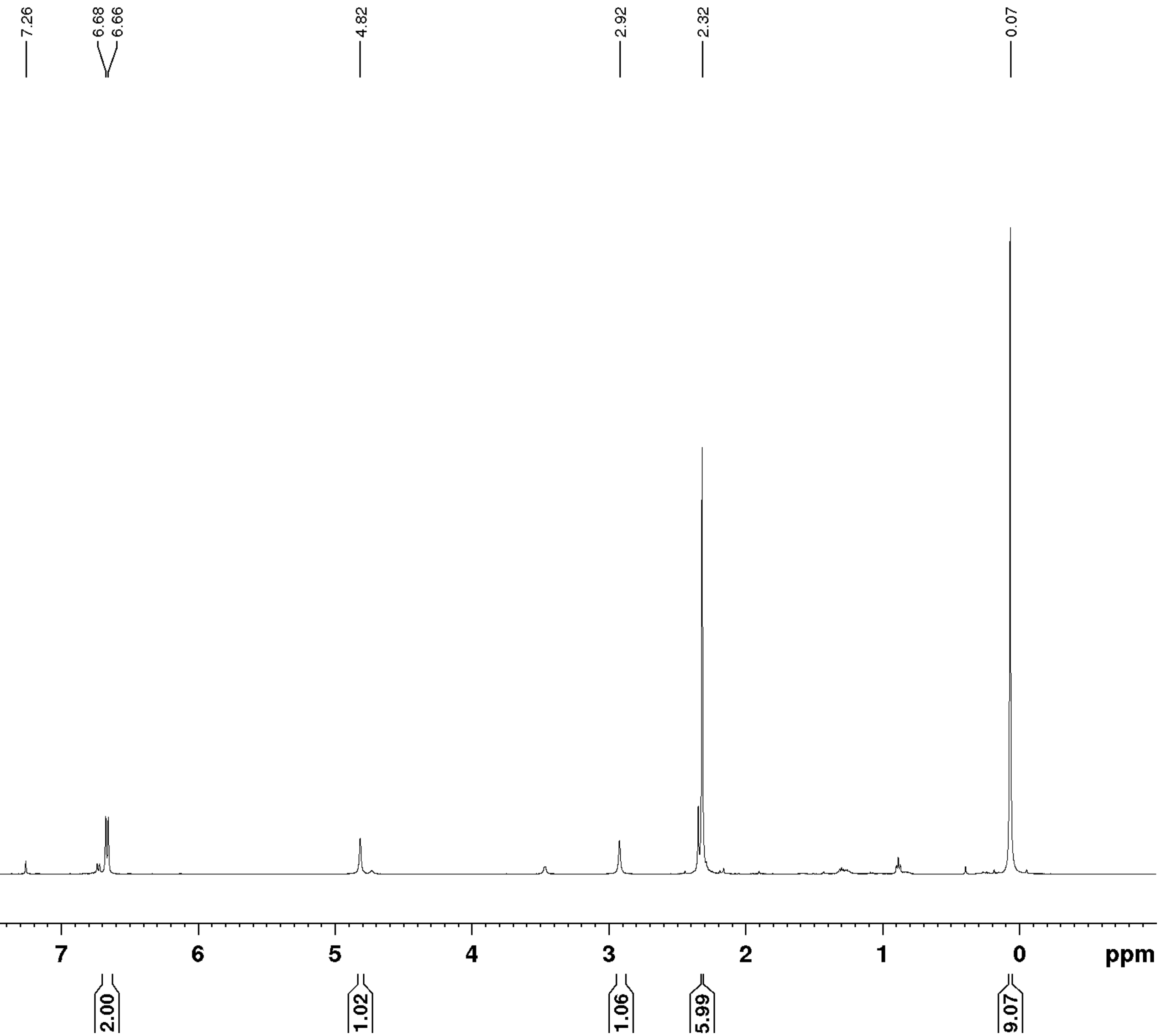

Figure S20. $^{13}C\{^1H\}$ NMR spectrum (126 MHz, $CDCl_3$) of 1-(4-fluoro-2,6-dimethylphenyl)-2-(trimethylsilyl)hydrazine (**S6**).

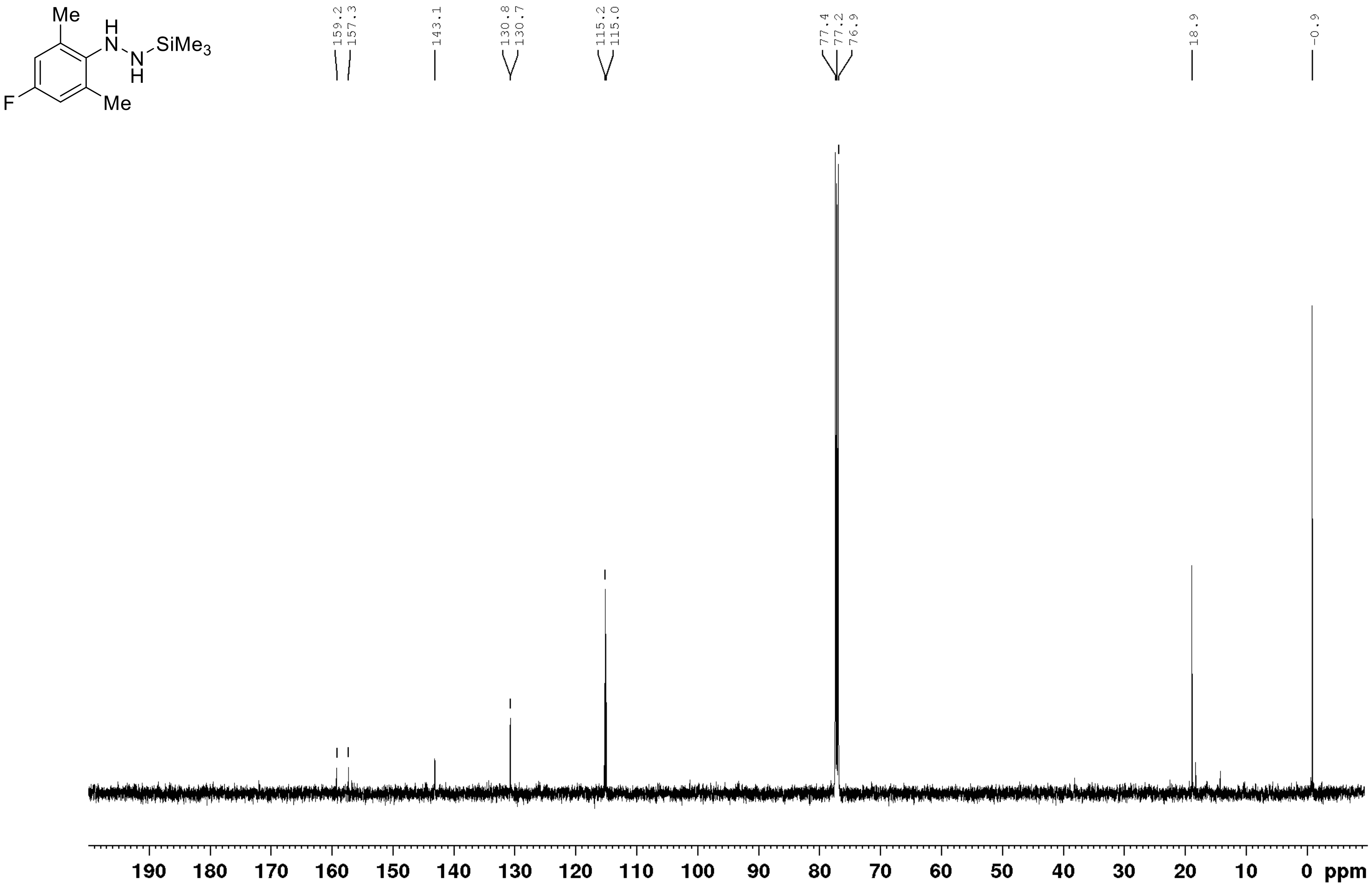

Figure S21. $^{19}F\{^1H\}$ NMR spectrum (471 MHz, $CDCl_3$) of 1-(4-fluoro-2,6-dimethylphenyl)-2-(trimethylsilyl)hydrazine (**S6**).

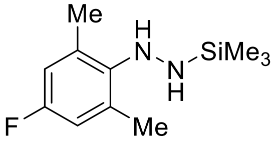

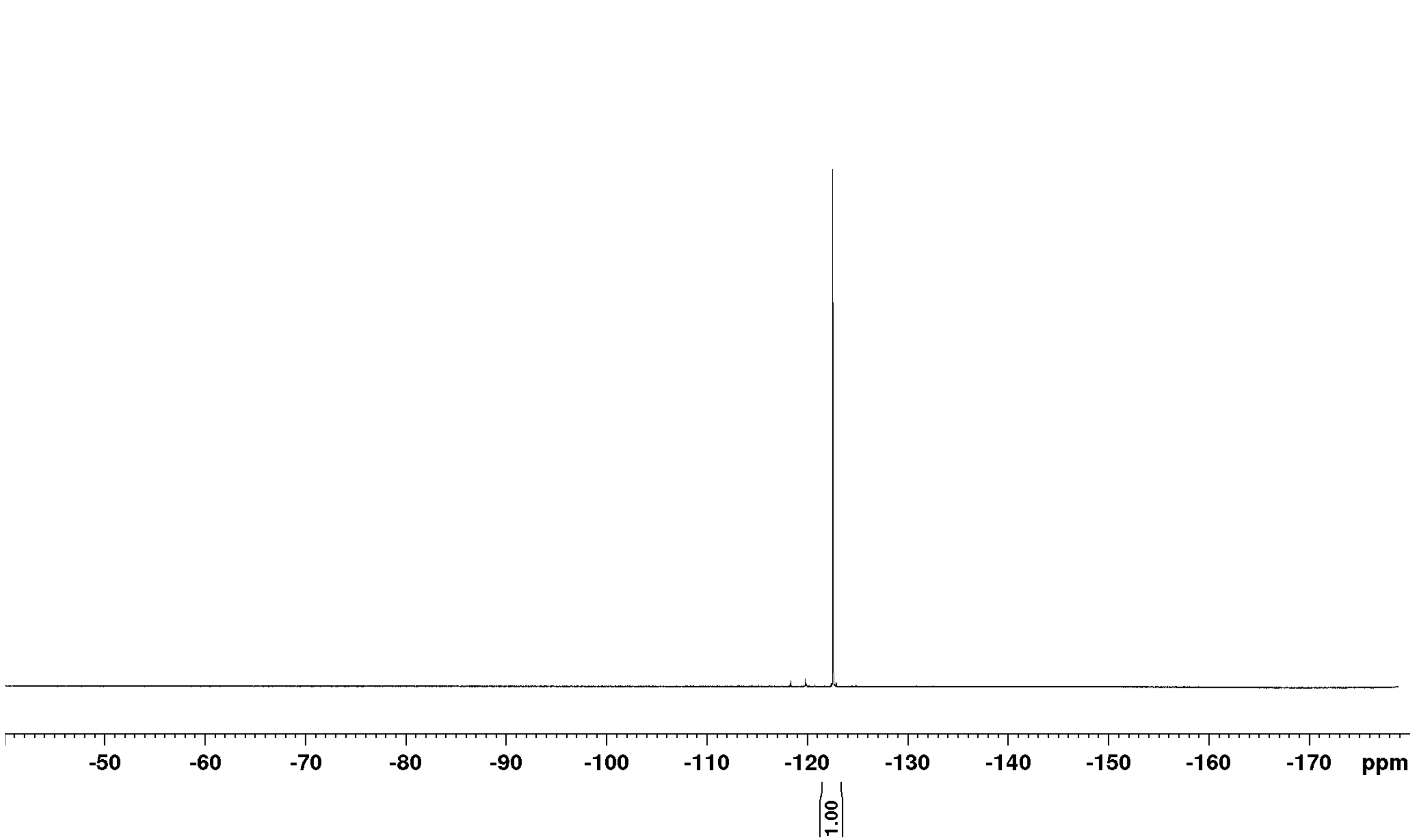

Figure S22. ^{1}H NMR spectrum (500 MHz, $CDCl_3$) of (*E*)-1-(4-fluoro-2,6-dimethylphenyl)-2-(trimethylsilyl)diazene (**1m**).

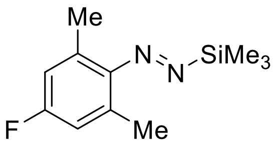

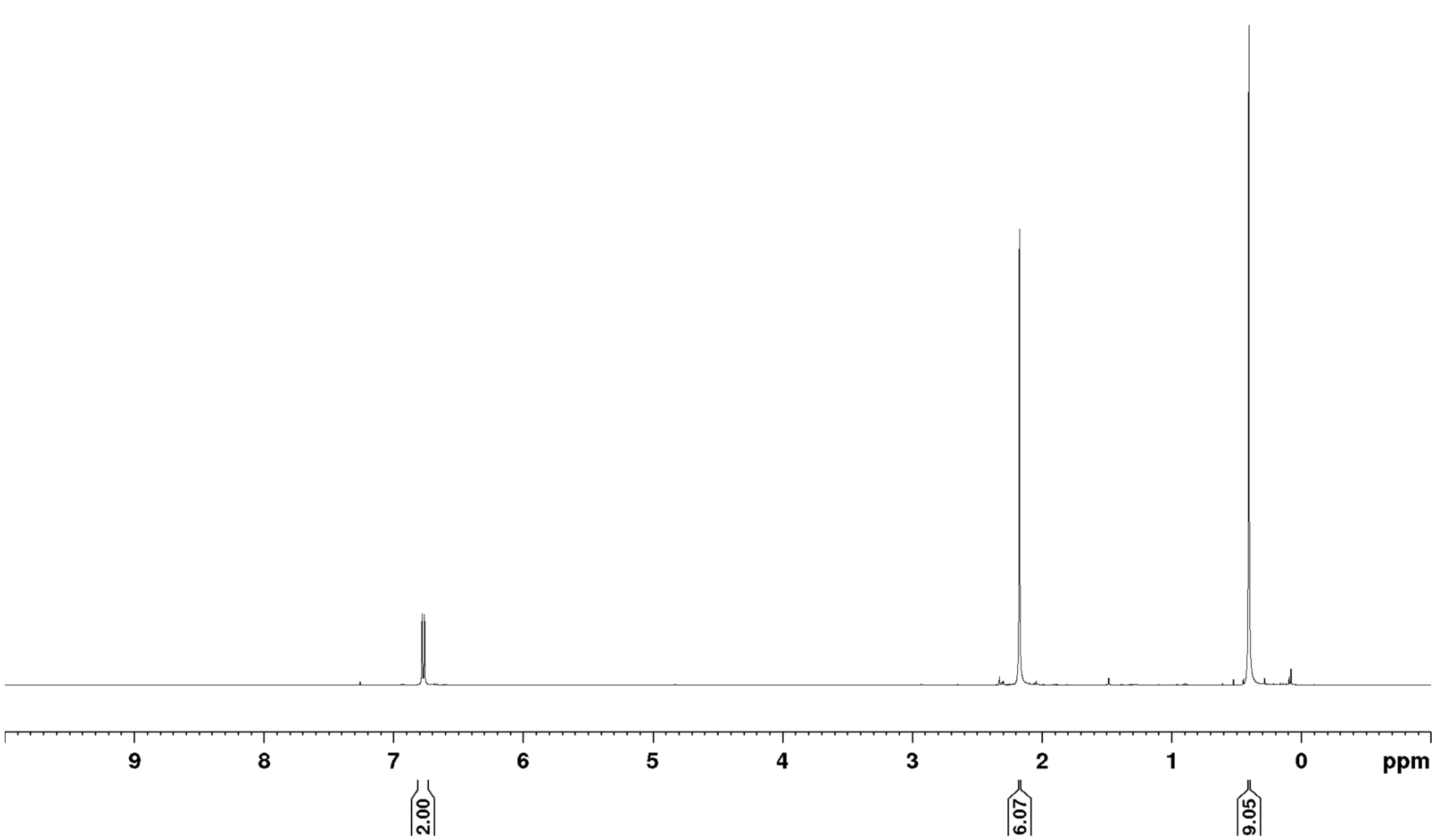

Figure S23. $^{13}C\{^{1}H\}$ NMR spectrum (126 MHz, $CDCl_3$) of (*E*)-1-(4-fluoro-2,6-dimethylphenyl)-2-(trimethylsilyl)diazene (**1m**).

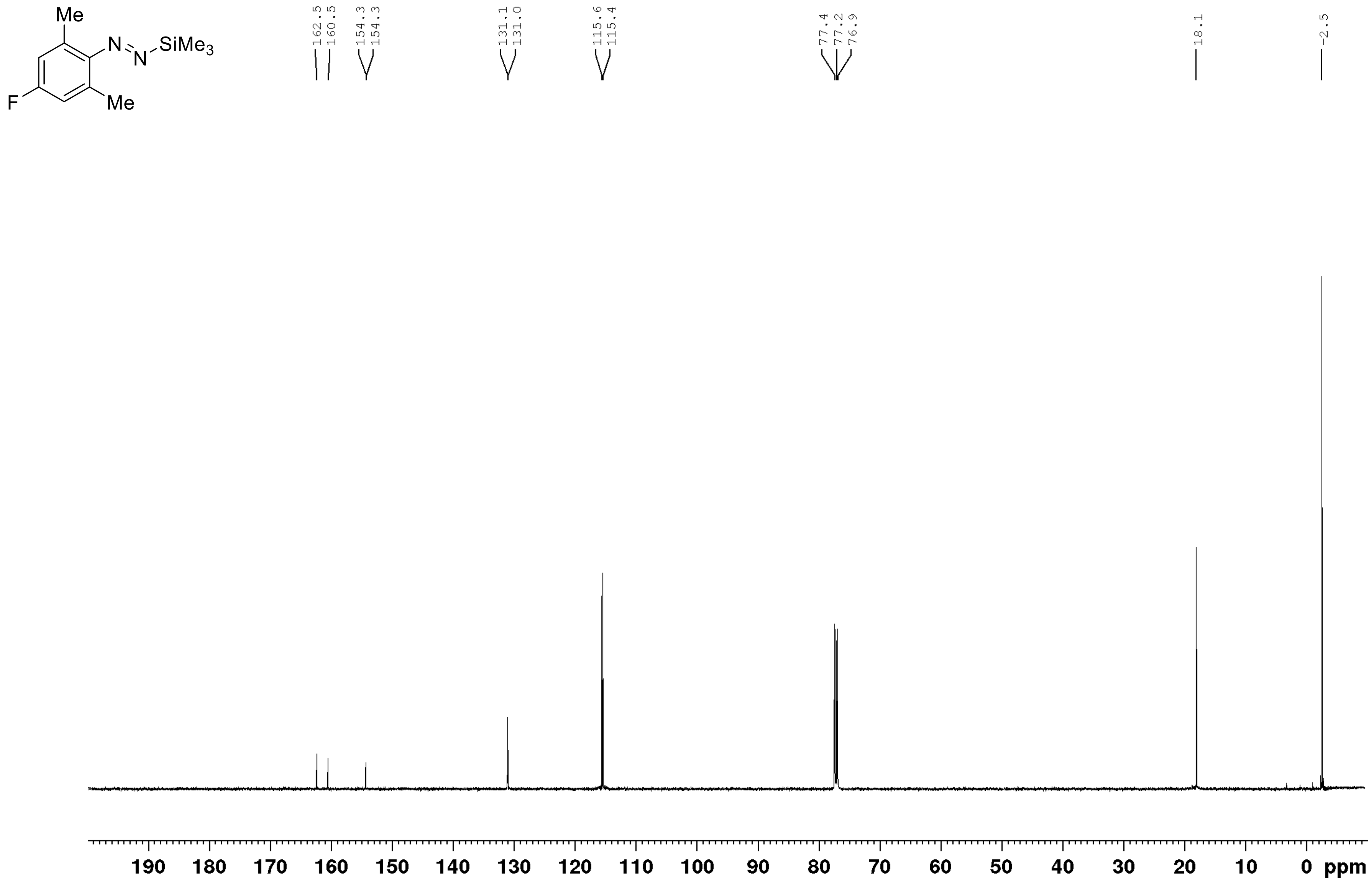

Figure S24. ^{19}F NMR spectrum (471 MHz, $CDCl_3$) of (*E*)-1-(4-fluoro-2,6-dimethylphenyl)-2-(trimethylsilyl)diazene (**1m**).

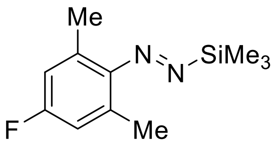

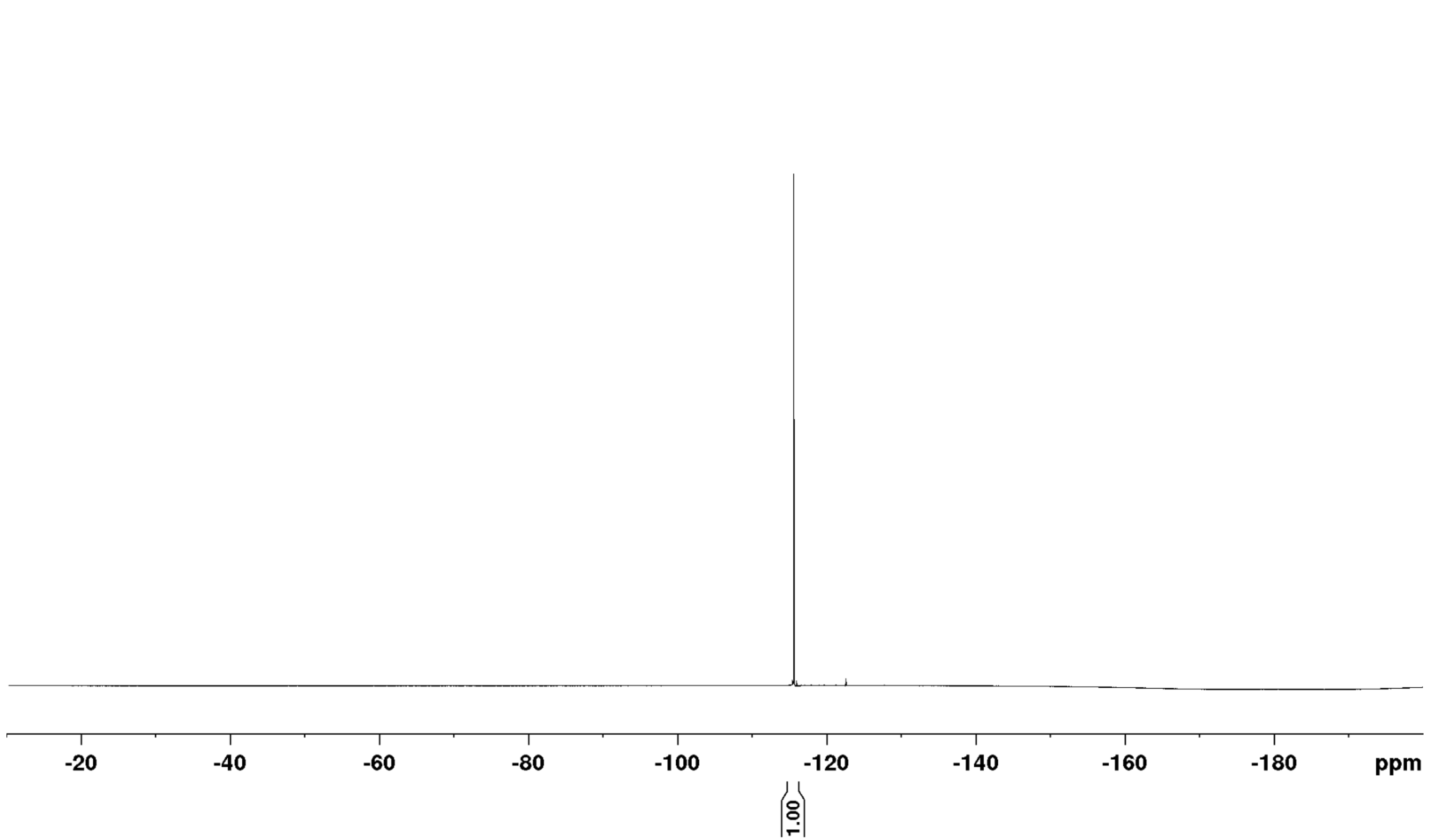

9.2 Polyfluorinated Biaryls

Figure S25. ^{1}H NMR spectrum (500 MHz, $CDCl_3$) of 2,3,4,5,6-pentafluoro-4'-methyl-1,1'-biphenyl (**3aa**).

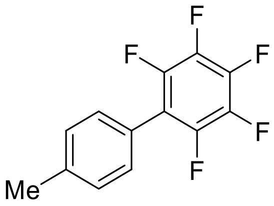

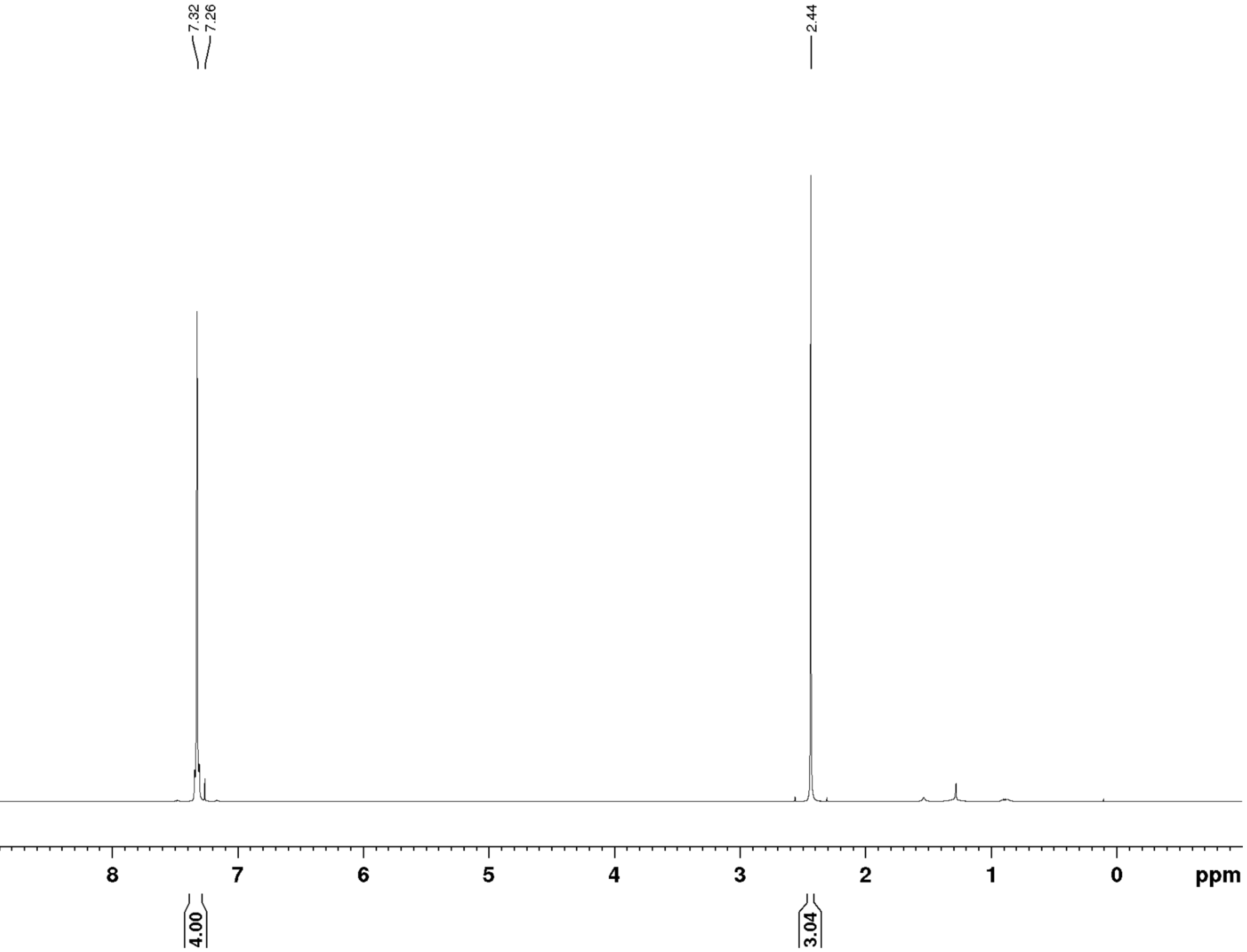

Figure S26. $^{13}C\{^1H\}$ NMR spectrum (126 MHz, $CDCl_3$) of 2,3,4,5,6-pentafluoro-4'-methyl-1,1'-biphenyl (**3aa**).

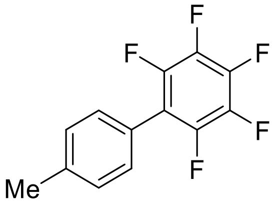

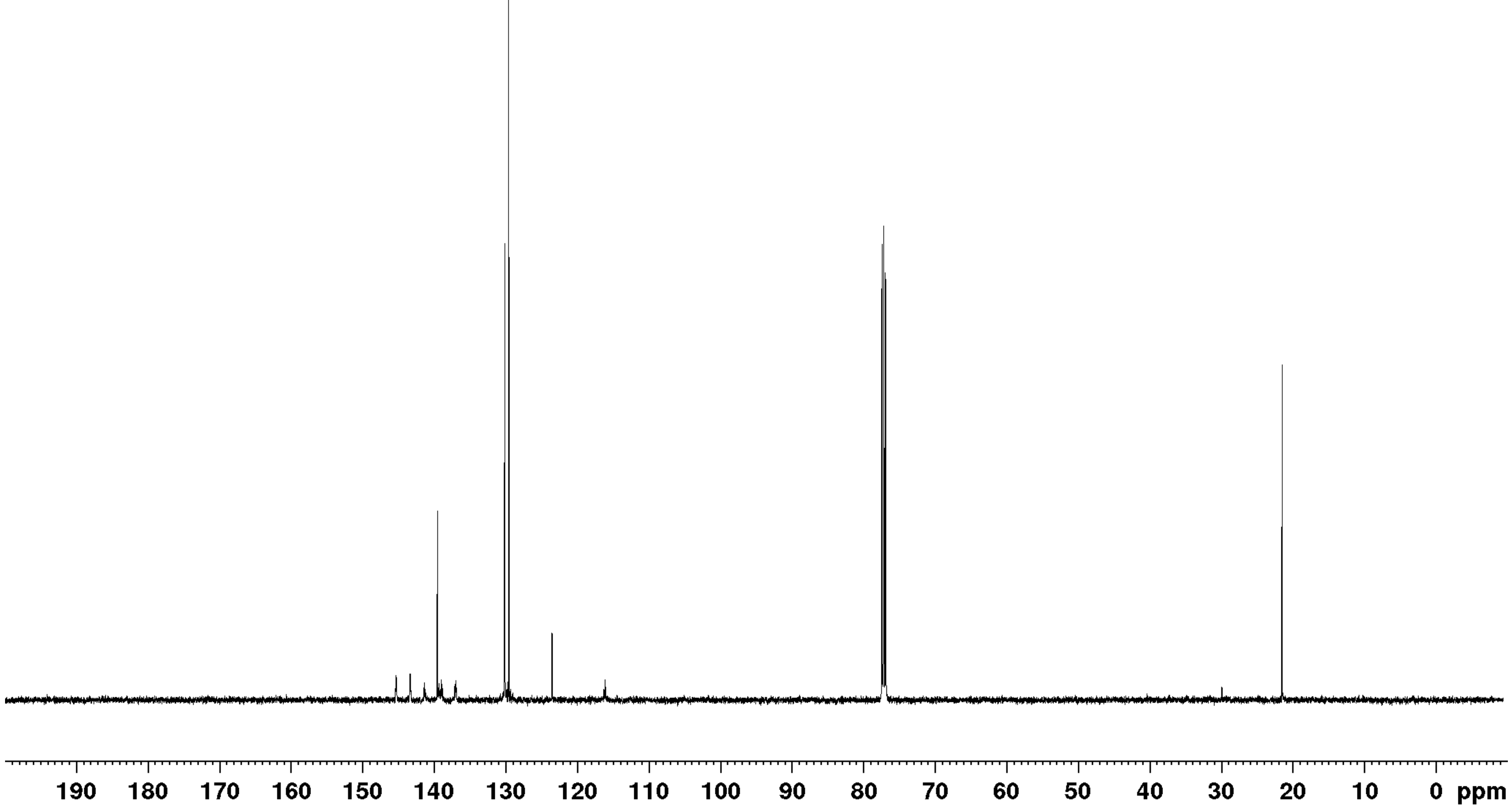

Figure S27. ^{19}F NMR spectrum (471 MHz, $CDCl_3$) of 2,3,4,5,6-pentafluoro-4'-methyl-1,1'-biphenyl (**3aa**).

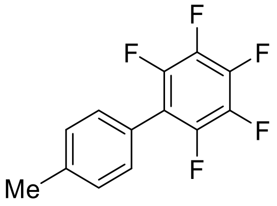

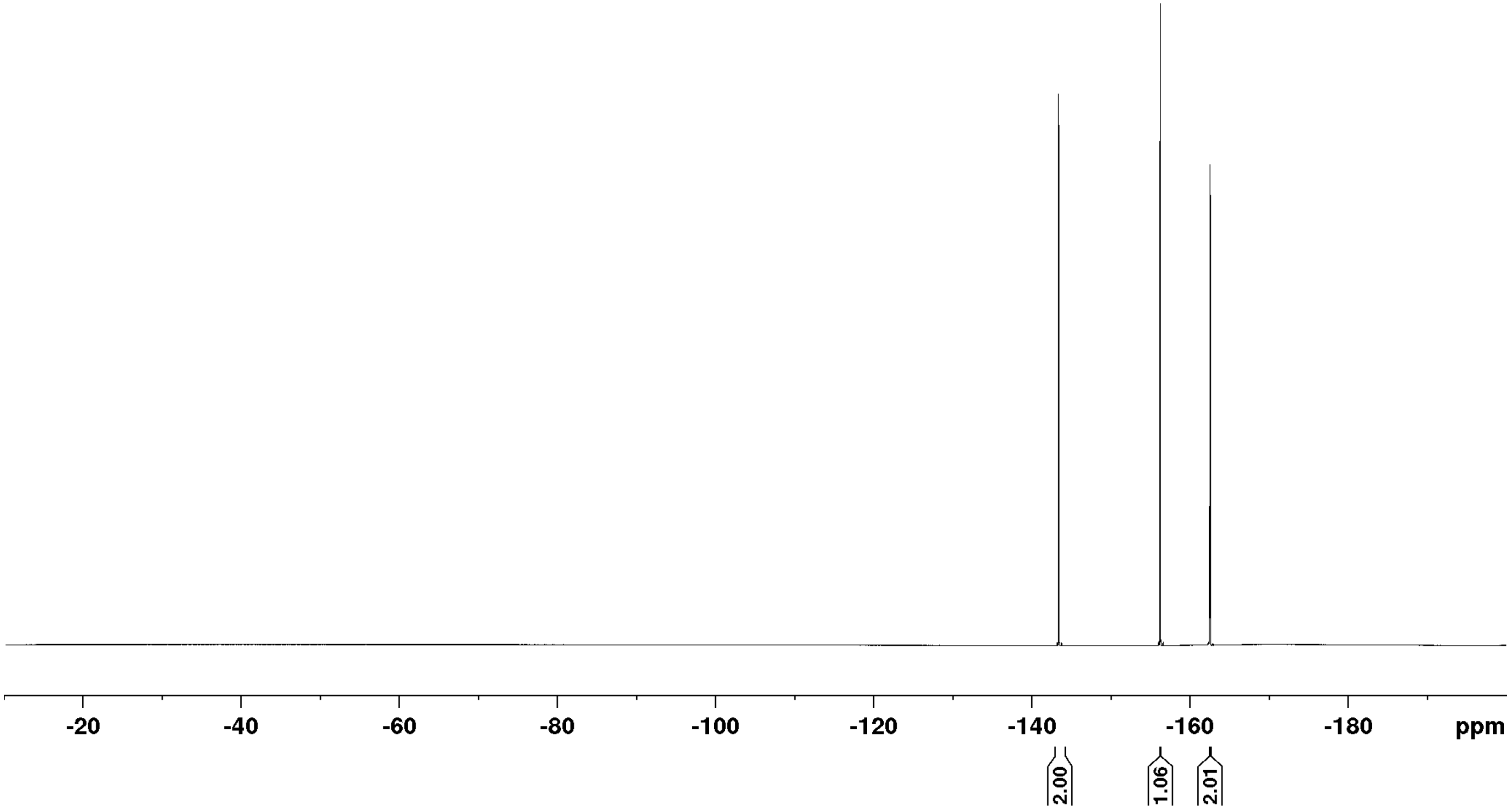

Figure S28. ^{1}H NMR spectrum (500 MHz, $CDCl_3$) of 2,3,4,5,6-pentafluoro-1,1'-biphenyl (**3ba**).

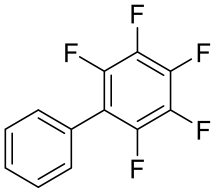

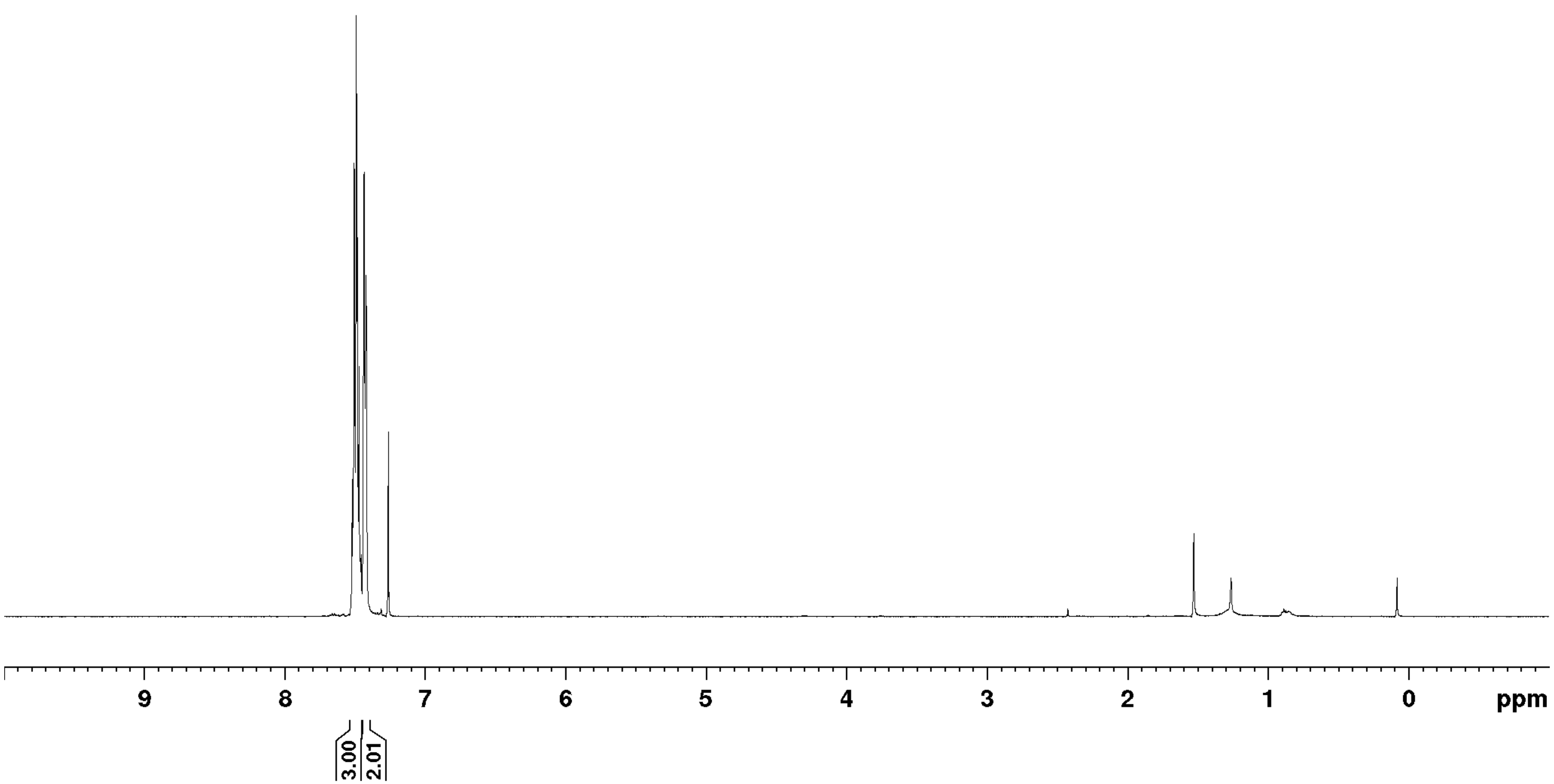

Figure S29. $^{13}C\{^{1}H\}$ NMR spectrum (101 MHz, $CDCl_3$) of 2,3,4,5,6-pentafluoro-1,1'-biphenyl (**3ba**).

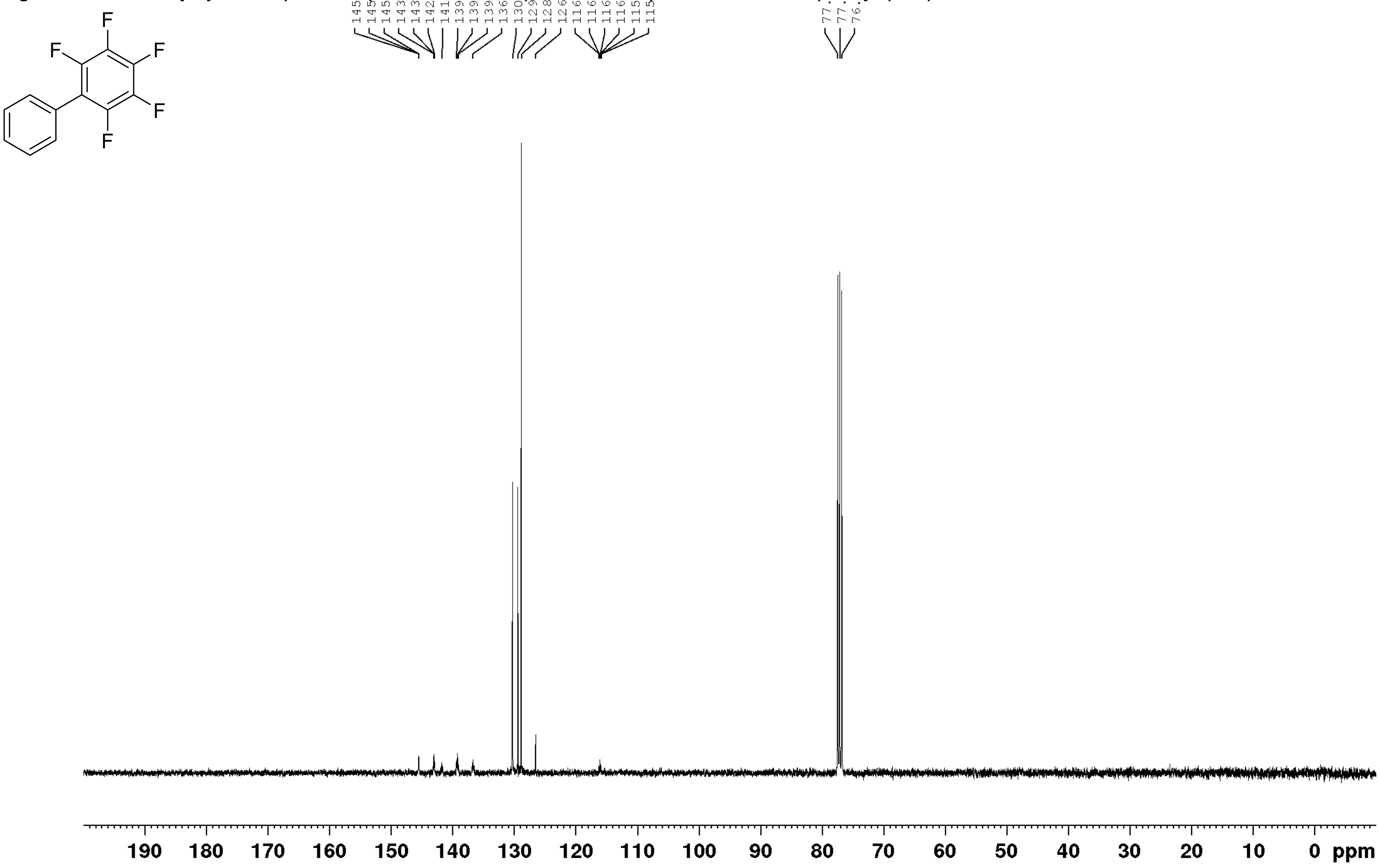

Figure S30. ^{19}F NMR spectrum (471 MHz, $CDCl_3$) of 2,3,4,5,6-pentafluoro-1,1'-biphenyl (**3ba**).

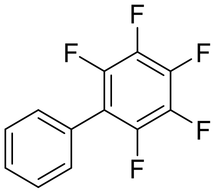

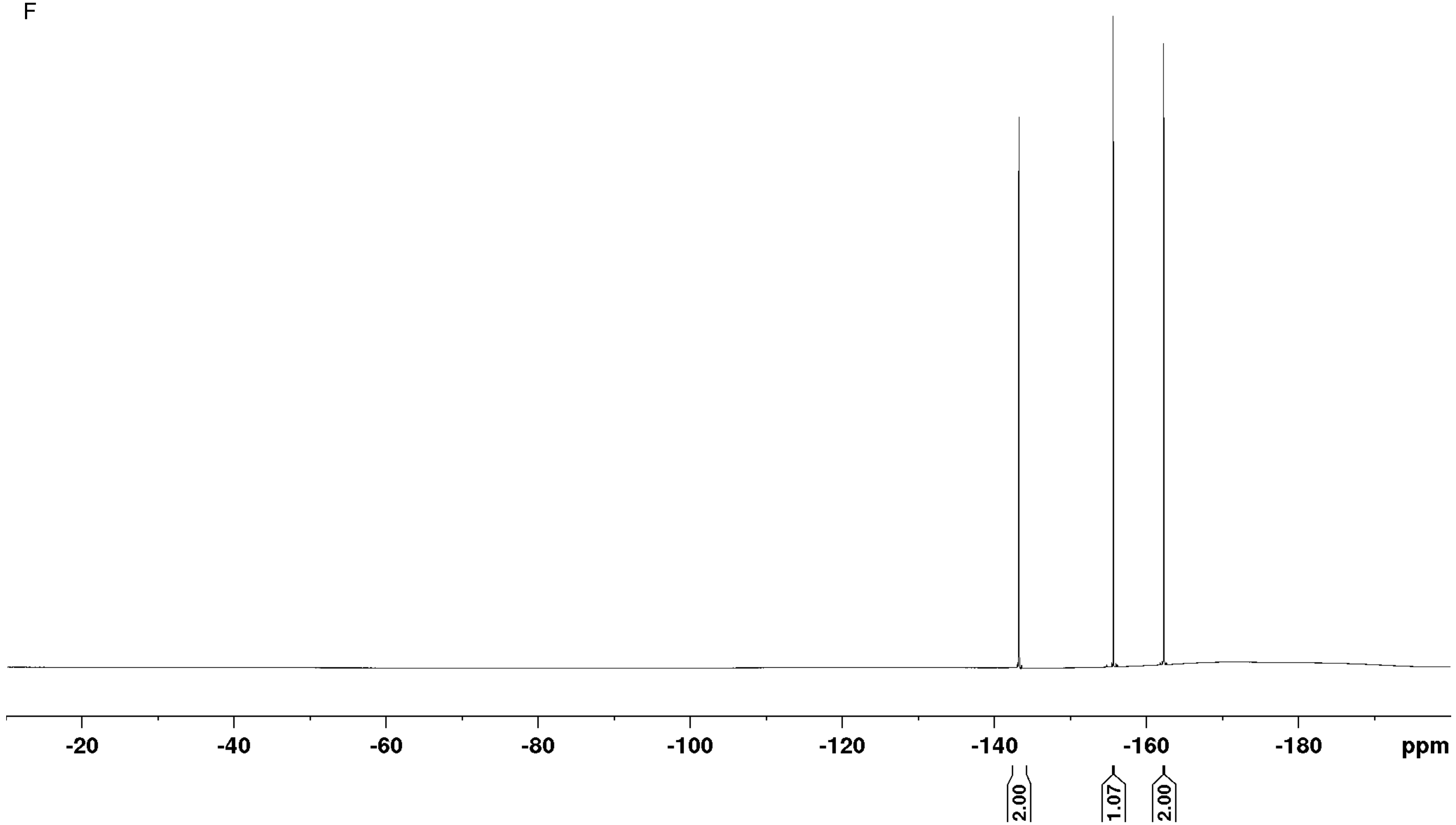

Figure S31. ^{1}H NMR spectrum (500 MHz, $CDCl_3$) of 2,3,4,5,6-pentafluoro-4'-methoxy-1,1'-biphenyl (**3ca**).

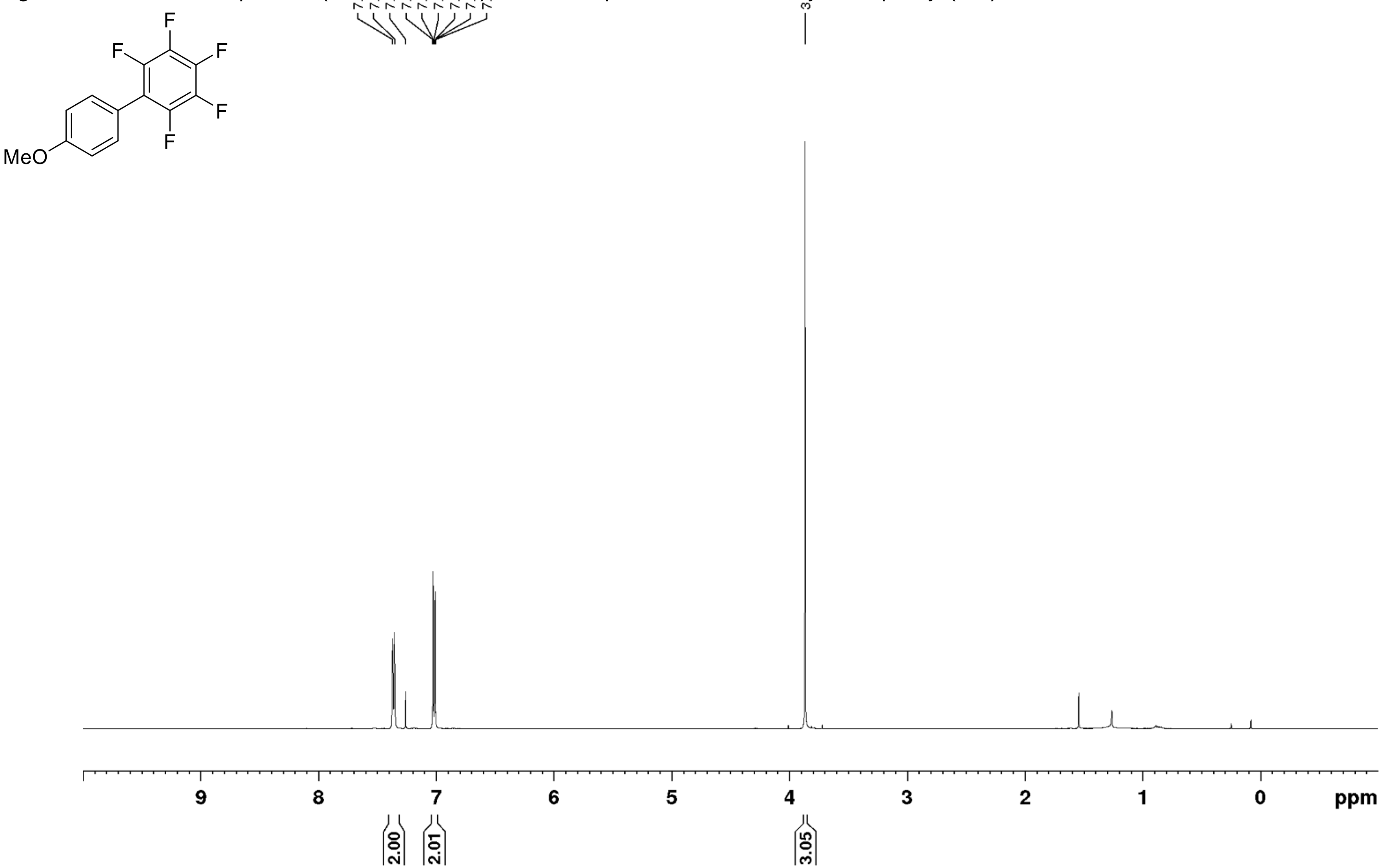

Figure S32. $^{13}C\{^{1}H\}$ NMR spectrum (101 MHz, $CDCl_3$) of 2,3,4,5,6-pentafluoro-4'-methoxy-1,1'-biphenyl (**3ca**).

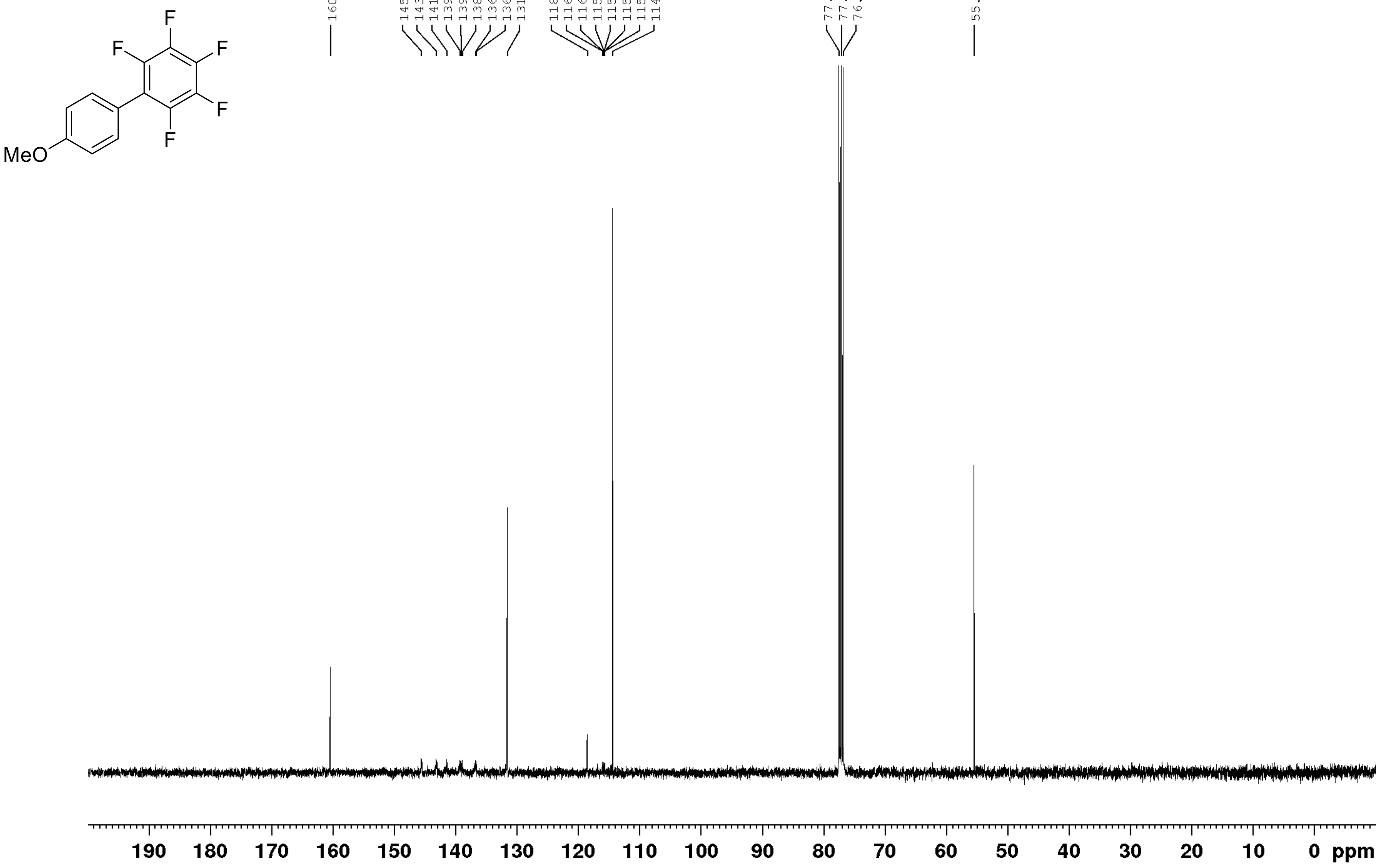

Figure S33. ^{19}F NMR spectrum (471 MHz, $CDCl_3$) of 2,3,4,5,6-pentafluoro-4'-methoxy-1,1'-biphenyl (**3ca**).

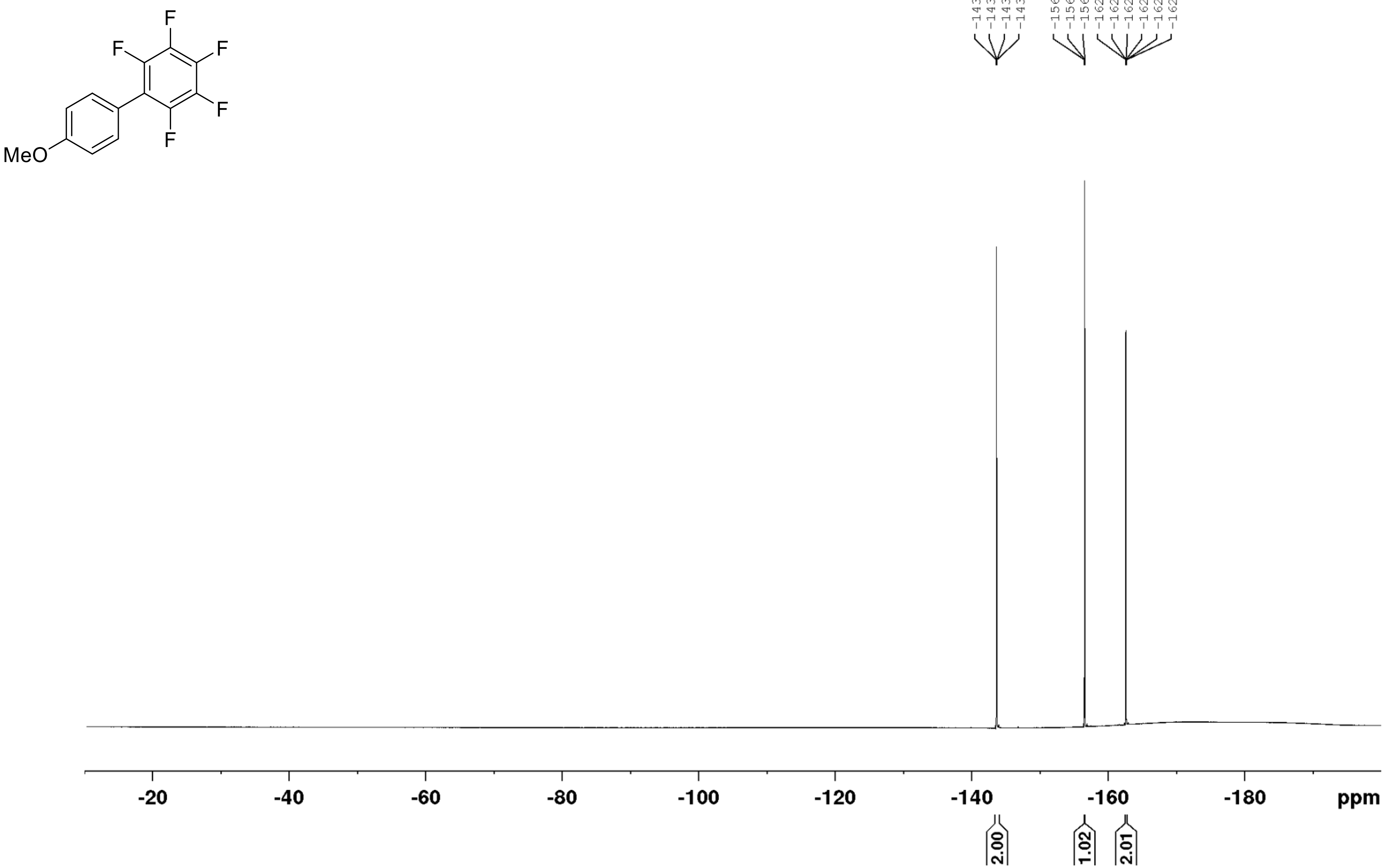

Figure S34. ^{1}H NMR spectrum (400 MHz, $CDCl_3$) of 2',3',4',5',6'-pentafluoro-[1,1'-biphenyl]-4-carbonitrile (**3da**).

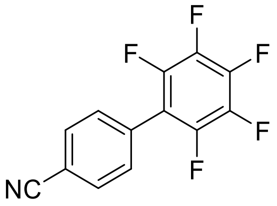

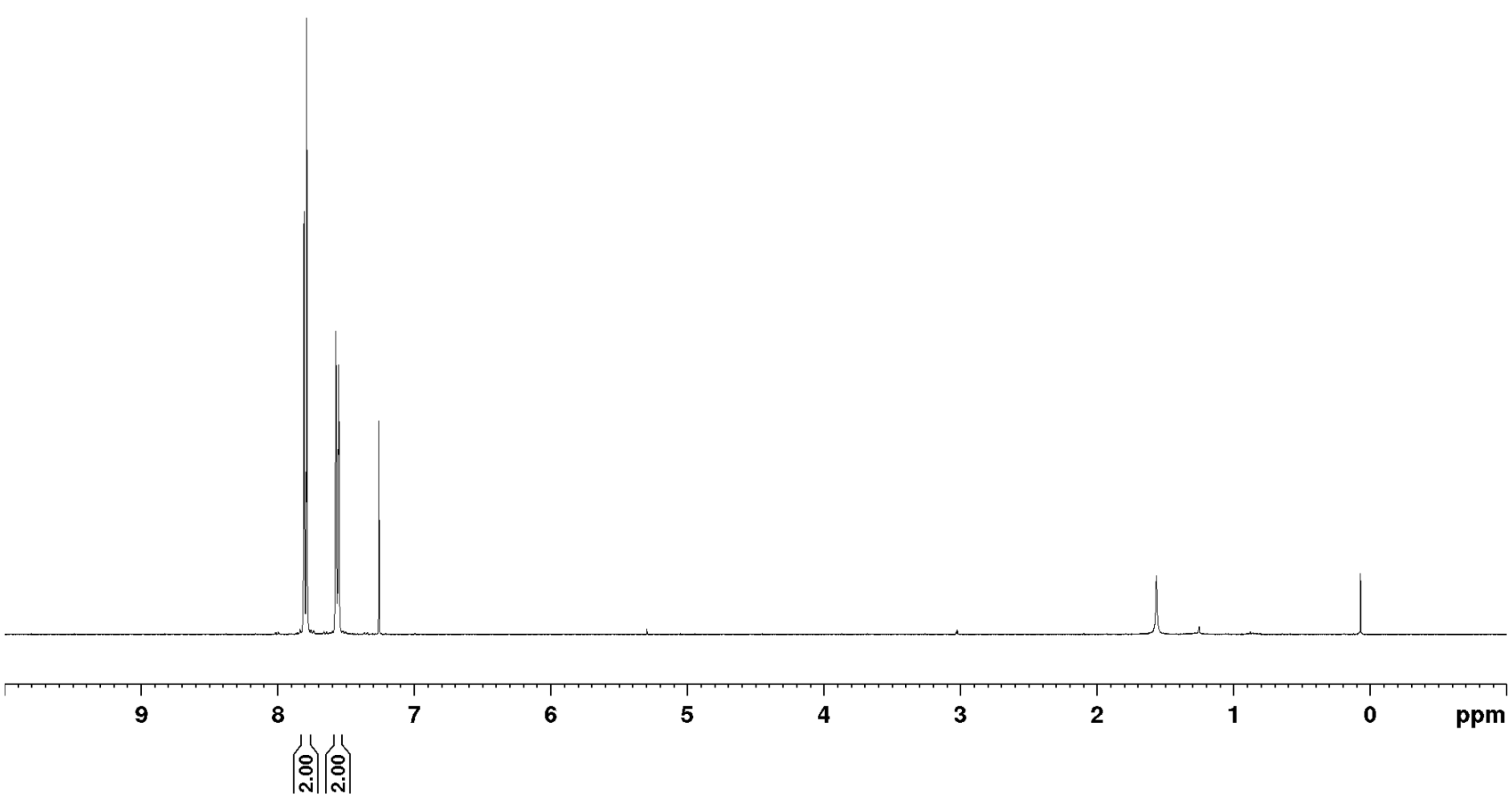

Figure S35. $^{13}C\{^{1}H\}$ NMR spectrum (101 MHz, $CDCl_3$) of 2',3',4',5',6'-pentafluoro-[1,1'-biphenyl]-4-carbonitrile (**3da**).

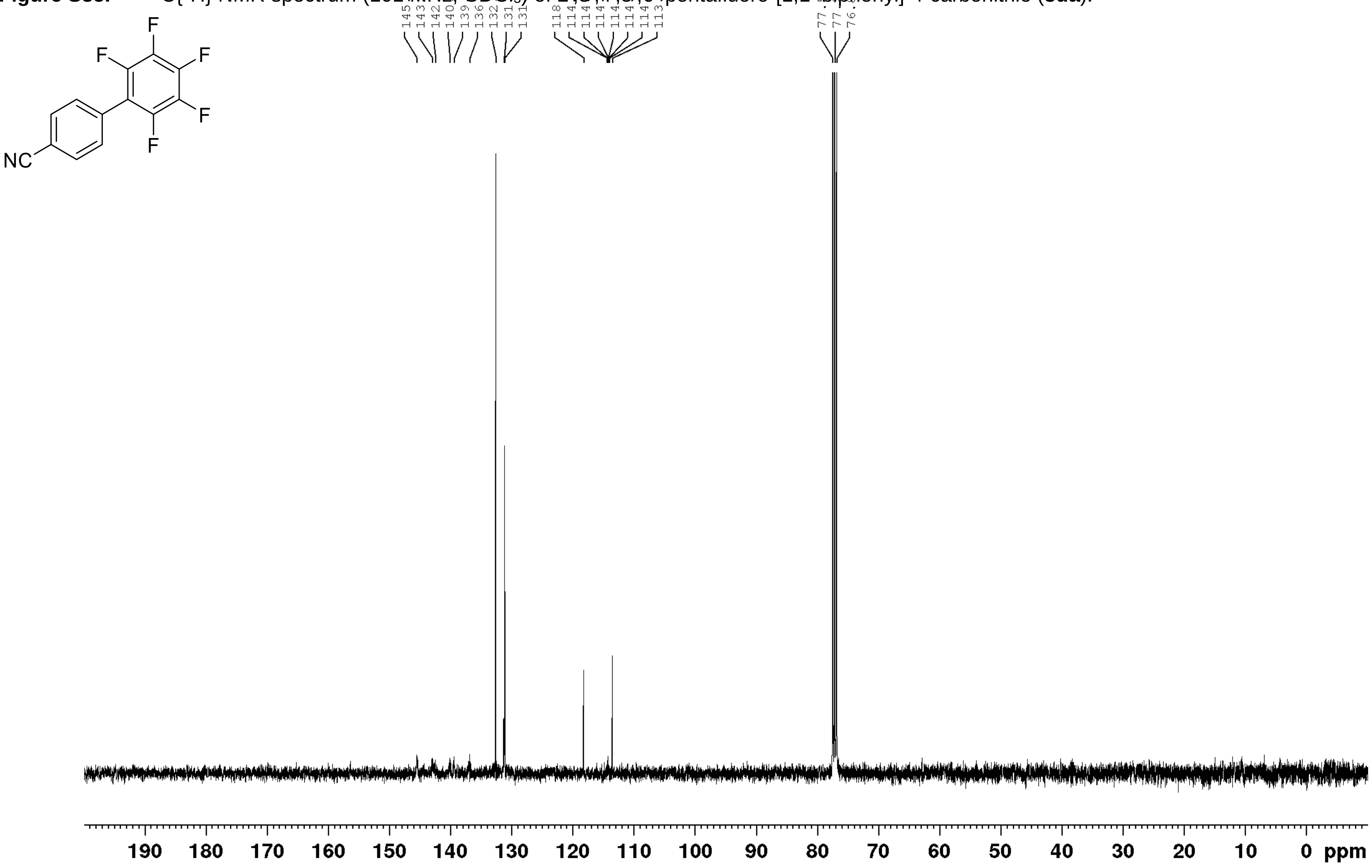

Figure S36. ^{19}F NMR spectrum (471 MHz, $CDCl_3$) of 2',3',4',5',6'-pentafluoro-[1,1'-biphenyl]-4-carbonitrile (**3da**).

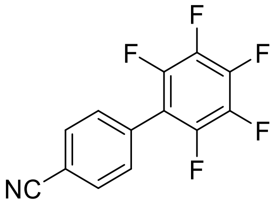

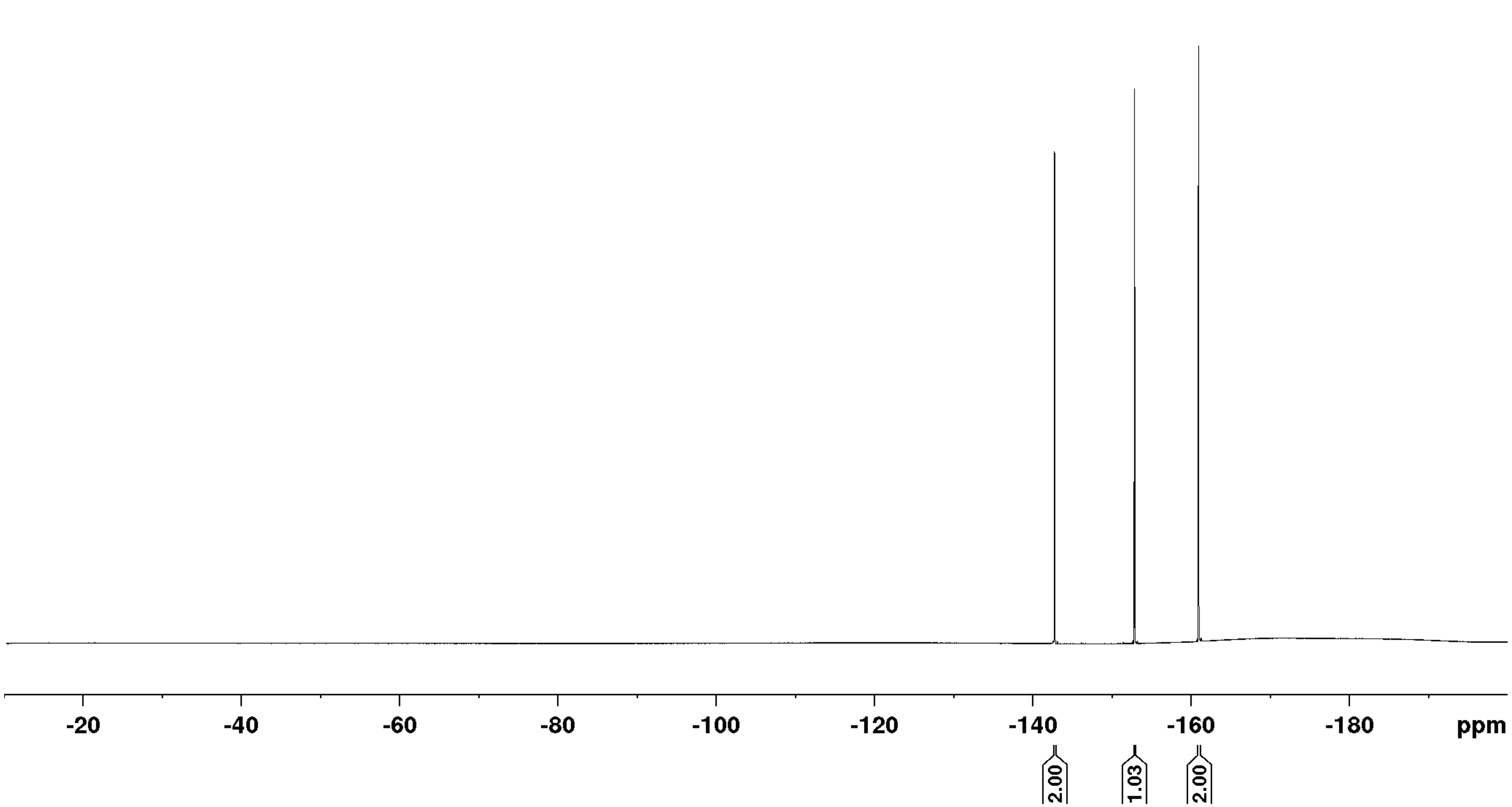

Figure S37. ^{1}H NMR spectrum (500 MHz, $CDCl_3$) of methyl 2',3',4',5',6'-pentafluoro-[1,1'-biphenyl]-4-carboxylate (**3ea**).

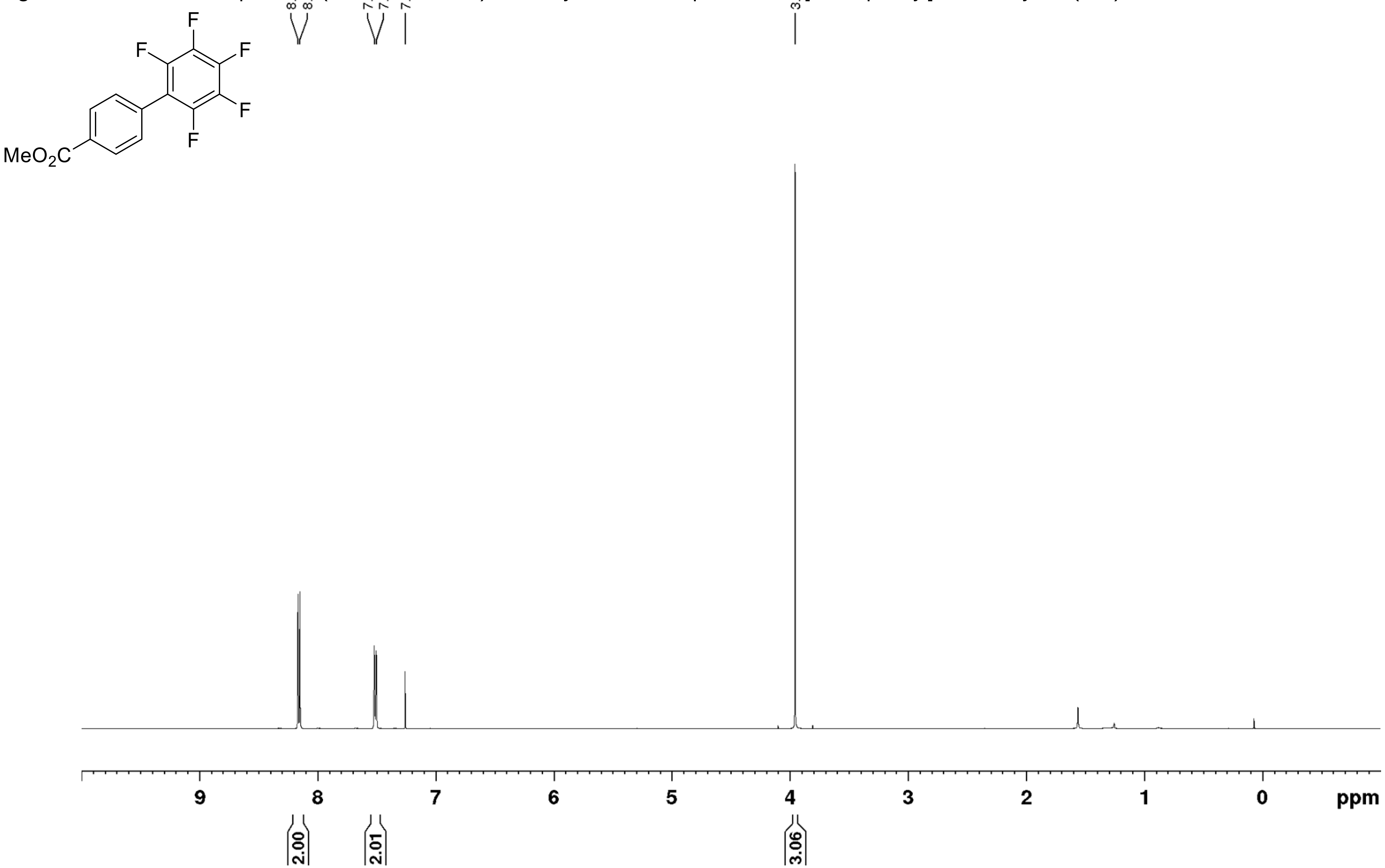

Figure S38. $^{13}C\{^{1}H\}$ NMR spectrum (126 MHz, $CDCl_3$) of methyl 2',3',4',5',6'-pentafluoro-[1,1'-biphenyl]-4-carboxylate (**3ea**).

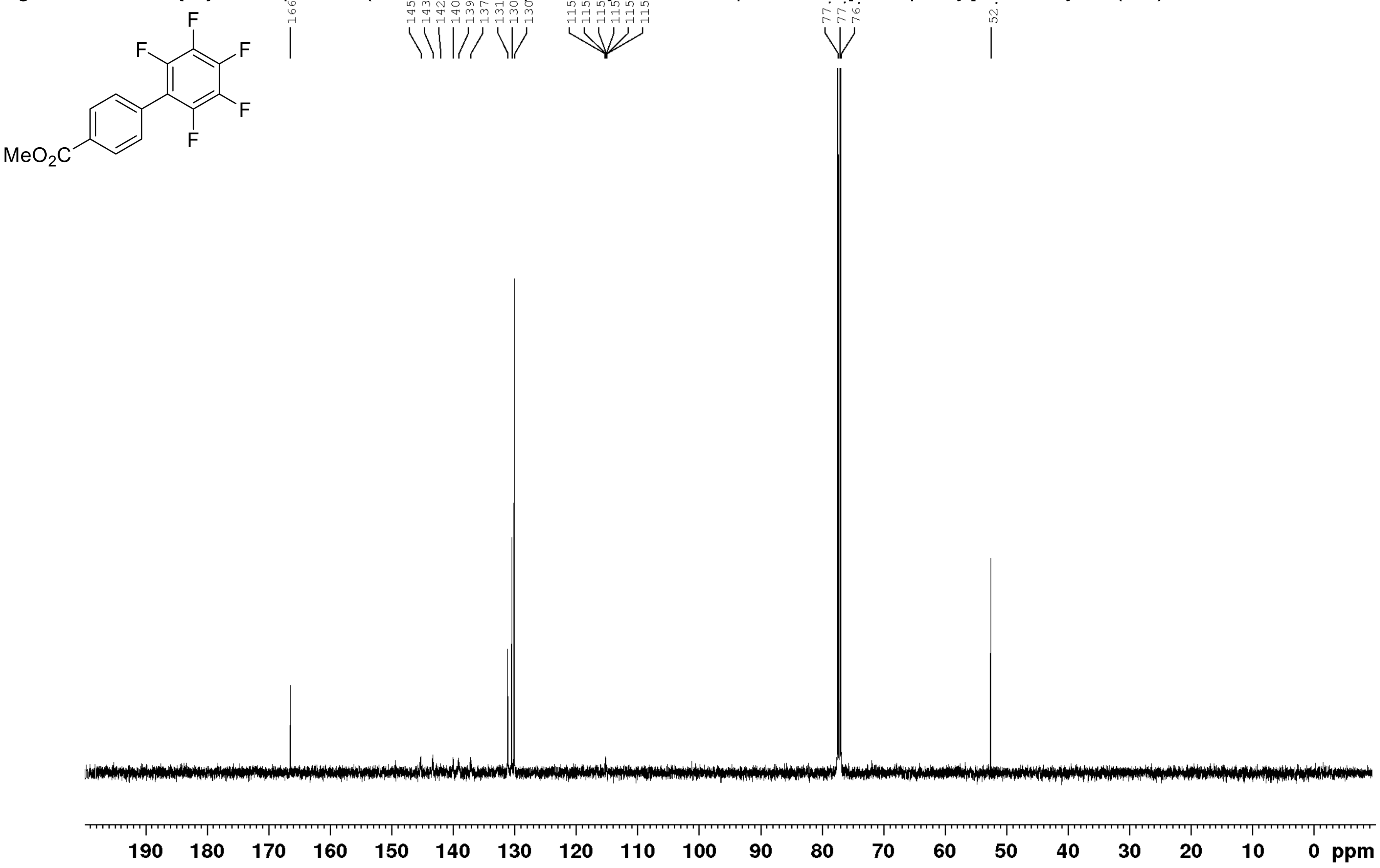

Figure S39. ^{19}F NMR spectrum (471 MHz, $CDCl_3$) of methyl 2',3',4',5',6'-pentafluoro-[1,1'-biphenyl]-4-carboxylate (**3ea**).

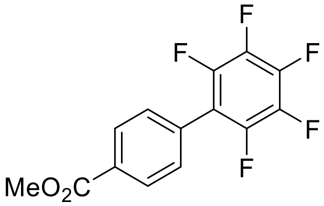

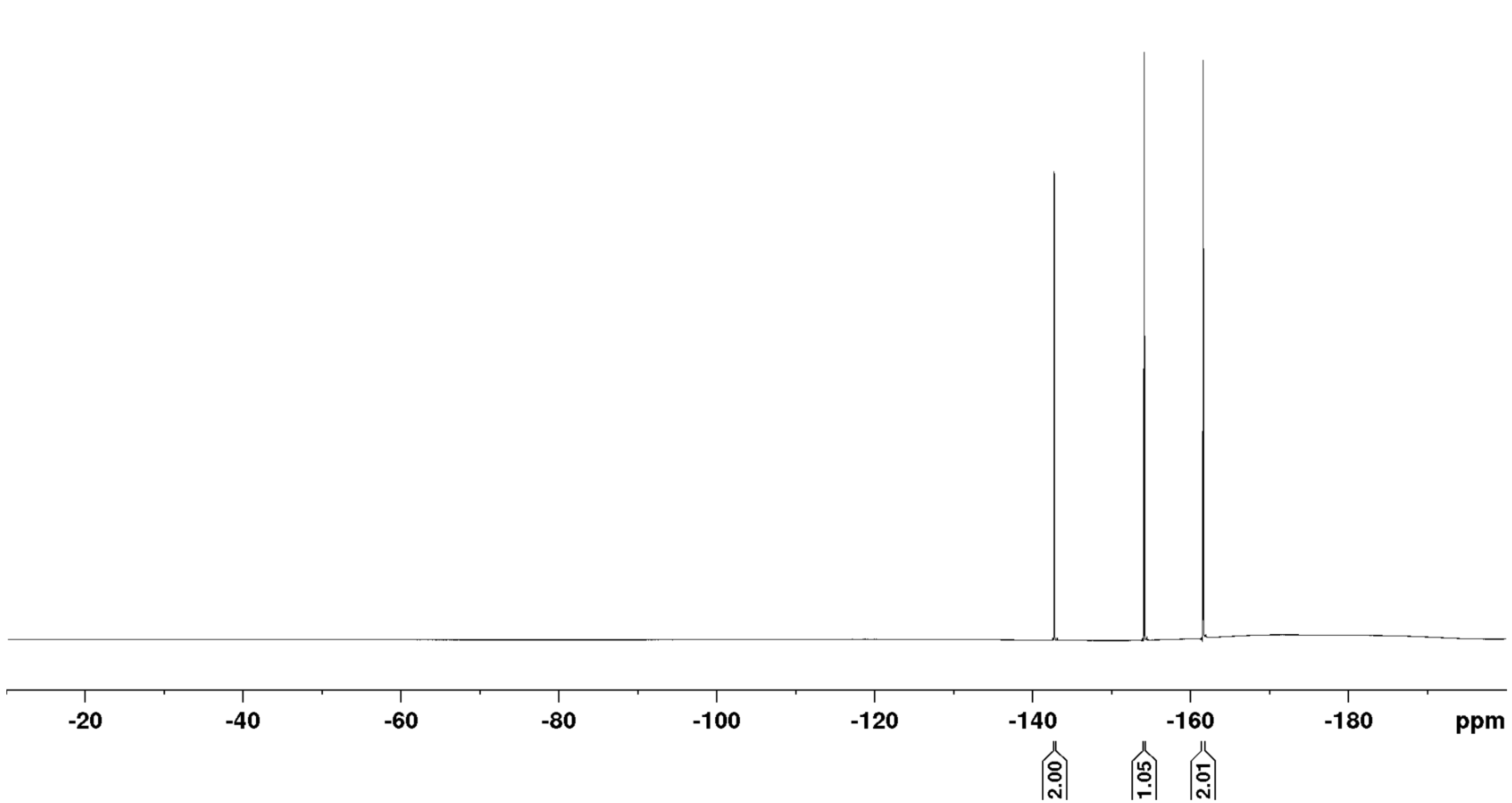

Figure S40. ^{1}H NMR spectrum (500 MHz, $CDCl_3$) of methyl 2,3,4,5,6-pentafluoro-3'-nitro-1,1'-biphenyl (**3fa**).

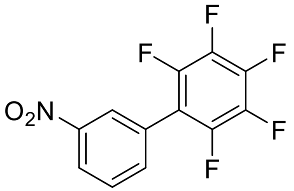

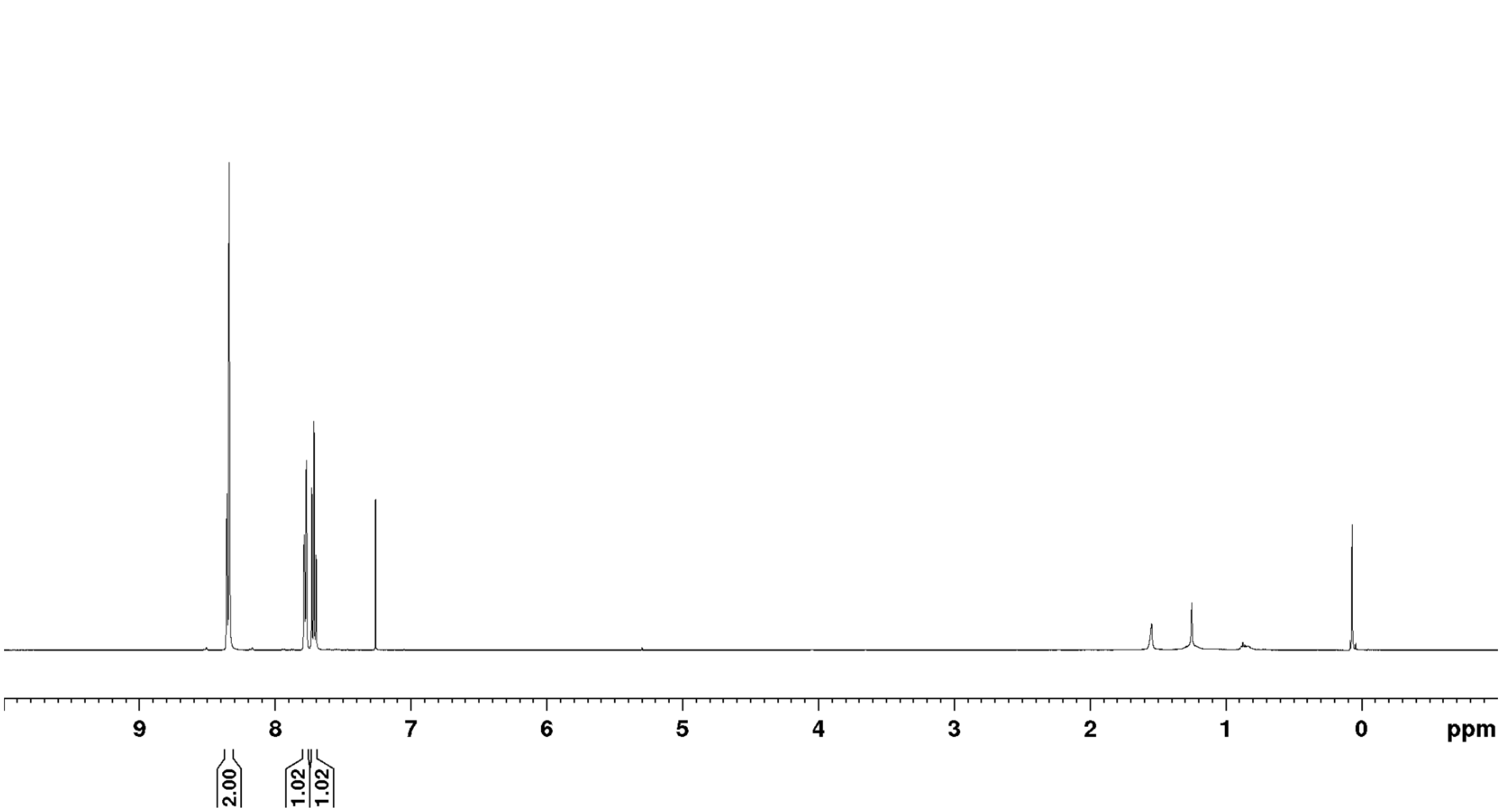

Figure S41. $^{13}C\{^1H\}$ NMR spectrum (126 MHz, $CDCl_3$) of methyl 2,3,4,5,6-pentafluoro-3'-nitro-1,1'-biphenyl (**3fa**).

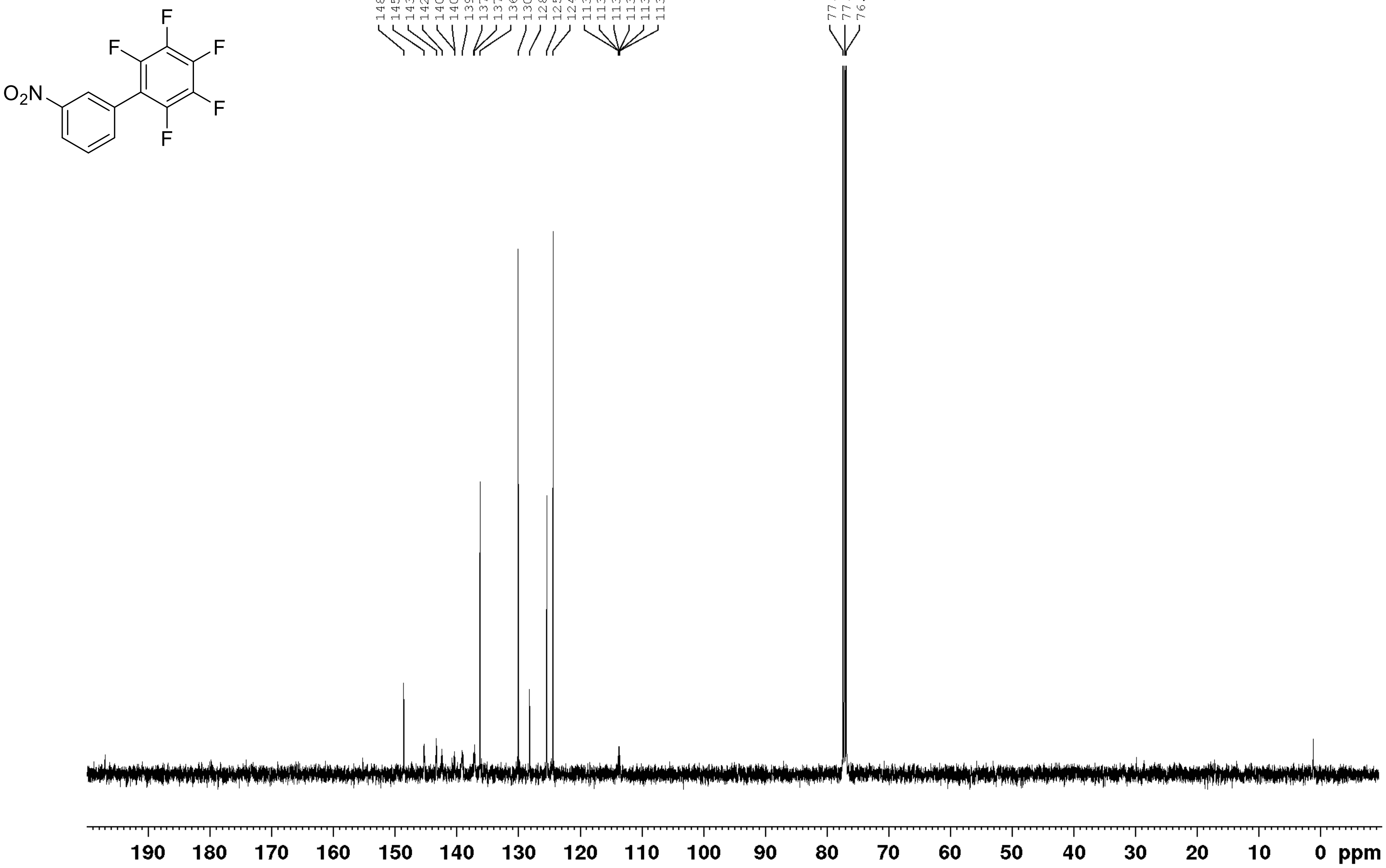

Figure S42. ^{19}F NMR spectrum (471 MHz, $CDCl_3$) of methyl 2,3,4,5,6-pentafluoro-3'-nitro-1,1'-biphenyl (**3fa**).

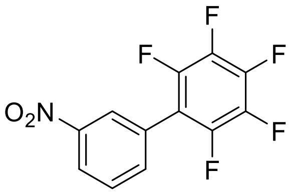

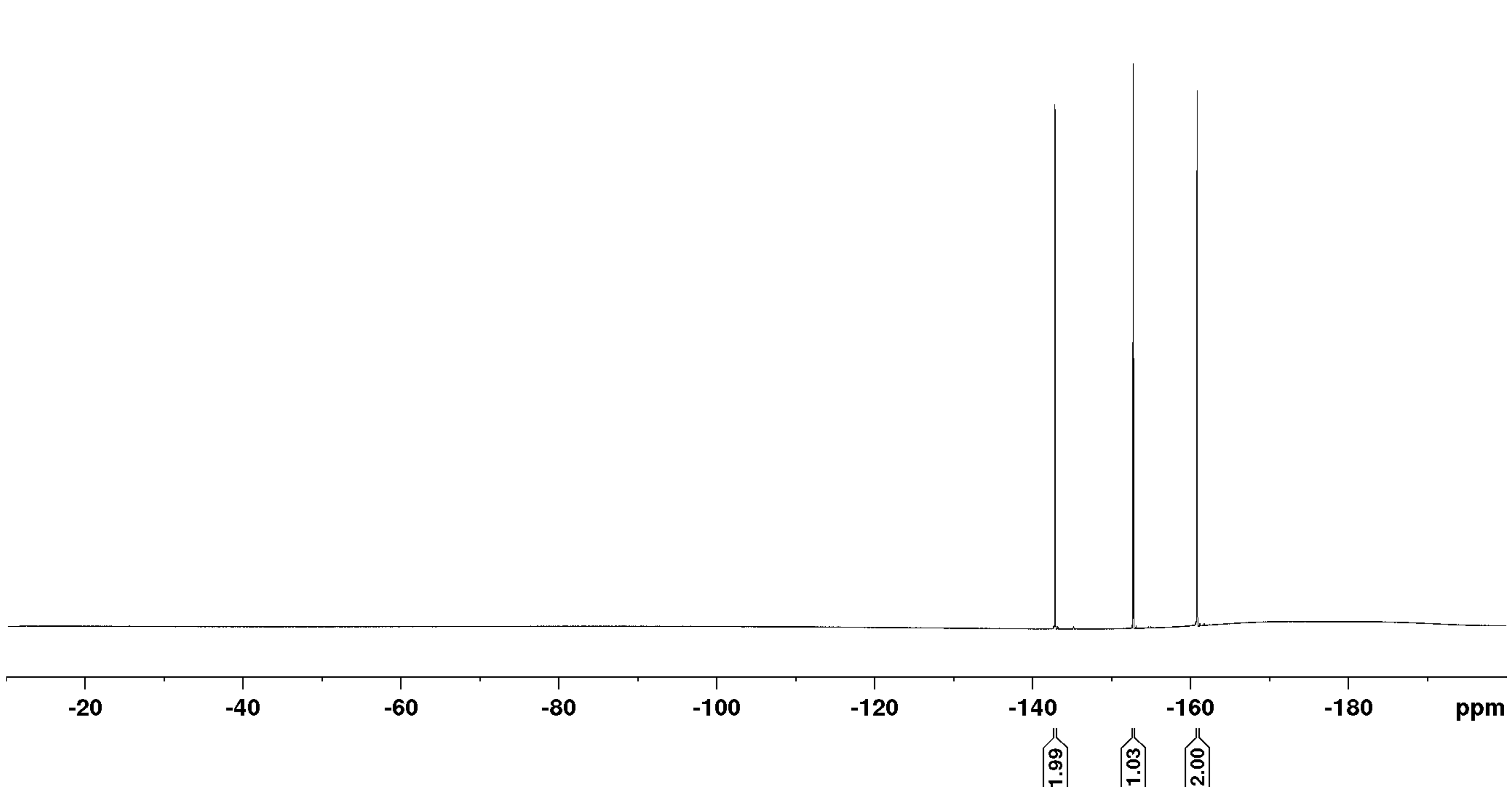

Figure S43. ^{1}H NMR spectrum (500 MHz, $CDCl_3$) of methyl 2,3,4,5,6-pentafluoro-4'-iodo-1,1'-biphenyl (**3ga**).

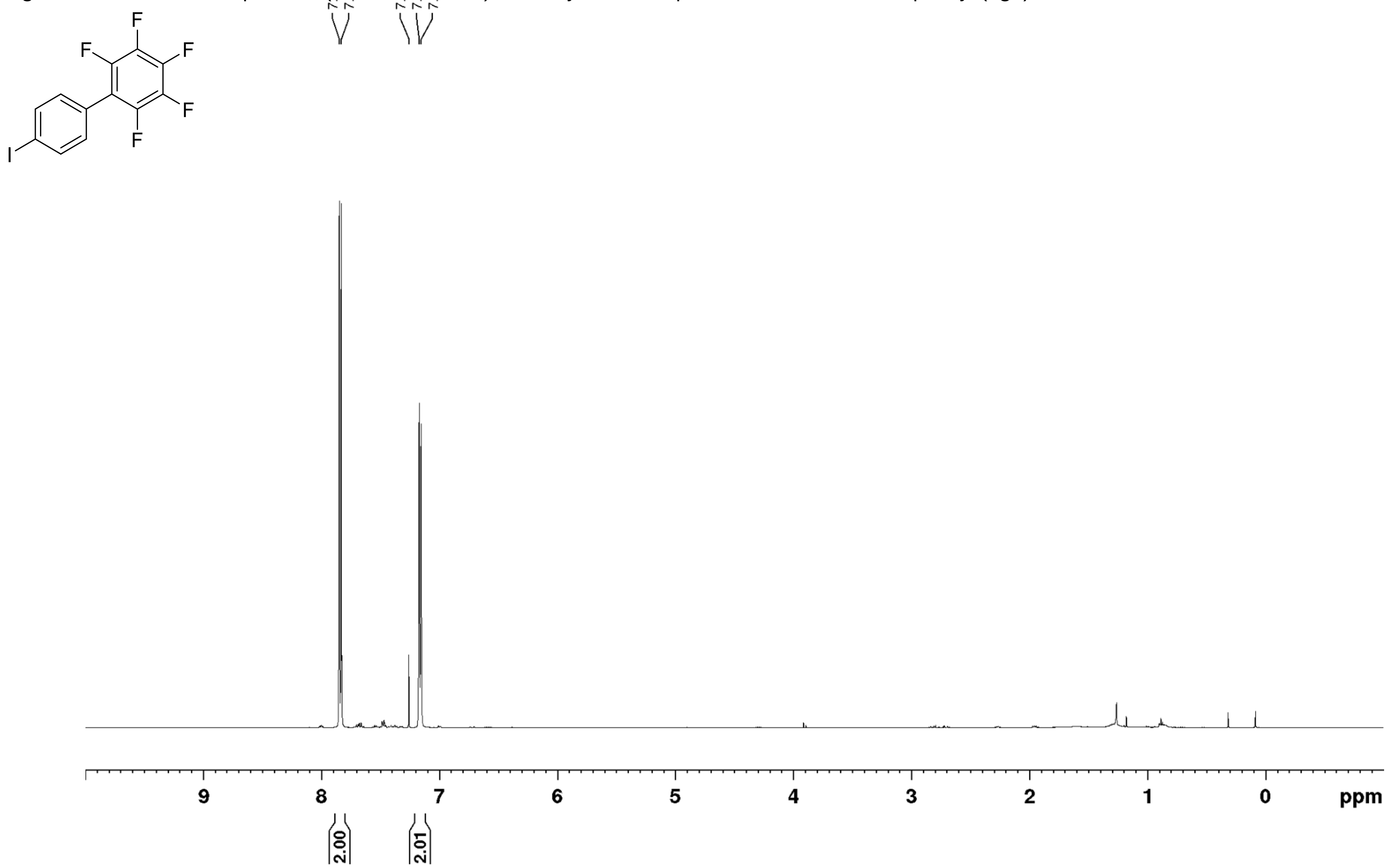

Figure S44. $^{13}C\{^{1}H\}$ NMR spectrum (126 MHz, $CDCl_3$) of methyl 2,3,4,5,6-pentafluoro-4'-iodo-1,1'-biphenyl (**3ga**).

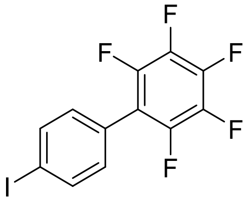

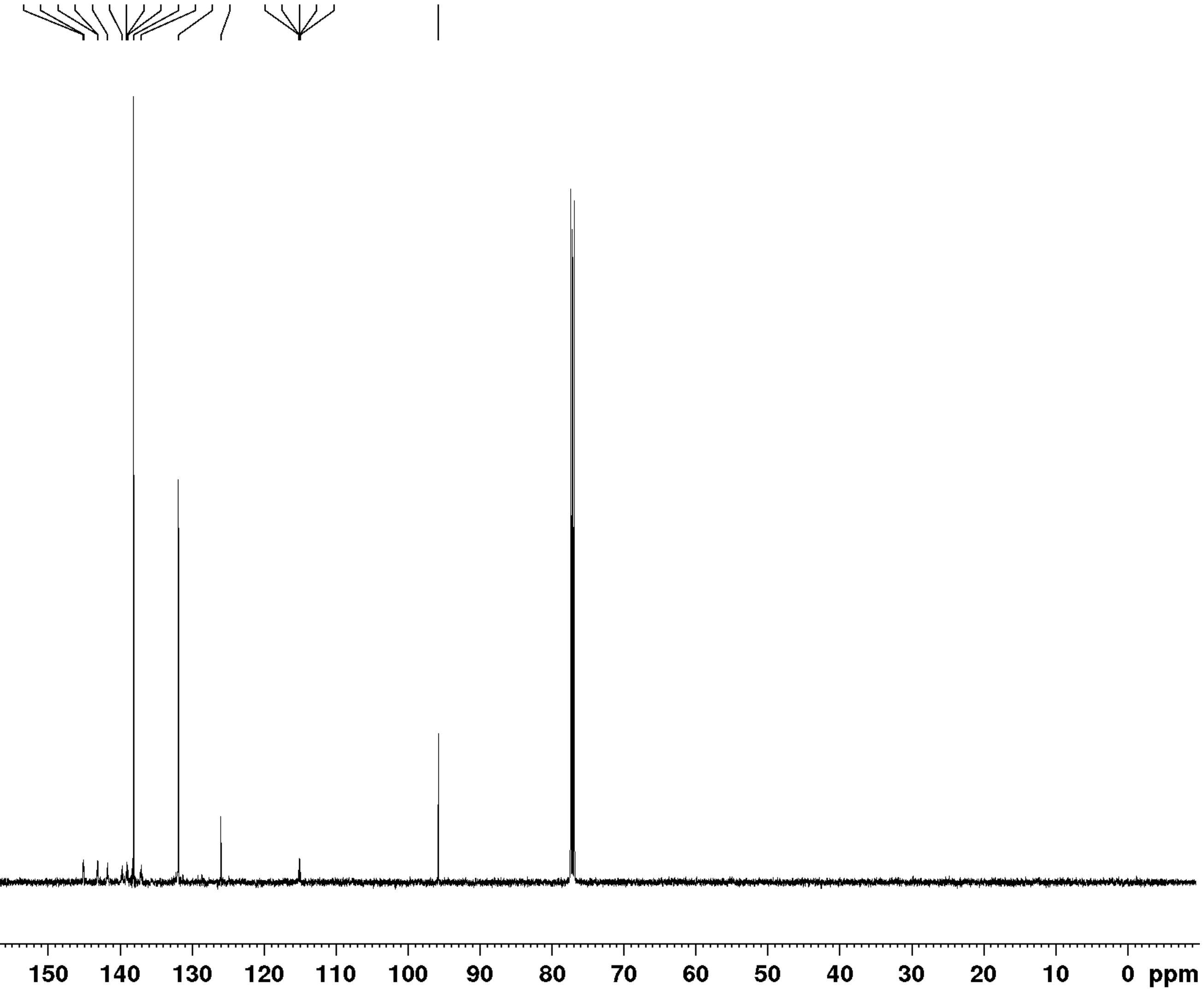

Figure S45. ^{19}F NMR spectrum (471 MHz, $CDCl_3$) of 2,3,4,5,6-pentafluoro-4'-iodo-1,1'-biphenyl (**3ga**).

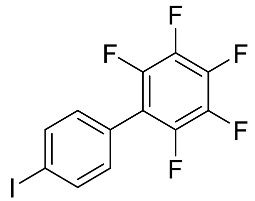

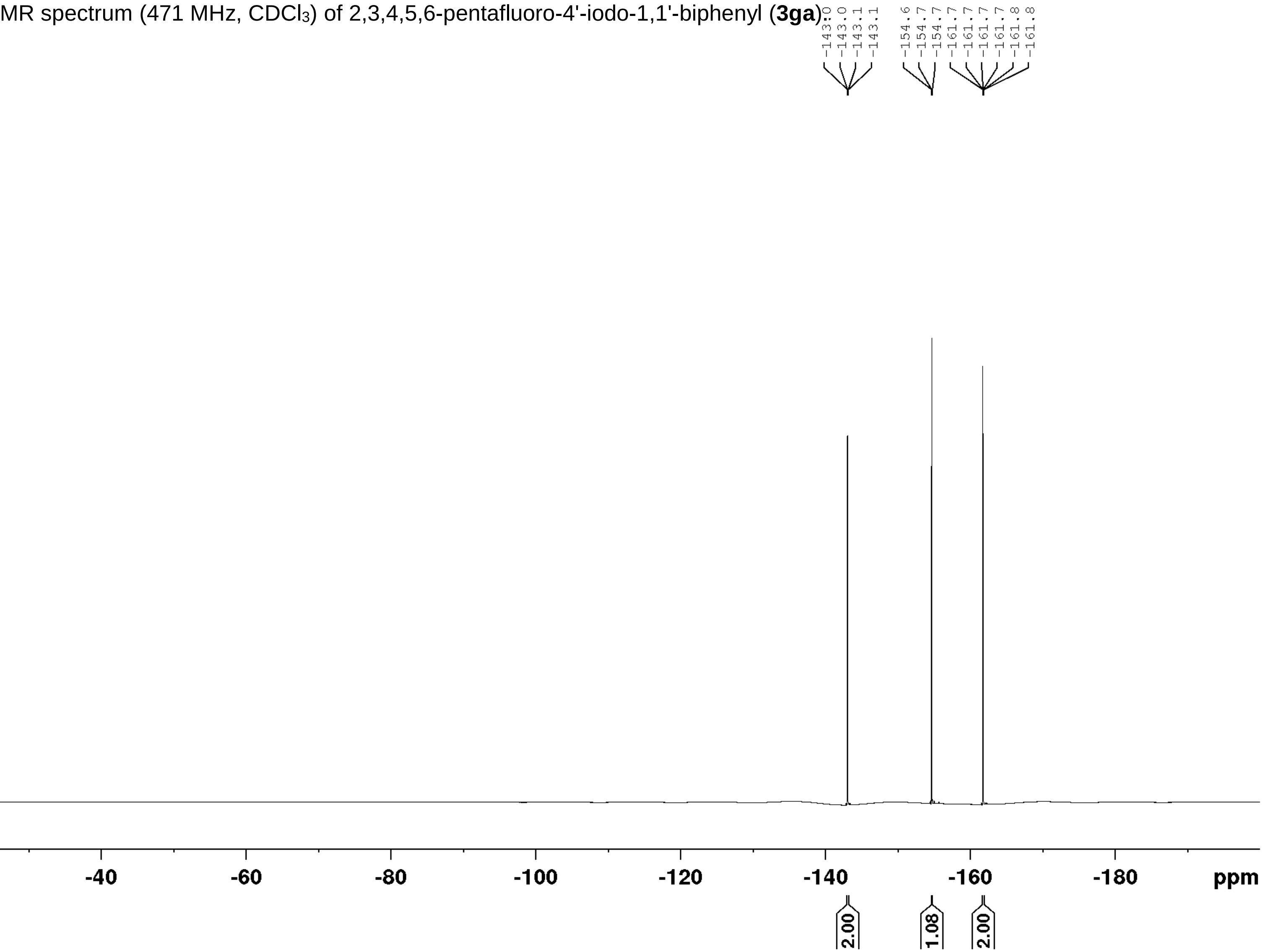

Figure S46. ^{1}H NMR spectrum (500 MHz, $CDCl_3$) of 4'-bromo-2,3,4,5,6-pentafluoro-1,1'-biphenyl (**3ha**).

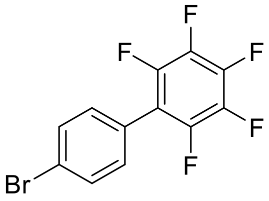

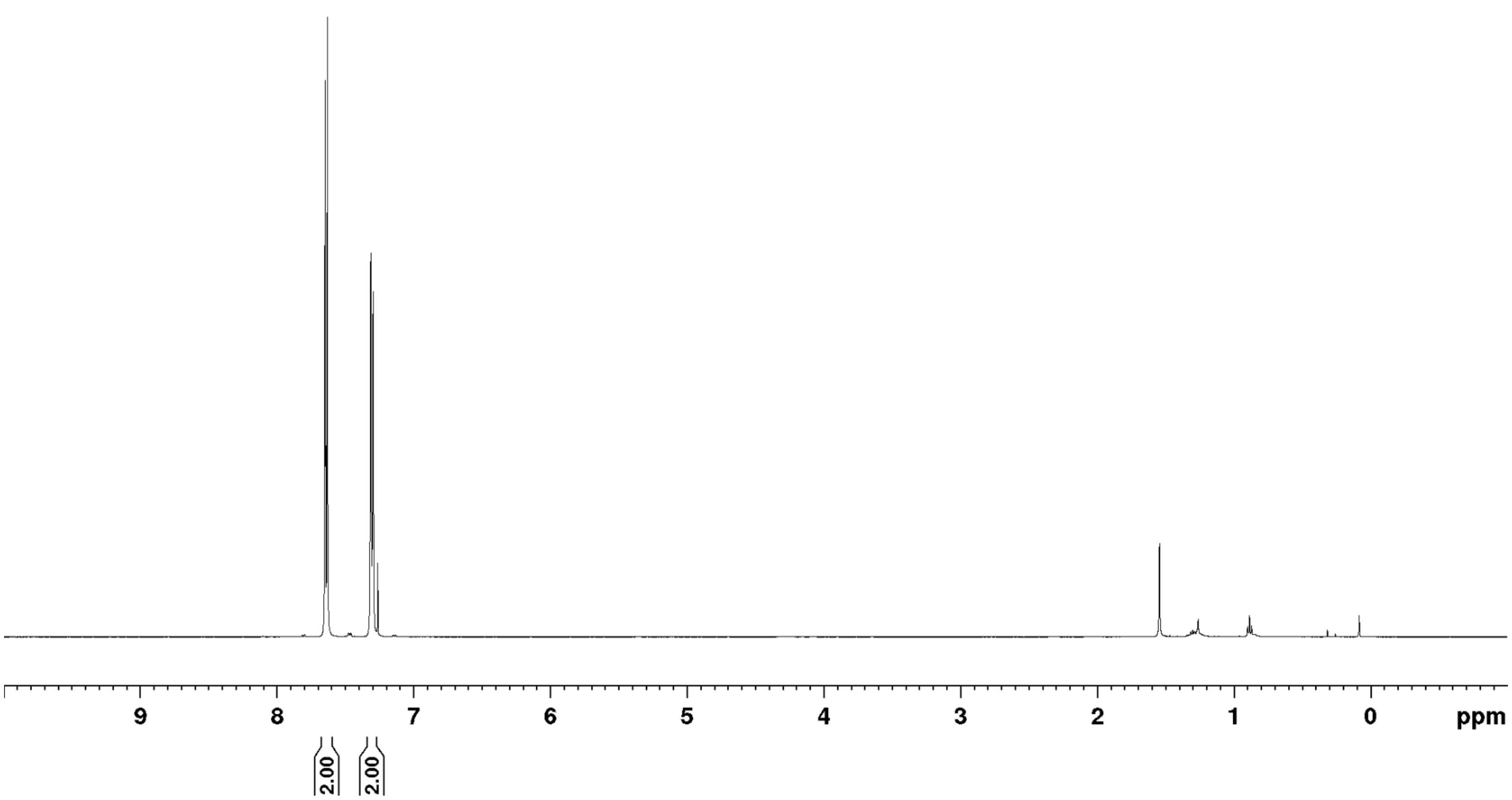

Figure S47. $^{13}C\{^{1}H\}$ NMR spectrum (126 MHz, $CDCl_3$) of 4'-bromo-2,3,4,5,6-pentafluoro-1,1'-biphenyl (**3ha**).

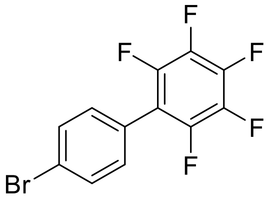

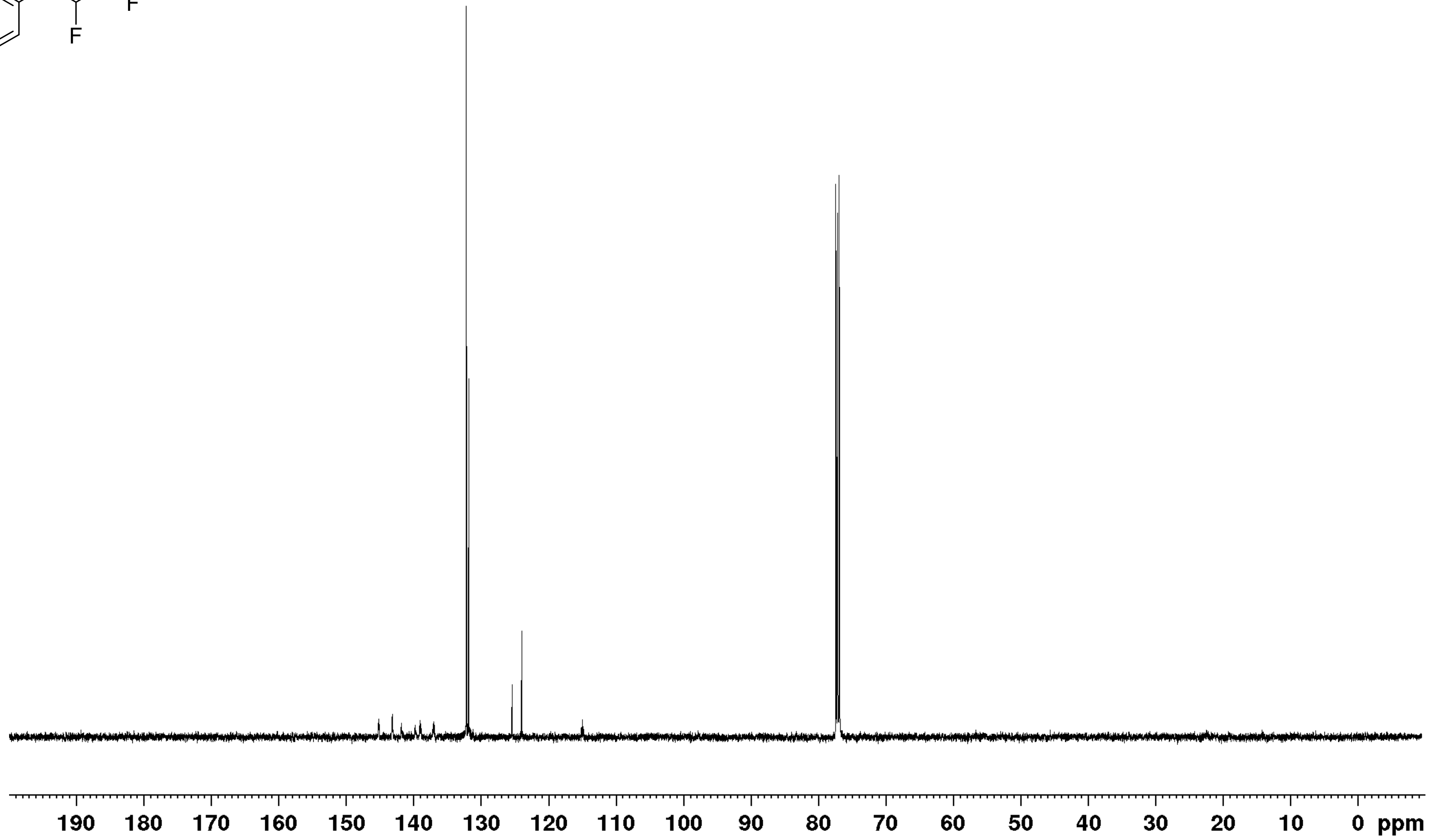

Figure S48. ^{19}F NMR spectrum (471 MHz, $CDCl_3$) of 4'-bromo-2,3,4,5,6-pentafluoro-1,1'-biphenyl (**3ha**).

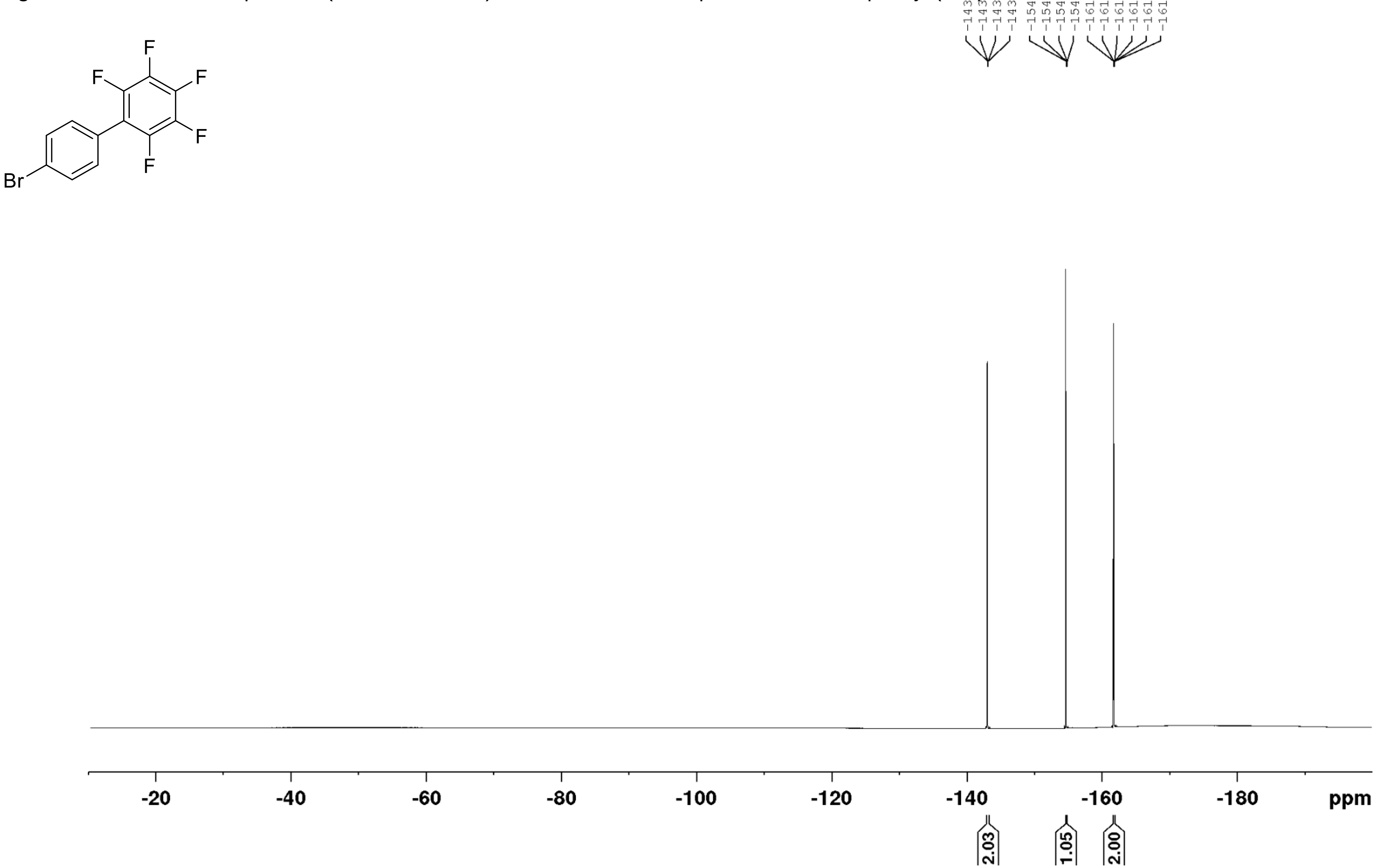

Figure S49. ^{1}H NMR spectrum (400 MHz, $CDCl_3$) of 4'-chloro-2,3,4,5,6-pentafluoro-1,1'-biphenyl (**3ia**).

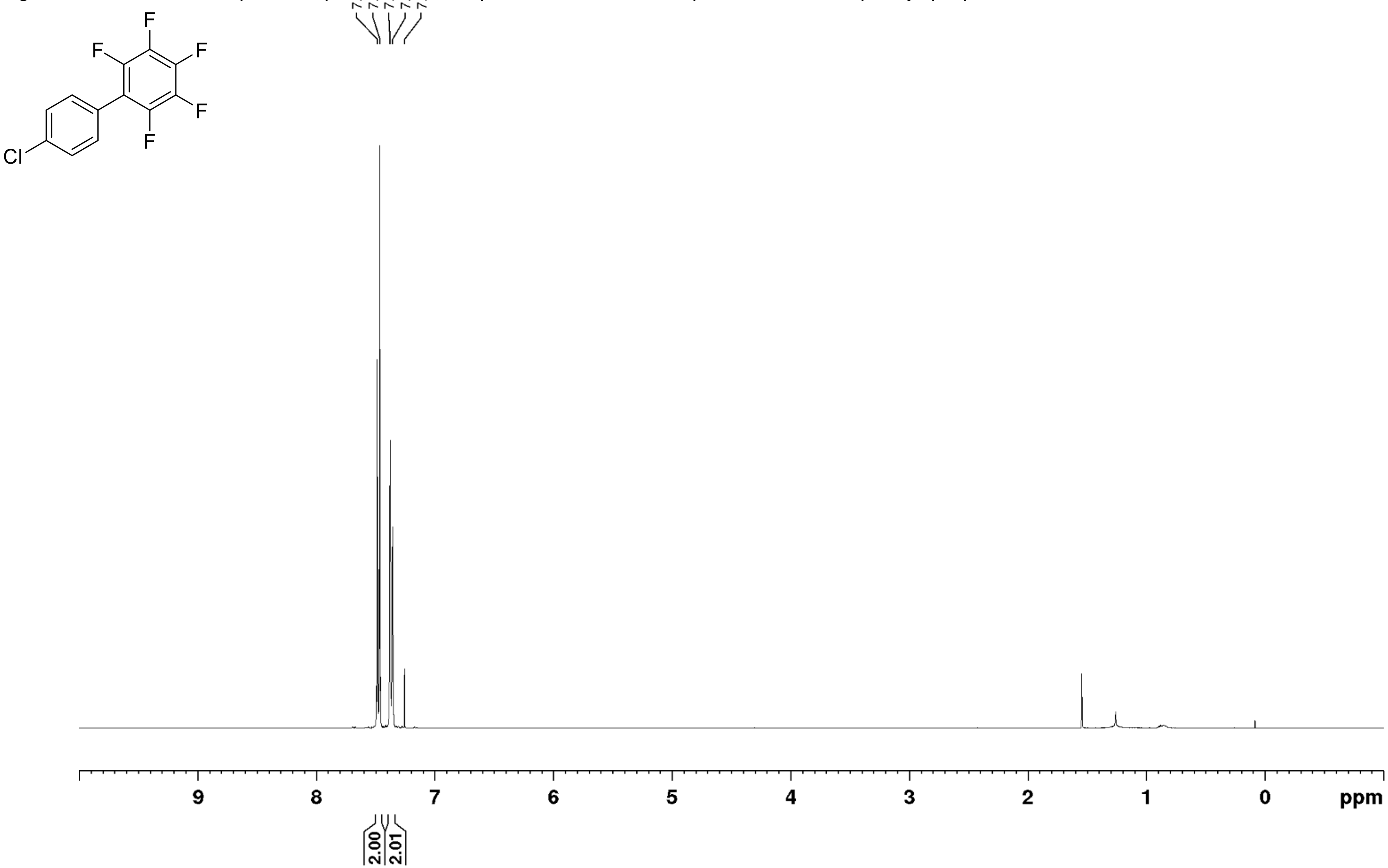

Figure S50. $^{13}C\{^{1}H\}$ NMR spectrum (101 MHz, $CDCl_3$) of 4'-chloro-2,3,4,5,6-pentafluoro-1,1'-biphenyl (**3ia**).

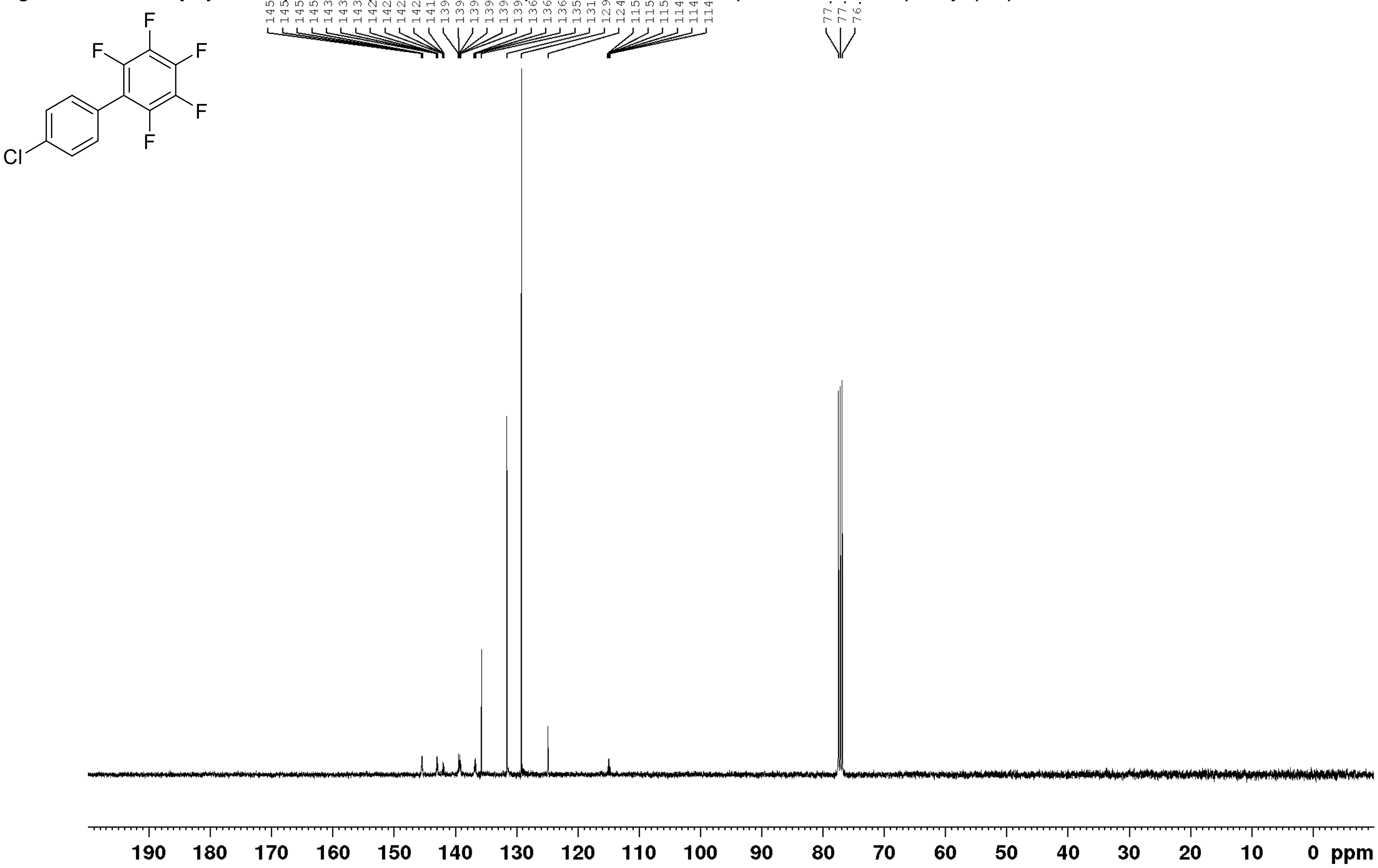

Figure S51. ^{19}F NMR spectrum (471 MHz, $CDCl_3$) of 4'-chloro-2,3,4,5,6-pentafluoro-1,1'-biphenyl (**3ia**).

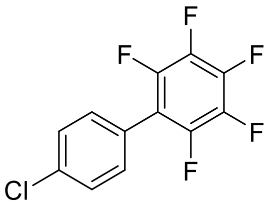

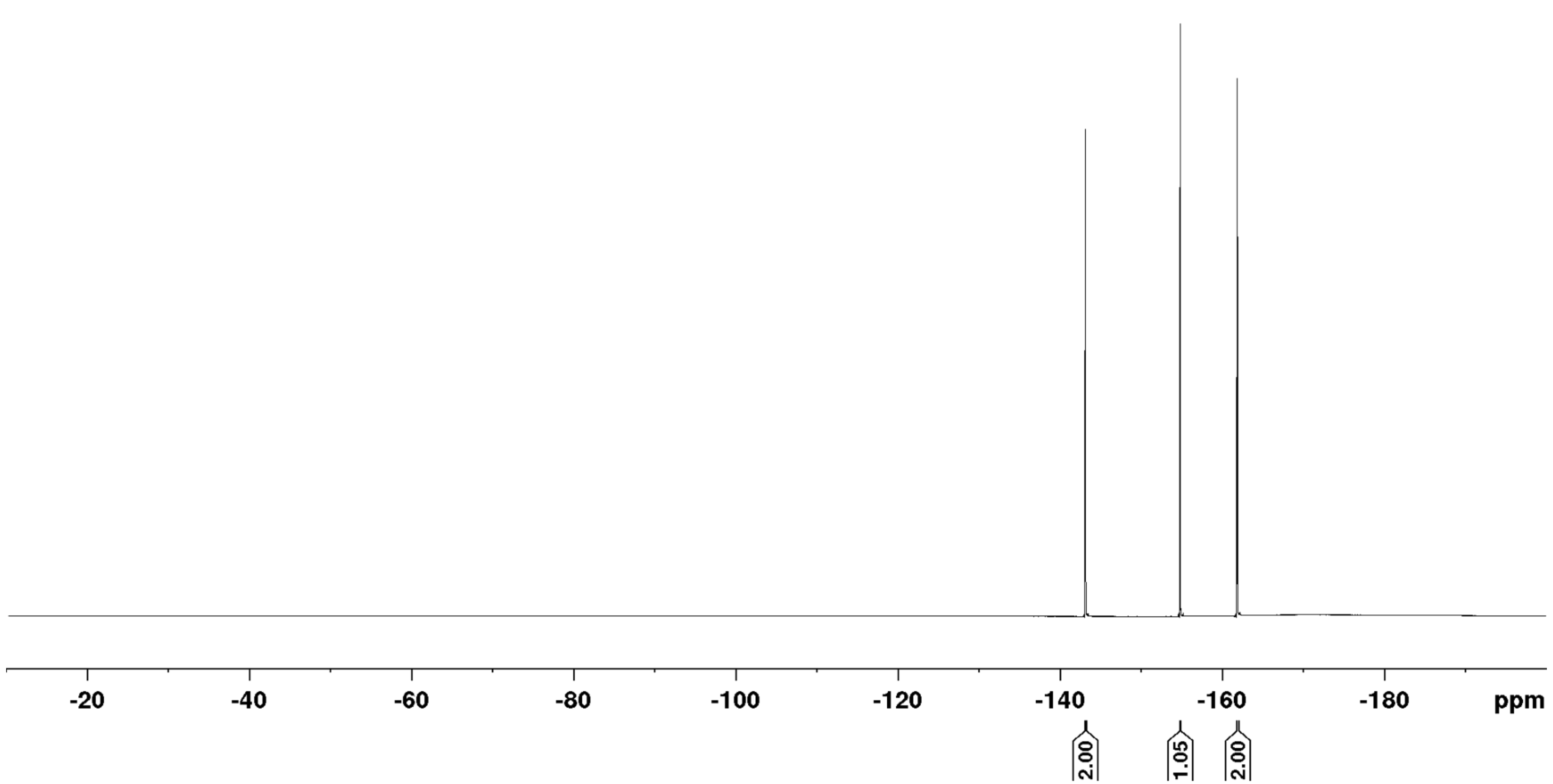

Figure S52. ^{1}H NMR spectrum (500 MHz, $CDCl_3$) of 2,3,4,4',5,6-hexafluoro-1,1'-biphenyl (**3ja**).

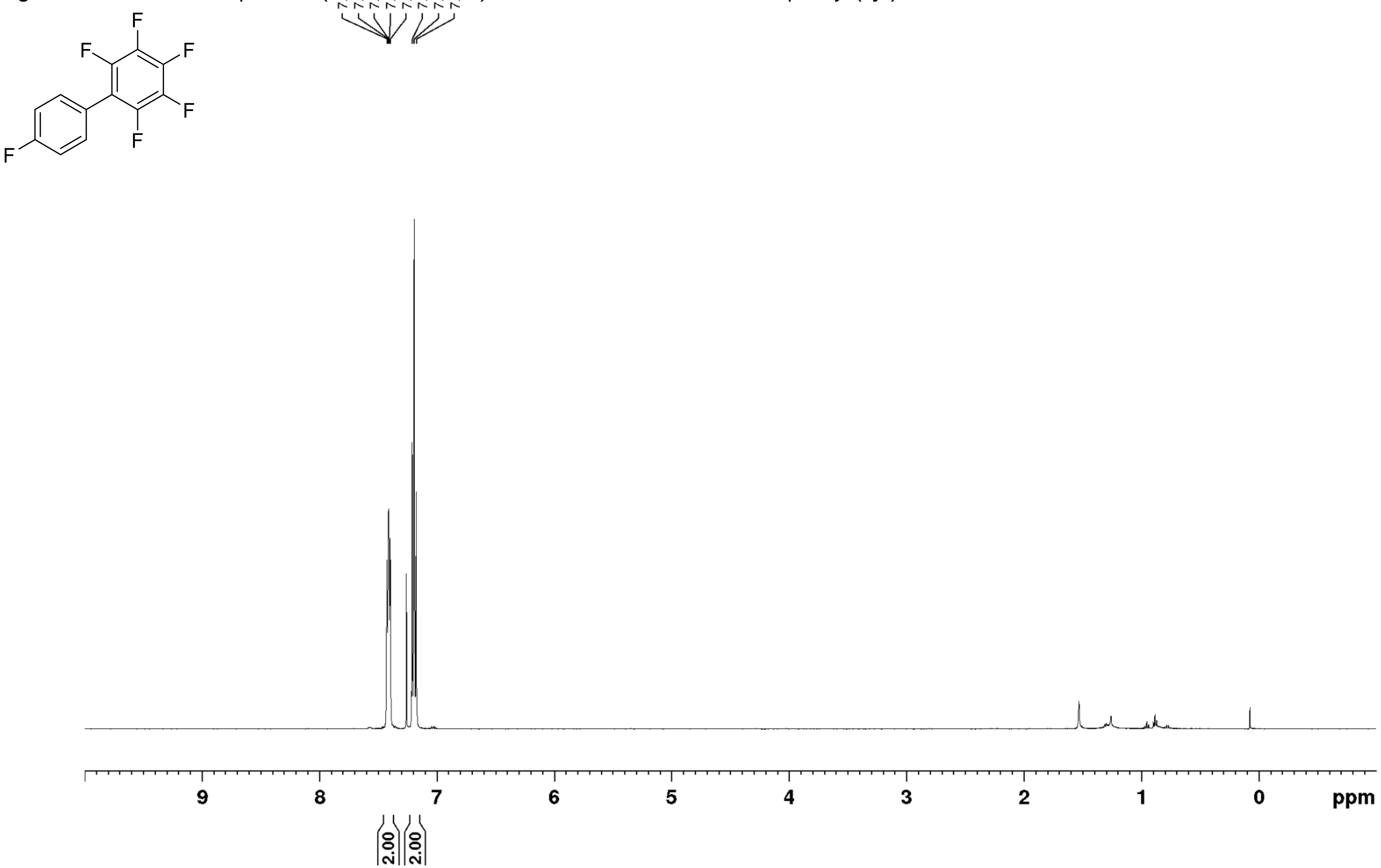

Figure S53. $^{13}C\{^{1}H\}$ NMR spectrum (126 MHz, $CDCl_3$) of 2,3,4,4',5,6-hexafluoro-1,1'-biphenyl (**3ja**).

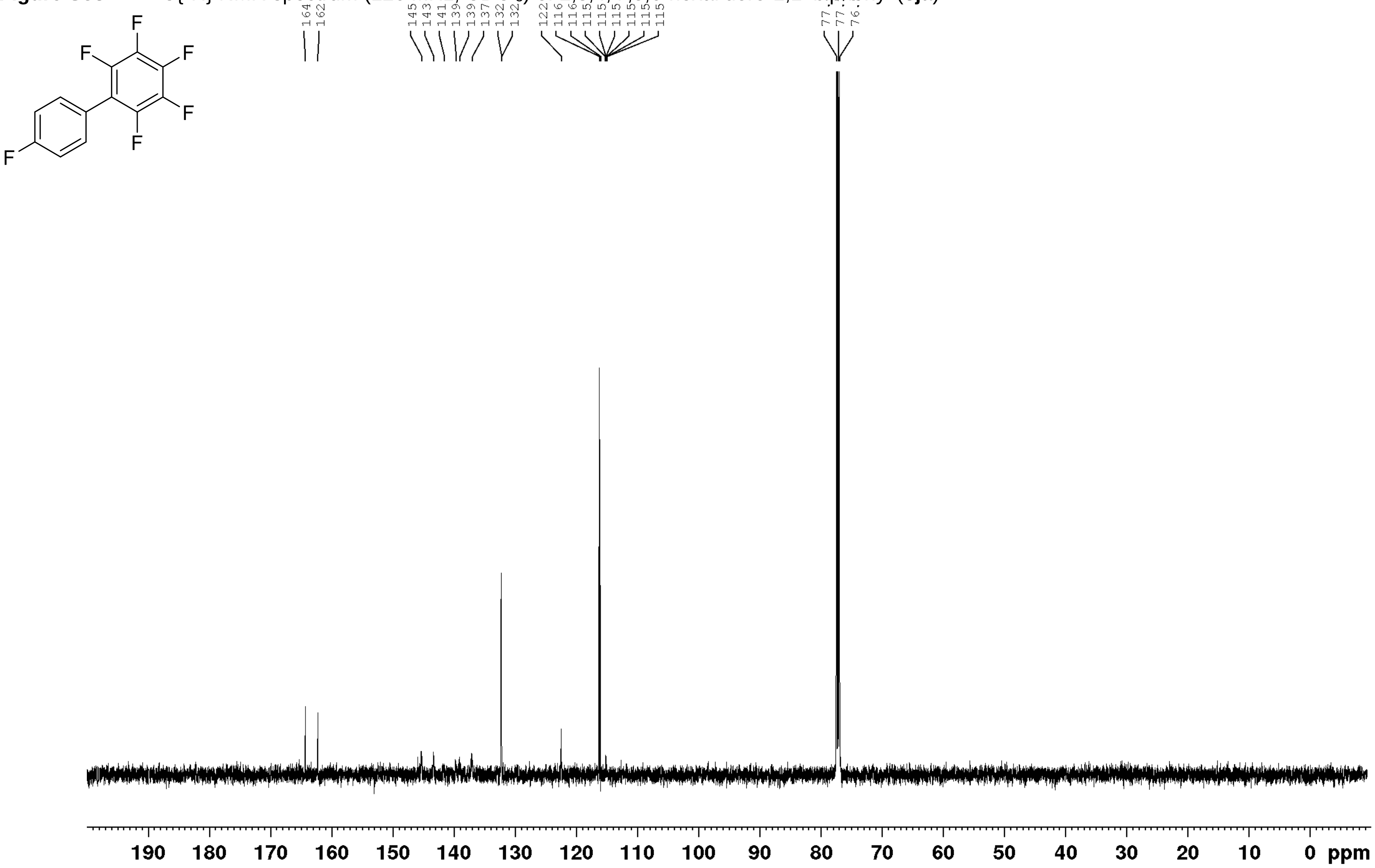

Figure S54. ^{19}F NMR spectrum (471 MHz, $CDCl_3$) of 2,3,4,4',5,6-hexafluoro-1,1'-biphenyl (**3ja**).

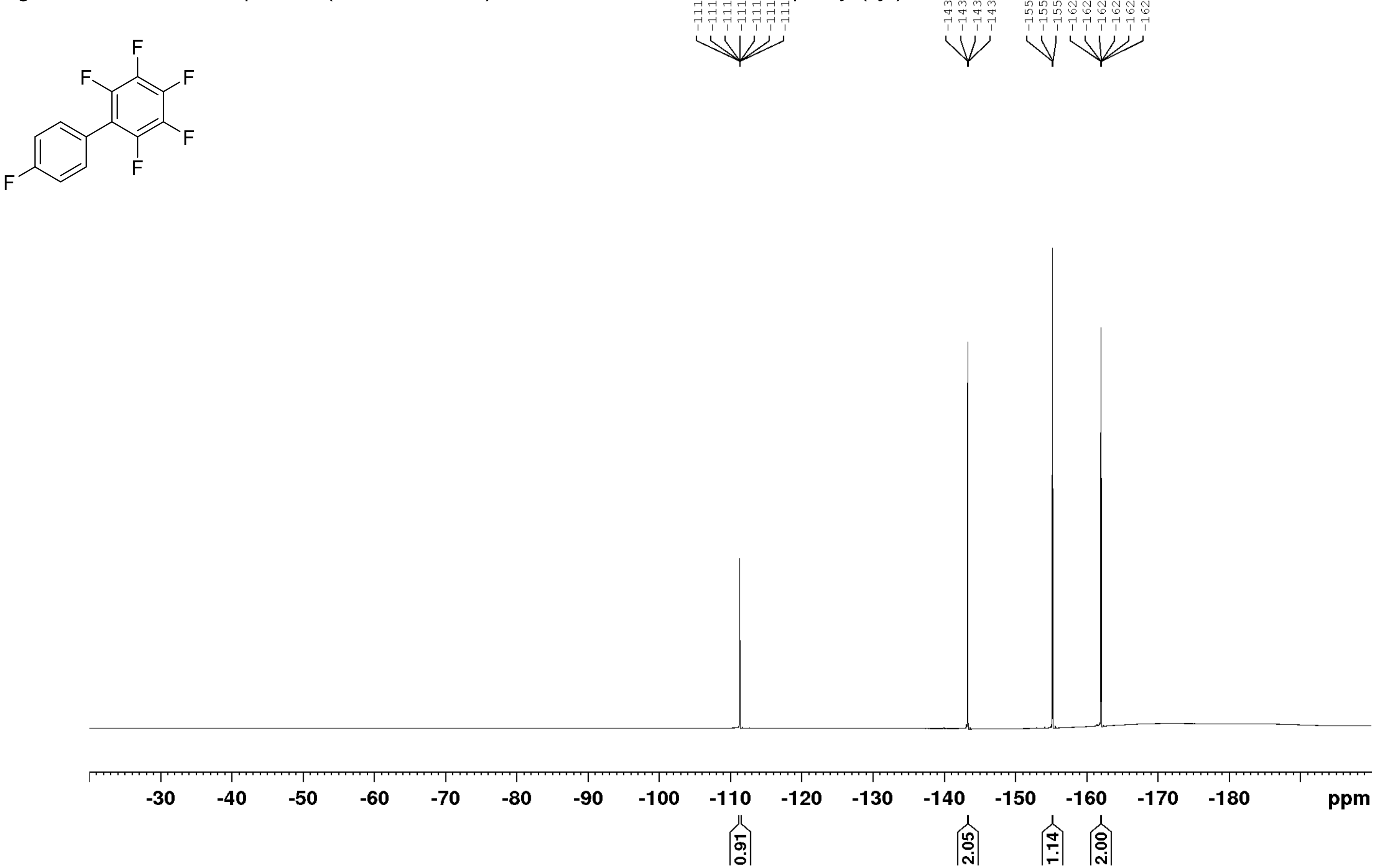

Figure S55. ^{1}H NMR spectrum (500 MHz, $CDCl_3$) of 2,3,4,5,6-pentafluoro-4'-(trifluoromethyl)-1,1'-biphenyl (**3ka**).

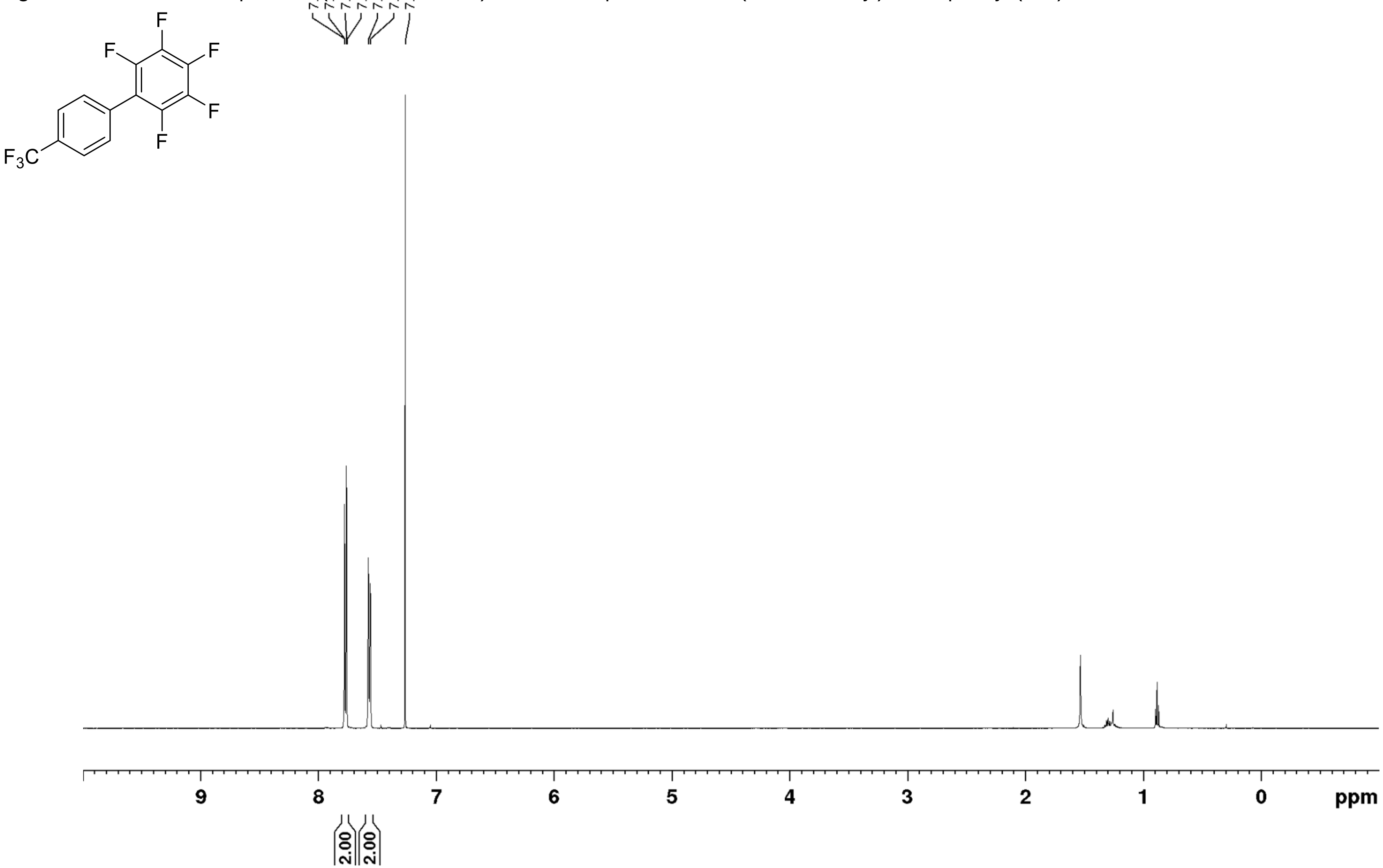

Figure S56. $^{13}C\{^{1}H\}$ NMR spectrum (126 MHz, $CDCl_3$) of 2,3,4,5,6-pentafluoro-4'-(trifluoromethyl)-1,1'-biphenyl (**3ka**).

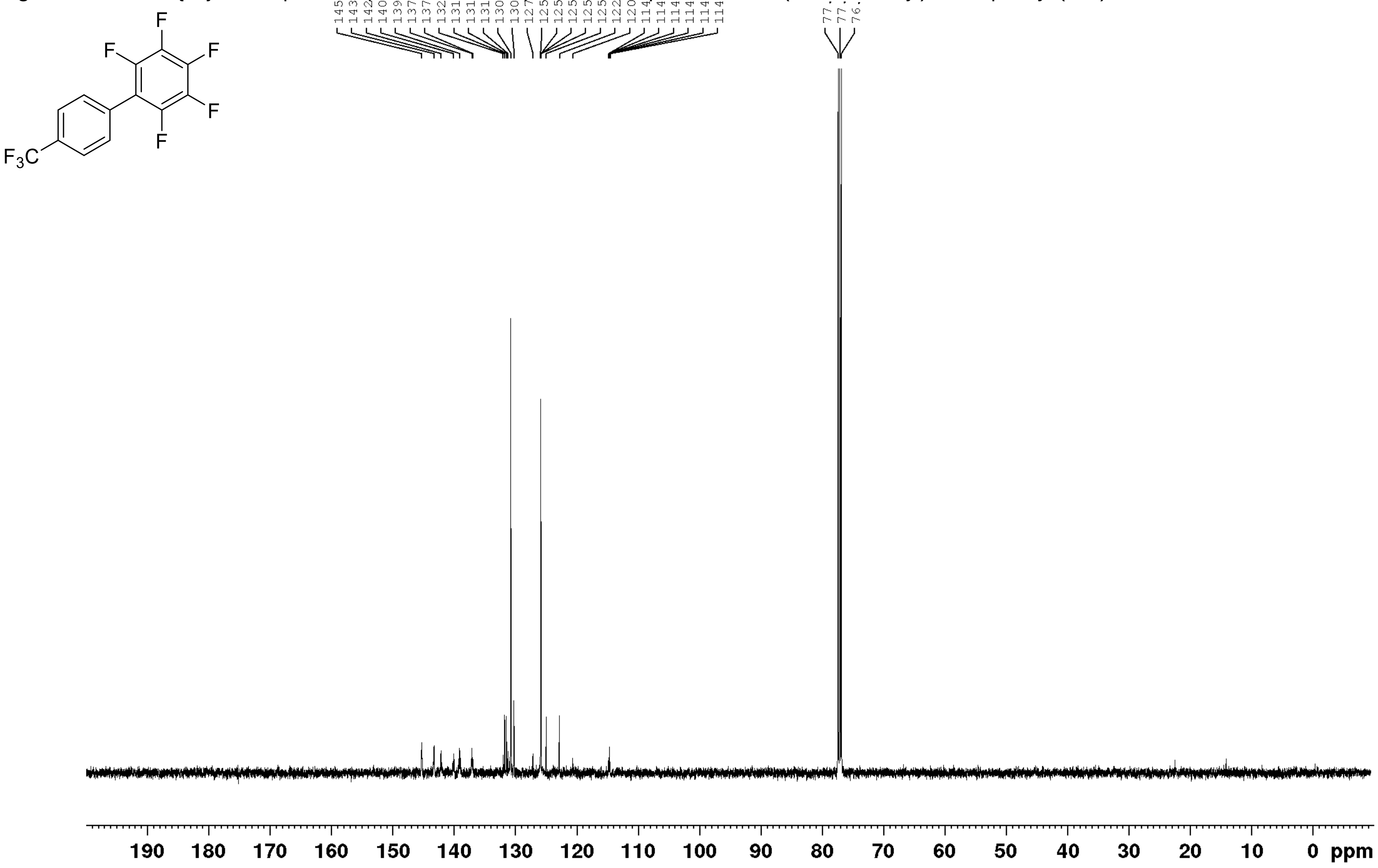

Figure S57. ^{19}F NMR spectrum (471 MHz, $CDCl_3$) of 2,3,4,5,6-pentafluoro-4'-(trifluoromethyl)-1,1'-biphenyl (**3ka**).

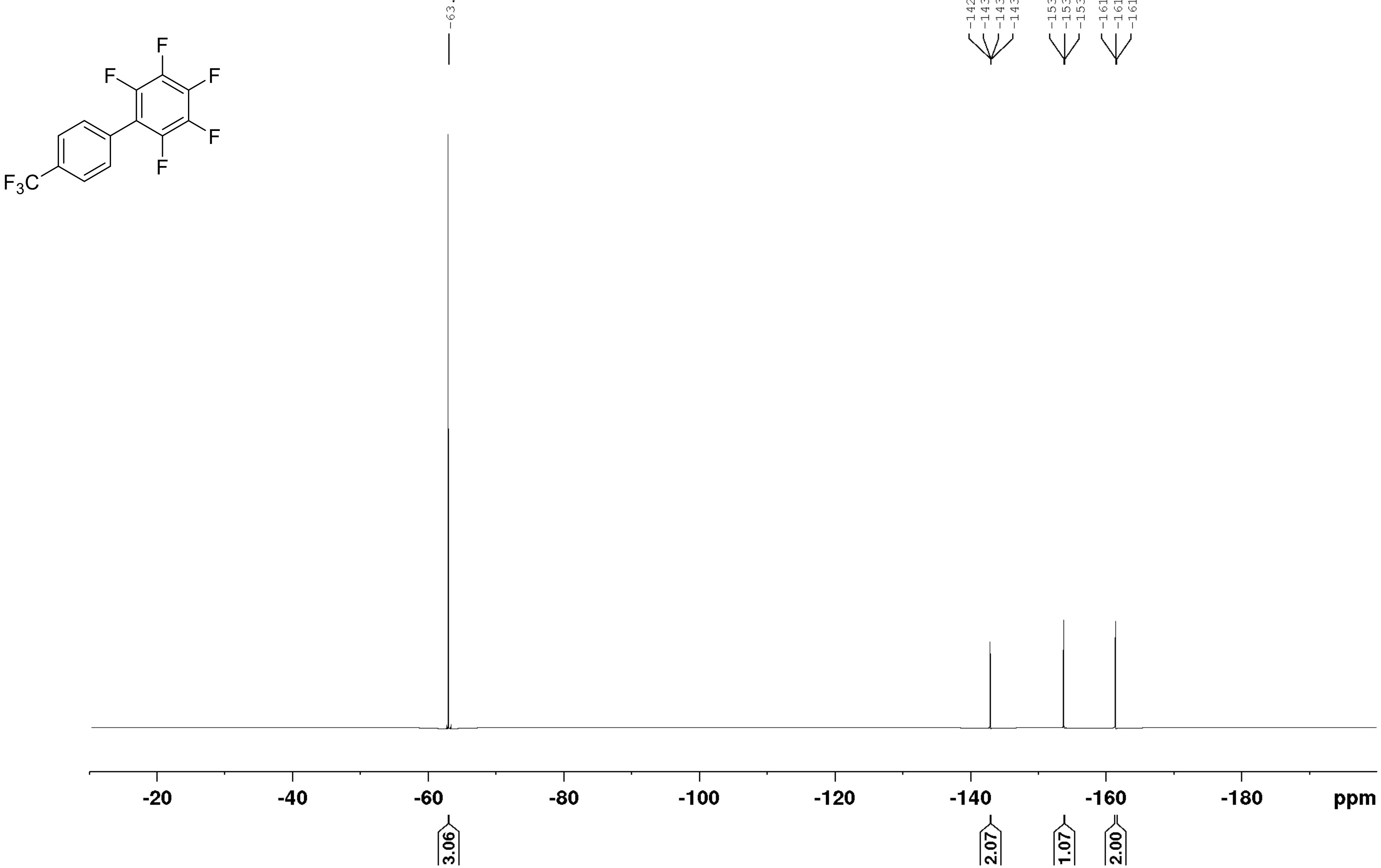

Figure S58. ^{1}H NMR spectrum (500 MHz, $CDCl_3$) of 2,2',3,4,5,6-hexafluoro-1,1'-biphenyl (**3la**).

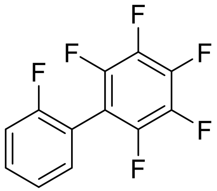

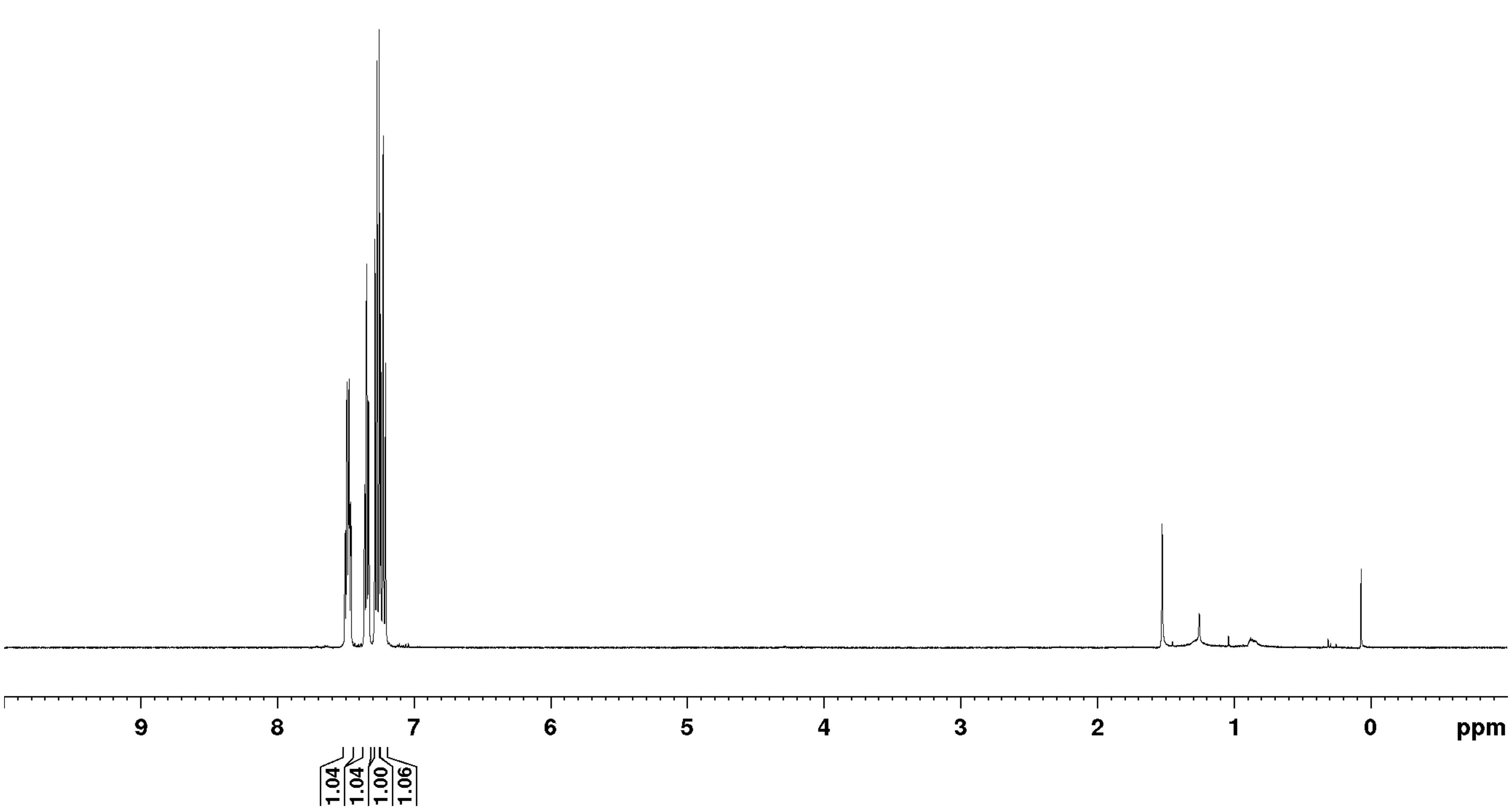

Figure S59. $^{13}C\{^{1}H\}$ NMR spectrum (126 MHz, $CDCl_3$) of 2,2',3,4,5,6-hexafluoro-1,1'-biphenyl (**3la**).

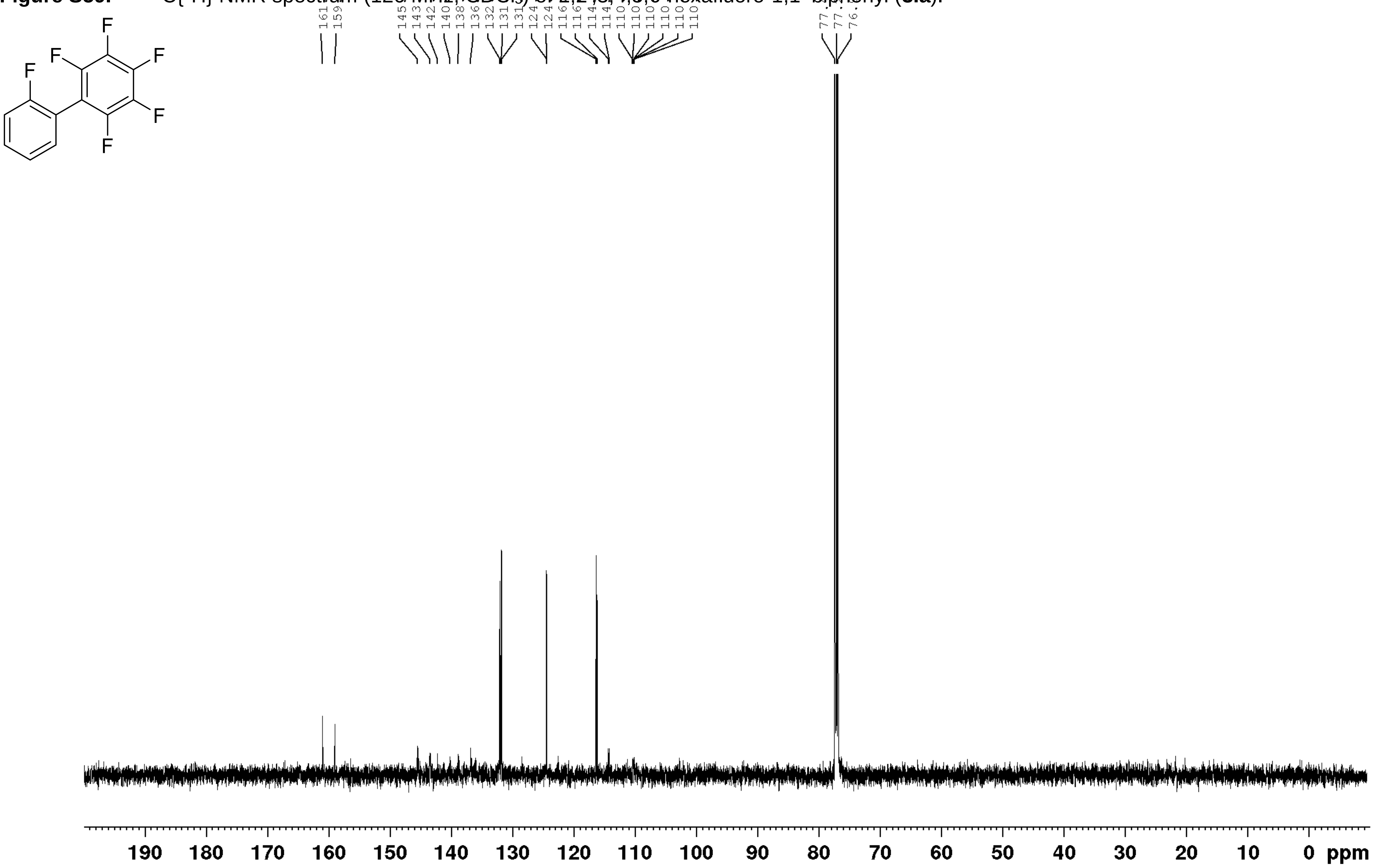

Figure S60. ^{19}F NMR spectrum (471 MHz, $CDCl_3$) of 2,2',3,4,5,6-hexafluoro-1,1'-biphenyl (**3la**).

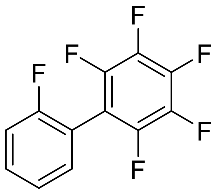

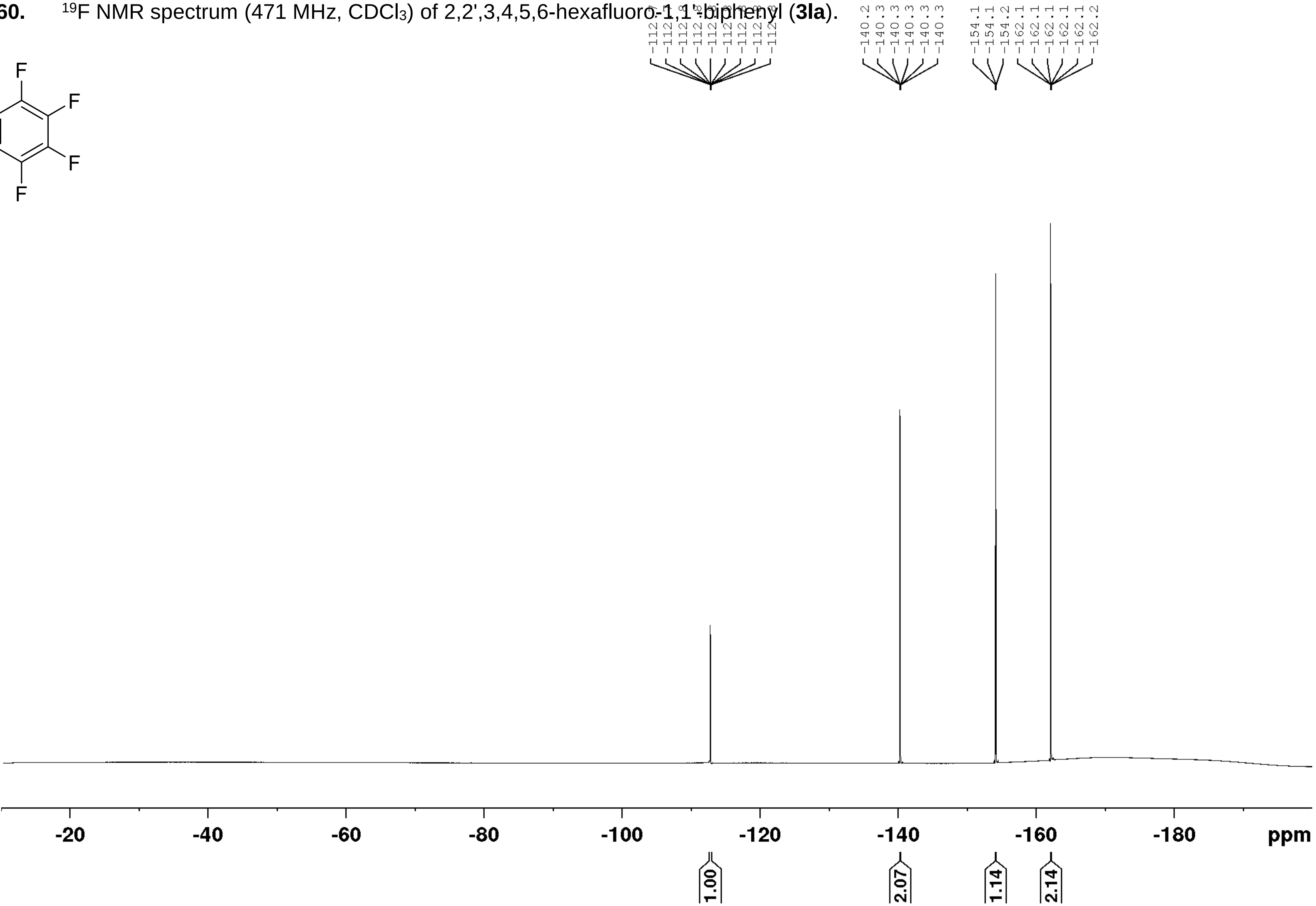

Figure S61. ^{1}H NMR spectrum (500 MHz, $CDCl_3$) of 2,3,4,4',5,6-hexafluoro-2',6'-dimethyl-1,1'-biphenyl (**3ma**).

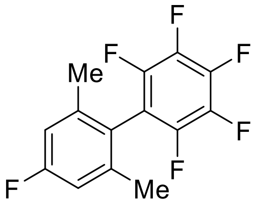

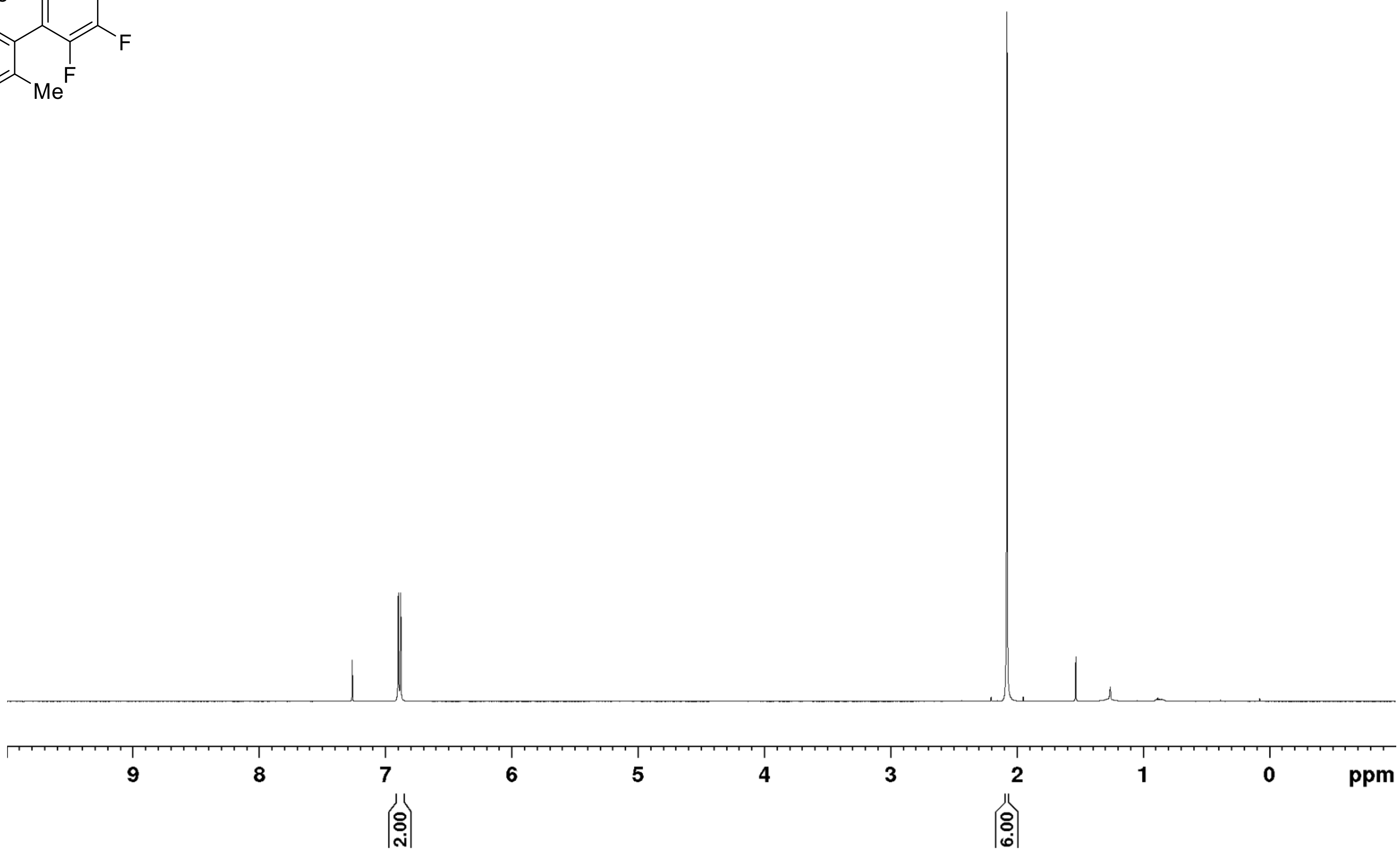

Figure S62. $^{13}C\{^{1}H\}$ NMR spectrum (126 MHz, $CDCl_3$) of 2,3,4,4',5,6-hexafluoro-2',6'-dimethyl-1,1'-biphenyl (**3ma**).

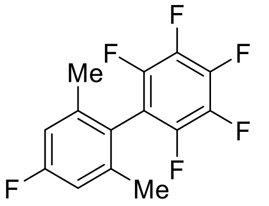

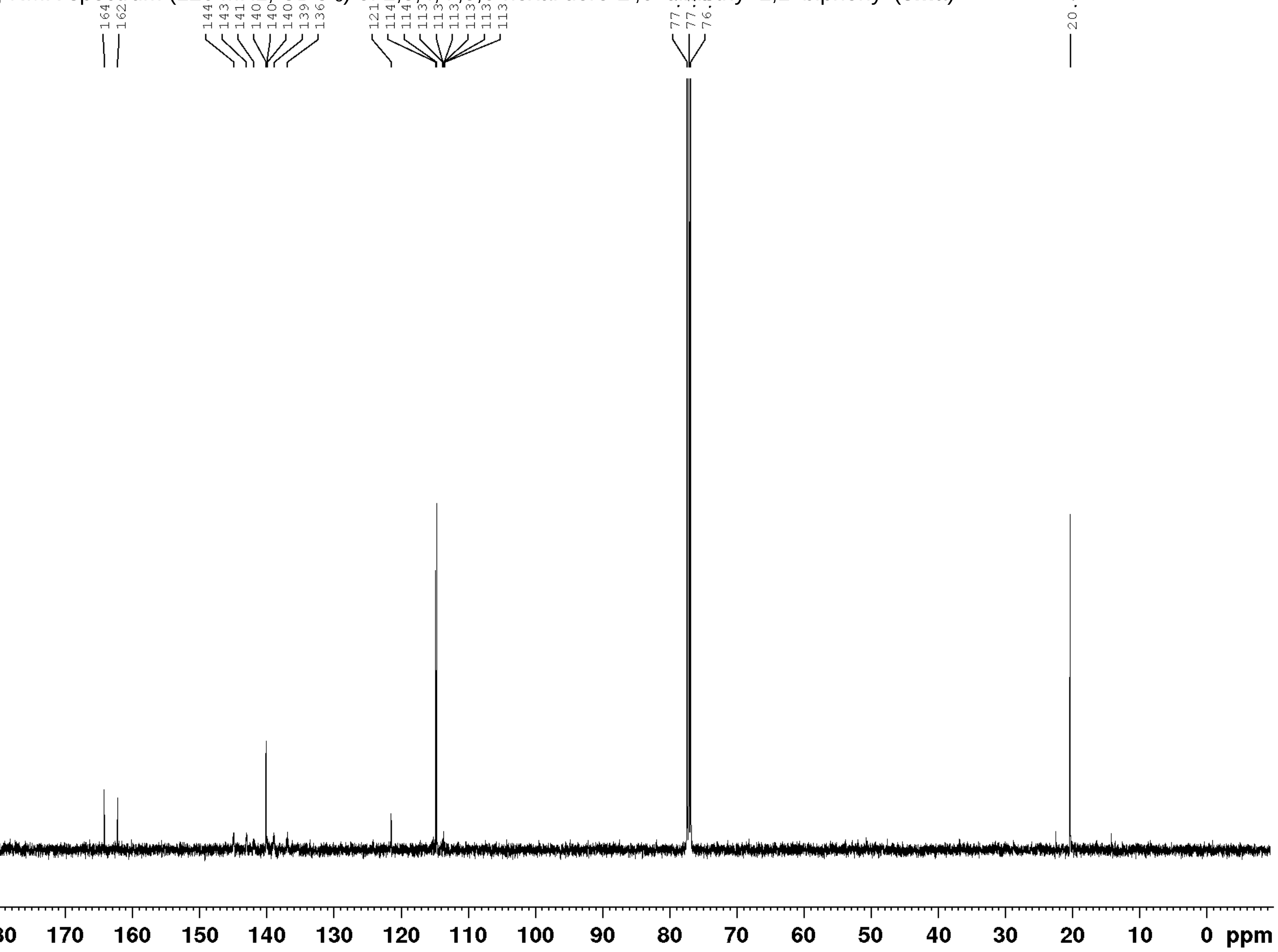

Figure S63. ^{19}F NMR spectrum (471 MHz, $CDCl_3$) of 2,3,4,4',5,6-hexafluoro-2',6'-dimethyl-1,1'-biphenyl (**3ma**).

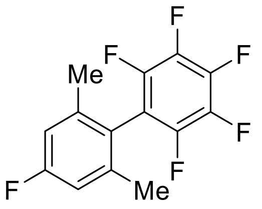

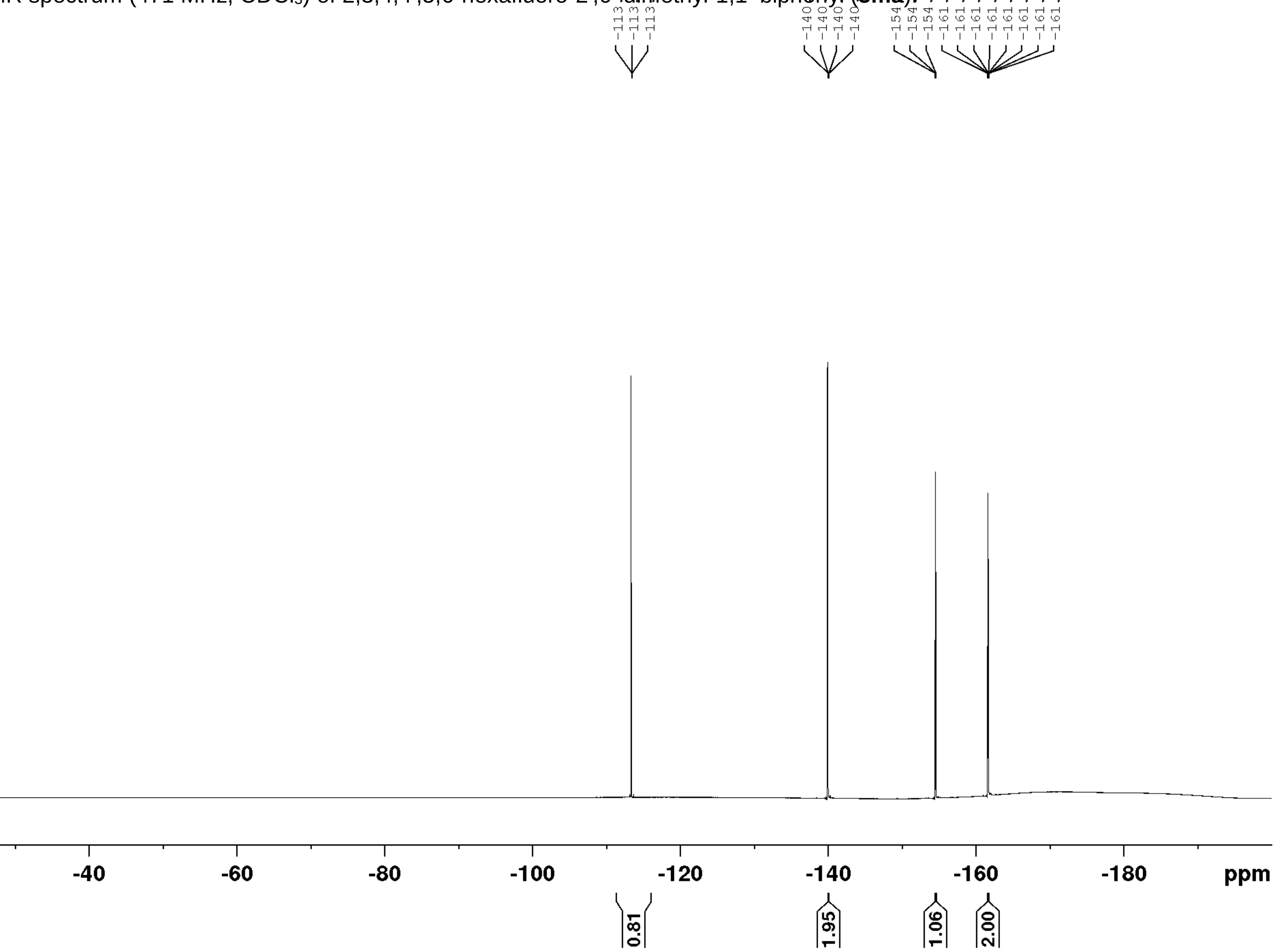

Figure S64. ^{1}H NMR spectrum (400 MHz, $CDCl_3$) of 2,3,4,5,6-pentafluoro-2',6'-dimethyl-1,1'-biphenyl (**3na**).

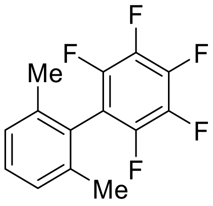

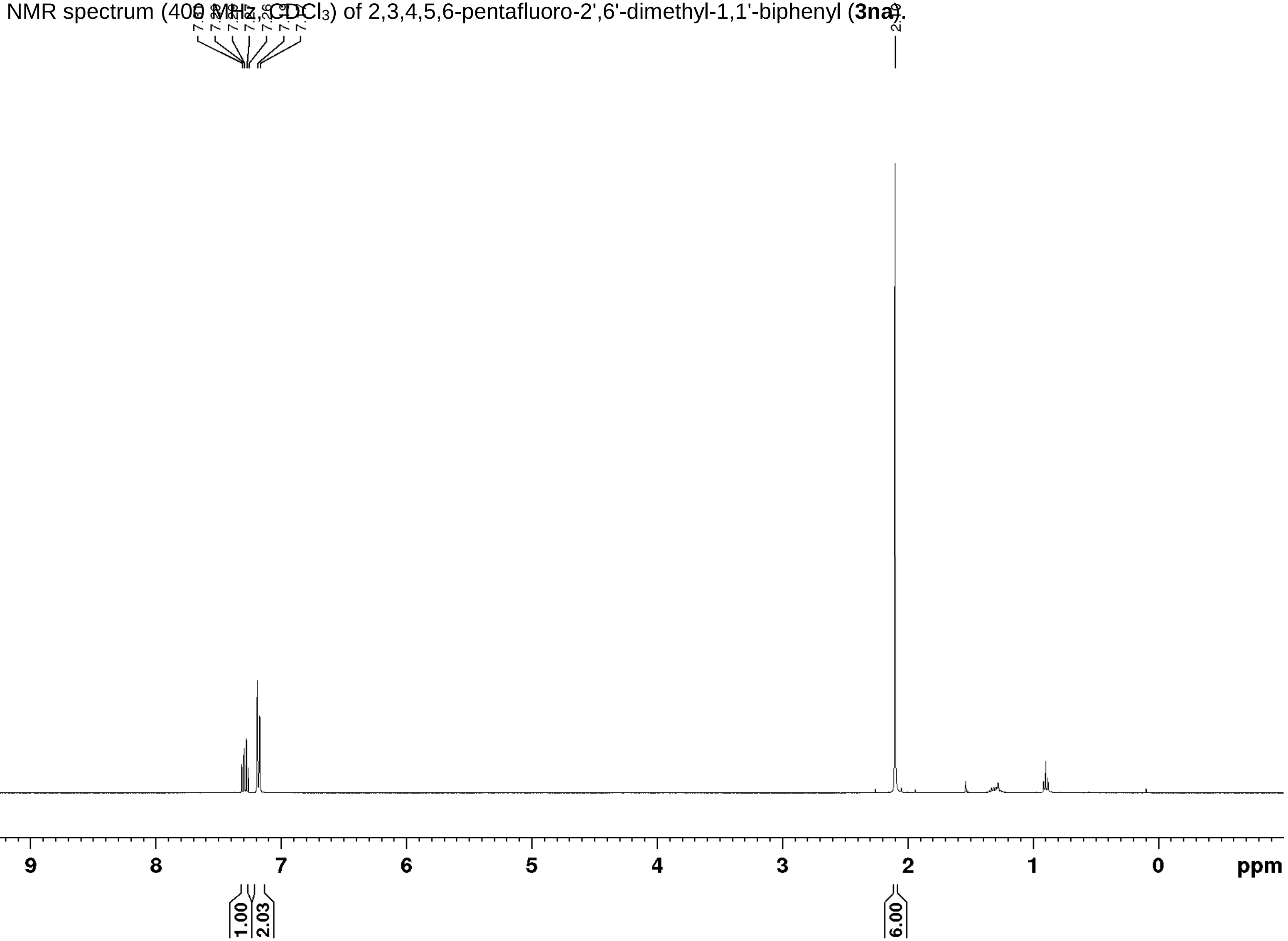

Figure S65. $^{13}C\{^{1}H\}$ NMR spectrum (101 MHz, $CDCl_3$) of 2,3,4,5,6-pentafluoro-2',6'-dimethyl-1,1'-biphenyl (**3na**).

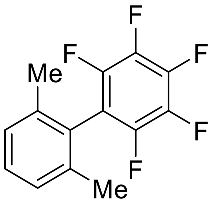

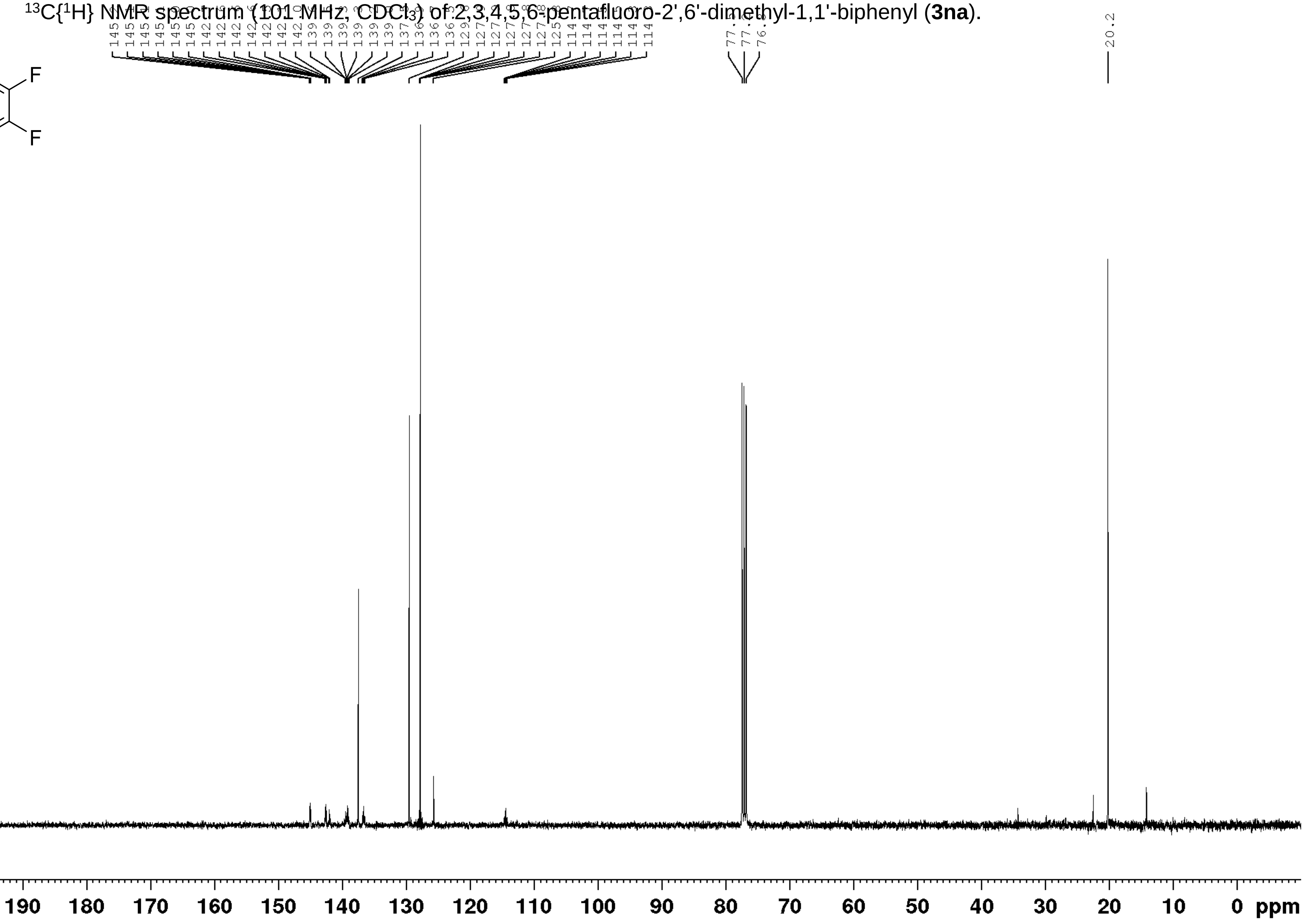

Figure S66. ^{19}F NMR spectrum (471 MHz, $CDCl_3$) of 2,3,4,5,6-pentafluoro-2',6'-dimethyl-1,1'-biphenyl (**3na**).

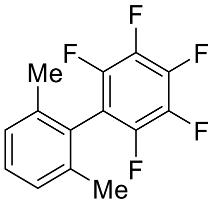

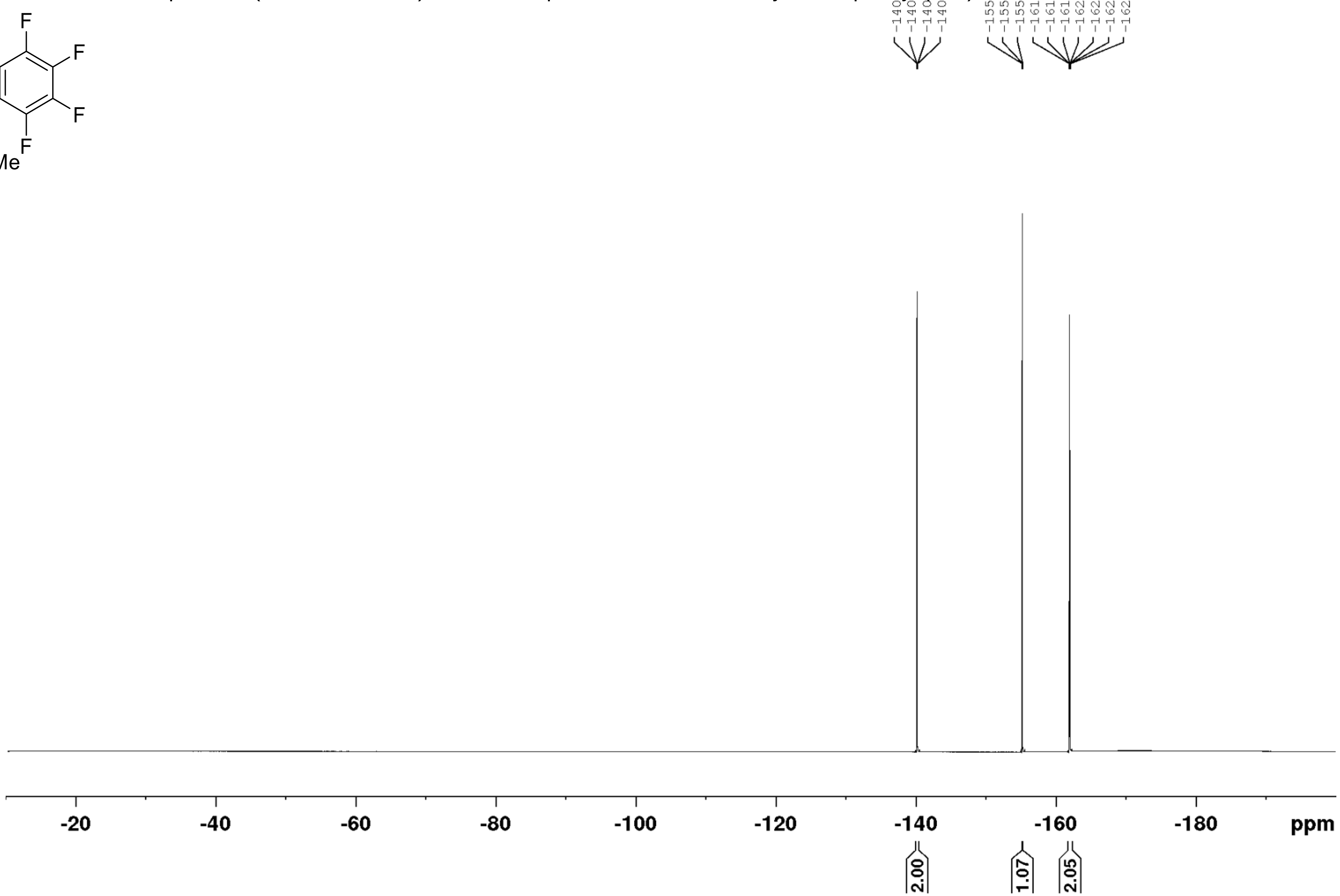

Figure S67. ^{1}H NMR spectrum (500 MHz, C_6D_6) of 2-(perfluorophenyl)naphthalene (**3oa**).

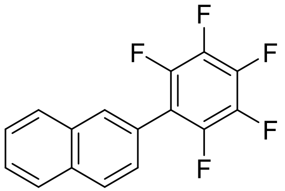

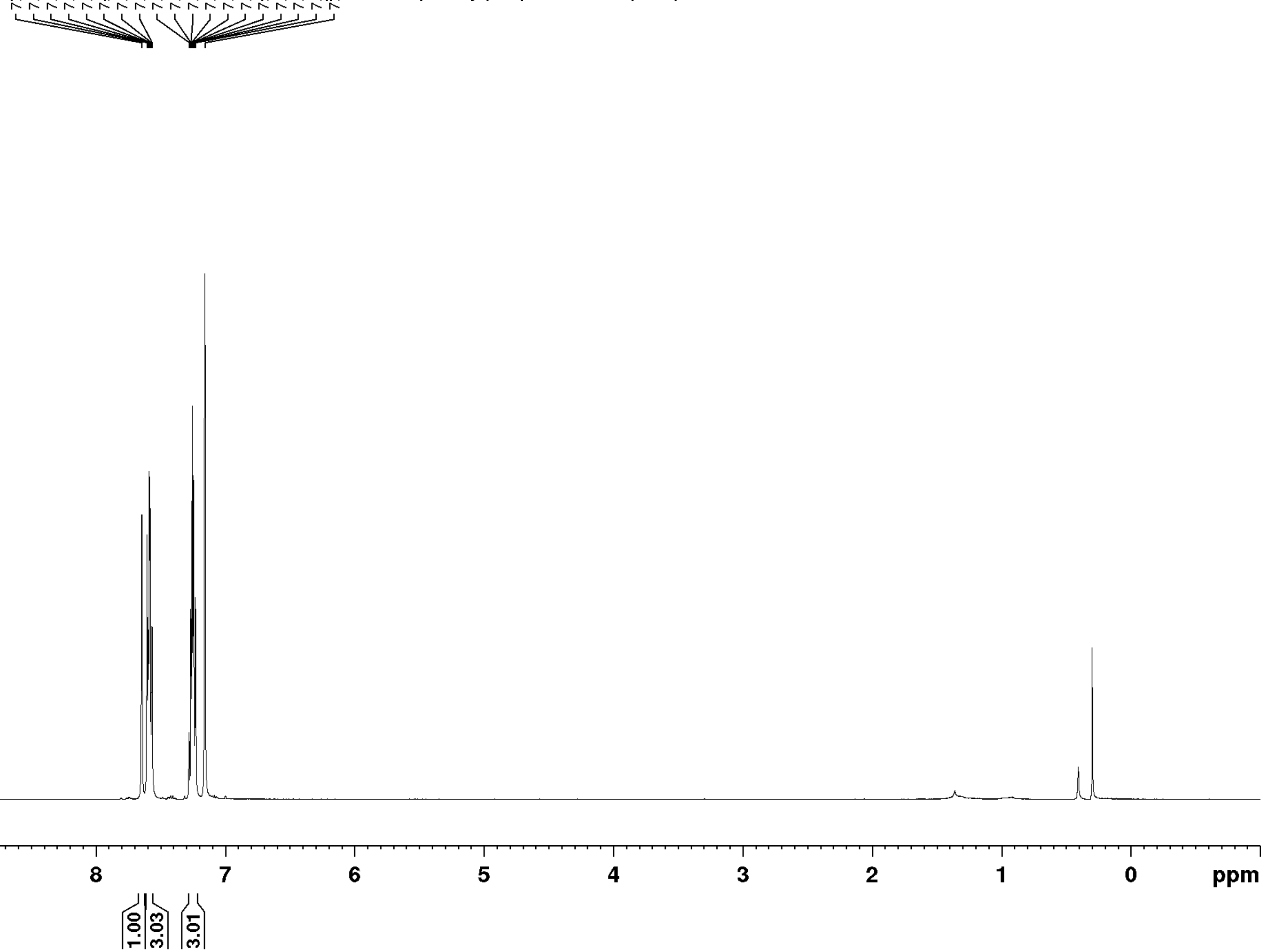

Figure S68. $^{13}C\{^{1}H\}$ NMR spectrum (101 MHz, CD_2Cl_2) of 2-(perfluorophenyl)naphthalene (**3oa**).

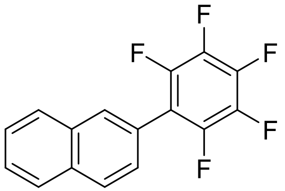

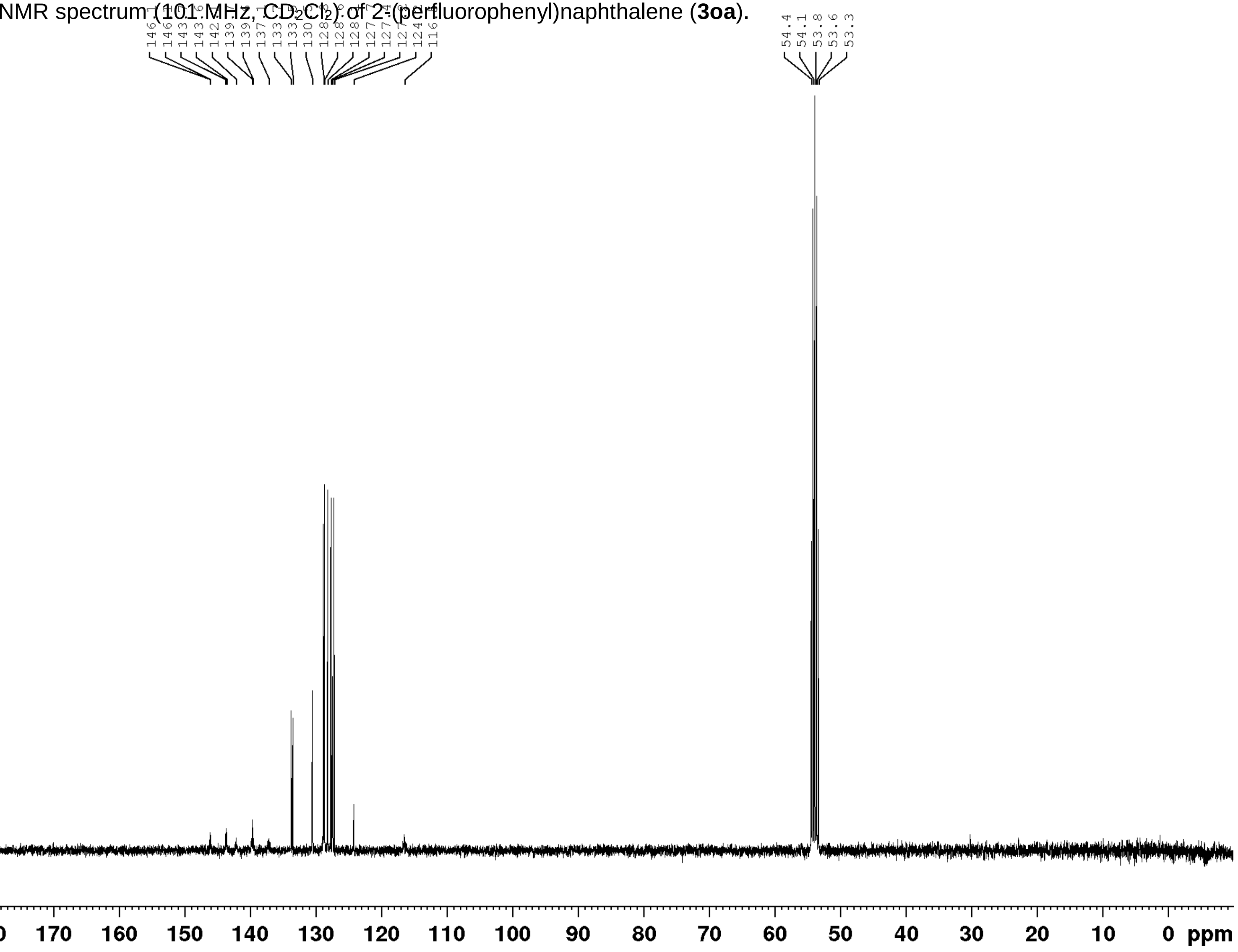

Figure S69. ^{19}F NMR spectrum (471 MHz, C_6D_6) of 2-(perfluorophenyl)naphthalene (**3oa**).

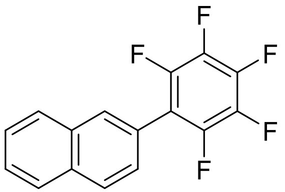

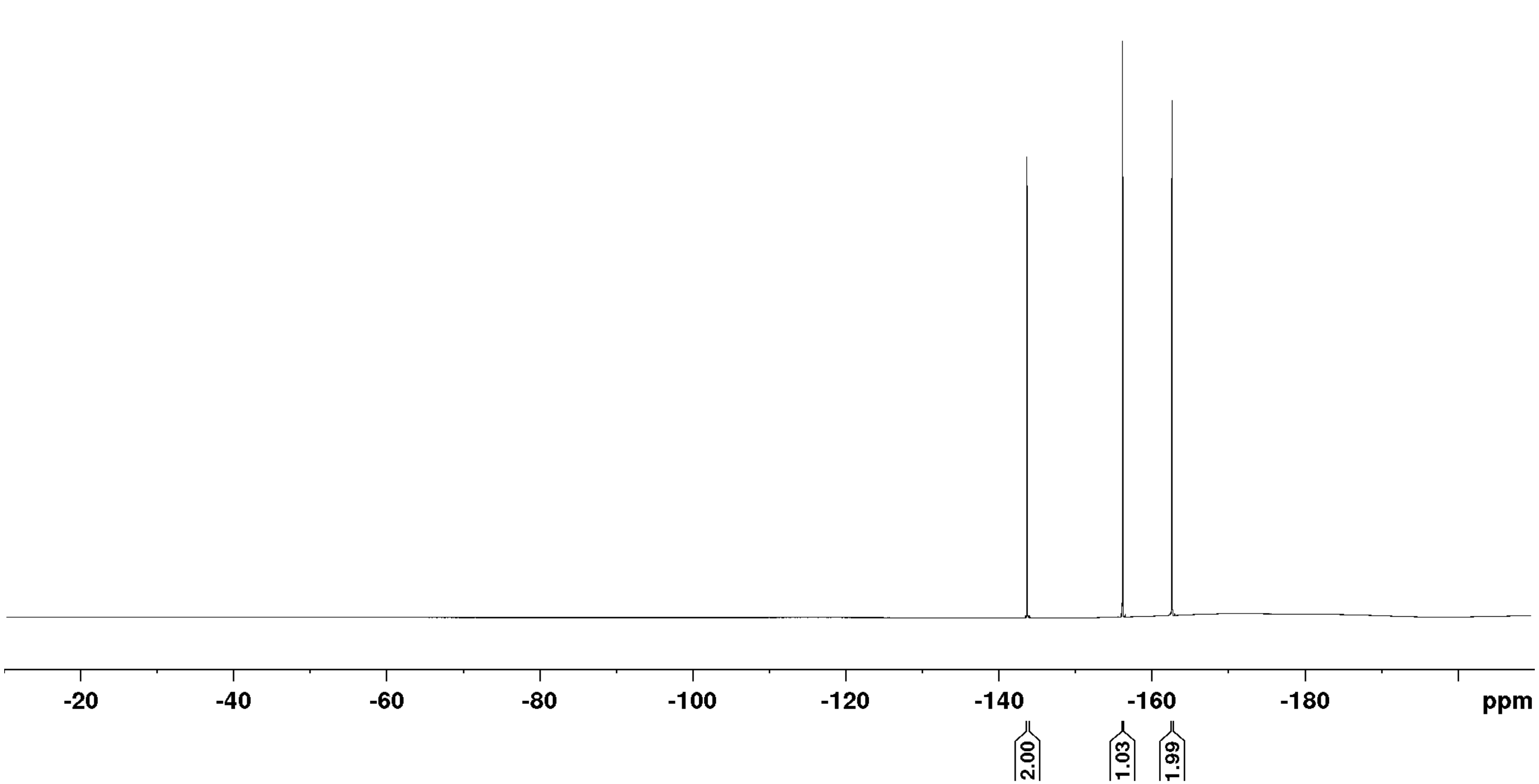

Figure S70. ^{1}H NMR spectrum (400 MHz, $CDCl_3$) of 2,2'',3,3'',4,4'',5,5'',6,6''-decafluoro-1,1':3',1''-terphenyl (**3pa**).

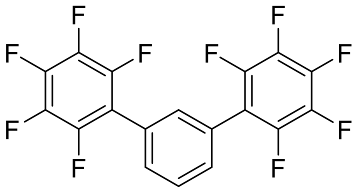

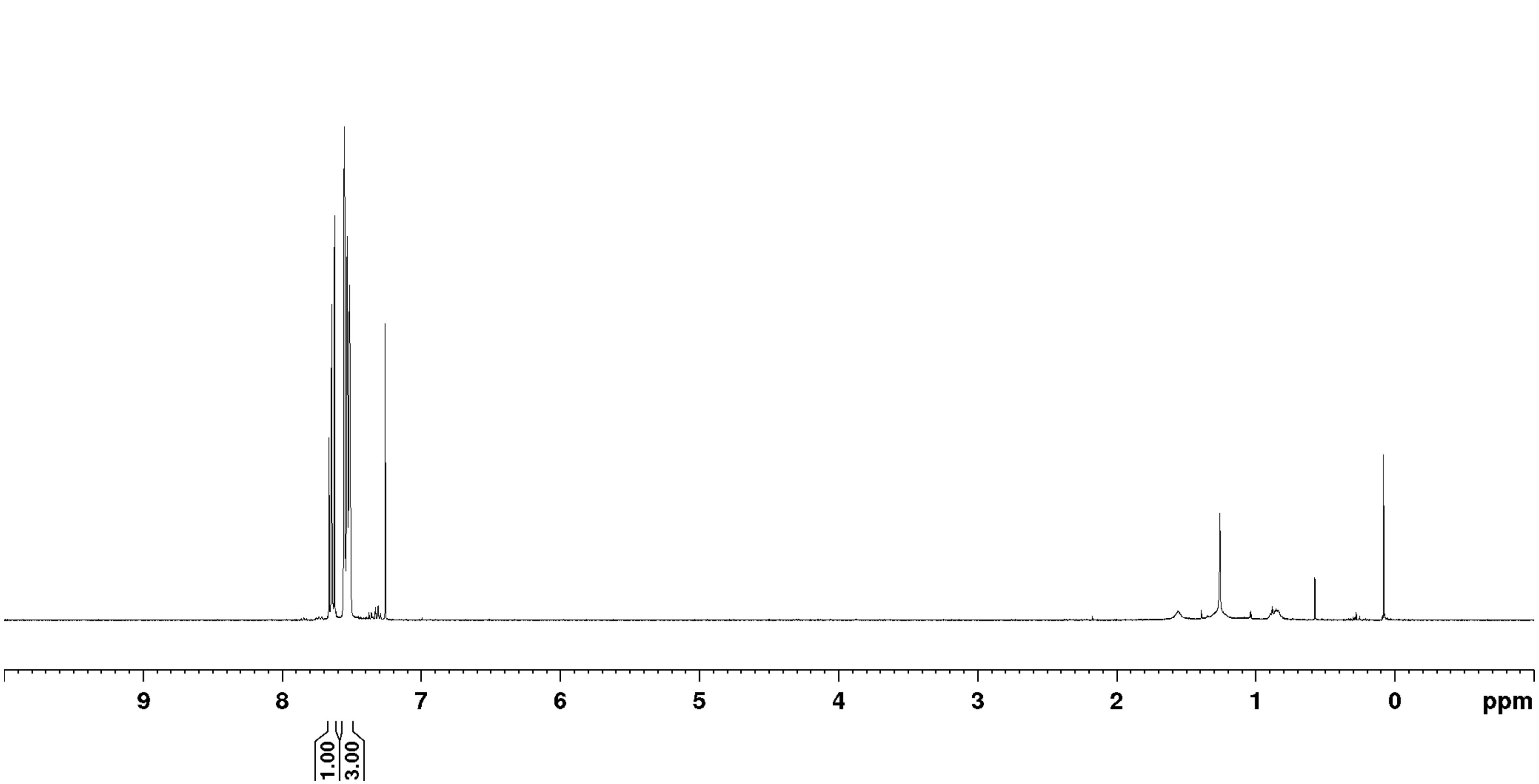

Figure S71. $^{13}C\{^1H\}$ NMR spectrum (101 MHz, $CDCl_3$) of 2,2'',3,3'',4,4'',5,5'',6,6''-decafluoro-1,1':3',1''-terphenyl (**3pa**).

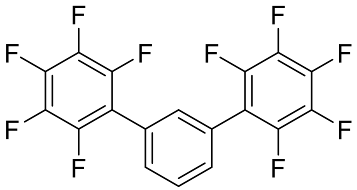

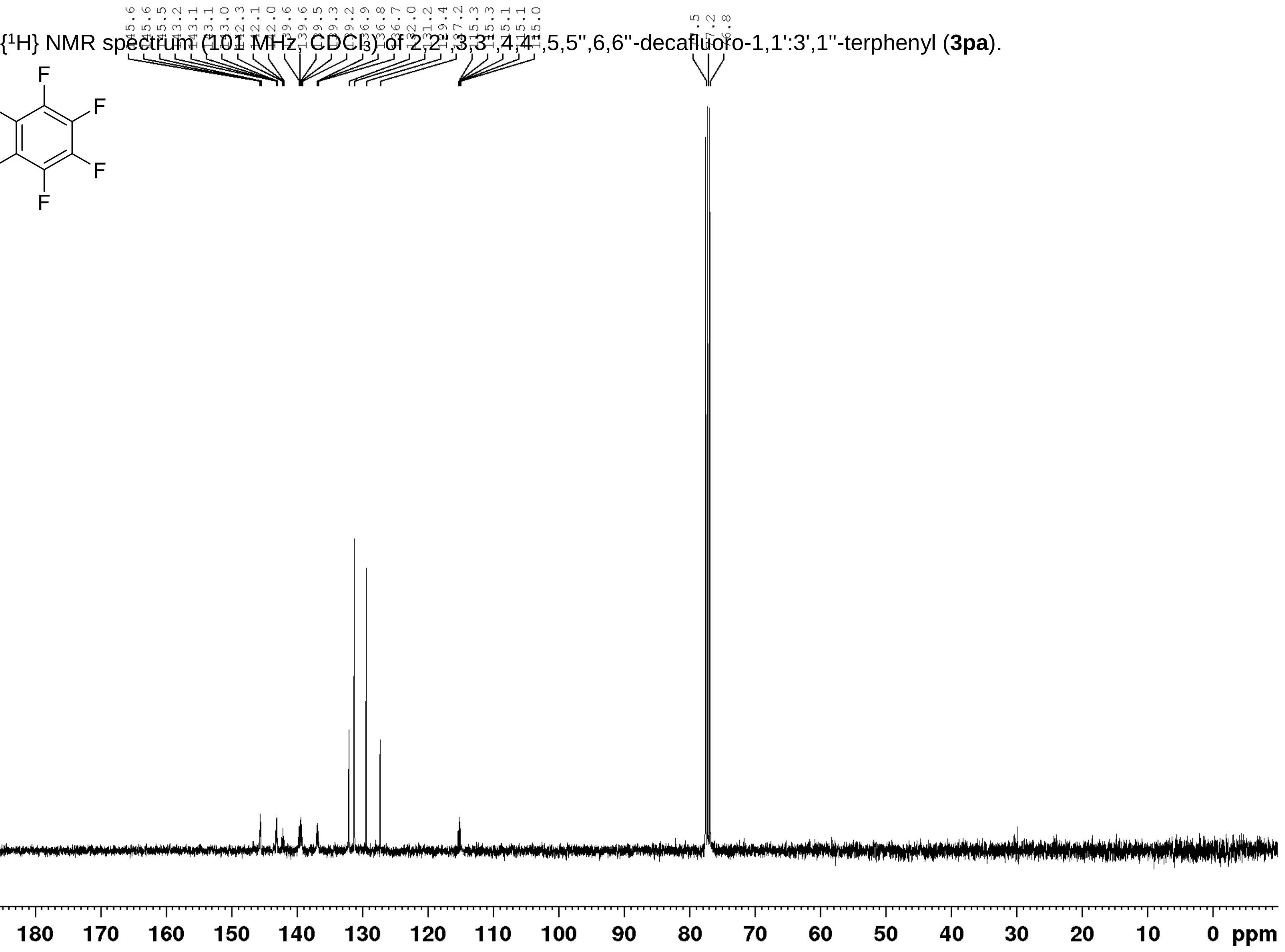

Figure S72. ^{19}F NMR spectrum (471 MHz, $CDCl_3$) of 2,2'',3,3'',4,4'',5,5'',6,6''-decafluoro-1,1':3',1''-terphenyl (**3pa**).

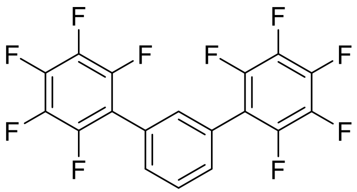

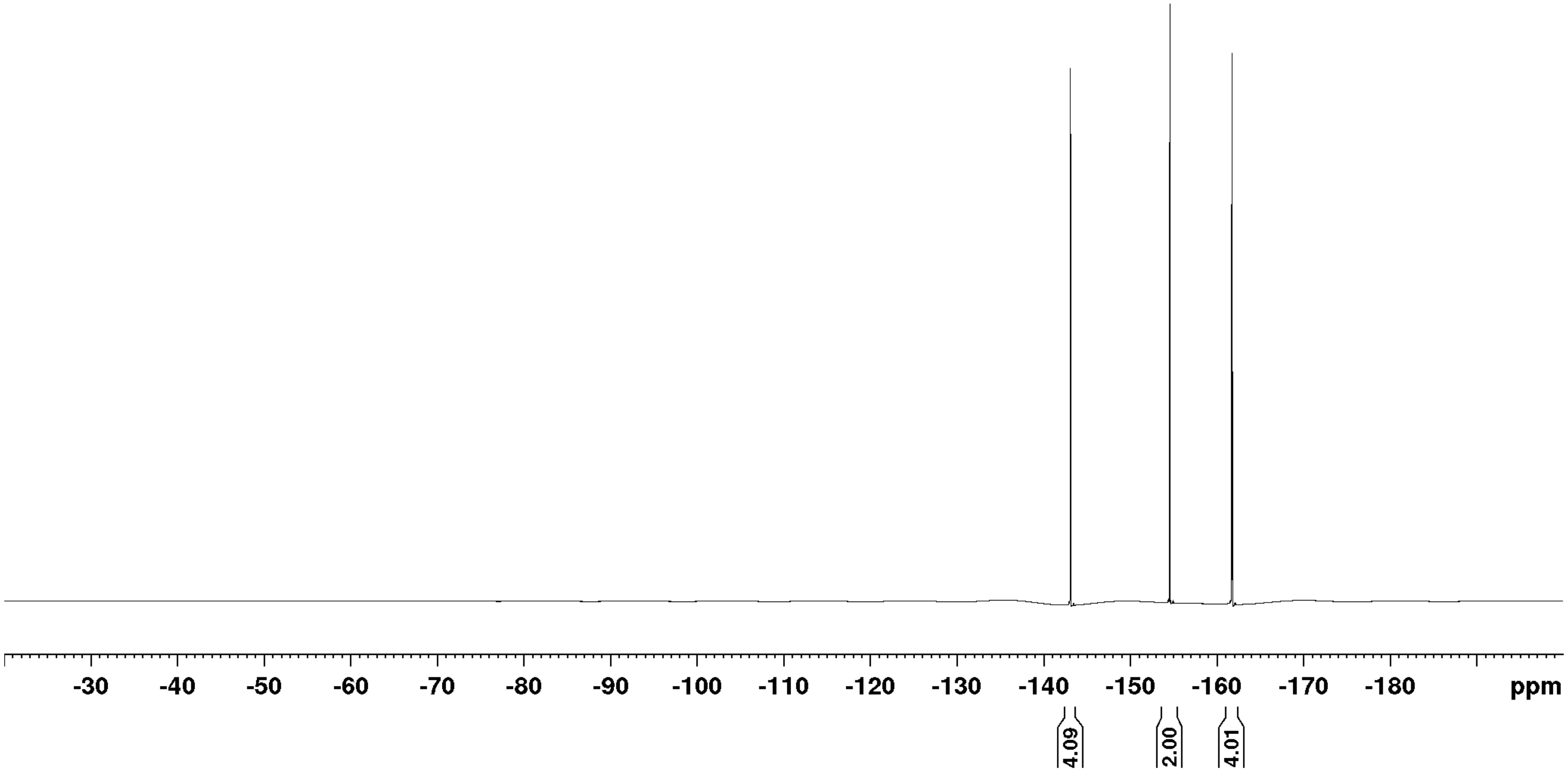

Figure S73. ^{1}H NMR spectrum (700 MHz, $CDCl_3$) of 2,3,5,6-tetrafluoro-4-(4-(trifluoromethyl)phenyl)pyridine (*para*-**3kb**).

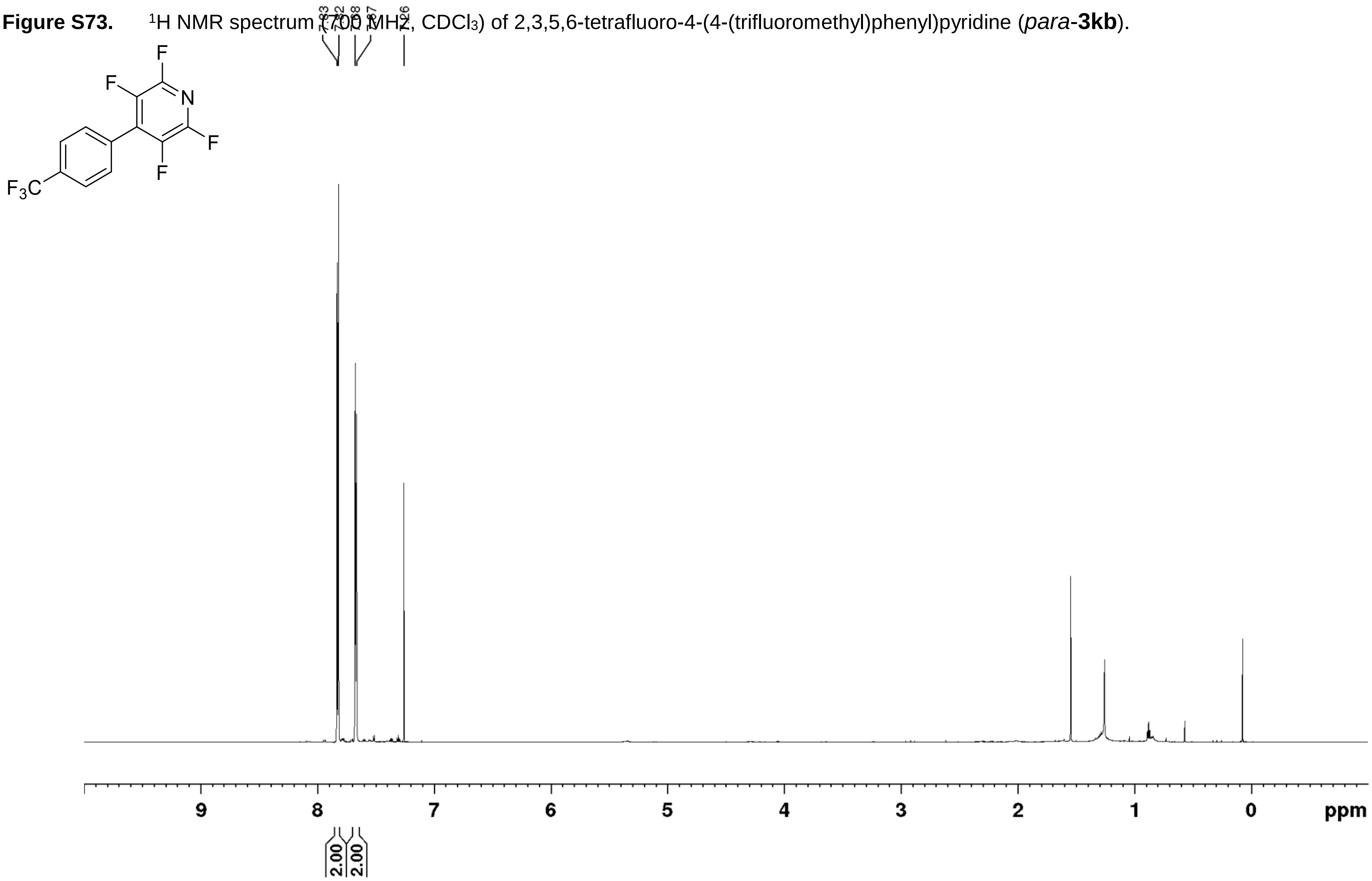

Figure S74. $^{13}C\{^{1}H\}$ NMR spectrum (176 MHz, $CDCl_3$) of 2,3,5,6-tetrafluoro-4-(4-(trifluoromethyl)phenyl)pyridine (*para*-**3kb**).

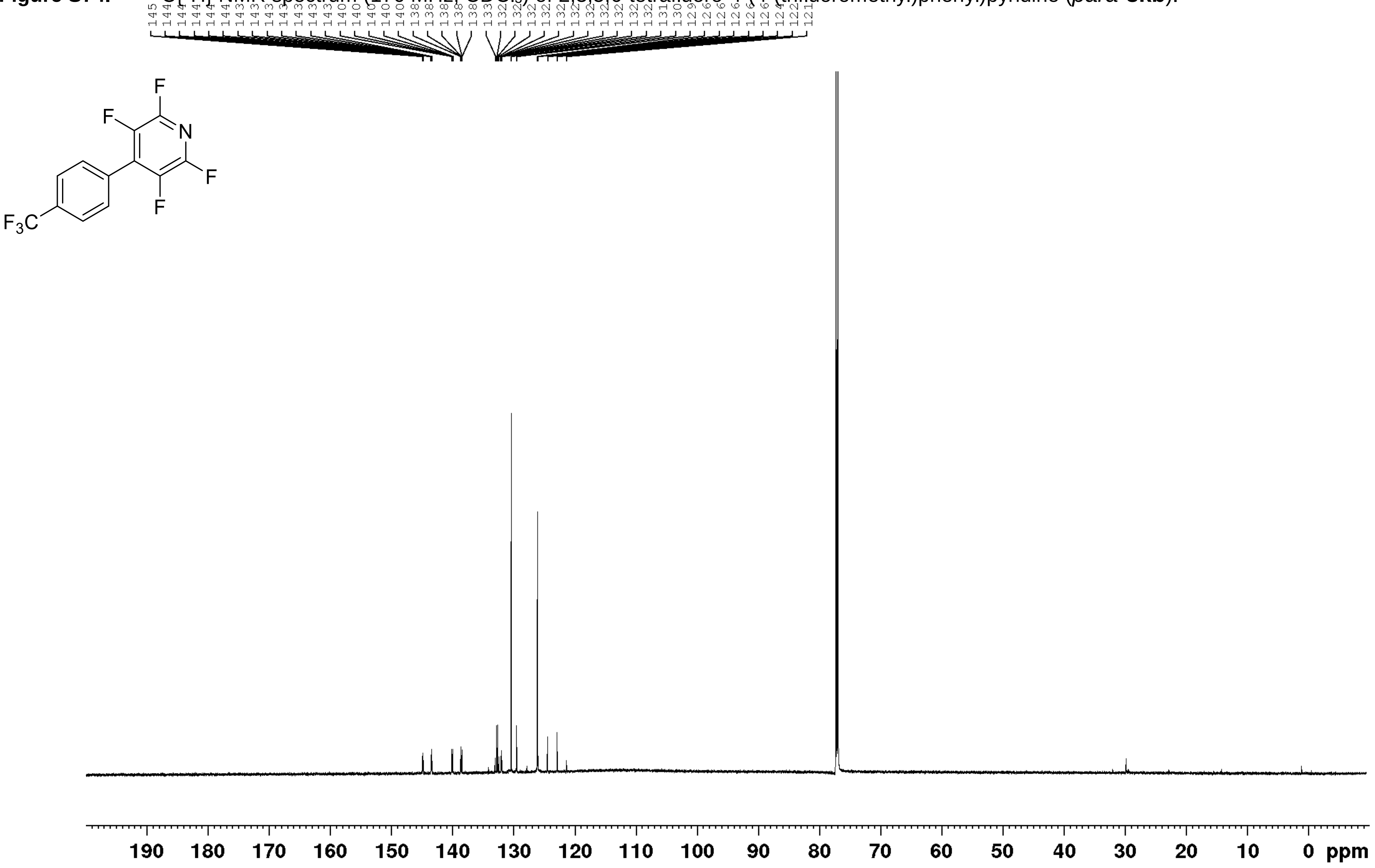

Figure S74. ^{19}F NMR spectrum (471 MHz, $CDCl_3$) of 2,3,5,6-tetrafluoro-4-(4-(trifluoromethyl)phenyl)pyridine (*para*-**3kb**).

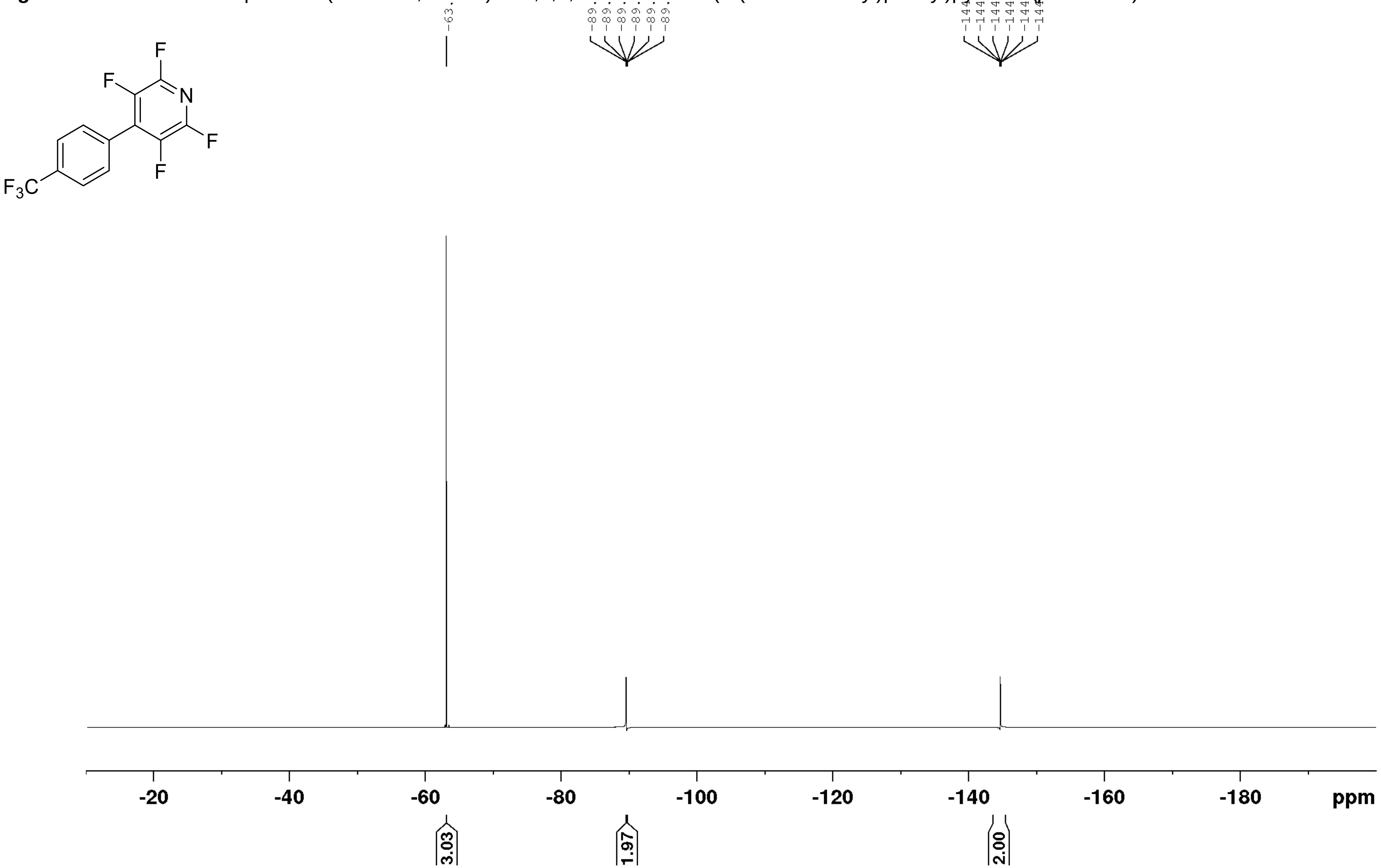

Figure S75. ^{1}H NMR spectrum (700 MHz, $CDCl_3$) of 2,3,4,5-tetrafluoro-6-(4-(trifluoromethyl)phenyl)pyridine (*ortho*-**3kb**).

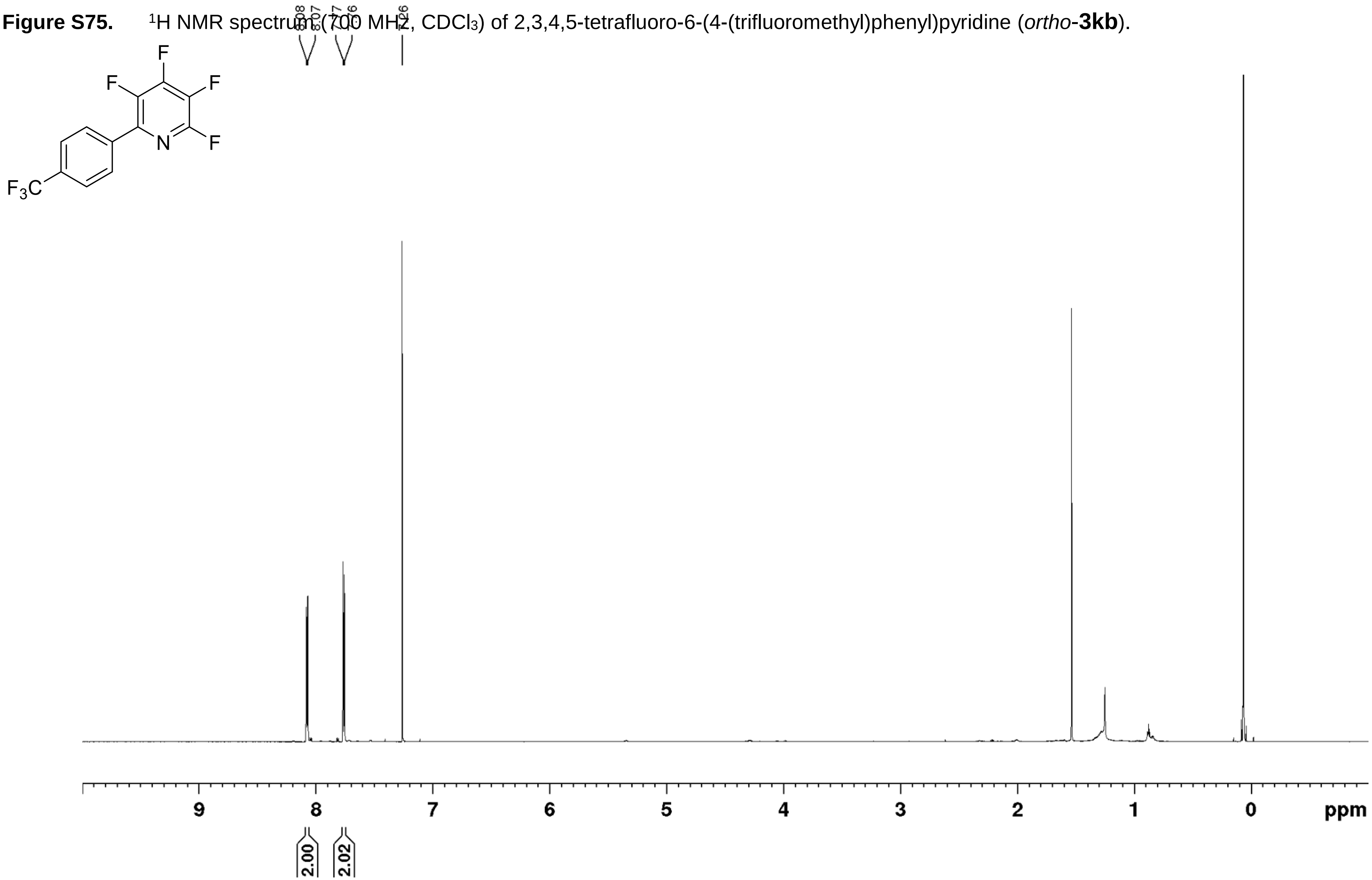

Figure S76. $^{13}C\{^{1}H\}$ NMR spectrum (176 MHz, $CDCl_3$) of 2,3,4,5-tetrafluoro-6-(4-(trifluoromethyl)phenyl)pyridine (*ortho*-**3kb**).

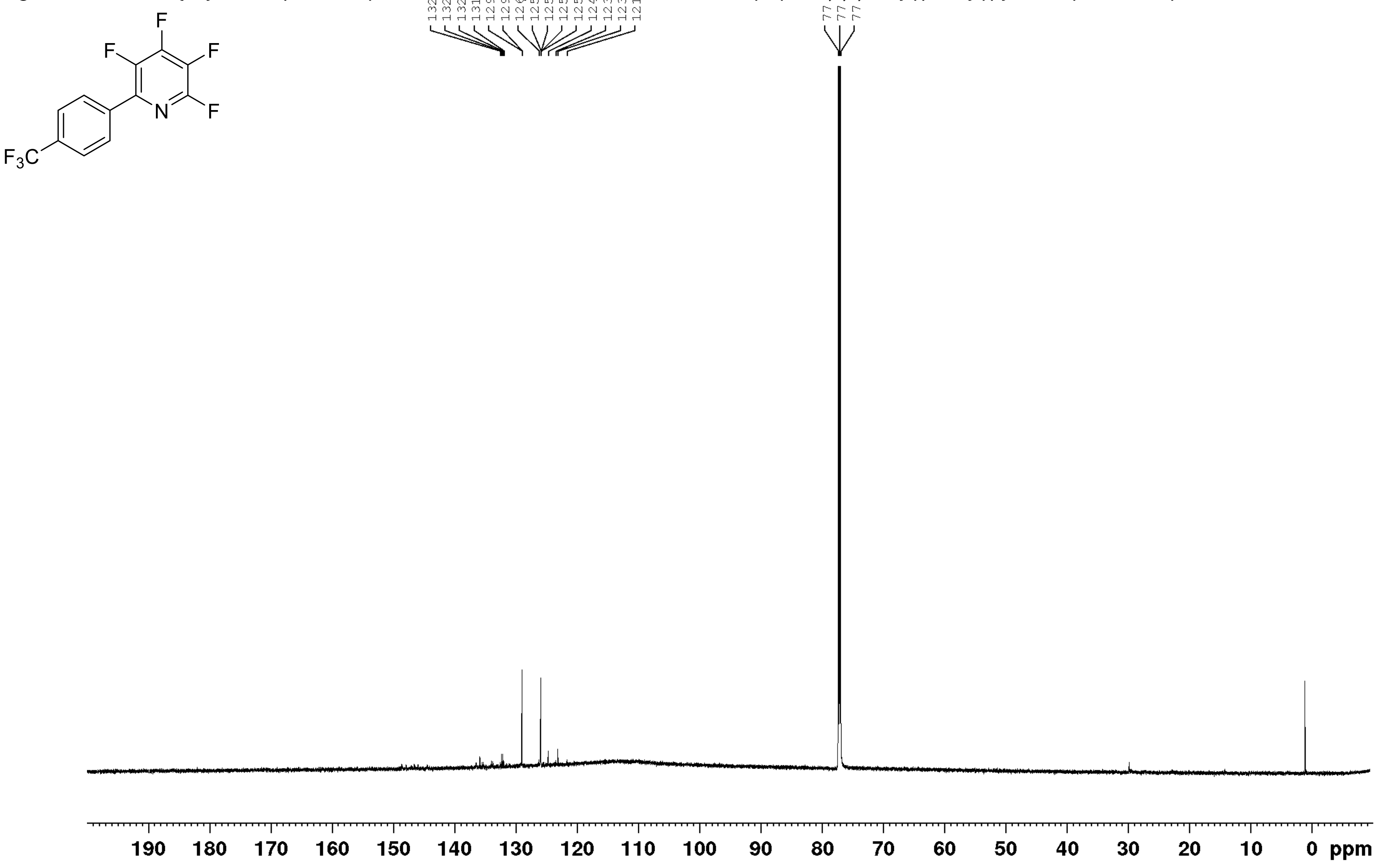

Figure S77. ^{19}F NMR spectrum (471 MHz, $CDCl_3$) of 2,3,4,5-tetrafluoro-6-(4-(trifluoromethyl)phenyl)pyridine (*ortho*-**3kb**).

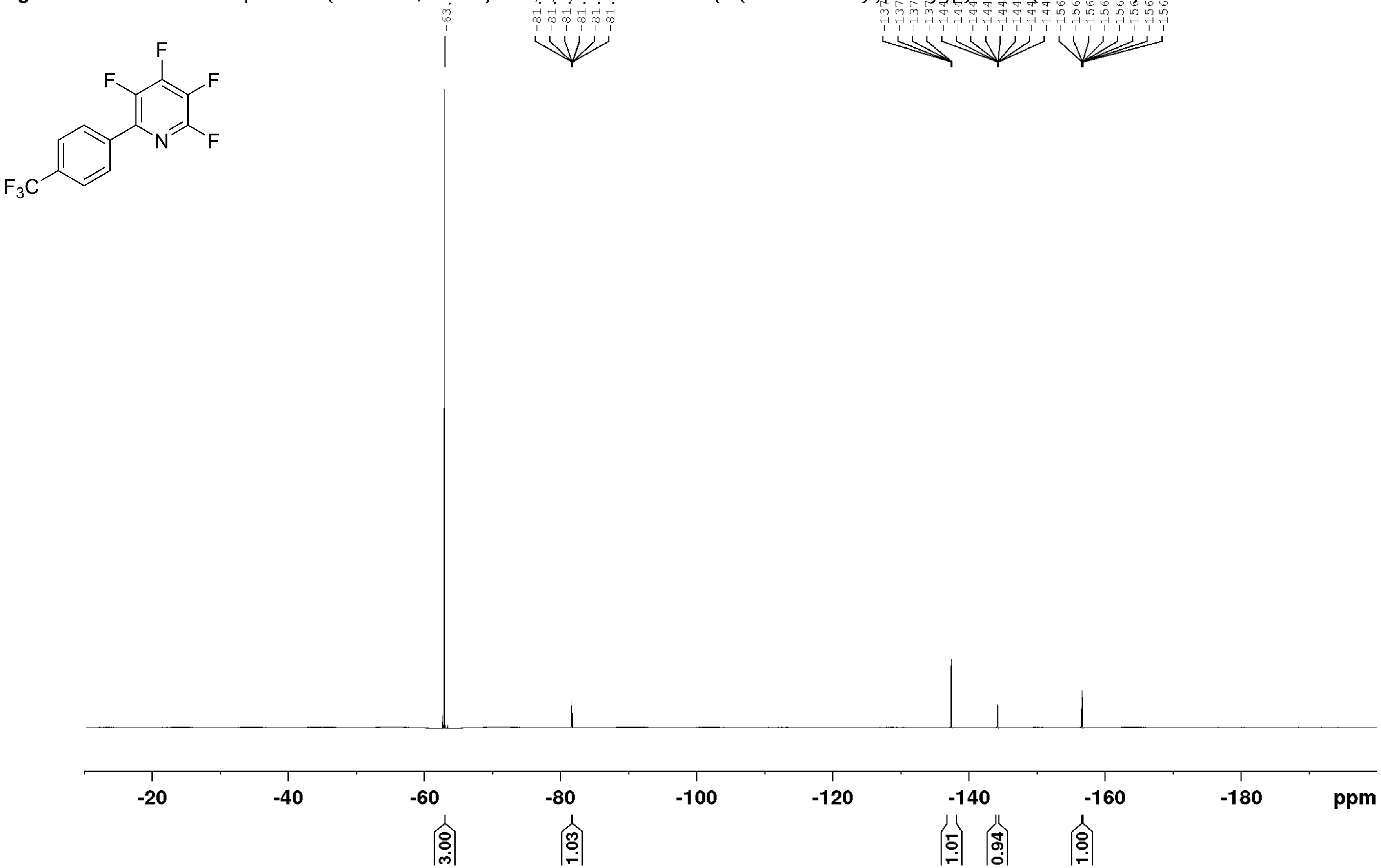

Figure S78. ^{1}H NMR spectrum (700 MHz, $CDCl_3$) of 2,3,5,6-tetrafluoro-4'-methoxy-4-vinyl-1,1'-biphenyl (**3ck**).

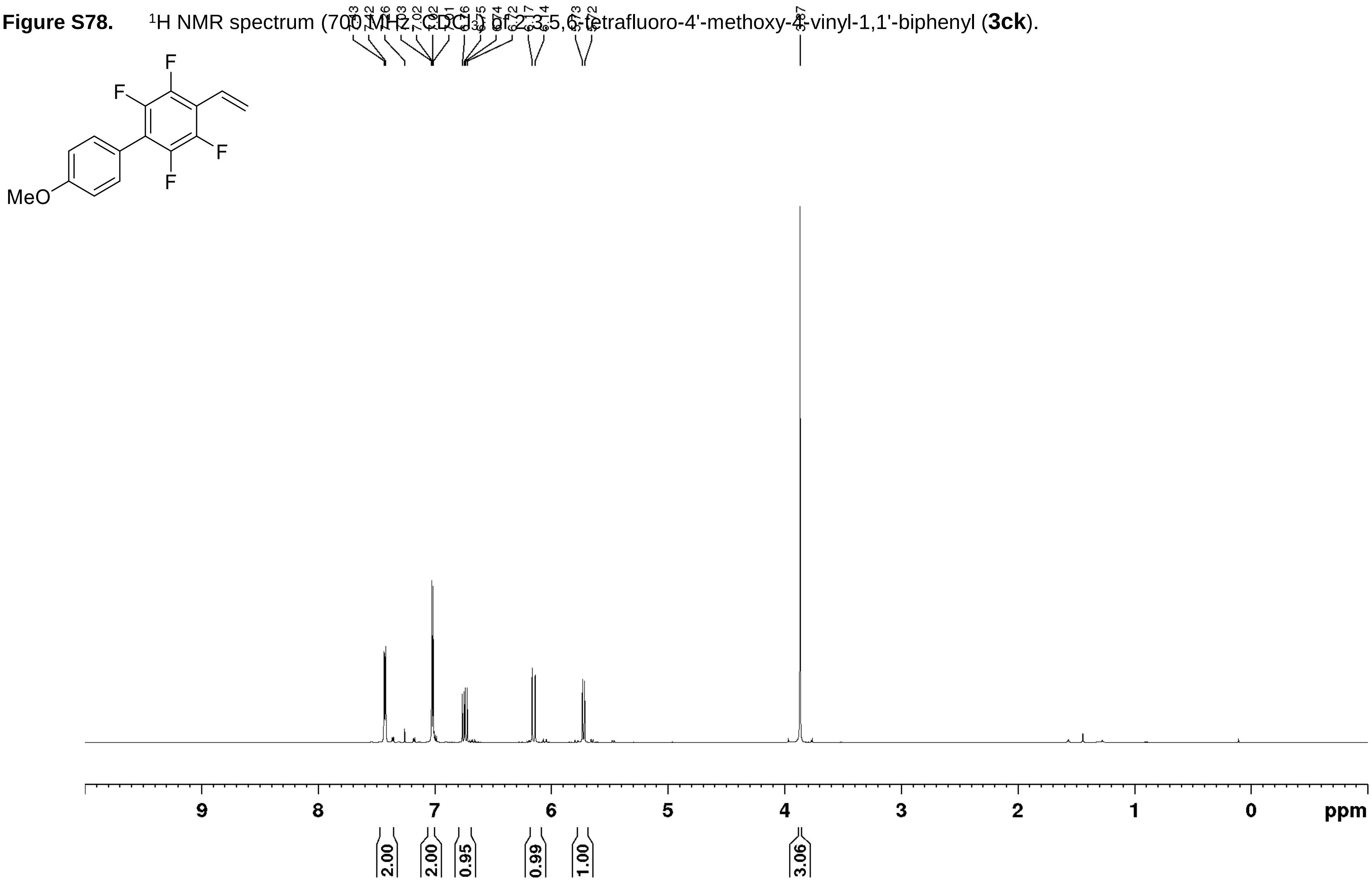

Figure S79. $^{13}C\{^{1}H\}$ NMR spectrum (176 MHz, $CDCl_3$) of 2,3,5,6-tetrafluoro-4''-methoxy-4-vinyl-1,1'-biphenyl (**3ck**).

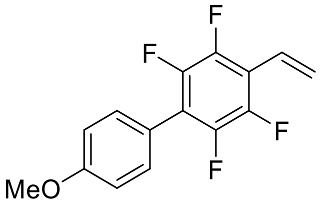

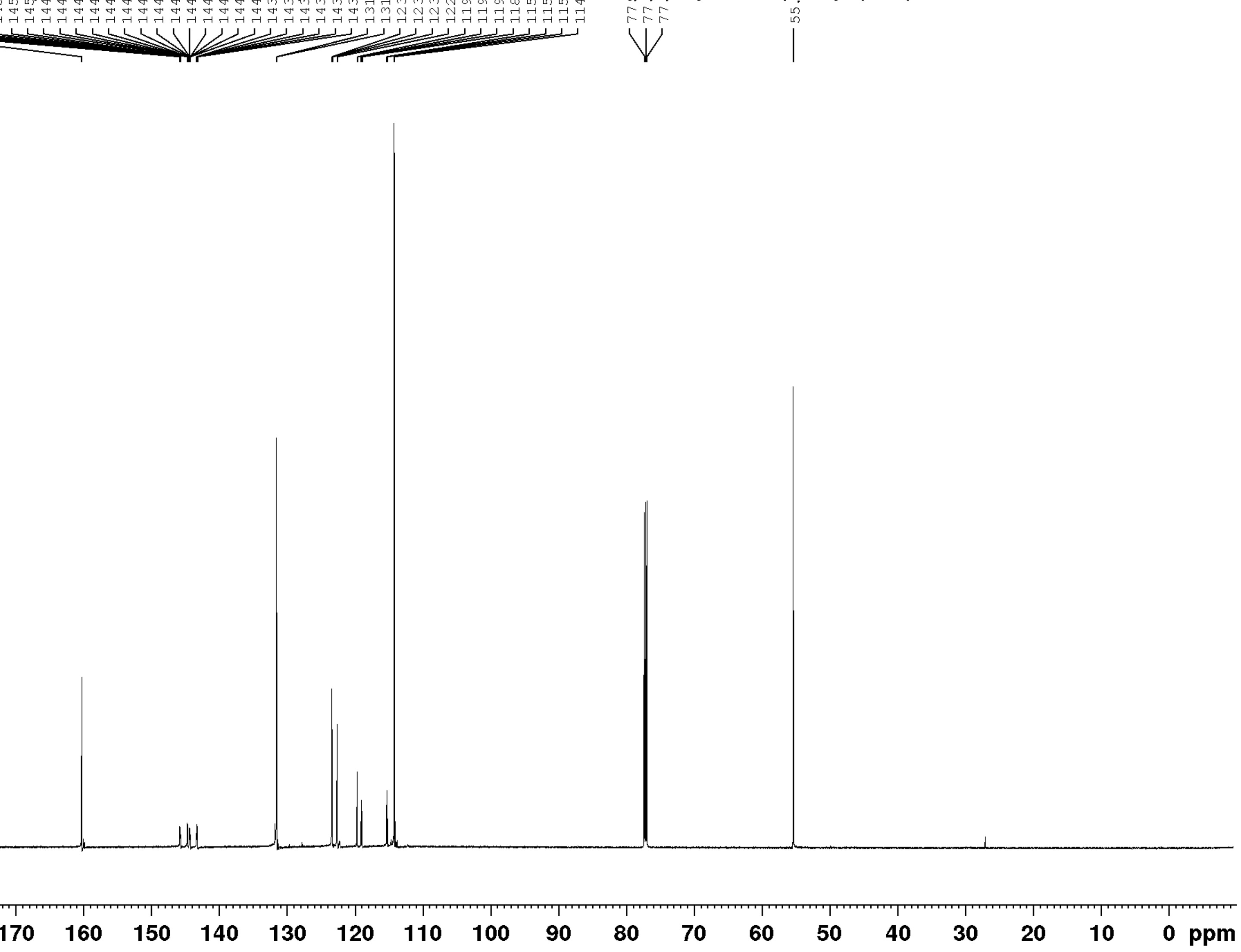

Figure S80. ^{19}F NMR spectrum (659 MHz, $CDCl_3$) of 2,3,5,6-tetrafluoro-4'-methoxy-4-vinyl-1,1'-biphenyl (**3ck**).

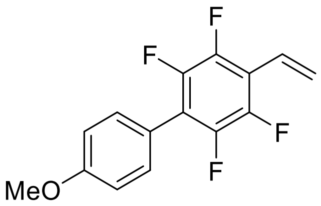

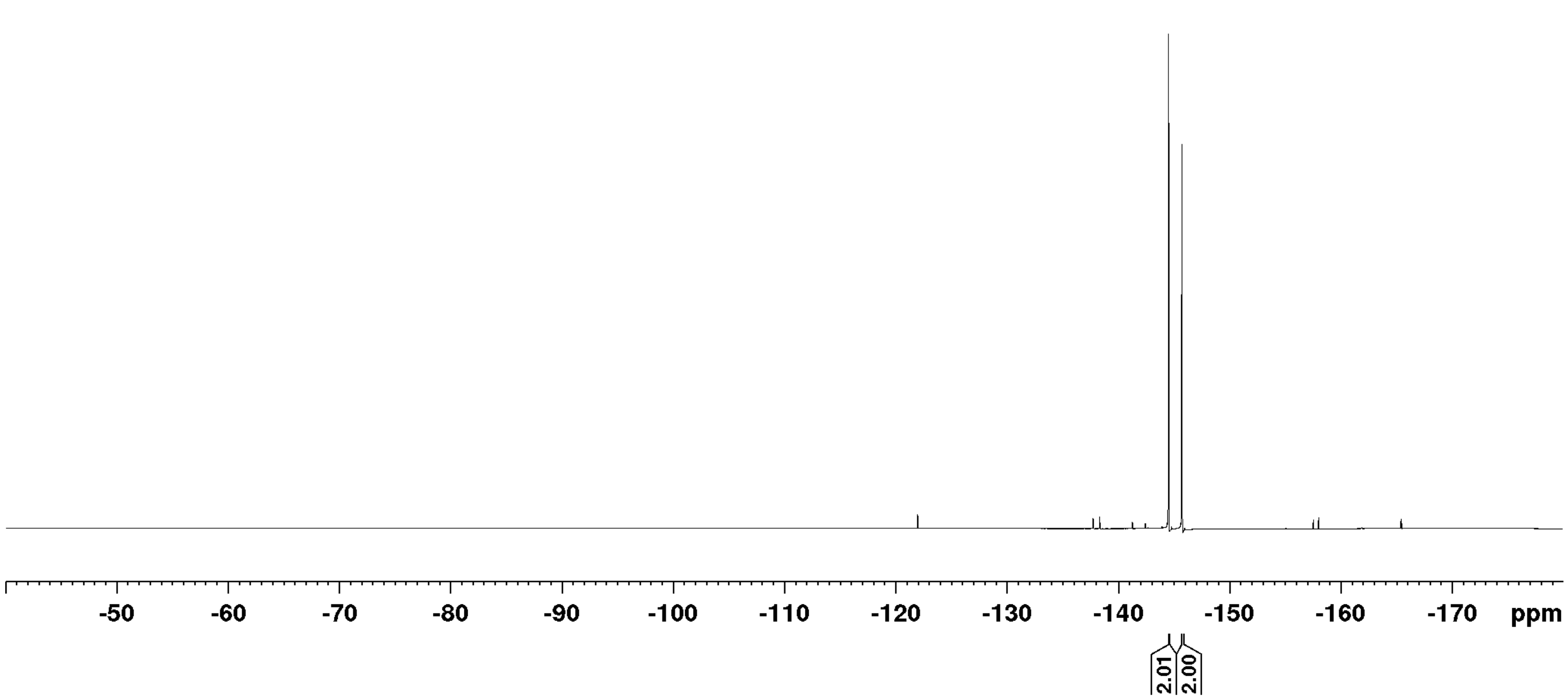

Figure S81. ^{1}H NMR spectrum (500 MHz, $CDCl_3$) of 2',3',5',6'-tetrafluoro-4'-vinyl-[1,1'-biphenyl]-4-carbonitrile (**3dk**).

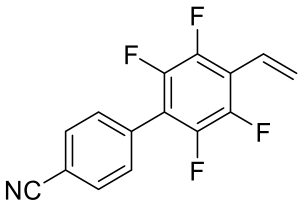

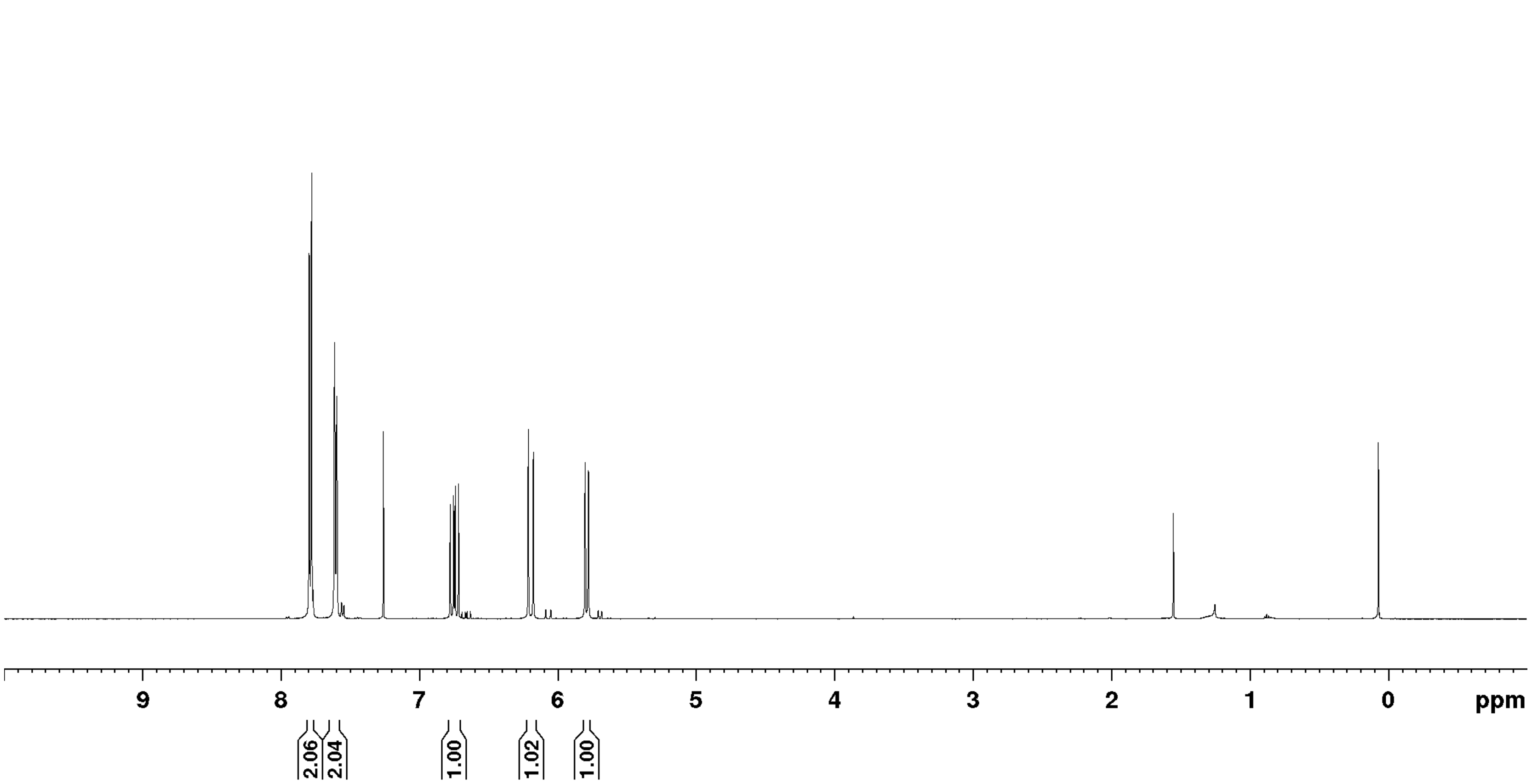

Figure S82. $^{13}C\{^{1}H\}$ NMR spectrum (126 MHz, $CDCl_3$) of 2',3',5',6'-tetrafluoro-4'-vinyl-[1,1'-biphenyl]-4-carbonitrile (**3dk**).

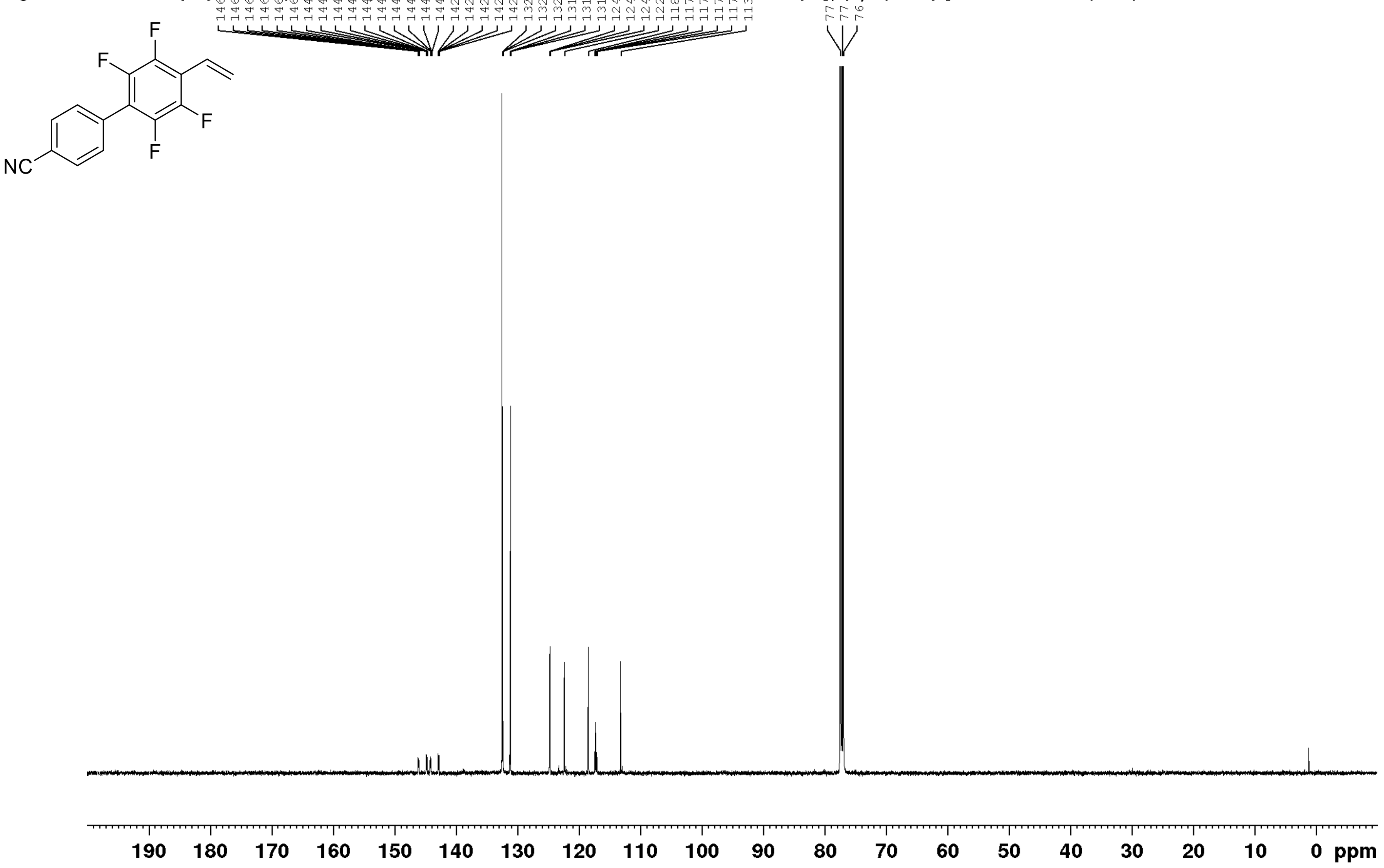

Figure S83. ^{19}F NMR spectrum (471 MHz, $CDCl_3$) of 2',3',5',6'-tetrafluoro-4'-vinyl-[1,1'-biphenyl]-4-carbonitrile (**3dk**).

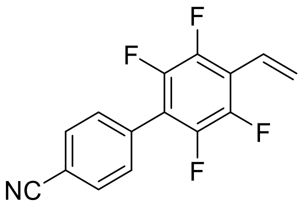

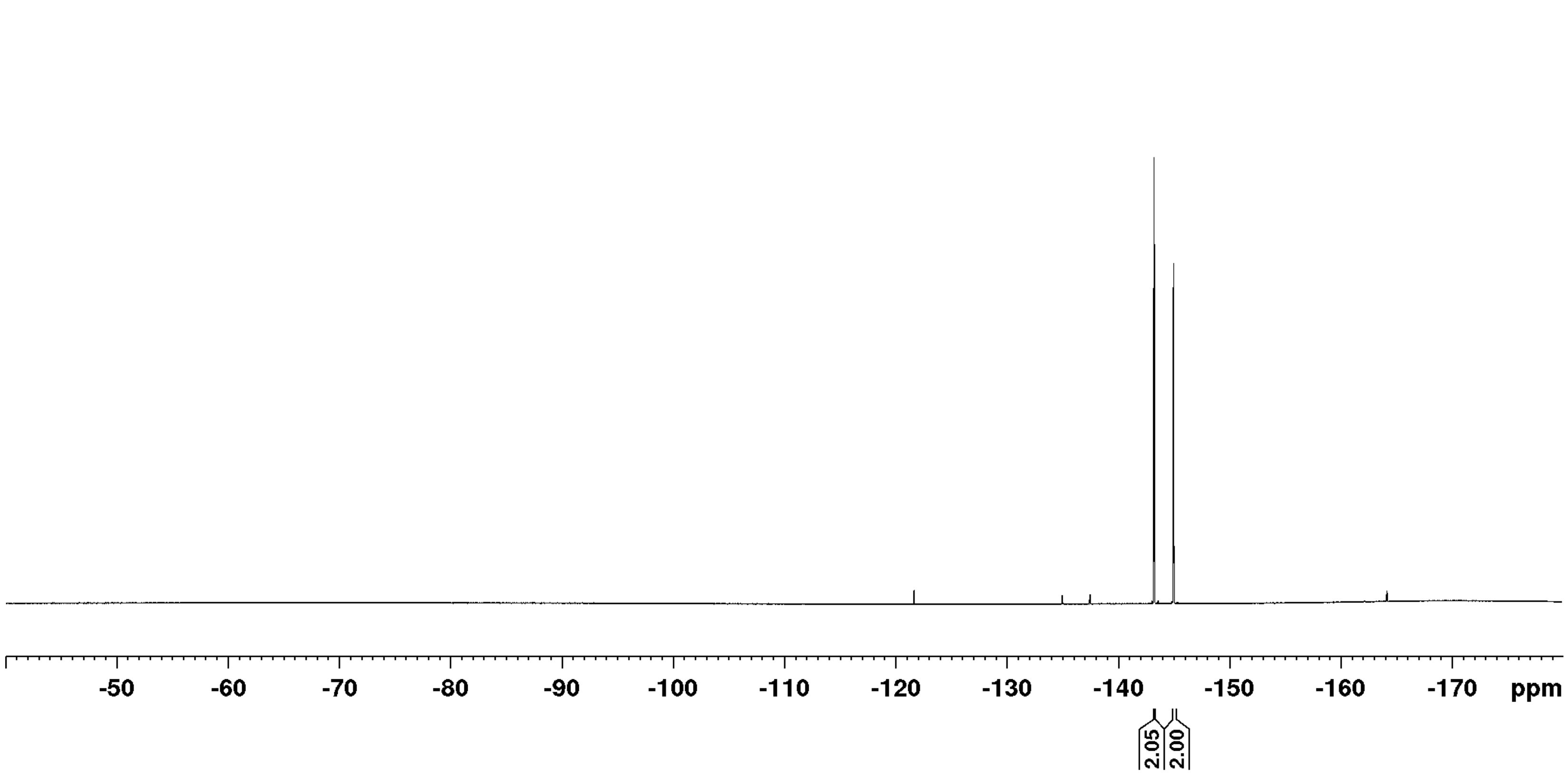

Figure S84. ^1H NMR spectrum (500 MHz, $CDCl_3$) of (4'-chloro-2,3,5,6-tetrafluoro-[1,1'-biphenyl]-4-yl)(phenyl)methanone (**3ie**).

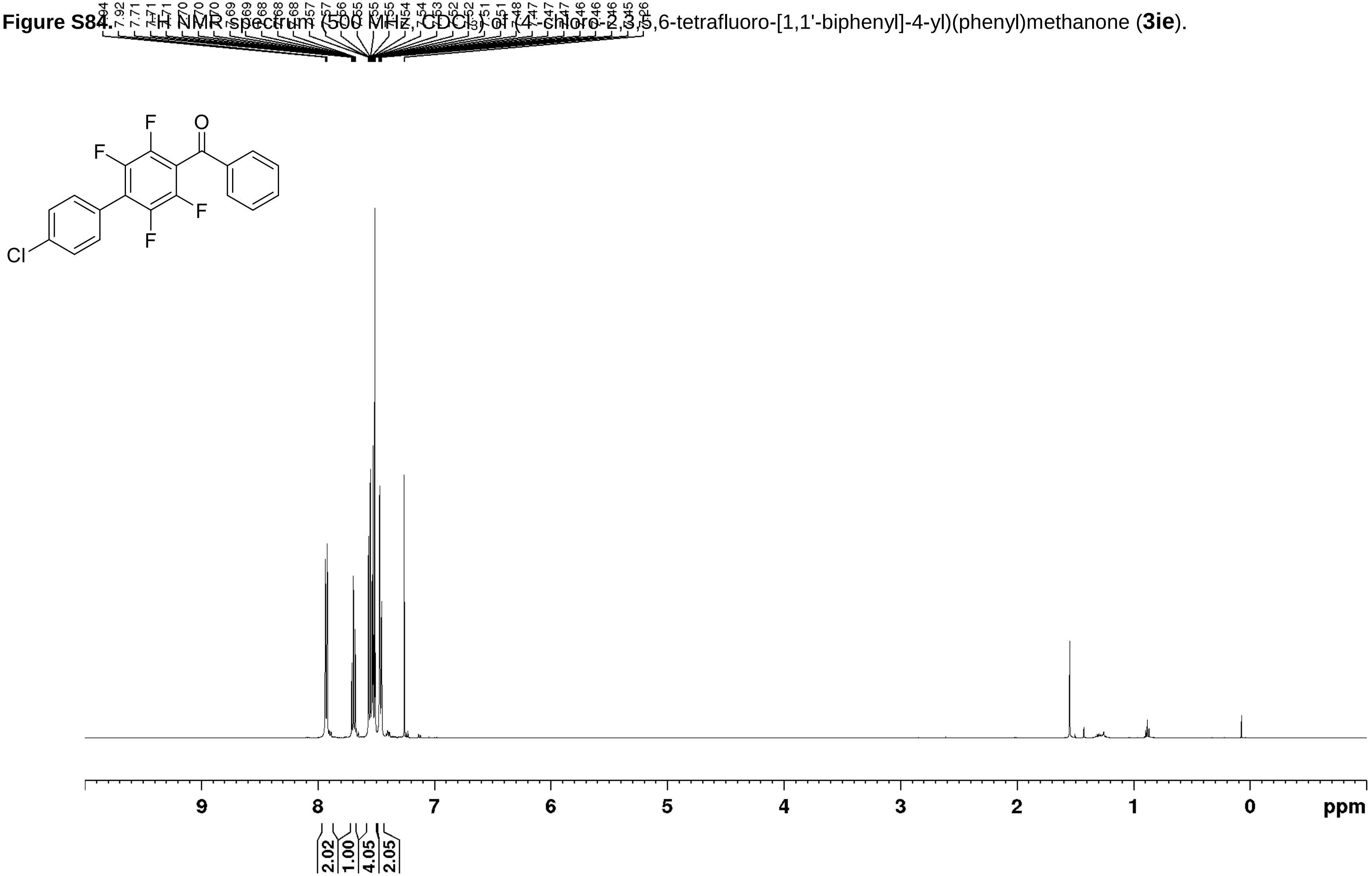

Figure S85. $^{13}C\{^1H\}$ NMR spectrum (126 MHz, $CDCl_3$) of (4'-chloro-2,3,5,6-tetrafluoro-[1,1'-biphenyl]-4-yl)(phenyl)methanone (**3ie**).

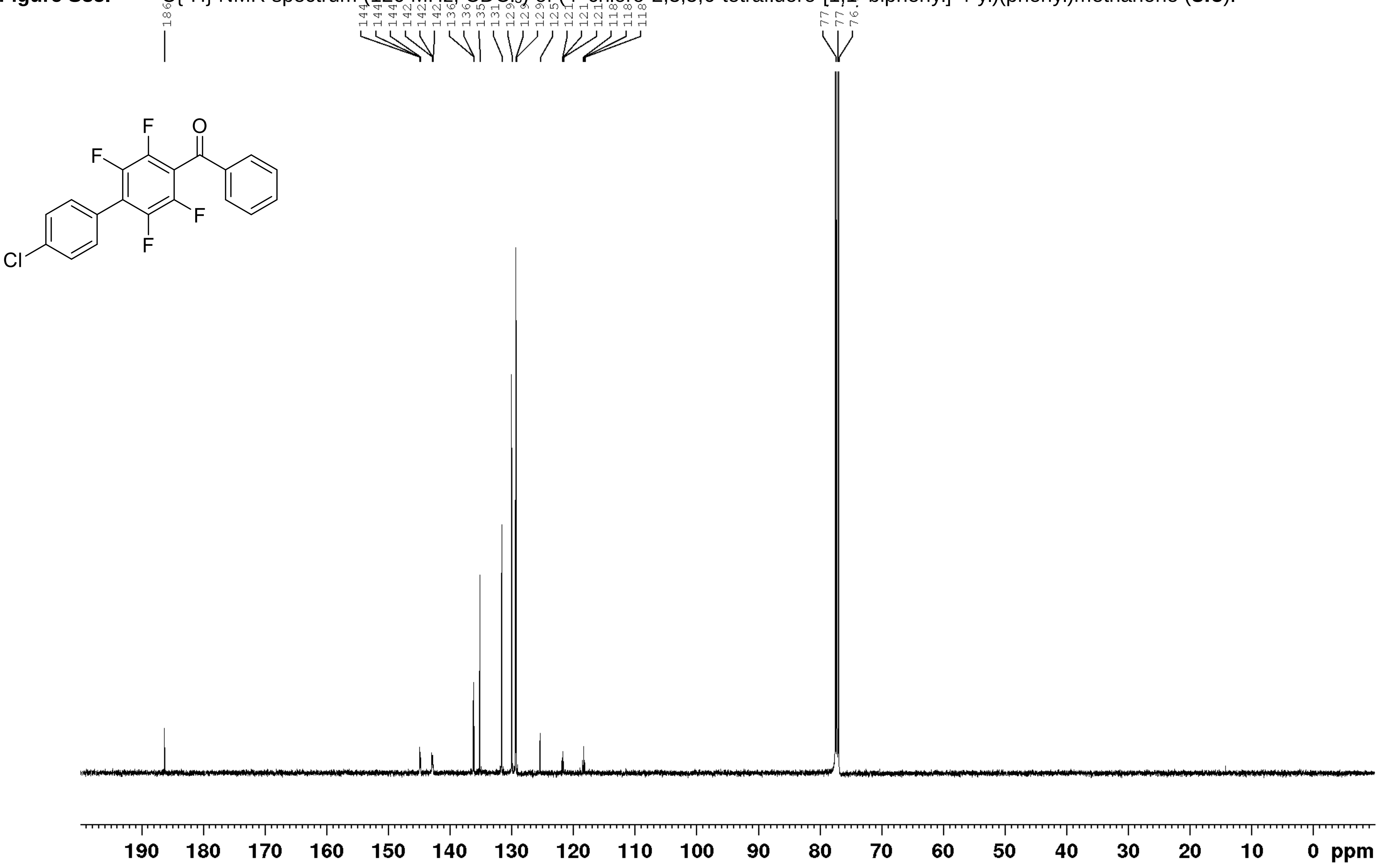

Figure S86. ^{19}F NMR spectrum (471 MHz, $CDCl_3$) of (4'-chloro-2,3,5,6-tetrafluoro-[1,1'-biphenyl]-4-yl)(phenyl)methanone (**3ie**).

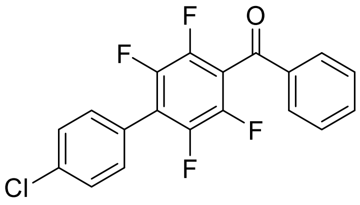

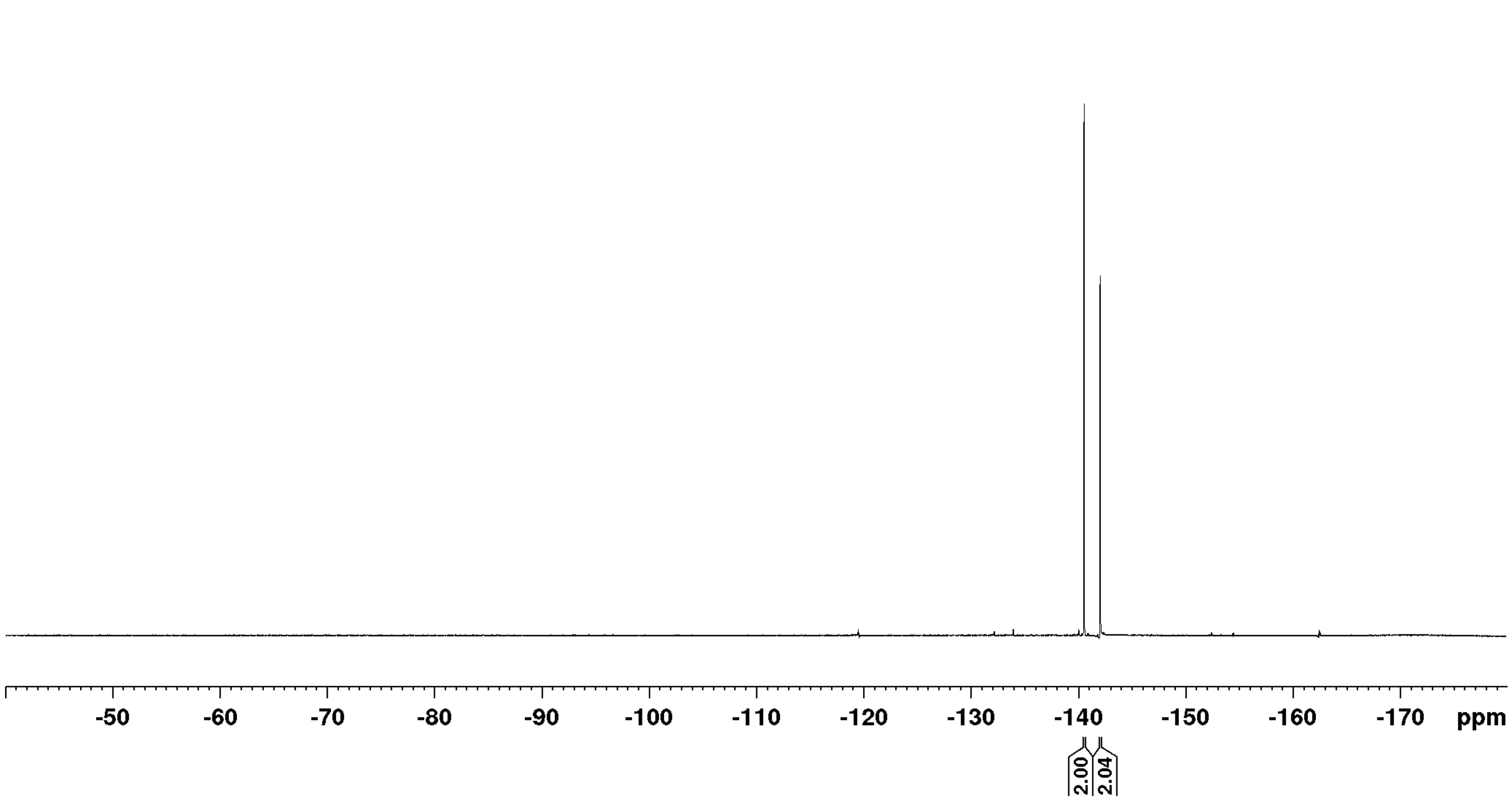

Figure S87. ^{1}H NMR spectrum (500 MHz, $CDCl_3$) of 2,3,5,6-tetrafluoro-4-(trifluoromethyl)-1,1'-biphenyl (**3bc**).

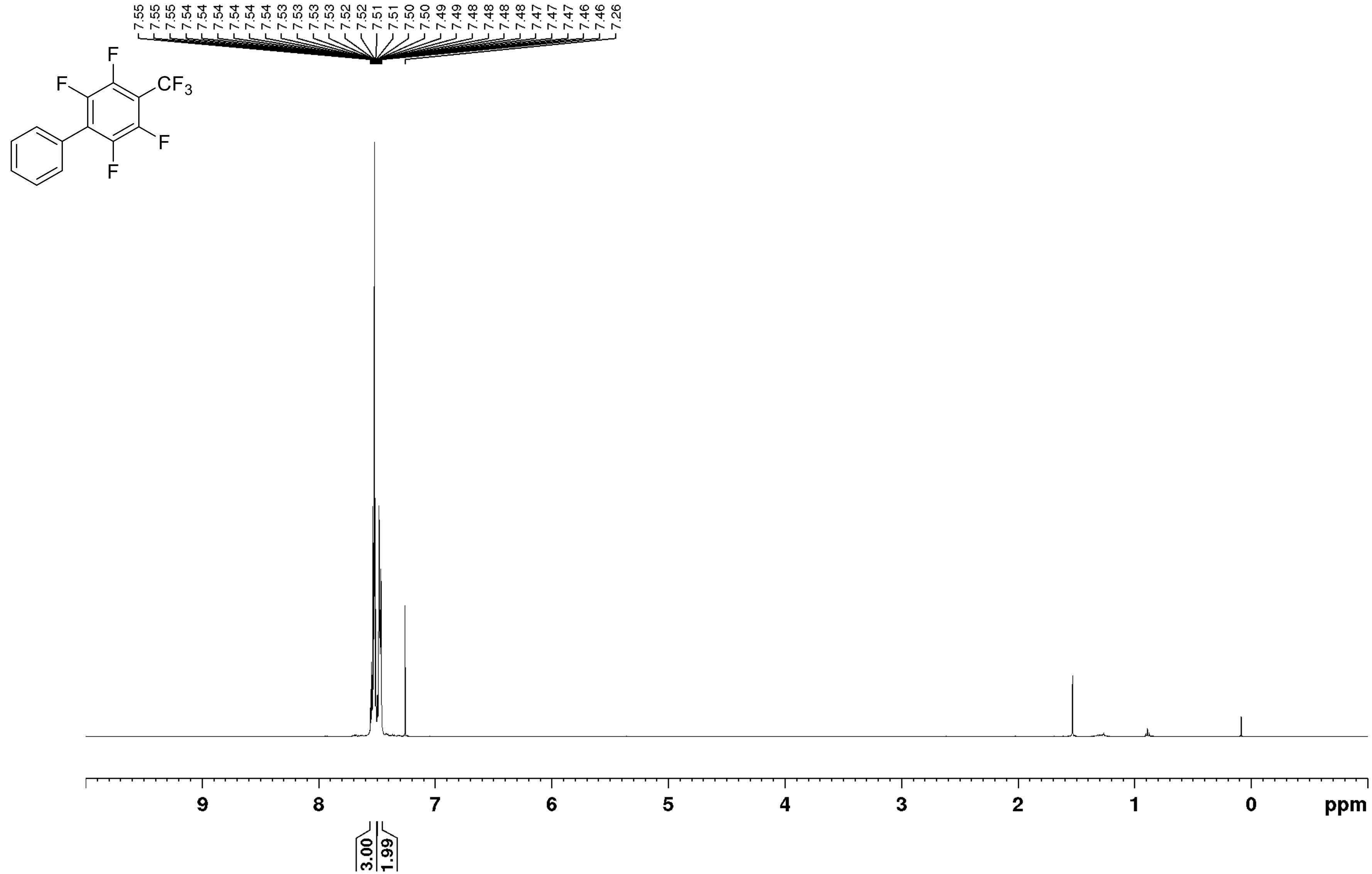

Figure S88. $^{13}C\{^{1}H\}$ NMR spectrum (126 MHz, $CDCl_3$) of 2,3,5,6-tetrafluoro-4-(trifluoromethyl)-1,1'-biphenyl (**3bc**).

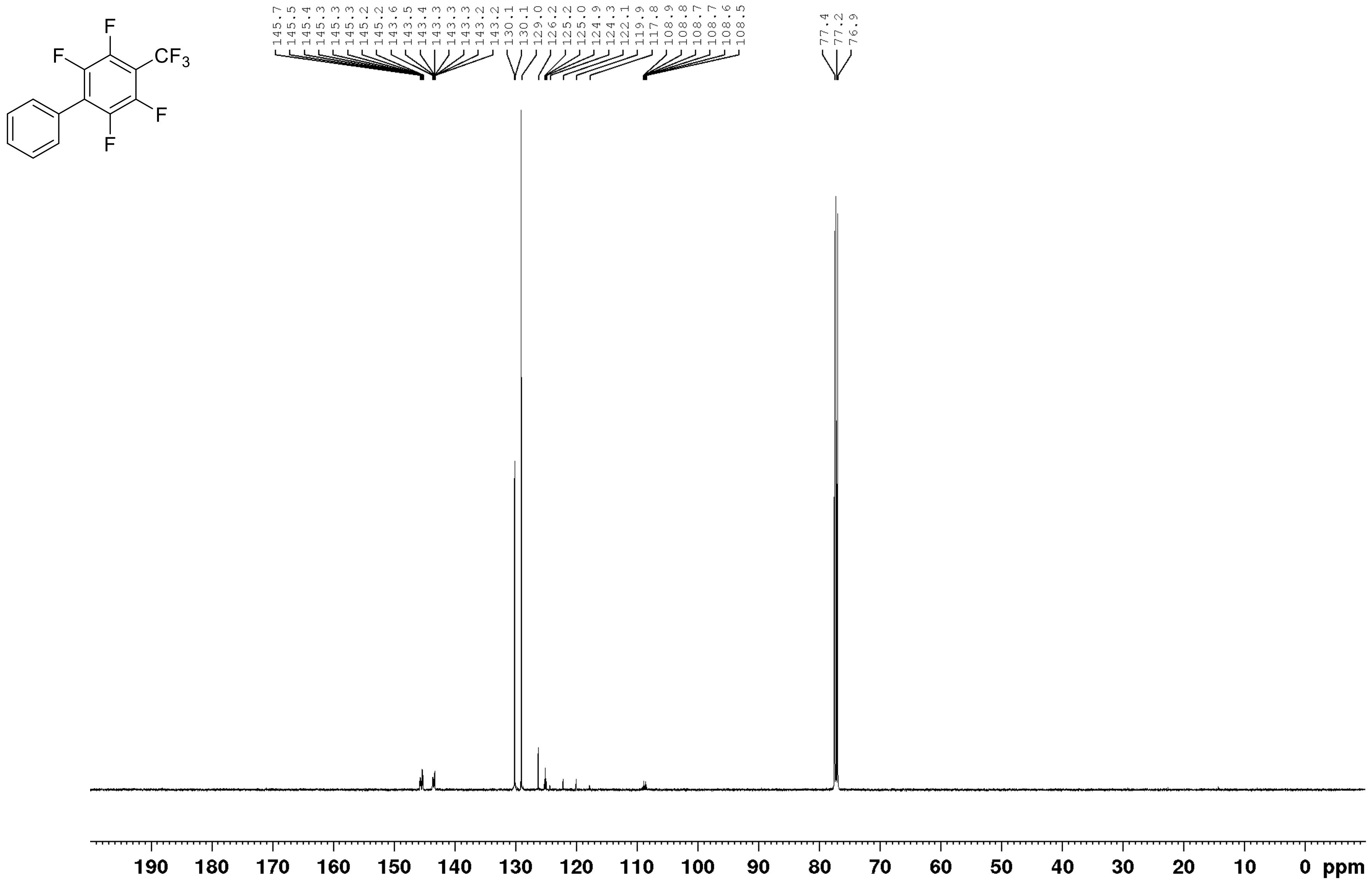

Figure S89. ^{19}F NMR spectrum (471 MHz, $CDCl_3$) of 2,3,5,6-tetrafluoro-4-(trifluoromethyl)-1,1'-biphenyl (**3bc**).

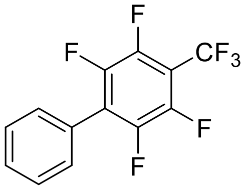

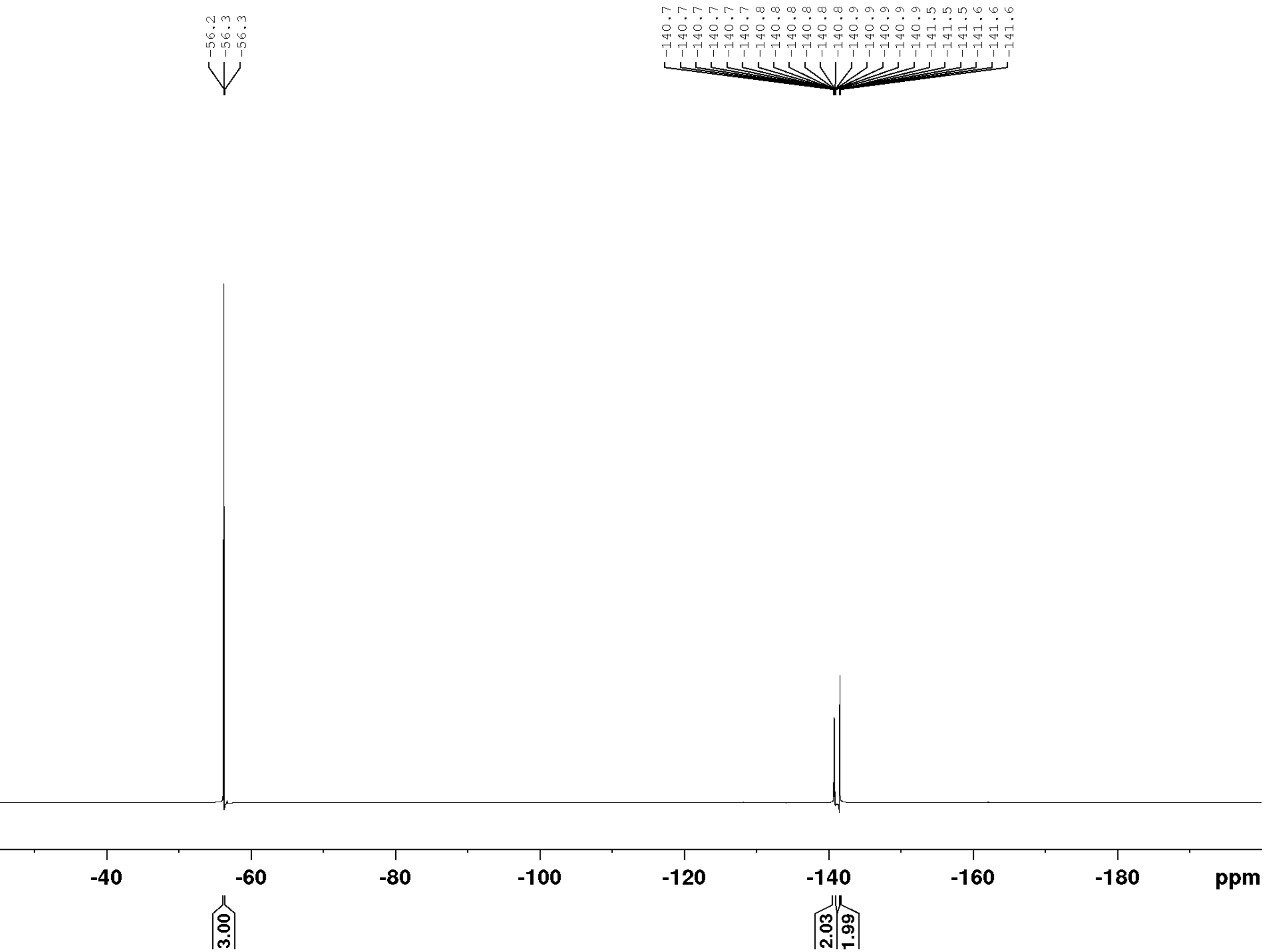

Figure S90. ^{1}H NMR spectrum (500 MHz, $CDCl_3$) of 4'-chloro-2,3,5,6-tetrafluoro-4-(trifluoromethyl)-1,1'-biphenyl (**3ic**).

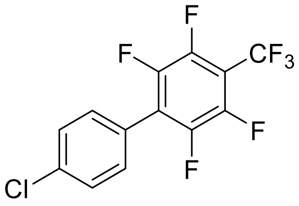

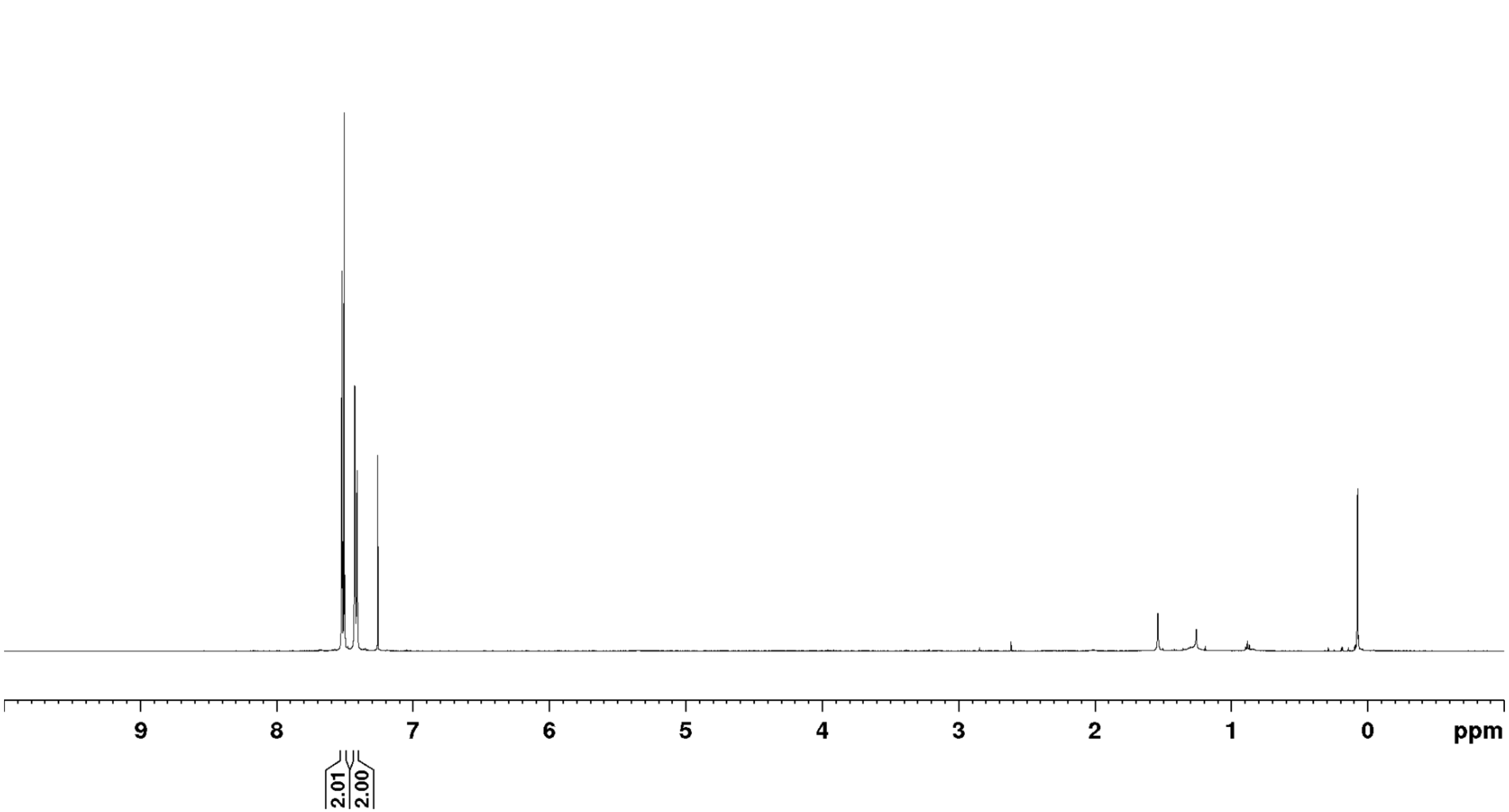

Figure S91. $^{13}C\{^{1}H\}$ NMR spectrum (126 MHz, $CDCl_3$) of 4'-chloro-2,3,5,6-tetrafluoro-4-(trifluoromethyl)-1,1'-biphenyl (**3ic**).

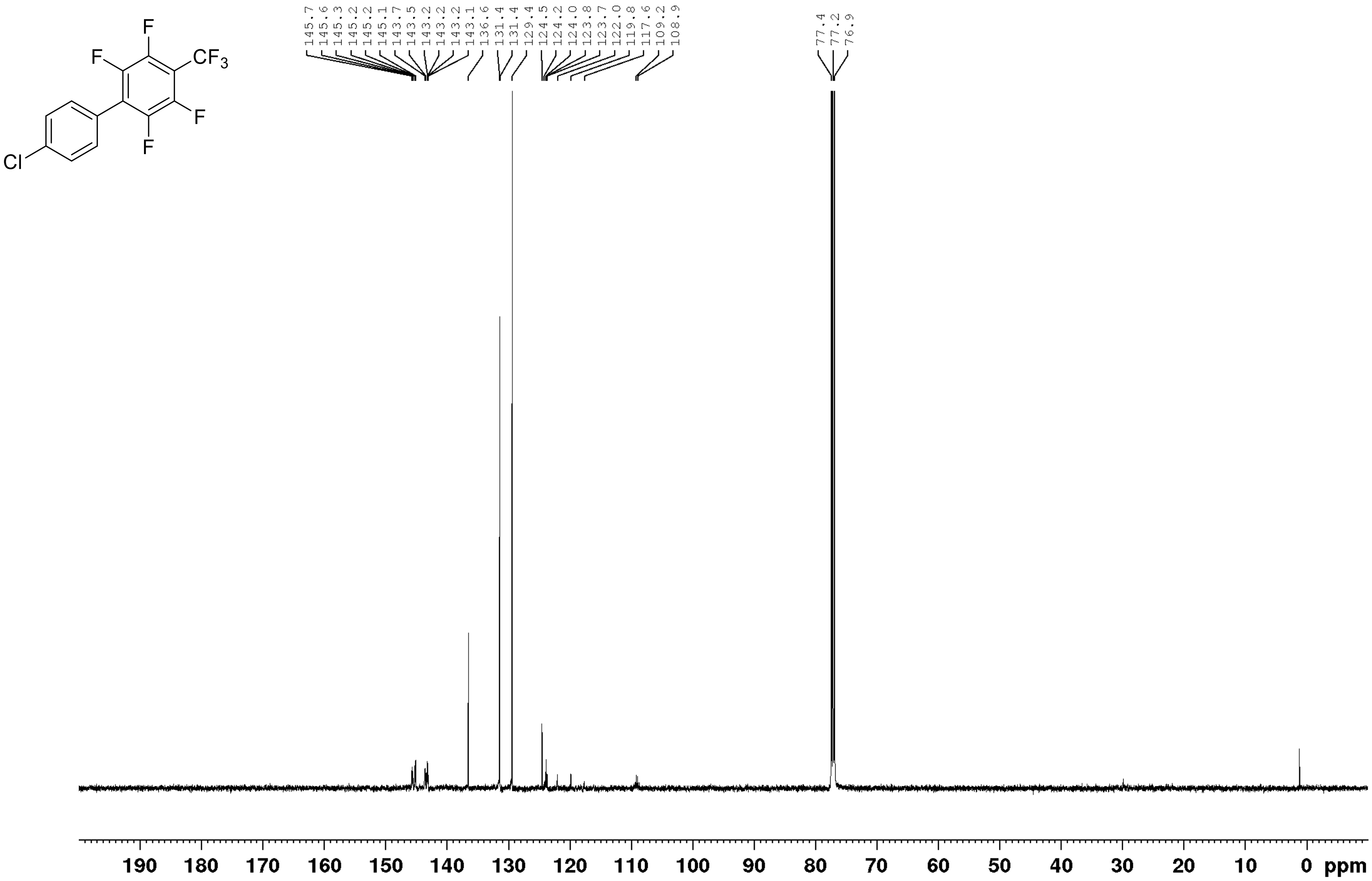

Figure S92. ^{19}F NMR spectrum (471 MHz, $CDCl_3$) of 4'-chloro-2,3,5,6-tetrafluoro-4-(trifluoromethyl)-1,1'-biphenyl (**3ic**).

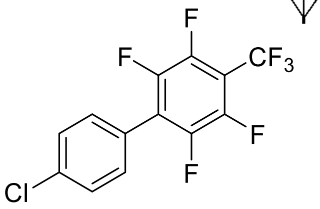

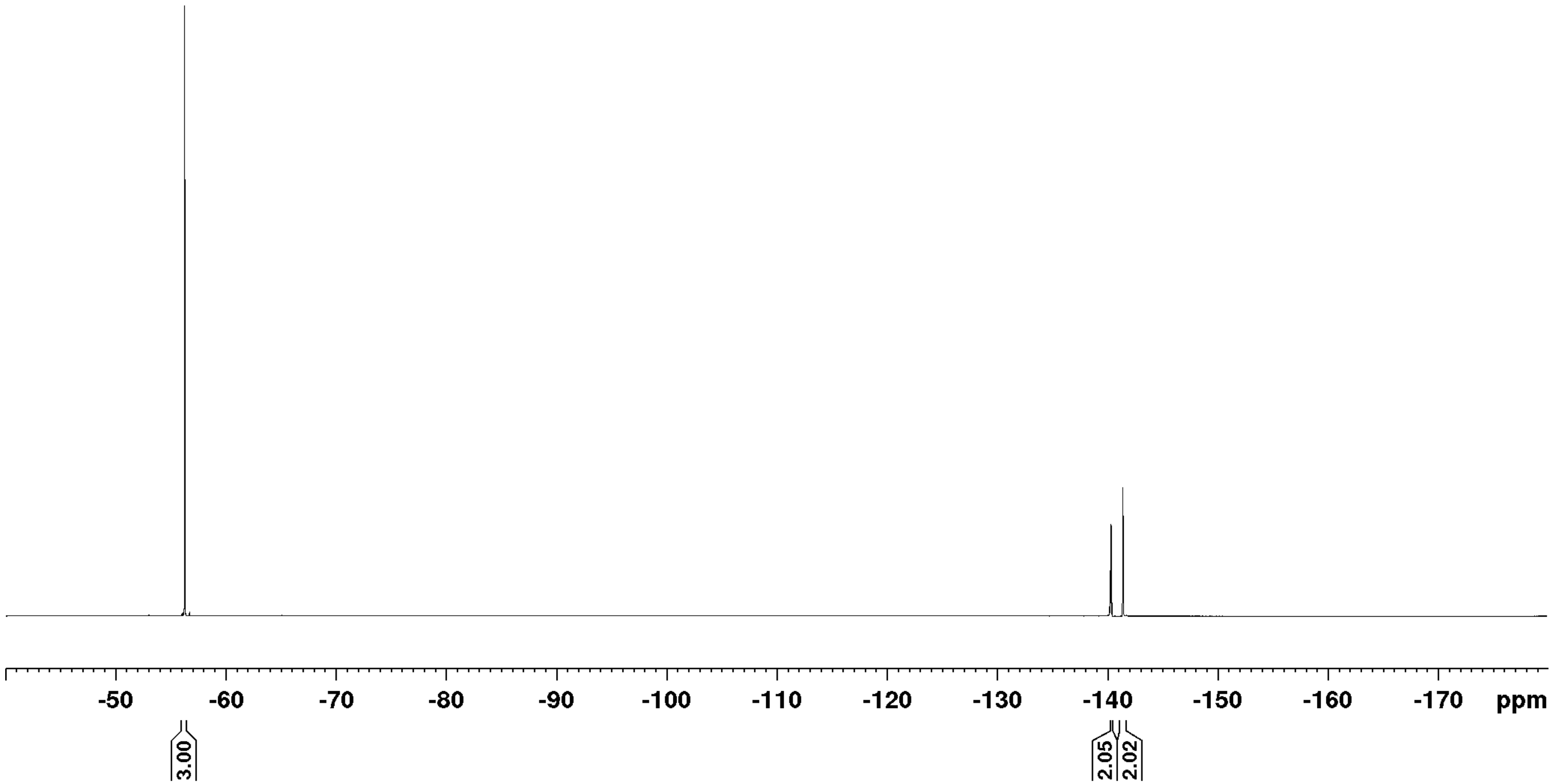

Figure S93. ^1H NMR spectrum (500 MHz, $CDCl_3$) of 2,2',3,5,6-pentafluoro-[1,1'-biphenyl]-4-carbonitrile (**3ld**).

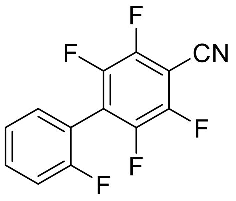

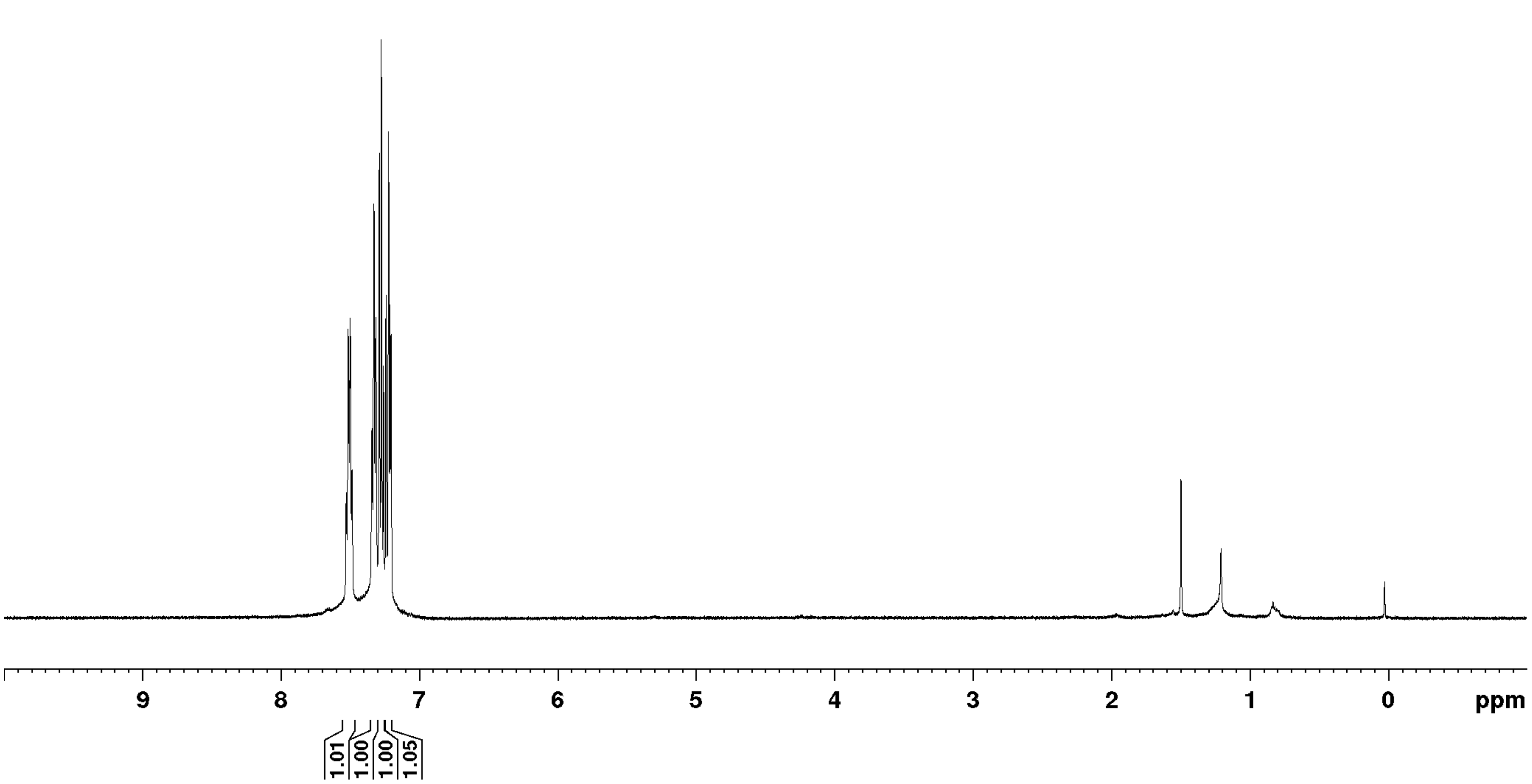

Figure S94. $^{13}C\{^{1}H\}$ NMR spectrum (126 MHz, $CDCl_3$) of 2,2',3,5,6-pentafluoro-[1,1'-biphenyl]-4-carbonitrile (**3ld**).

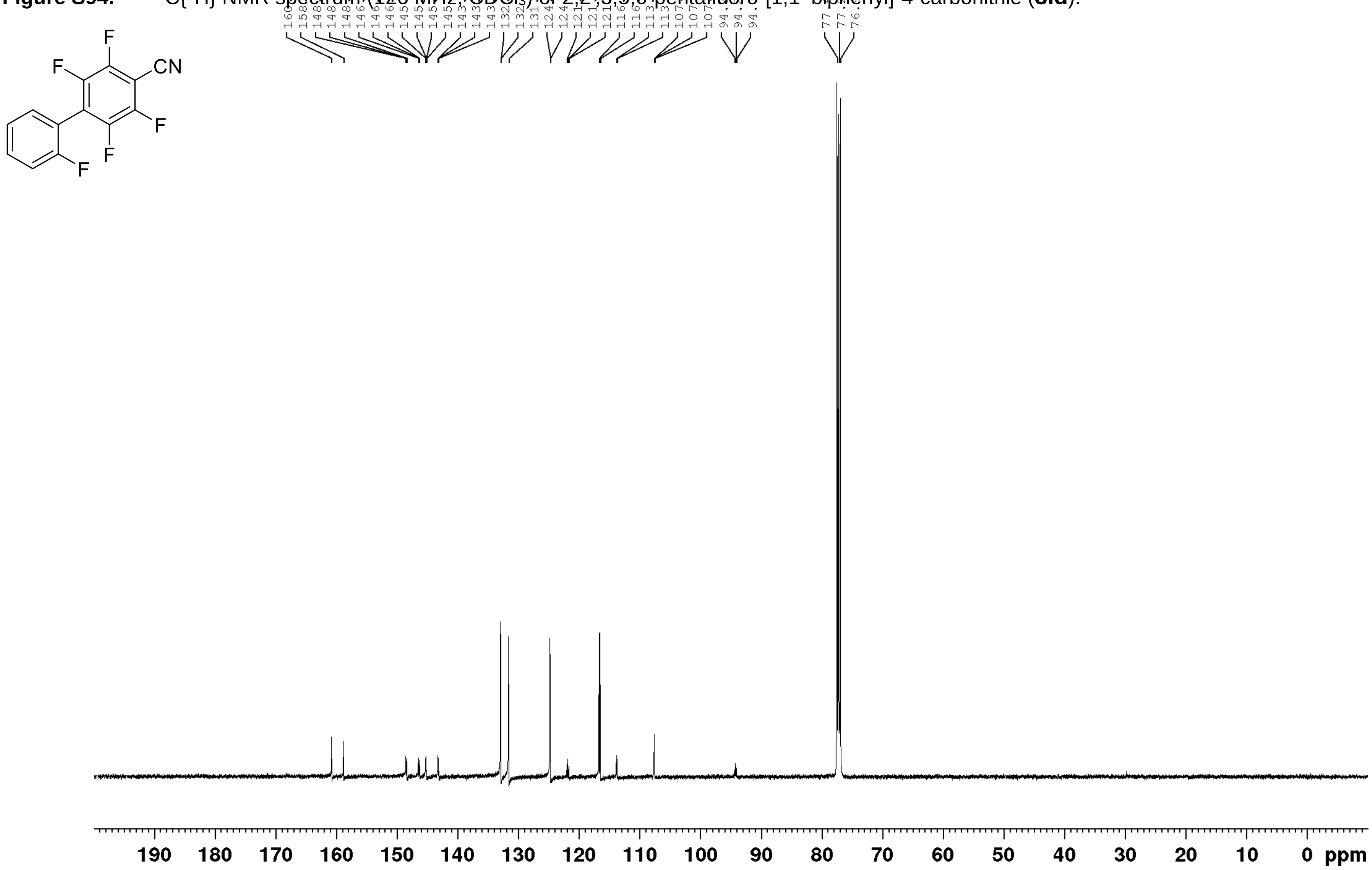

Figure S95. ^{19}F NMR spectrum (471 MHz, $CDCl_3$) of 2,2',3,5,6-pentafluoro-[1,1'-biphenyl]-4-carbonitrile (**3ld**).

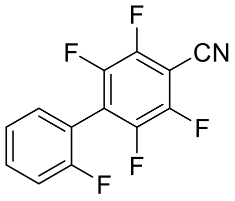

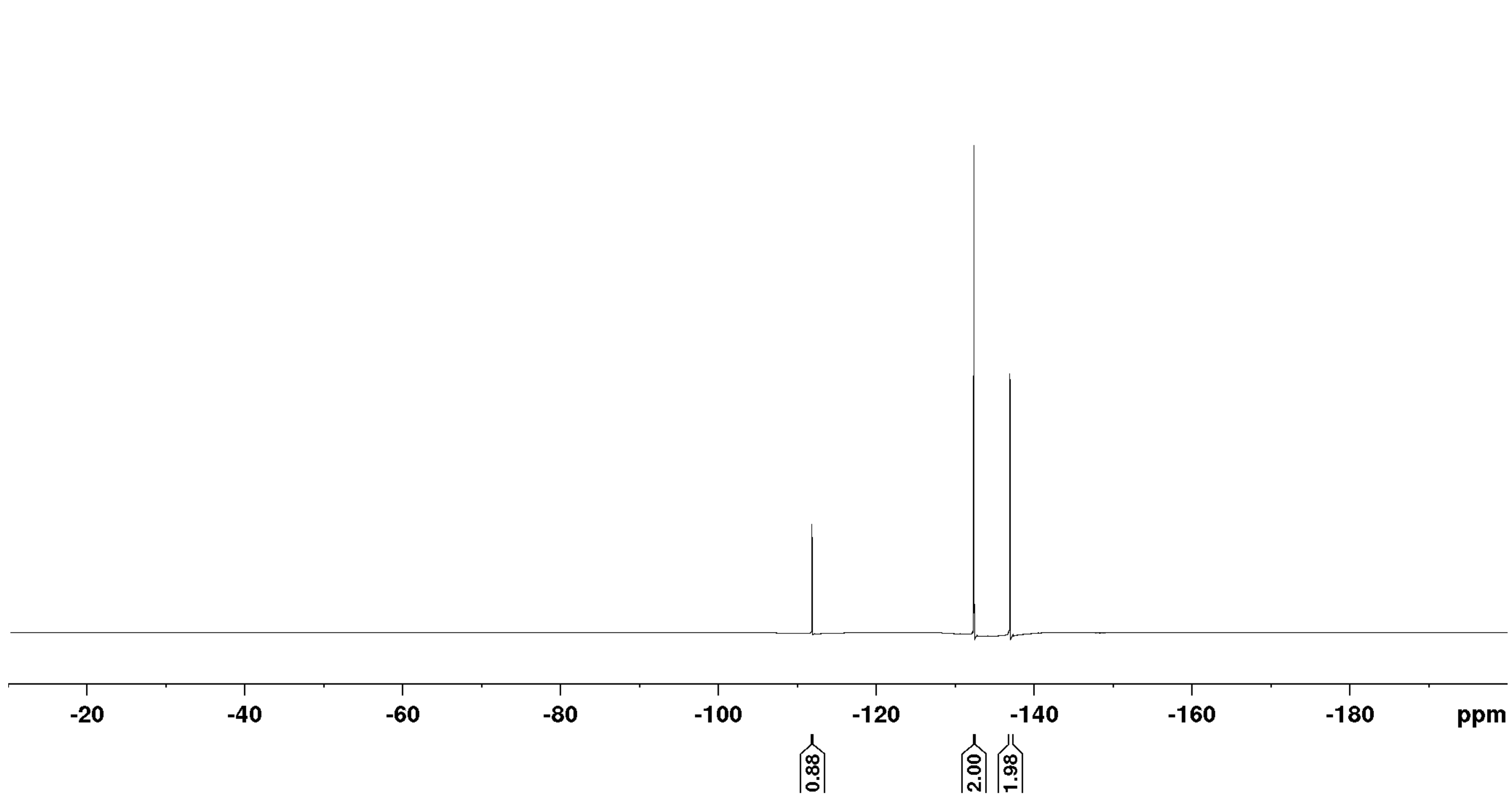

Figure S96. ^{1}H NMR spectrum (700 MHz, $CDCl_3$) of 2-(4-bromophenyl)-1,3,4,5,6,7,8-heptafluoronaphthalene (**3hf**).

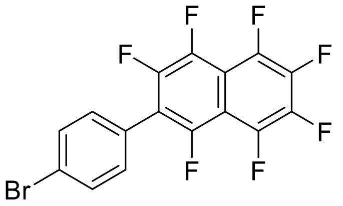

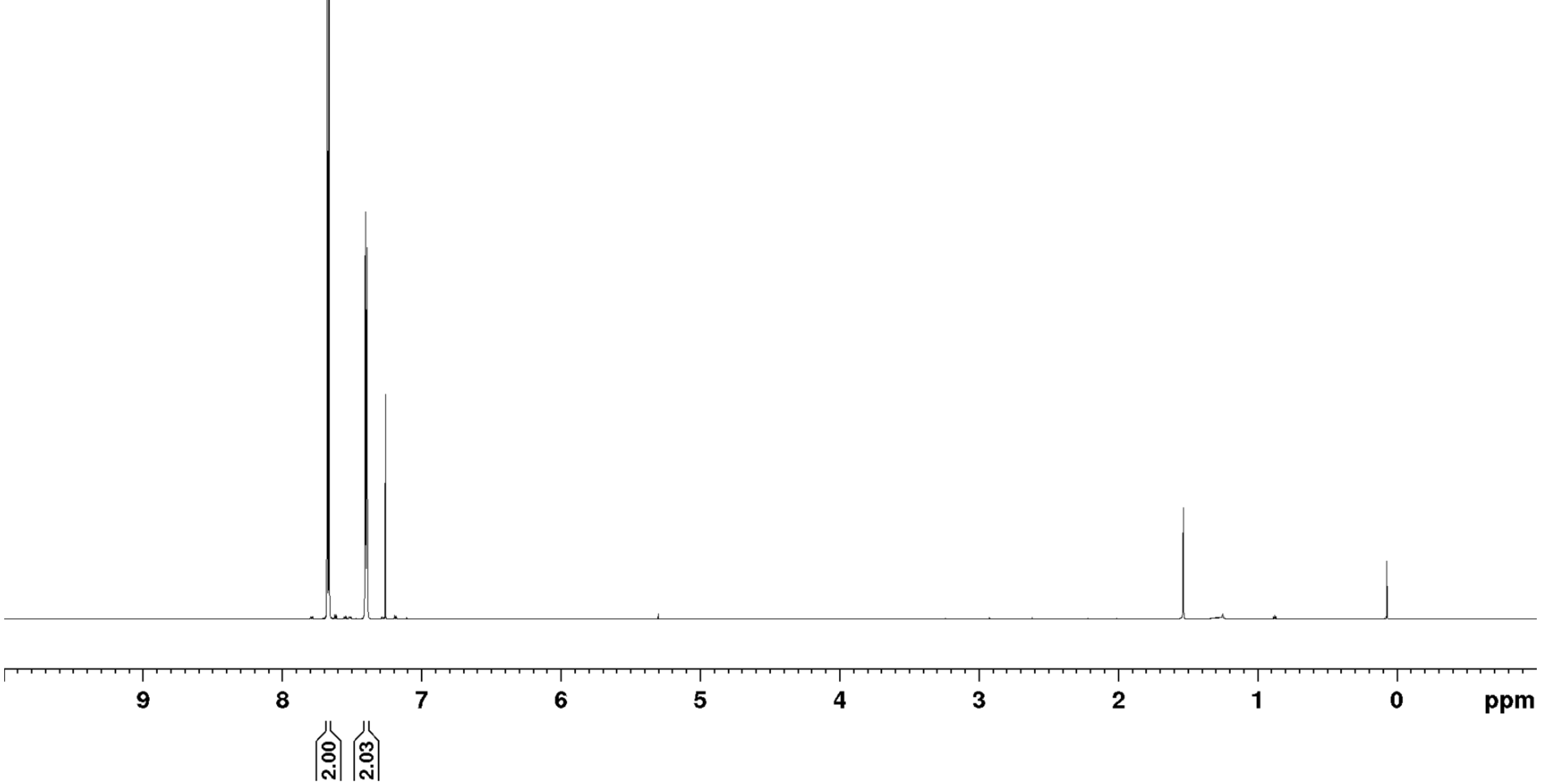

Figure S97. $^{13}C\{^{1}H\}$ NMR spectrum (176 MHz, $CDCl_3$) of 2-(4-bromophenyl)-1,3,4,5,6,7,8-heptafluoronaphthalene (**3hf**).

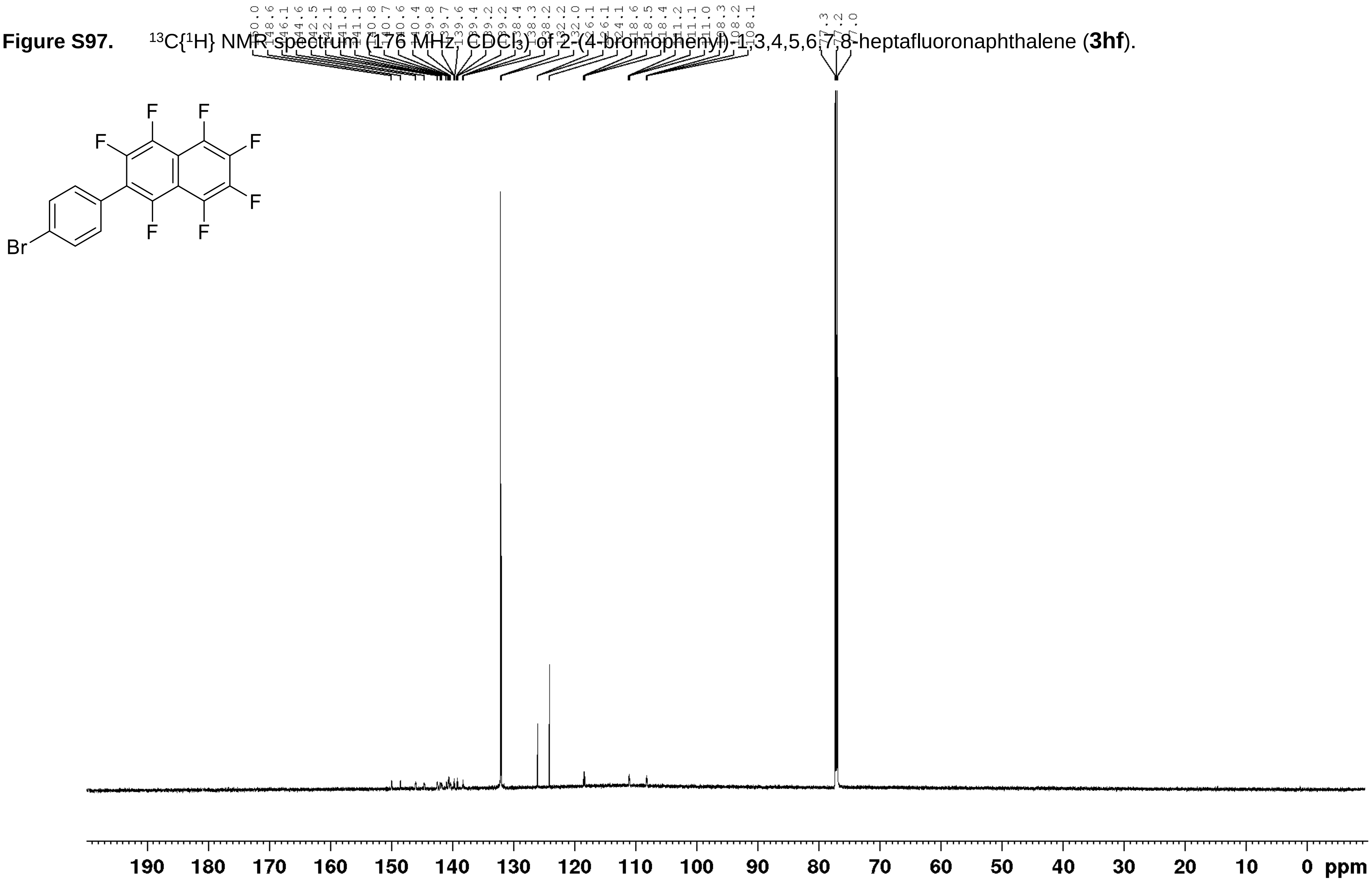

Figure S98. ^{19}F NMR spectrum (659 MHz, $CDCl_3$) of 2-(4-bromophenyl)-1,3,4,5,6,7,8-heptafluoronaphthalene (**3hf**).

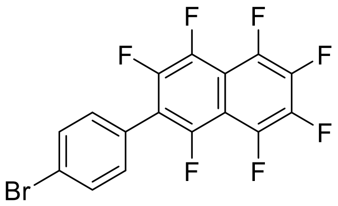

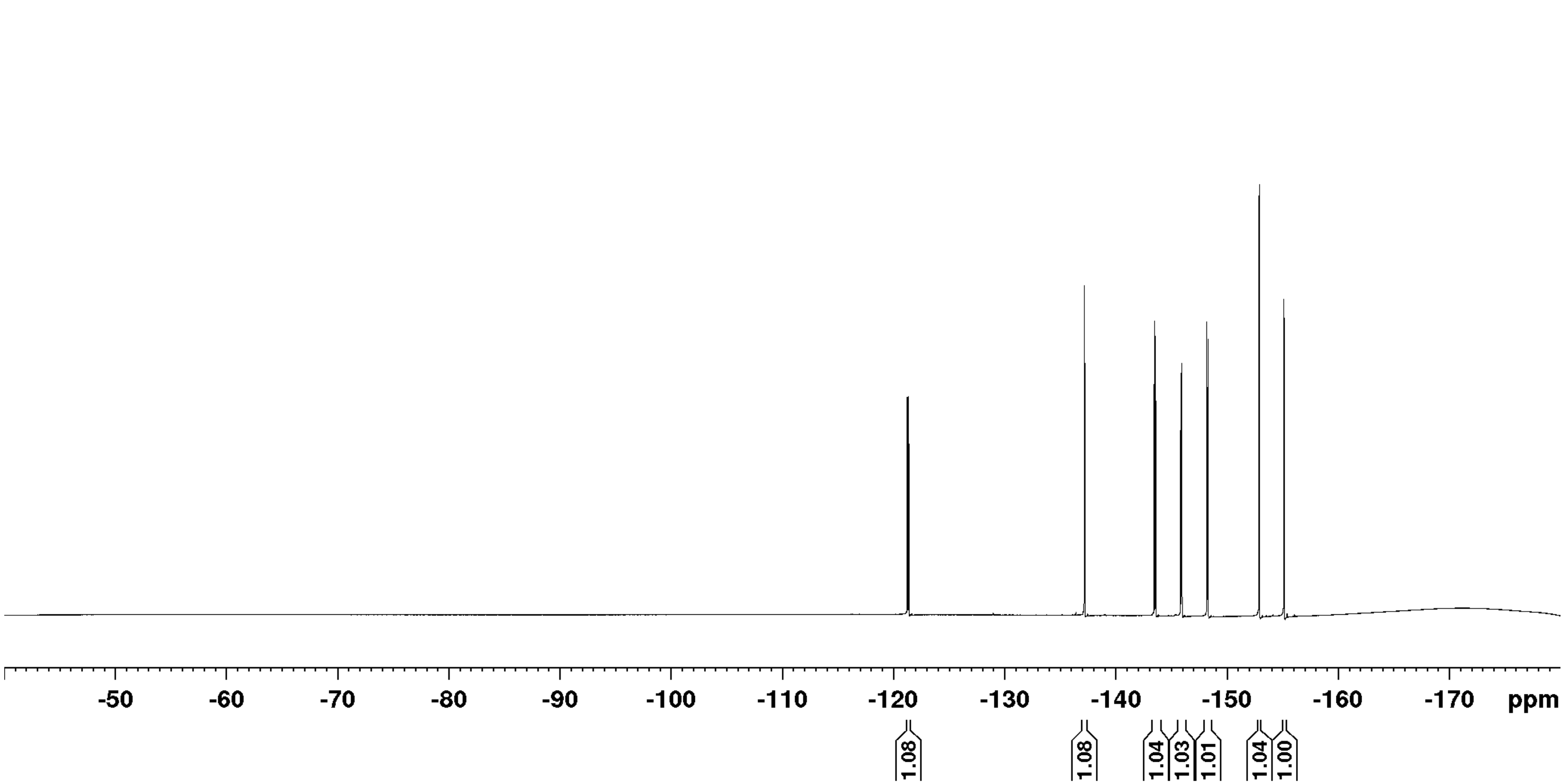

Figure S99. ^{1}H NMR spectrum (500 MHz, $CDCl_3$) of 2,2',3,3',4,5,5',6,6'-nonafluoro-4''-methoxy-1,1':4',1''-terphenyl (**3cg**).

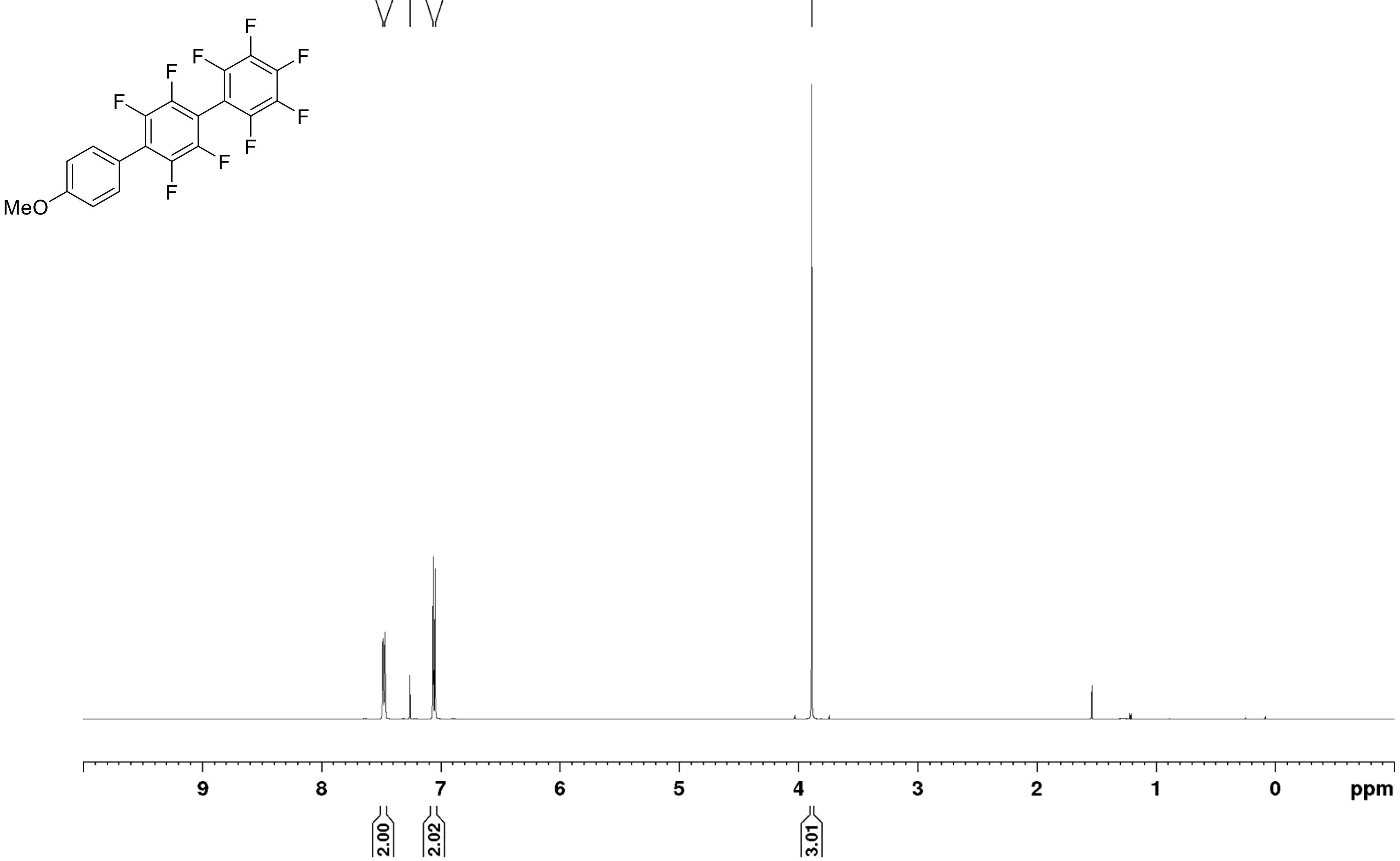

Figure S100. $^{13}C\{^1H\}$ NMR spectrum (126 MHz, $CDCl_3$) of 2,2',3,3',4,5,5',6,6'-nonafluoro-4''-methoxy-1,1':4',1''-terphenyl (**3cg**).

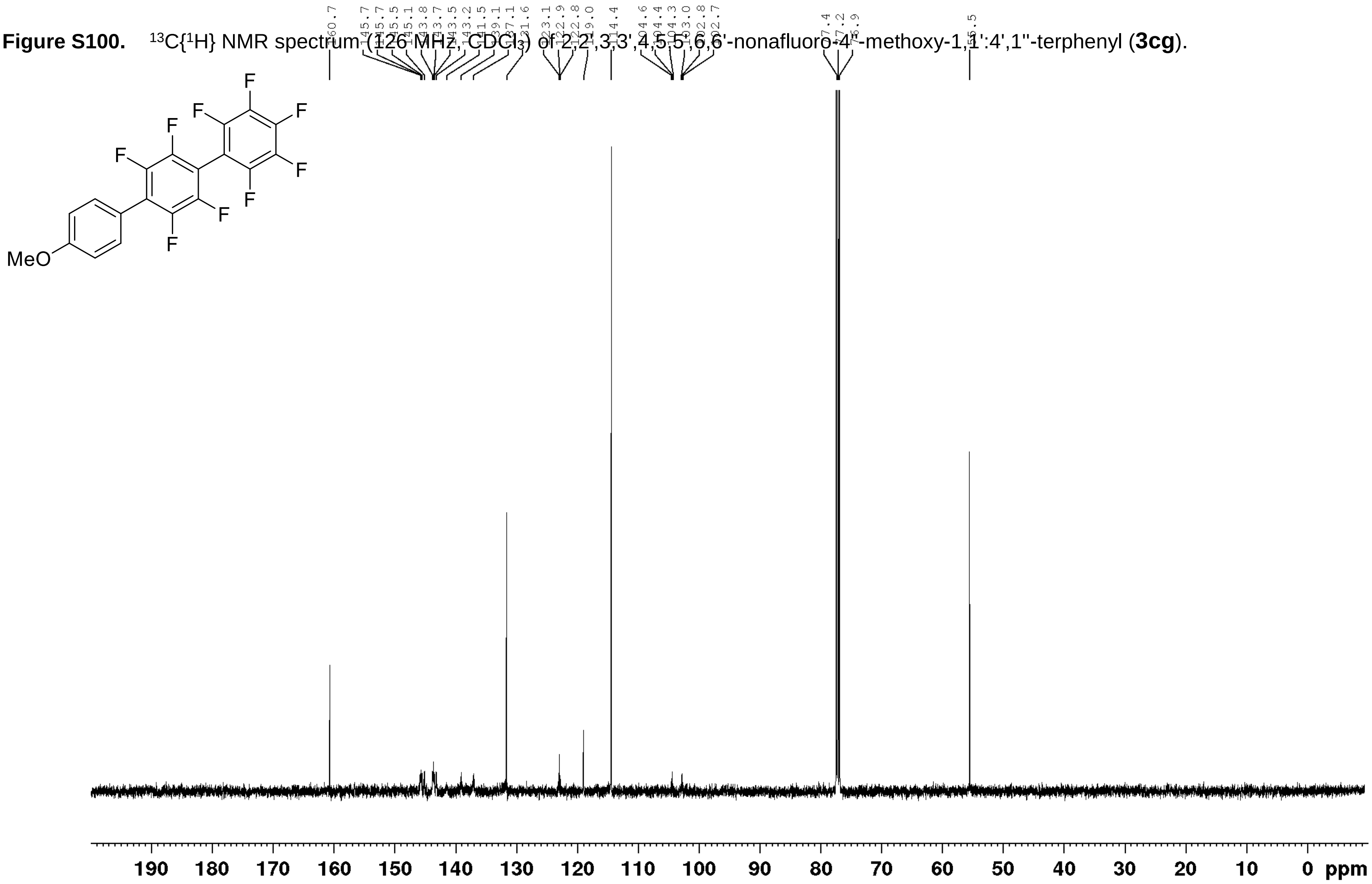

Figure S101. ^{19}F NMR spectrum (471 MHz, $CDCl_3$) of 2,2',3,3',4,5,5',6,6'-nonafluoro-4''-methoxy-1,1':4',1''-terphenyl (**3cg**).

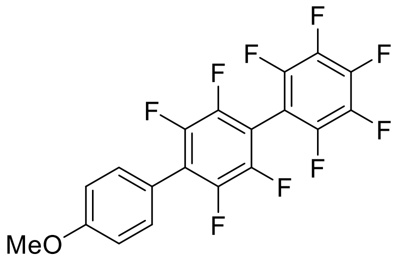

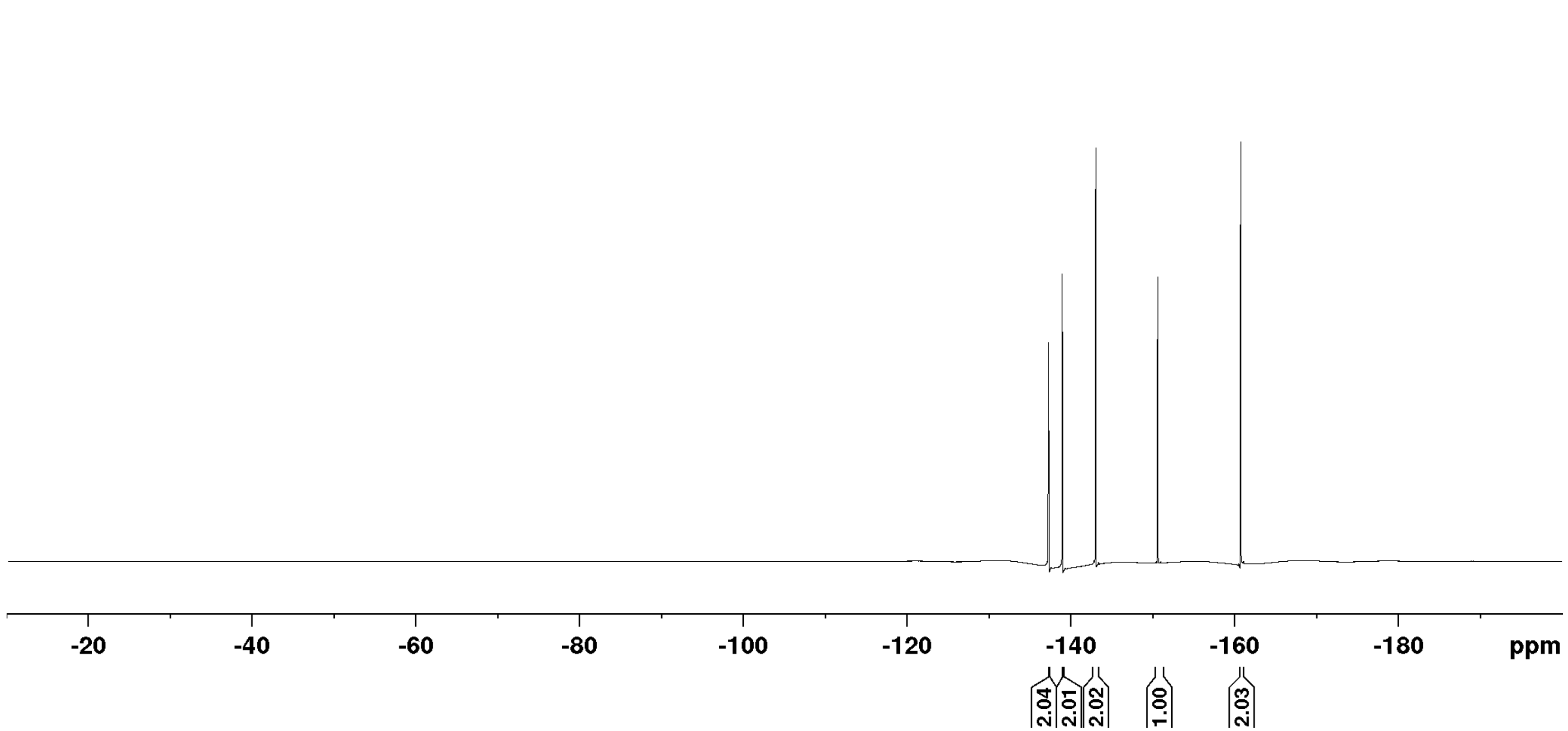

9.3 Diversified Coupling Products

Figure S102. ^{1}H NMR spectrum (500 MHz, $CDCl_3$) of (*E*)-2,3,5,6-tetrafluoro-4'-methoxy-4-styryl-1,1'-biphenyl (**7cka**).

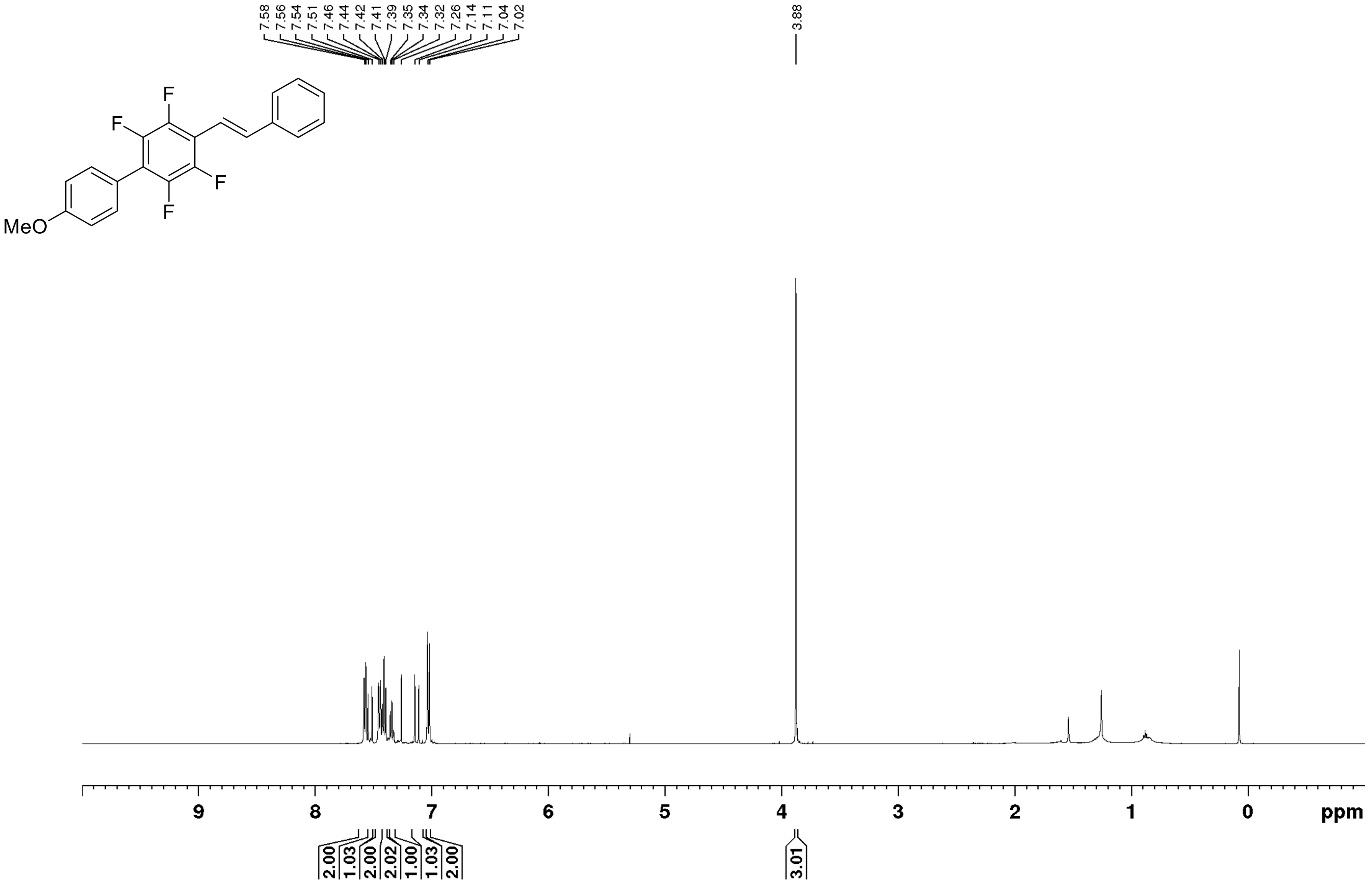

Figure S103. $^{13}C\{^{1}H\}$ NMR spectrum (126 MHz, $CDCl_3$) (*E*)-2,3,5,6-tetrafluoro-4'-methoxy-4-styryl-1,1'-biphenyl (**7cka**).

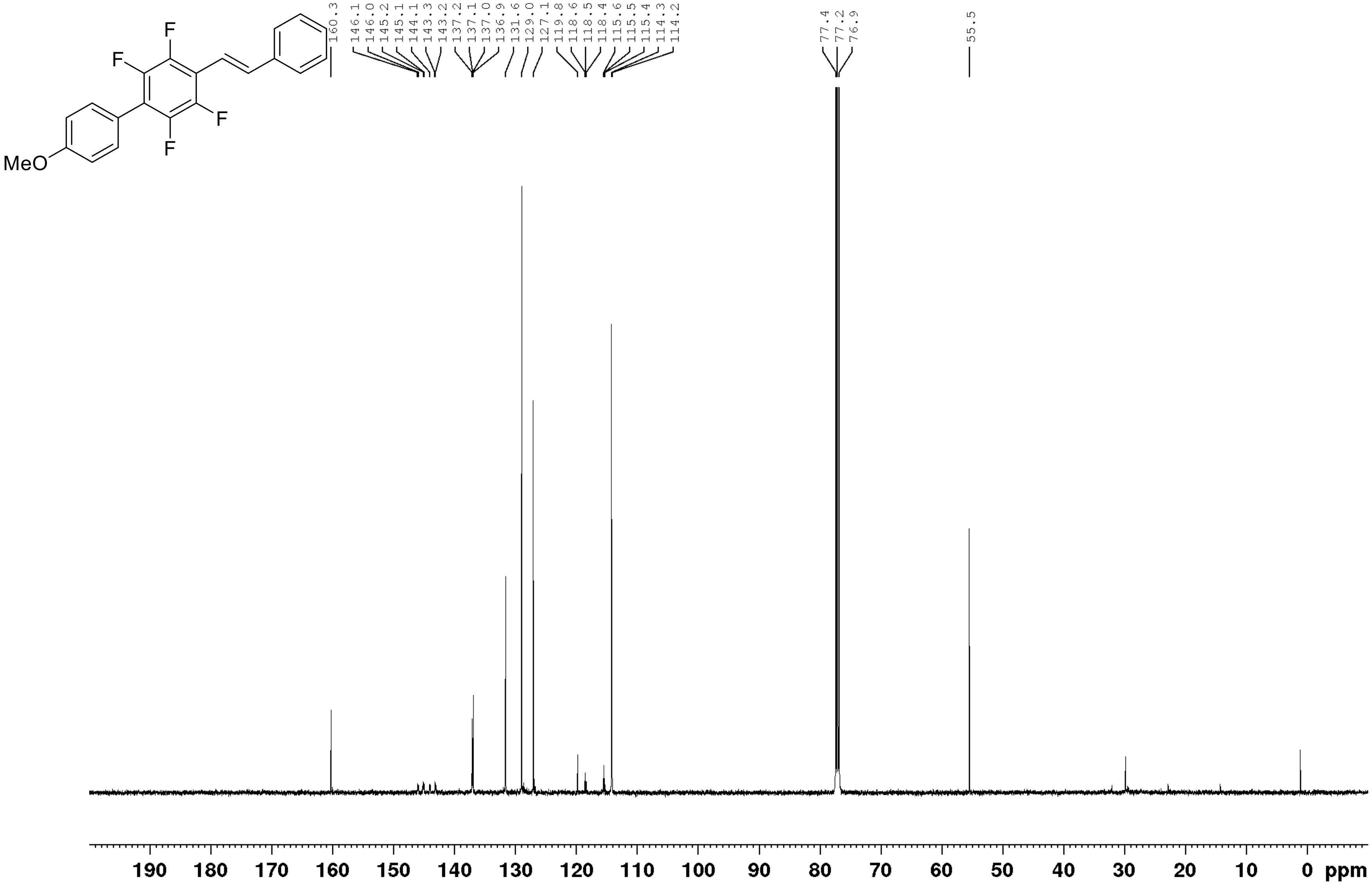

Figure S104. ^{19}F NMR spectrum (471 MHz, $CDCl_3$) of (*E*)-2,3,5,6-tetrafluoro-4'-methoxy-4-styryl-1,1'-biphenyl (**7cka**).

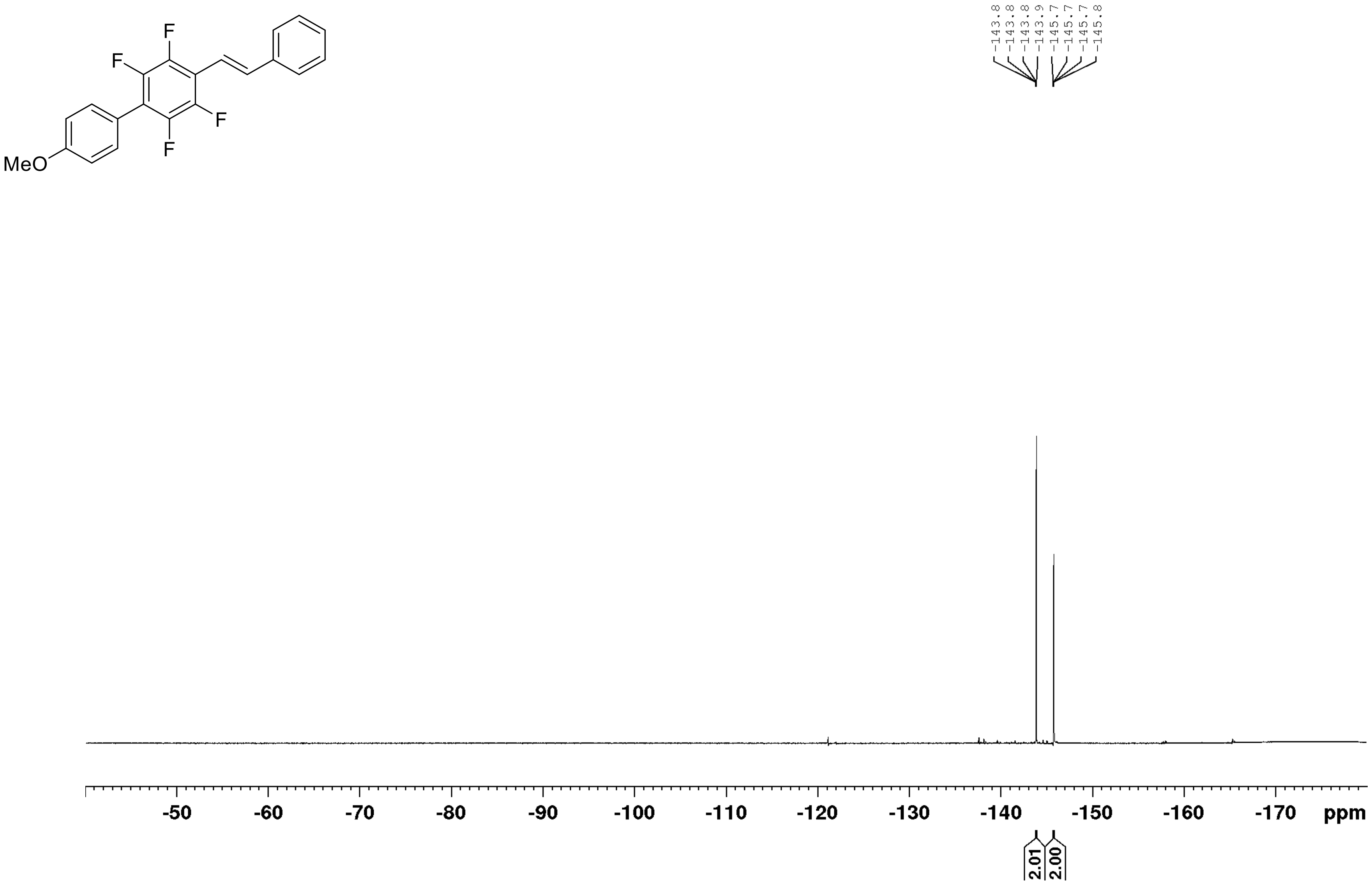

Figure S105. ^{1}H NMR spectrum (500 MHz, CD_2Cl_2) of methyl (*E*)-4-(2-(4'-cyano-2,3,5,6-tetrafluoro-[1,1'-biphenyl]-4-yl)vinyl)benzoate (**7dkb**).

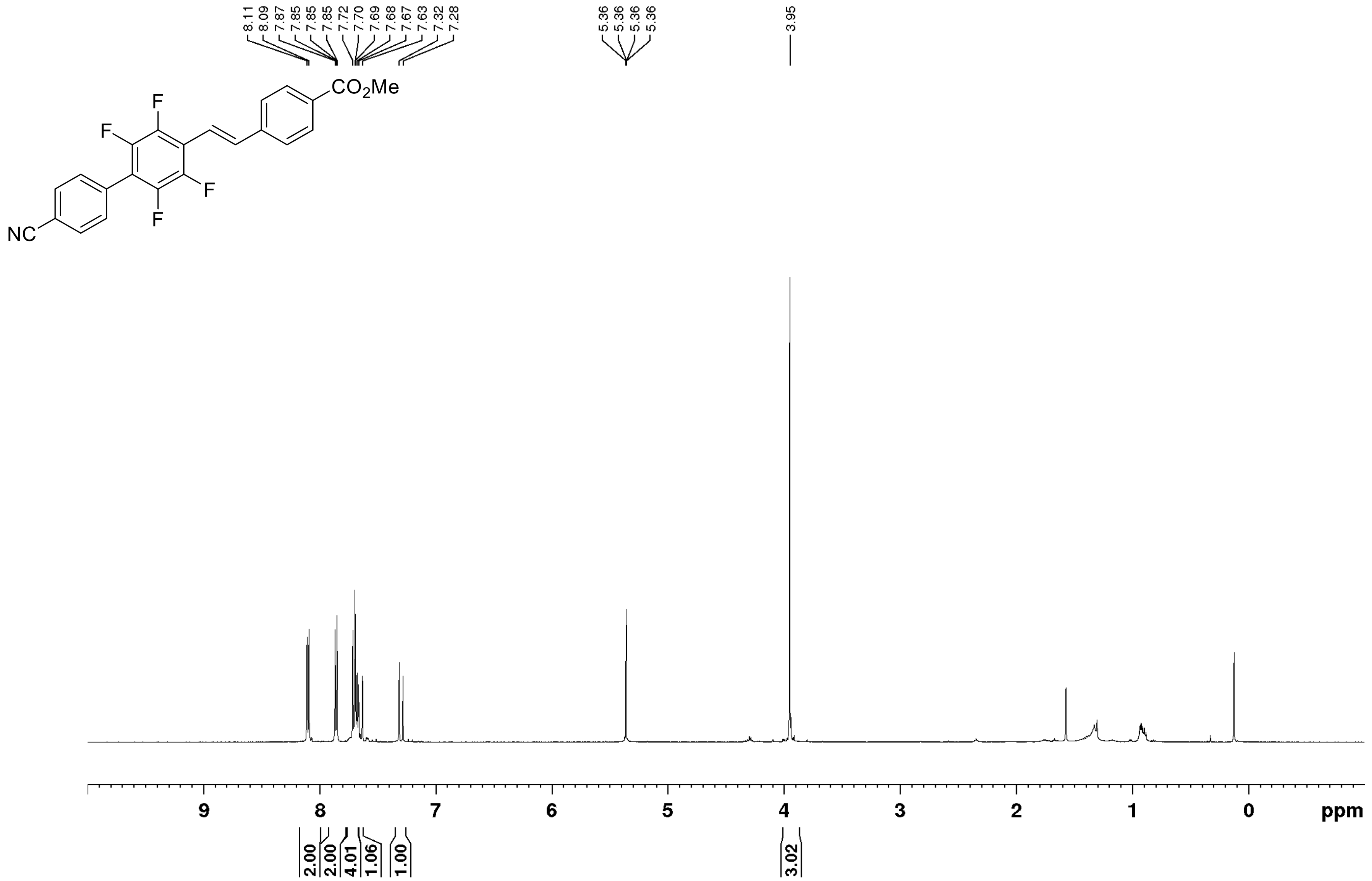

Figure S106. $^{13}C\{^1H\}$ NMR spectrum (126 MHz, CD_2Cl_2) methyl (*E*)-4-(2-(4'-cyano-2,3,5,6-tetrafluoro-[1,1'-biphenyl]-4-yl)vinyl)benzoate (**7dkb**).

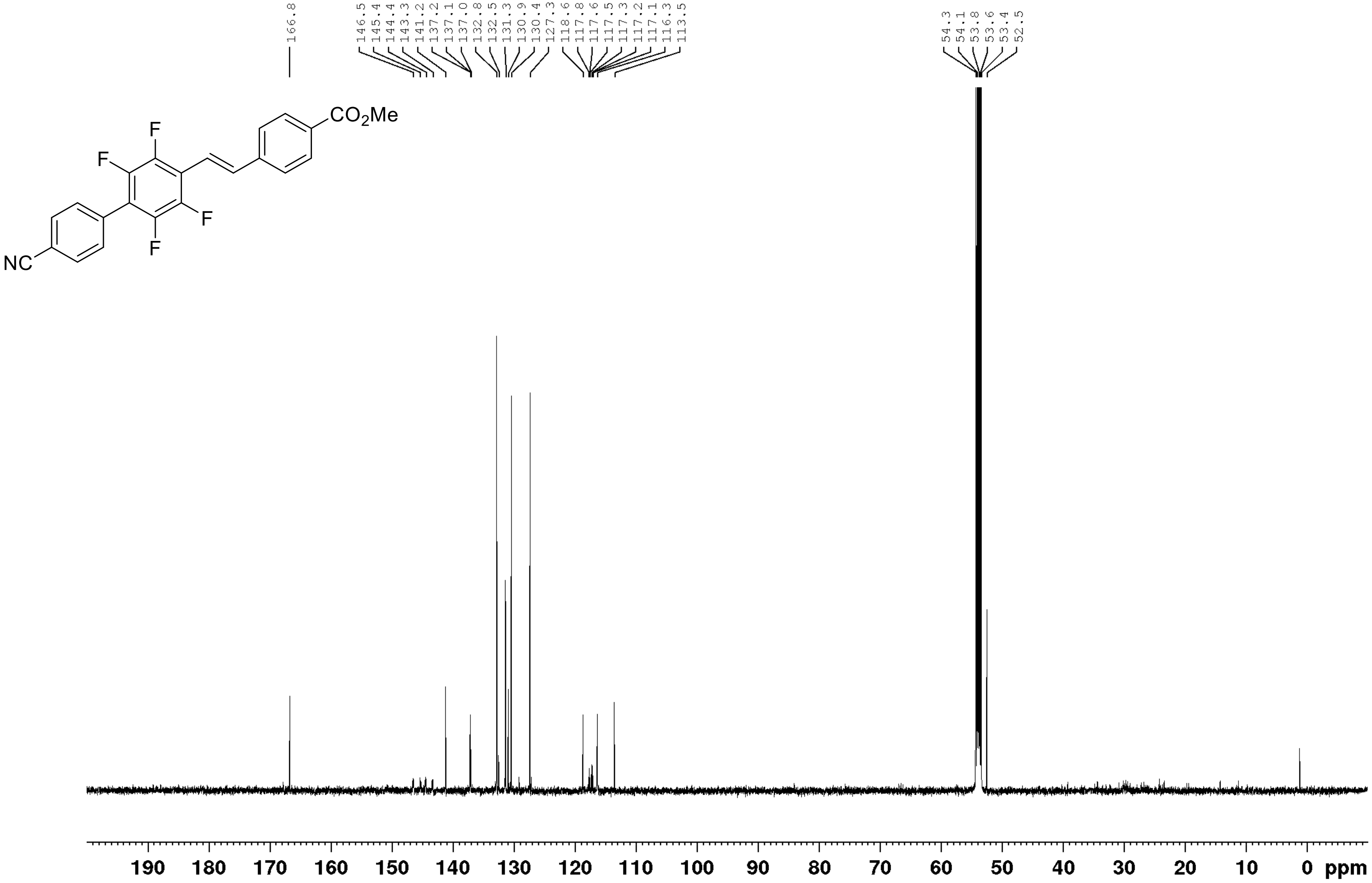

Figure S107. ^{19}F NMR spectrum (471 MHz, CD_2Cl_2) of methyl (*E*)-4-(2-(4'-cyano-2,3,5,6-tetrafluoro-[1,1'-biphenyl]-4-yl)vinyl)benzoate (**7dkb**).

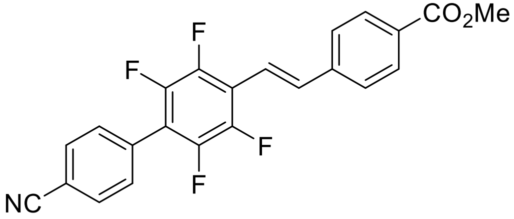

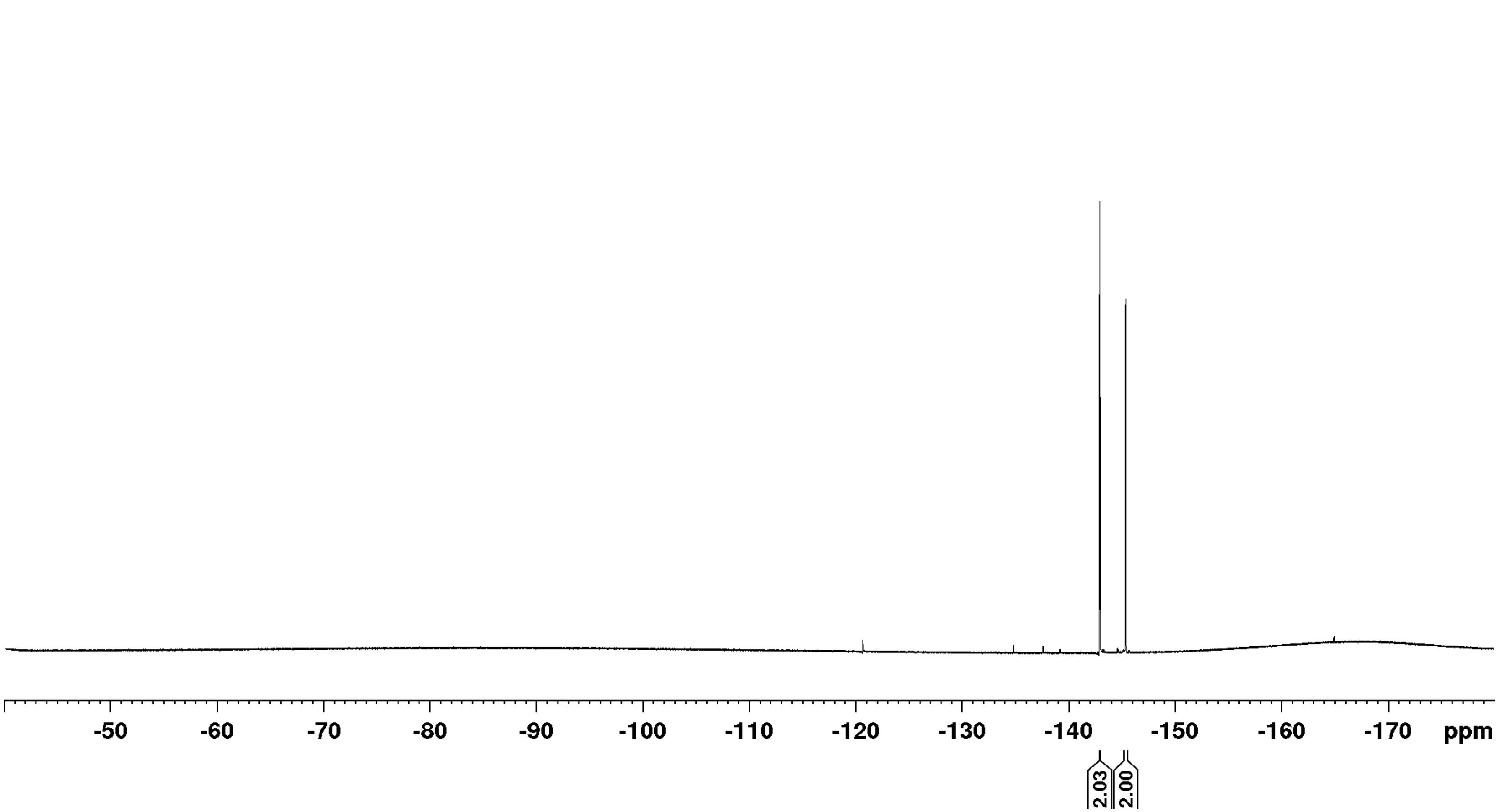

10 References

[S1] C. Chauvier, L. Finck, S. Hecht, M. Oestreich, *Organometallics* **2019**, *38*, 4679–4686.

[S2] W. C. Still, M. Kahn, A. Mitra, *J. Org. Chem.* **1978**, *43*, 2923–2925.

[S3] a) H. E. Gottlieb, V. Kotlyar, A. Nudelman, *J. Org. Chem.* **1997**, *62*, 7512–7515; b) G. R. Fulmer, A. J. M. Miller, N. H. Sherden, H. E. Gottlieb, A. Nudelman, B. M. Stoltz, J. E. Bercaw, K. I. Goldberg, *Organometallics* **2010**, *29*, 2176–2179.

[S4] R. K. Harris, E. D. Becker, S. M. Cabral de Menezes, R. Goodfellow, P. Granger, *Pure Appl. Chem.* **2001**, *73*, 1795–1818.

[S5] C. Chauvier, L. Finck, E. Irran, M. Oestreich, *Angew. Chem. Int. Ed.* **2020**, *59*, 12337–12341; *Angew. Chem.* **2020**, *132*, 12436–12440.

[S6] a) E. J. F. Klotz, T. D. W. Claridge, H. L. Anderson, *J. Am. Chem. Soc.* **2006**, *128*, 15374–15375; b) J. Atchison, S. Kamila, H. Nesbitt, K. A. Logan, D. M. Nicholas, C. Fowley, J. Davis, B. Callan, A. P. McHale, J. F. Callan, *Chem. Commun.* **2017**, *53*, 2009–2012.

[S7] a) N. E. Wurz, C. G. Daniliuc, F. Glorius, *Chem. Eur. J.* **2012**, *18*, 16297–16301; b) S. Dong, M. Frings, H. Cheng, J. Wen, D. Zhang, G. Raabe, C. Bolm, *J. Am. Chem. Soc.* **2016**, *138*, 2166–2169; c) V. R. Yatham, W. Harnying, D. Kootz, J.-M. Neudörfl, N. E. Schlörer, A. Berkessel, *J. Am. Chem. Soc.* **2016**, *138*, 2670–2677.

[S8] M. Simonetti, G. J. P. Perry, X. C. Cambeiro, F. Julia-Hernandez, J. N. Arokianathar, I. Larrosa, *J. Am. Chem. Soc.* **2016**, *138*, 3596–3606.

[S9] R. Shang, Y. Fu, Y. Wang, Q. Xu, H.-Z. Yu, L. Liu, *Angew. Chem. Int. Ed.* **2009**, *48*, 9350–9354.

[S10] A. Dahiya, C. Fricke, F. Schoenebeck, *J. Am. Chem. Soc.* **2020**, *142*, 7754–7759.

[S11] H.-Q. Do, R. M. K. Khan, O. Daugulius, *J. Am. Chem. Soc.* **2008**, *130*, 15185–15192.

[S12] A. Dewanji, R. F. Bülow, M. Rueping, *Org. Lett.* **2020**, *22*, 1611–1617.

[S13] T. Fuchikami, M. Yatabe, I. Ojima, *Synthesis* **1981**, *1981*, 365–366.
